# Prebiotic Catalytic Peptide Ligation Yields Proteinogenic Peptides by Intramolecular Amide Catalyzed Hydrolysis Facilitating Regioselective Lysine Ligation in Neutral Water

**DOI:** 10.1021/jacs.2c03486

**Published:** 2022-05-31

**Authors:** Jyoti Singh, Daniel Whitaker, Benjamin Thoma, Saidul Islam, Callum S. Foden, Abil E. Aliev, Tom D. Sheppard, Matthew W. Powner

**Affiliations:** ‡ Department of Chemistry, University College London, 20 Gordon Street, London WC1H 0AJ, United Kingdom; § Department of Chemistry, King’s College London, 7 Trinity Street, London SE1 1DB, United Kingdom

## Abstract

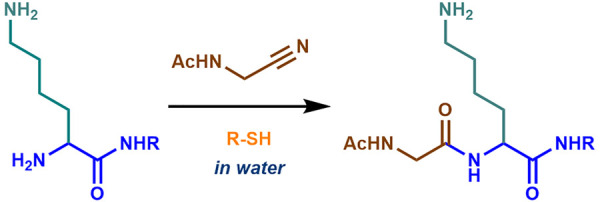

The prebiotic origin of catalyst-controlled peptide synthesis is fundamental to understanding the emergence of life. Building on our recent discovery that thiols catalyze the ligation of amino acids, amides, and peptides with amidonitriles in neutral water, we demonstrate the outcome of ligation depends on pH and that high p*K*_a_ primary thiols are the ideal catalysts. While the most rapid thiol catalyzed peptide ligation occurs at pH 8.5–9, the most selective peptide ligation, that tolerates all proteinogenic side chains, occurs at pH 7. We have also identified the highly selective mechanism by which the intermediate peptidyl amidines undergo hydrolysis to α-peptides while demonstrating that the hydrolysis of amidines with nonproteinogenic structures, such as β- and γ-peptides, displays poor selectivity. Notably, this discovery enables the highly α-selective protecting-group-free ligation of lysine peptides at neutral pH while leaving the functional ε-amine side chain intact.

Peptide synthesis is one of the most important processes in chemistry and biology.^1^ Peptide biosynthesis is a highly evolved system,^2,3^ that could not have spontaneously appeared in its current form,^4^ but what nonenzymatic chemistry preceded it and how these reactions influenced the structure of biological peptides remains unknown. We recently reported that α-peptidyl nitriles **1** are activated for biomimetic peptide synthesis^5^ and that the in-built reactivity of **1**(6) can drive catalytic peptide ligation (CPL; Figure 1). CPL requires no activating agent to ligate **1** with amino acid derivatives (**2**)^5b^ and is a rare example of organocatalysis in water.^7^ The nitrile’s kinetic stability means ligation must be thiol catalyzed, and so catalyst-gated reactivity, which is an essential feature of biochemistry, is observed. As a mechanism for prebiotic peptide synthesis CPL has several appealing characteristics: it uses simple prebiotic reactants; is selective for α-amidonitriles, and therefore proteinogenic peptides; generates high peptide yields under conditions where peptides are very stable;^8^ and is orthogonal to (biological) phosphate activation,^9^ which would in principle enable independent catalytic modulation of both peptide and nucleic acid synthesis. Intriguingly, CPL produces amidines **3** when amino acids are the nucleophilic coupling partner (Figure 1; **2**, X = OH), whereas peptides **5′** are formed when α-amino amides or peptides are used (Figure 1; **2′**, X = NHR^4^).^5b^ With this in mind, we set out to explore the conditions under which CPL delivers the highest selectivity for α-peptide formation.^10^

**Figure 1:**
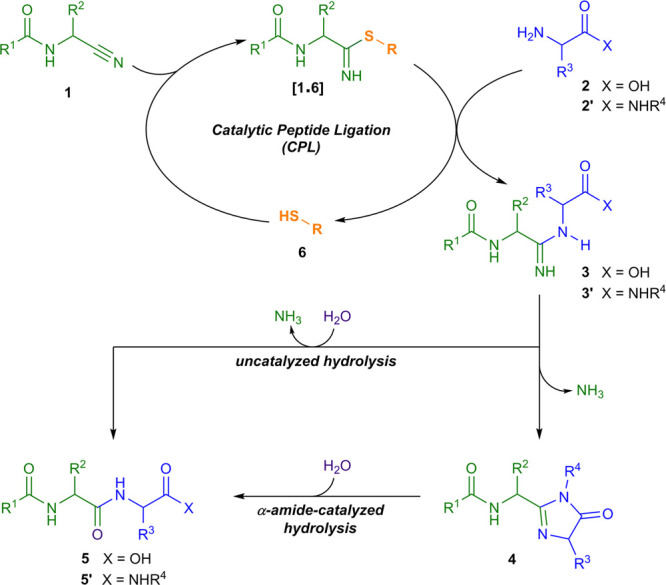
Catalytic peptide ligation (CPL) in water. Thiol-catalyzed coupling of peptide nitriles (**1**) with amines (**2**, X = OH or **2′**, X = NHR^4^). R = alkyl or aryl; R^1^ = peptide or alkyl; R^2^ and R^3^ = aminoacyl side chain; R^4^ = H or peptide; X = OH, NH_2_ or peptide. Compounds **2**–**5** and **2′**–**5′** are labeled with subscripts corresponding to the single letter amino acid code.

Our preliminary study of CPL focused on reactions at neutral pH, but we envisaged that pH would have a profound effect on CPL, as the nucleophile and catalyst could both deprotonate at higher pH. Pleasingly, we observed that ligation of alanine (H-Ala-OH, **2**_**A**_) to nitrile **1** catalyzed by Ac-Cys-OH (**6a**) is more rapid at pH 8.5 than at pH 7 (Table 1). In line with our prediction, we observed negligible reactivity at pH 5 but, surprisingly, slow and low yielding CPL at pH 10. This is likely due to suppressed thioimidate [**1·6**] protonation at pH 10. Accordingly, the optimal rate for **6a**-catalyzed CPL was observed at pH 8.5–9.0.

**Table 1:**
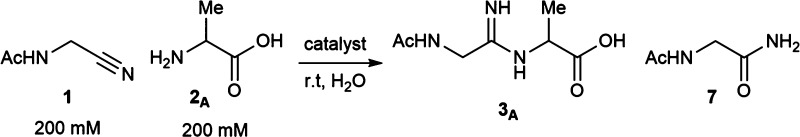

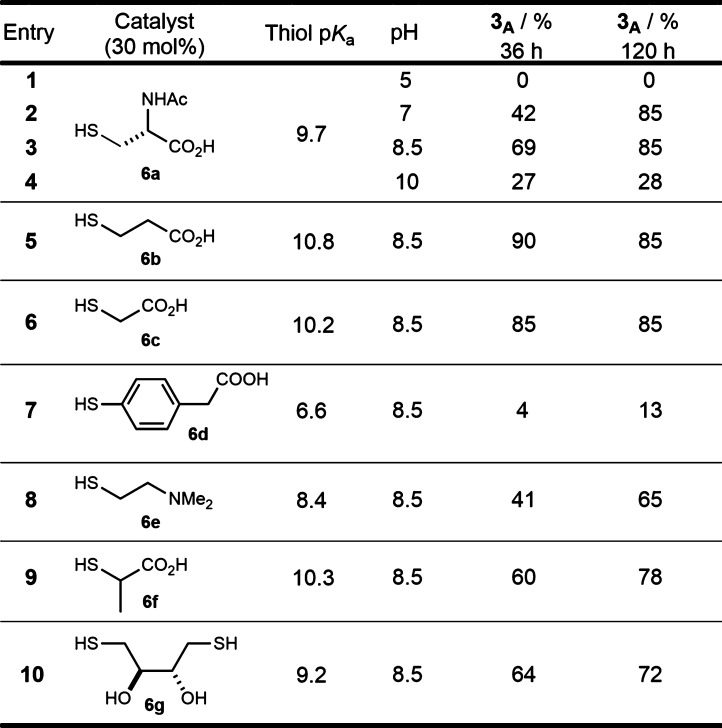
Effect of pH and Catalyst on Amidine **3** Formation^10^

3-Mercaptopropionic acid (**6b**) and thioglycolic acid (**6c**) promoted CPL faster than **6a**, giving 85–90% amidine (**3**_**A**_) after 36 h, at pH 8.5 and rt (Table 1). Low p*K*_a_ thiols (e.g., **6d** and **6e**) are sluggish, with **6d** only furnishing 4% amidine **3**_**A**_ after 36 h. Sterically hindered **6f** also retarded the rate of CPL. Limited hydration of **1** to Ac-Gly-NH_2_ (**7**) (∼5%) was observed with most catalysts (Figure S12), but **6g** yielded significant amide **7** (25%). We suspect **6g** undergoes S-to-O acyl transfer, followed by thiirane formation, leading to **7** (Figures S13 and S14).^11^ These results demonstrate high-p*K*_a_ primary thiols are best suited as CPL-catalysts. This stands in stark contrast to thioester ligations (e.g., native chemical ligation), which are accelerated by low p*K*_a_ thiols such as **6d**.^12^

High amidine **3** yields (82–95%) were observed in H-Gly-OH (**2**_**G**_), H-Ala-OH (**2**_**A**_), H-Leu-OH (**2**_**L**_), H-Ile-OH (**2**_**I**_), H-Phe-OH (**2**_**F**_), H-Met-OH (**2**_**M**_), H-Val-OH (**2**_**V**_), H-Arg-OH (**2**_**R**_), H-Glu-OH (**2**_**E**_), H-Asp-OH (**2**_**D**_), H-Gln-OH (**2**_**Q**_), H-Trp-OH (**2**_**W**_), H-Pro-OH (**2**_**P**_), and H-His-OH (**2**_**H**_) couplings (Figures S16–S51). H-Cys-OH (**2**_**C**_) coupling does not require catalysis, due to the thiol side chain.^5b^ H-Lys-OH (**2**_**K**_, 86%) exhibited moderate α-selectivity (2:1, α/ε) with 60% α-amidine formation (Figure S37), while β-hydroxy amino acids (i.e., H-Ser-OH, **2**_**S**_ and H-Thr-OH, **2**_**T**_) yielded peptides rather than amidines. We have postulated that this is due to the formation and hydrolysis of oxazoline **8** (Figure 2A). Here, at room temperature, we observed **8** as the major product (**8**_**S**_ (55%) and **8**_**T**_ (67%); Figures S45 and S49). Oxazoline **8** formed rapidly from nitrile **1** and **2**_**S**_ or **2**_**T**_, but its hydrolysis is slow at 25 °C. However, heating **8**_**S**_ and **8**_**T**_ at 60 °C for 12 h resulted in high yielding conversion to **5**_**S**_ and **5**_**T**_.

**Figure 2:**
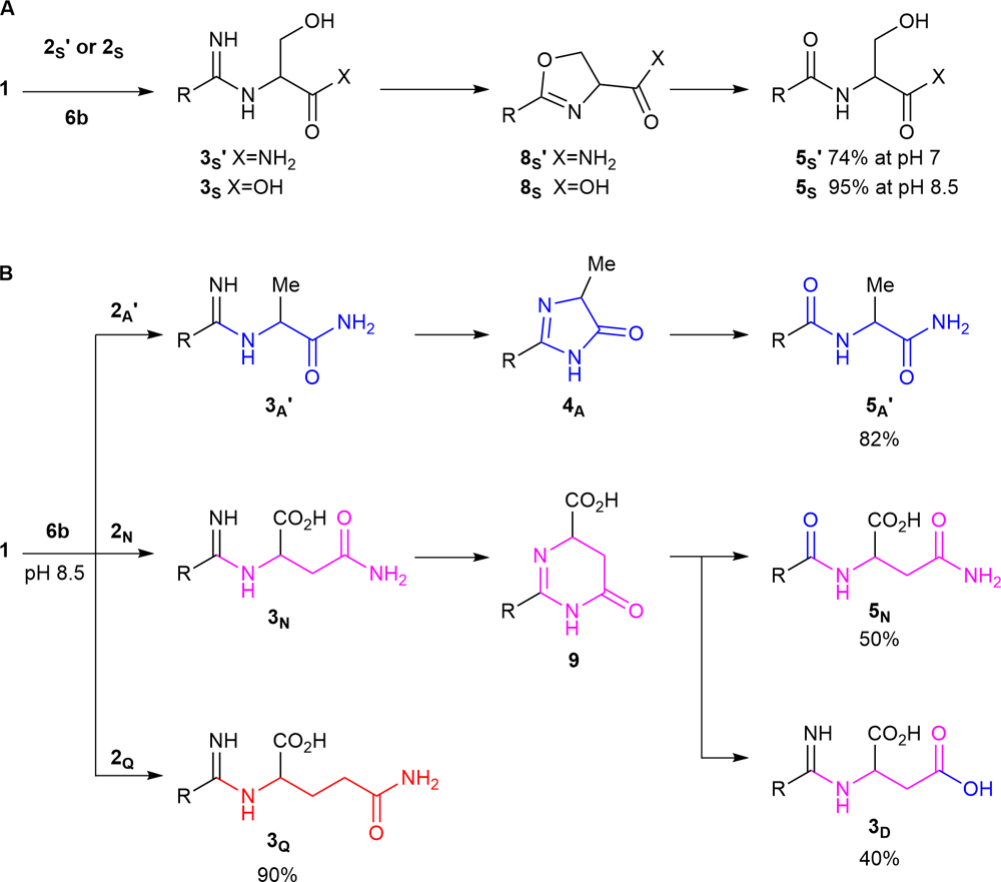
Intramolecular catalysis of amidine hydrolysis. (A) Coupling of nitrile **1** (200 mM) with serinamide (**2**_**S**_**′**, 2 equiv) yields peptide **5**_**S**_**′** at pH 7, via oxazoline **8**, whereas serine (**2**_**S,**_ 1 equiv) yields peptide **5**_**S**_ at pH 7 (ref (5b)) or pH 8.5. (B) Coupling of **1** (200 mM) with alaninamide (**2**_**A**_**′**, 2 equiv), asparagine (**2**_**N**_, 1 equiv), glutamine (**2**_**Q**_, 1 equiv) demonstrates the effect of α-, β-, and γ-amides amidine hydrolysis during CPL. R = CH_2_NHCOCH_3_.

Like **2**_**S**_ and **2**_**T**_, α-amino amide (**2′**) nucleophiles directly form peptides **5′** in CPL. This selective peptide formation, promoted by the α-peptide backbone, warranted further investigation. Amidine **3′** can in principle hydrolyze through substitution of ammonia or amino amide (**2′**), and their similar p*K*_aH_ values suggested that direct hydrolysis should yield peptide **5′** and amide **7** in comparable yields. However, peptide **5′** forms selectively, implicating intramolecular catalysis. Upon coupling H-Ala-NH_2_ (**2**_**A**_**′**) and nitrile **1**, we observed slow hydrolysis of amidine **3**_**A**_**′** to **5**_**A**_**′**. At room temperature, we also observed an imidazolone intermediate (**4**_**A**_) (Figure 2B, Figures S55 and S118–S120). This cyclization explains the selective formation of peptide **5′**, with intramolecular substitution promoting loss of ammonia.

We speculated this selectivity would be uniquely effective for (biogenic) α-peptides. To test this, we used alaninamide (H-Ala-NH_2_, **2**_**A**_**′**), asparagine (H-Asn-OH, **2**_**N**_), and glutamine (H-Gln-OH, **2**_**Q**_) as homologous nucleophiles (with α-, β-, and γ-amides) to investigate amide-catalyzed amidine hydrolysis (Figure 2B). α-Amides cyclize to 5-membered imidazolone **4** and hydrolyze selectively to α-peptides **5′**. Although β-amides (e.g., **2**_**N**_) also cyclize, they yield 6-membered dihydropyrimidone **9** that hydrolyze with poor selectivity yielding a mixture of peptide **5**_**N**_ (50%) and amidine **3**_**D**_ (40%). Thus, unlike α-amides, β-amides undergo significant β-peptide hydrolysis (Figure S22). Extending the series further inhibited cyclization completely, and γ-amide **2**_**Q**_ only formed amidine **3**_**Q**_ (Figure S28). These results demonstrate the disposition of α-amino amides (i.e., proteinogenic peptides) to catalyze selective amidine-to-peptide hydrolysis, while nonproteinogenic β- or γ-amino amides are either poor catalysts or catalytically inactive.

Cyclization of **3**_**A**_**′** to **4**_**A**_ and hydrolysis of **4**_**A**_ to peptide **5**_**A**_**′** exhibits a strong pH dependence, and both are rapid at pH 10 and sluggish at pH 7 (Figures 3, S124, and S126). However, at pH 7, both are accelerated by phosphate buffer (100 mM, Figure S123) but not by **6b** (Figure 3C). This suggests that both hydrolysis and cyclization are general acid–base catalyzed. Moreover, hydrolysis can be catalyzed by a combination of intra- and intermolecular catalysis, and orthogonal catalysts (i.e., thiols and phosphates) can independently catalyze amidine ligation and hydrolysis.

**Figure 3:**
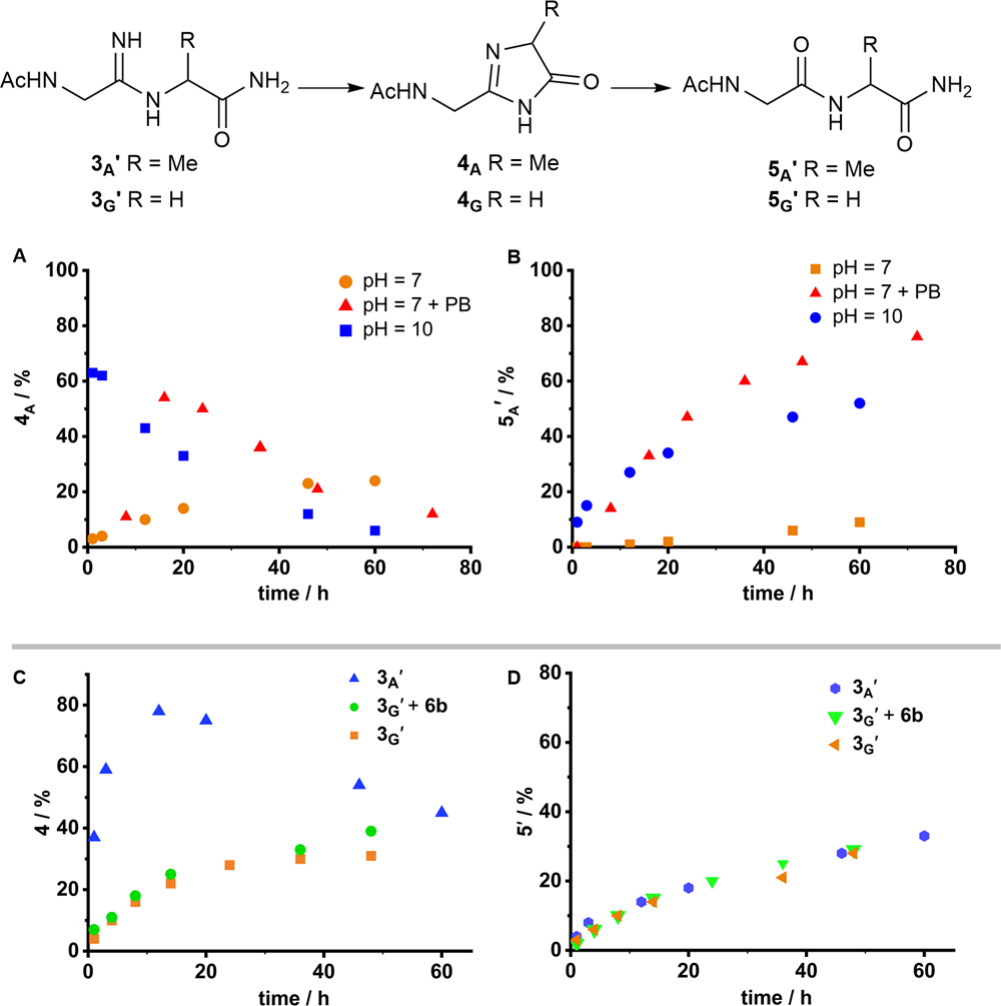
Effect of pH, buffer and catalyst on imidazolone formation and hydrolysis. Time courses to show the (A) formation of imidazolone **4**_**A**_ from amidine **3**_**A**_**′** (25 mM) at rt and pH 10 or pH 7, with and without phosphate buffer (PB, 100 mM); (B) formation of peptide **5**_**A**_**′** from **3**_**A**_**′** (25 mM) at r.t and pH 10 or pH 7, with and without PB (100 mM); (C) formation of **4**_**A**_ from **3**_**A**_**′** (25 mM) and **4**_**G**_ from **3**_**G**_**′** (25 mM) at rt and pH 9, with and without **6b** (100 mM); (D) formation of peptides **5**_**A**_**′** from **3**_**A**_**′** (25 mM) and **5**_**G**_**′** from **3**_**G**_**′** (25 mM) at rt and pH 9, with and without **6b** (100 mM).

To investigate the effect of side chains on amidine hydrolysis, proteinogenic amino amides (**2′**) were studied in CPL with **1**. Peptides were formed in 70–90% yield with H-Ala-NH_2_ (**2**_**A**_**′**), H-Val-NH_2_ (**2**_**V**_**′**), H-Leu-NH_2_ (**2**_**L**_**′**), H-Phe-NH_2_ (**2**_**F**_**′**), H-Arg-NH_2_ (**2**_**R**_**′**), H-His-NH_2_ (**2**_**H**_**′**), H-Pro-NH_2_ (**2**_**P**_**′**), H-Tyr-NH_2_ (**2**_**Y**_**′**), H-Trp-NH_2_ (**2**_**W**_**′**), H-Glu-NH_2_ (**2**_**E**_**′**) and H-Asp-NH_2_ (**2**_**D**_**′**) (Figure S54–S101). H-Ile-NH_2_ (**2**_**I**_**′**) forms a mixture of diastereomers (11:9 ratio) in high yield (86%). Since H-Ile-OH (**2**_**I**_) forms only one amidine diastereomer, this implies racemization occurs during the cyclization-hydrolysis process. Slightly lower yields were observed with H-Gly-NH_2_ (**2**_**G**_**′**, 50%, Figure S70), H-Gln-NH_2_ (**2**_**Q**_**′**, 66%, Figure S68) and H-Met-NH_2_ (2_**M**_**′**, 63%, Figure S81). All NMR data were consistent with formation of intermediates **4**. The course of the reaction was different for the amino amides (H-Ser-NH_2_**2**_**S**_**′**, H-Thr-NH_2_**2**_**T**_**′** and H-Asn-NH_2_**2**_**N**_**′**) with side chains that promote amidine hydrolysis. Asparaginamide (**2**_**N**_**′**) formed a mixture of Asn and Asp peptides in 50% yield after 10 days, alongside **7** (45%; Figure S62), but Asn peptides undergo facile hydrolysis,^13^ so this low yield is likely intrinsic to this side chain and no attempt was made to optimize H-Asn-NH_2_ coupling.

Surprisingly, coupling with **2**_**S**_**′** led to decomposition at pH 8.5, forming no detectable peptide (Figure S88). Oxazoline **8**_**S**_**′** (Figure 2) was, however, observed at pH 7, and heating **8**_**S**_**′** at 60 °C led to peptide **5**_**S**_**′** (74%) after 36 h (Figure S89). Similarly, **2**_**T**_**′** was converted to **5**_**T**_′ (85%) after 36 h at 60 °C and pH 7 (Figure S97) as a single diastereomer. On the other hand, nonproteinogenic *O*-methyl serinamide **2**_**MeS**_**′** decomposed rather than forming peptide even at pH 7 (Figure S142). These results demonstrate that, at pH 7, oxazoline formation overcomes the incompatibility of β-hydroxyl residues with CPL at elevated pH. Likewise, CPL with peptide nucleophiles (e.g., H-Ala-Gly-Ala-OH **2**_**AGA**_; Figures S102–S106) at pH 8.5 and 60 °C only furnished tetrapeptide Ac-Gly-Ala-Gly-Ala-OH (**5**_**AGA**_) in 50% yield, alongside substantial **7** (30%, Figure S104), whereas, at pH 7 and 60 °C, CPL was much more selective and ligation was observed to yield **5**_**AGA**_ (81%) (Figure S102). Thus, though faster at pH 8.5, CPL is only universally compatible and high yielding with proteinogenic peptides at neutral pH.

We next turned our attention to (uncatalyzed) hydrolysis of amidine **3** (X = OH). Whereas high selectivity of amidine-to-peptide hydrolysis was observed for α-amide **3**_**A**_**′**, α-acid **3**_**A**_ furnished a mixture of **7** and **5**_**A**_. At 80 °C, moderate selectivity for hydrolysis of **3**_**A**_ to **7** (2:1 **7**/**5**_**A**_) was observed at pH 7–9 (Table 2, entries 2–4). We postulated that this selectivity arose due to the difference in amine p*K*_aH_ (**2**_**A**_ = 9.7; ammonia = 9.2), with the higher p*K*_aH_ amine selectively substituted. This suggested a new mechanism to effect selective α-ligation of lysine peptides in water.^5a,14^ The high p*K*_aH_ of the ε-amine (10.8) compared to ammonia (9.2) suggested that hydrolysis of ε-lysyl amidines would selectively yield the free ε-amine. Furthermore, because α-lysyl peptide amidines (e.g., **3**_**K**_**′**) undergo effective (intramolecular amide-catalyzed) hydrolysis to α-peptides, we envisaged α-peptide ligation and ε-hydrolysis would operate together and reinforce selectivity for proteinogenic peptide ligation.

**Table 2:**
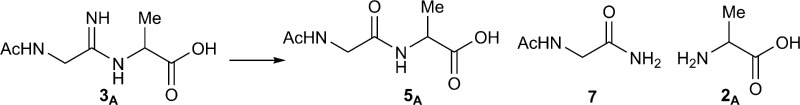
Hydrolysis of Amidine **3**_**A**_^a^

entry	pH	temp, °C	buffer (500 mM)	time	3_A_, %	5_A_, %	2_A_, %
1	7	20		30 days	100	0	0
2	7	80		18 h	34	15	36
3	7	80	PB	18 h	11	31	52
4	9	80	BB	18 h	0	38	61

a PB = phosphate buffer, BB = borate buffer.

Upon coupling of H-Lys-NH_2_ (**2**_**K**_**′**) and **1**, at pH 9, we observed that α-peptide **5**_**K**_**′** (20%) was a minor product, formed alongside ε-amide **11** (15%) and substantial amounts of *N*-acetylglycinamide **7** (59%) after 24 h at 80 °C (Table 3, entry 2 and Figure S135). This demonstrates that α-ligation is disfavored at pH 9 and the predominant product is hydration (i.e., **7**). However, at neutral pH, the selectivity for α-ligation was dramatically increased. At pH 7 peptide **5**_**K**_**′** was the major product after 6 days, yielding **5**_**K**_**′** (58–65%) and only 7% ε-amide **11** (α:ε 9:1; Figure S137). Neutral pH ligation of lysyl peptides was similarly effective; H-Lys-Gly-OH (**2**_**KG**_) and H-Lys-Lys-OH (**2**_**KK**_) were ligated selectively to afford peptide Ac-Gly-Lys-Gly-OH **5**_**KG**_ (α:ε 7:1; Figures S138 and 139) and Ac-Gly-Lys-Lys-OH **5**_**KK**_ (α:ε 5:1; Figures S140–141).

**Table 3:**
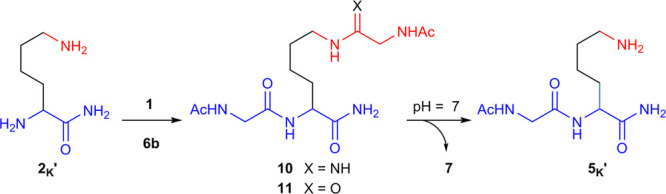
Selectivity for Ligation at α- or ε-Amine of Lysyl Peptides^a^

entry	pH	ε-amidine **10**, %	total amide (α + ε), %	ratio α:ε acylation
1	9^b^	0	35	1.3
2	7^b^	19	67	3.5
3	7^c^	0	72	9.3

a Selective α-lysyl peptide (blue) over ε-lysyl amine (red) coupling of 200 mM **2**_**K**_′ with 200 mM **1**, 30 mol % **6b** at 80 °C.

b Yields after 1 day.

c Yields after 6 days.

In conclusion, we have demonstrated that proteinogenic substrates undergo selective CPL to furnish racemic α-peptides catalyzed by the adjacent α-amide/peptide at neutral pH. β-Hydroxyl α-amides retain chirality via hydroxyl catalysis, but O-methylation inhibits peptide formation in these substrates even at neutral pH. The impact and value of peptide racemization and stereoretention, within a (likely racemic) prebiotic environment, during self-catalyzed peptidyl-amidine hydrolysis remains an open question.^5b^ By studying the (uncatalyzed) hydrolysis of amidines we have discovered a preference for the substitution of the higher p*K*_aH_ amine. This uncatalyzed reaction operates in tandem with α-amide-catalyzed hydrolysis to enhance the selectivity for lysine α-peptide synthesis at neutral pH in water while retaining the lysyl (functional) ε-amine group.

## Supporting Information Available

Experimental procedures and spectroscopic data (PDF)
