# Supplementary material for: Prebiotic Catalytic Peptide Ligation Yields Proteinogenic Peptides by Intramolecular Amide Catalyzed Hydrolysis Facilitating Regioselective Lysine Ligation in Neutral Water
Source: J Am Chem Soc. 2022 May 31;144(23):10151–5. doi: 10.1021/jacs.2c03486 (PMC9204760; doi:10.1021/jacs.2c03486)
Supplement: Supplementary file 1 — ja2c03486_si_001.pdf [file ja2c03486_si_001.pdf]

Prebiotic catalytic peptide ligation yields proteinogenic peptides by intramolecular amide catalyzed hydrolysis facilitating regioselective lysine ligation in neutral water.

Jyoti Singh‡[1], Daniel Whitaker‡[1], Benjamin Thoma[1], Saidul Islam[1,2], Callum S. Foden[1], Abil E. Aliev[1], Tom D. Sheppard[1], and Matthew W. Powner*[1]

[1]Department of Chemistry, University College London, 20 Gordon Street, London, WC1H 0AJ, UK

[2]Department of Chemistry, King's College London, 7 Trinity Street, London, SE1 1DB, UK

Contents

General 4

Catalyst screening for the coupling of N-acetylglycine nitrile **1** with alanine $\mathbf{2_A}$ 5

Coupling of 1 with L-alanine $\mathbf{2_A}$ catalyzed by **6a** at room temperature 6

Coupling of 1 with L-alanine $\mathbf{2_A}$ catalyzed by **6b** at room temperature 10

Coupling of 1 with L-alanine $\mathbf{2_A}$ catalyzed by **6c** at room temperature 12

Coupling of 1 with L-alanine $\mathbf{2_A}$ catalyzed by **6d** at room temperature 14

Coupling of 1 with L-alanine $\mathbf{2_A}$ catalyzed by **6e** at room temperature 15

Coupling of 1 with L-alanine $\mathbf{2_A}$ catalyzed by **6f** at room temperature 16

Coupling of 1 with L-alanine $\mathbf{2_A}$ catalyzed by **6g** at room temperature 18

Coupling of 1 with L-alanine $\mathbf{2_A}$ catalyzed by **6h** at room temperature 22

Coupling of N-acetylglycine nitrile **1** with α-amino acids **2** at pH 8.5 and room temperature 25

Coupling of **1** with L-alanine $\mathbf{2_A}$ at pH 8.5 and room temperature 26

Coupling of **1** with L-aspartic acid $\mathbf{2_D}$ at pH 8.5 and room temperature 28

Coupling of **1** with L-arginine $\mathbf{2_R}$ at pH 8.5 and room temperature 30

Coupling of **1** with L-asparagine $\mathbf{2_N}$ at pH 8.5 and room temperature 32

Coupling of **1** with L-glutamic acid $\mathbf{2_E}$ at pH 8.5 and room temperature 34

Coupling of **1** with L-glutamine $\mathbf{2_Q}$ at pH 8.5 and room temperature 36

Coupling of **1** with glycine $\mathbf{2_G}$ at pH 8.5 and room temperature 38

Coupling of **1** with L-histidine $\mathbf{2_H}$ at pH 8.5 and room temperature 40

Coupling of **1** with L-isoleucine $\mathbf{2_I}$ at pH 8.5 and room temperature 42

Coupling of **1** with L-leucine $\mathbf{2_L}$ at pH 8.5 and room temperature 44

Coupling of **1** with L-lysine $\mathbf{2_K}$ at pH 8.5 and room temperature 46

Coupling of **1** with DL-methionine $\mathbf{2_M}$ at pH 8.5 and room temperature 48

Coupling of **1** with L-phenylalanine $\mathbf{2_F}$ at pH 8.5 and room temperature 50

Coupling of **1** with L-proline $\mathbf{2_P}$ at pH 8.5 and room temperature 52

Coupling of **1** with L-serine $\mathbf{2_S}$ at pH 8.5 and room temperature 54

Coupling of **1** with L-tryptophan $\mathbf{2_W}$ at pH 8.5 and room temperature 56

Coupling of **1** with L-threonine $\mathbf{2_T}$ at pH 8.5 and room temperature 58

Coupling of **1** with L-valine $\mathbf{2_V}$ at pH 8.5 and room temperature 60

Competitive coupling of **1** with glycine $\mathbf{2_G}$ and ammonium chloride 61

Coupling of N-acetylglycine nitrile **1** with α-amino amides **2’** at pH 8.5 and room temperature 63

Coupling of N-acetylglycine nitrile **1** with L-alaninamide $\mathbf{2_A'}$ at pH 8.5 and room temperature .. 64

Coupling of N-acetylglycine nitrile **1** with L-aspartic acid amide $\mathbf{2_D'}$ at pH 8.5 and room temperature 66

Coupling of N-acetylglycine nitrile **1** with L-arginine amide **2_R'** at pH 8.5 and room temperature 69
Coupling of N-acetylglycine nitrile **1** with L-asparagine amide **2_N'** at pH 8.5 and room temperature 72
Coupling of N-acetylglycine nitrile **1** with L-glutamic acid amide **2_E'** at pH 8.5 and room temperature 75
Coupling of N-acetylglycine nitrile **1** with L-glutamine amide **2_Q'** at pH 8.5 and room temperature 78
Coupling of N-acetylglycine nitrile **1** with glycinamide **2_G'** at pH 8.5 and room temperature 80
Coupling of N-acetylglycine nitrile **1** with L-histidine amide **2_H'** at pH 8.5 and room temperature 83
Coupling of N-acetylglycine nitrile **1** with L-isoleucinamide **2_I'** at pH 8.5 and room temperature 86
Coupling of N-acetylglycine nitrile **1** with L-leucinamide **2_L'** at pH 8.5 and room temperature 88
Coupling of N-acetylglycine nitrile **1** with DL-methionine amide **2_M'**at pH 8.5 and room temperature 91
Coupling of N-acetylglycine nitrile **1** with L-phenylalaninamide **2_F'** at pH 8.5 and room temperature 94
Coupling of N-acetylglycine nitrile **1** with L-prolinamide **2_P'** at pH 8.5 and room temperature 96
Coupling of N-acetylglycine nitrile **1** with L-serinamide **2_S'** at pH 8.5 and room temperature 98
Coupling of N-acetylglycine nitrile **1** with L-tryptophan amide **2_W'** at pH 8.5 and room temperature 101
Coupling of N-acetylglycine nitrile **1** with L-tyrosinamide **2_Y'** at pH 8.5 and room temperature 104
Coupling of N-acetylglycine nitrile **1** with L-threoninamide **2_T'** at pH 8.5 and room temperature 106
Coupling of N-acetylglycine nitrile **1** with L-valinamide **2_V'** at pH 8.5 and room temperature 109
Coupling of **1** with peptide fragments catalyzed by **6b** 112
Coupling of **1** with L-alanylglycyl-L-alanine **2_{AGA}** *112*
Coupling of **1** with L-alanyl-glycine **2_{AG}** *117*
Preparative synthesis of amidines **3'** 119
Isolation of **3_A** 128
Hydrolysis of authentic amidines **3'** 130
Hydrolysis of **3_A'** at pH 7 in phosphate buffer (100 mM) 130
Hydrolysis of **3_A'** at pH 7 131
Hydrolysis of **3_A'** at pH 9 132
Hydrolysis of **3_A'** at pH 10 133
Hydrolysis of **3_G'** at pH 9 134

Hydrolysis of **3_G'** at pH 9 in the presence of 3-mercaptopropionic acid **6b** ... 135
Hydrolysis of **3_G'** at pH 9 in the presence of L-alanine (**2_A**) ... 136
Hydrolysis of **3_G'** at pH 9 in the presence of L-alaninamide (**2_A'**) ... 137
Hydrolysis of **3_A** ... 138
Hydrolysis of **3_A** at pH 7 ... 138
Hydrolysis of **3_A** at pH 9 ... 139
α-selective ligation of lysine-peptides ... 140
Coupling of N-acetylglycine nitrile **1** with L-lysinamide **2_K'** at pH 8.5 and room temperature ... 140
Coupling of N-acetylglycine nitrile **1** with L-lysinamide **2_K'** at pH 9.0, 80 °C ... 142
Coupling of N-acetylglycine nitrile **1** with L-lysinamide **2_K'** at pH 7.0, 80°C ... 144
Coupling of N-acetylglycine nitrile **1** with L-lysylglycine **2_{KG}** at pH 7.0, 80 °C ... 145
Coupling of N-acetylglycine nitrile **1** with L-lysyl-L-lysine **2_{KK}** at pH 7.0, 80 °C ... 147
Coupling of N-acetylglycine nitrile **1** with O-methyl serinamide **2_{MeS}'** catalyzed by **6b** ... 149

General

^{1}H, ^{13}C and ^{19}F NMR spectra were recorded on Bruker NMR spectrometers AVANCE Neo 700, AVANCE III 600 and AVANCE III 400, equipped with a Bruker room temperature 5 mm multinuclear gradient probe (700 MHz), 5 mm DCH cryoprobe (600 MHz) and a gradient probe (400 MHz). Unless otherwise stated ^{13}C NMR spectra were acquired with broadband ^{1}H-decoupling. All chemical shifts (δ) are reported in parts per million (ppm) relative to residual solvent peaks, and ^{1}H and ^{13}C chemical shifts relative to TMS were calibrated using the residual solvent peak (residual solvent peaks: (δH/ppm) D_2O – 4.75; $CDCl_3$ – 7.26; CD_3OD – 3.31). Nuclear assignments were made using 2D NMR homo- and heteronuclear correlation spectroscopy (^{1}H–^{1}H COSY; ^{1}H–^{13}C HSQC; ^{1}H–^{13}C HMBC). Where noted, solvent suppression pulse sequence with presaturation and spoil gradients was used to obtain ^{1}H NMR spectra (noesygppr1d, Bruker) and ^{1}H–^{13}C HMBC NMR spectra (hmbcgplpndprqf, Bruker). Coupling constants are reported in Hertz (Hz). Spin multiplicities are indicated by symbols: s (singlet); d (doublet); t (triplet); q (quartet); qn (quintet); spt (septet); oct octet), m (multiplet); obs. (obscured/coincidental signals), or a combination of these. Spectra were recorded at 298 K. Reagents and solvents were obtained and used without further purification, unless specified, from the following commercial sources: Alfa Aesar, Acros Organics, Apollo Scientific, BDH, Sigma Aldrich, Fluorochem, MerckMillipore, Fisher Scientific, VWR International, Carbosynth, Manchester Organics, Lancaster, Molekula, Honeywell, TCI and Santa Cruz Biotechnology. Deionized water was obtained from an Elga Option 3 purification system. Infrared spectra (IR) were recorded on a Shimadzu IR Tracer 100 FT-IR spectrometer as a solid or neat oil/liquid. Absorption maxima are reported in wavenumber (cm^{-1}). Mass spectra and accurate mass measurements were recorded on a Waters LCT Premier QTOF connected to a Waters Autosampler Manager 2777C, Thermo Finnigan MAT900, and an Agilent LC connected to an Agilent 6510 QTOF mass spectrometer at the Department of Chemistry, University College London. Solution pH values were measured using a Mettler Toledo Seven Compact pH meter with a Mettler Toledo InLab semi-micro pH probe, or a Corning 430 pH meter with a Fisherbrand FB68801 semi-micro pH probe. The readings for D_2O solutions are reported as pD, and corrected as measured pH + 0.4.[1] The readings for H_2O and H_2O/D_2O (9:1) solutions are reported uncorrected**.**

Catalyst screening for the coupling of N-acetylglycine nitrile **1** with alanine **2_A**

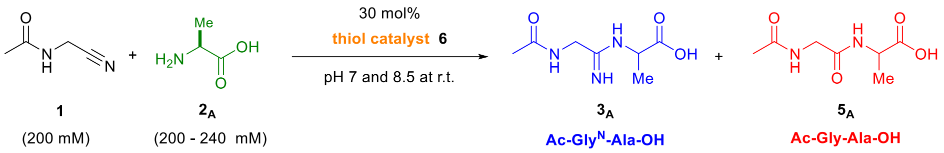

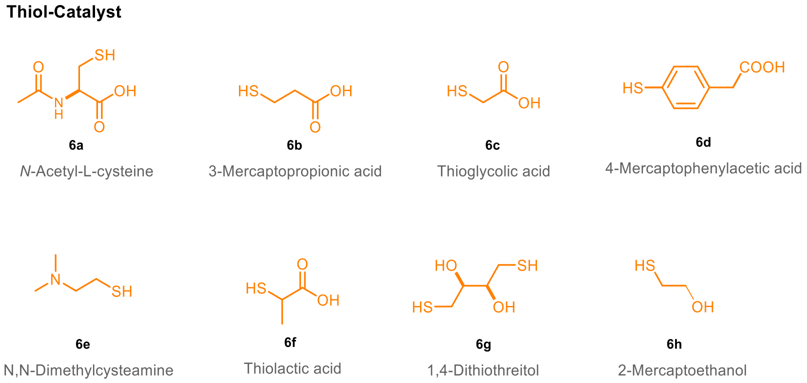

Figure S1: Formation of Ac-Gly^N-Ala-OH **3_A** by thiol-catalyzed (Catalyst–SH; 30 mol%) coupling of N-acetyl glycine nitrile, **1** (200 mM) and *L*-Ala, **2_A** (200 - 240 mM).

Coupling of 1 with L-alanine ***2***$_{\textbf{A}}$ *catalyzed by* ***6a*** *at room temperature*

N-Acetylglycine nitrile (**1**, 200 mM), L-alanine (**2**$_{\textbf{A}}$, 200 mM), and L-N-acetyl cysteine (**6a**, 30 mol%, 60 mM) were dissolved in H_2O/D_2O (9:1, 0.8 mL) along with methyl sulfonyl methane (MSM; 14 mM) as internal standard. The pH of the solution was adjusted to the desired value with 4 M HCl/NaOH, the solution volume was increased to 1 mL and NMR spectra were obtained periodically.

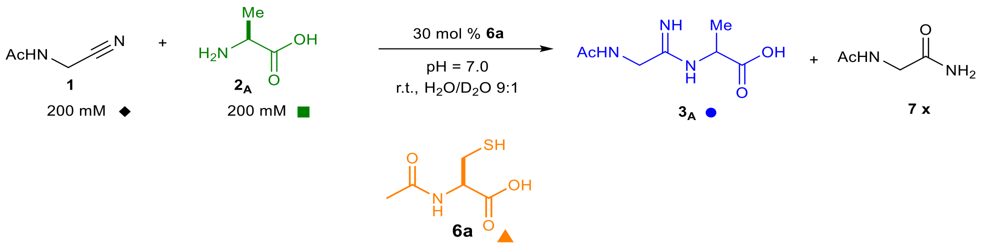

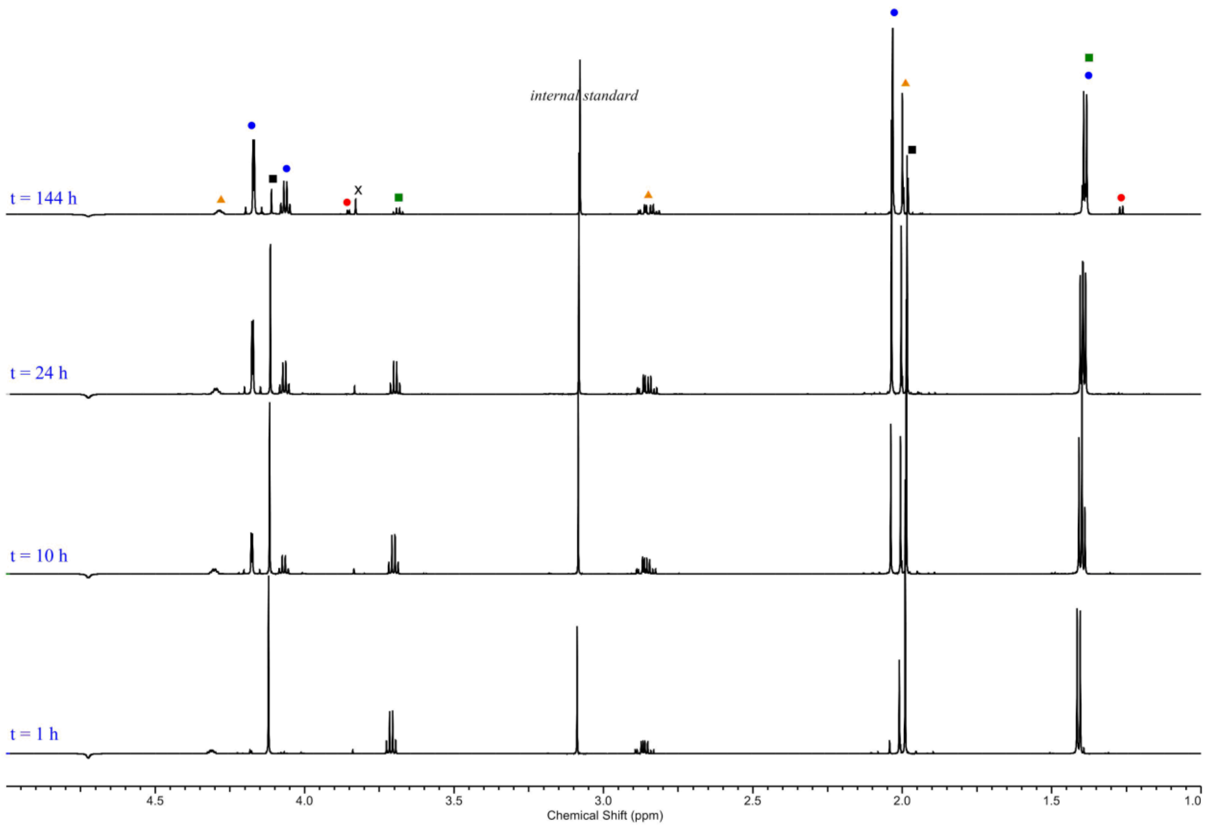

Figure S2: ^1H NMR (700 MHz, H_2O/D_2O 9:1, noesygppr1d, 1.0-5.0 ppm) spectrum to show the reaction of N-acetyl glycine nitrile (**1**, 200 mM) with L-alanine (**2**$_{\textbf{A}}$, 200 mM) and L-N-acetyl cysteine (**6a**, 60 mM), with MSM (14 mM) as an internal standard, at pH 7, room temperature.

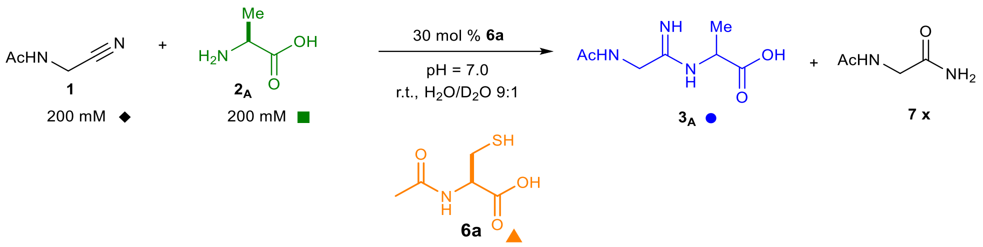

Entry	time / h	**1** / %	**2**$_A$ / %	**3**$_A$ / %	**7** / %
1	1	91	92	1	2
2	7	88	89	6	3
3	12	83	84	11	3
4	25	69	72	25	4
5	37	58	60	37	4
6	49	47	50	46	4
7	61	40	42	54	4
8	73	34	36	61	5
9	97	25	27	69	5
10	121	19	22	74	5
11	145	16	18	77	6

Table S1. Yields over time for the reaction between N-acetyl glycine nitrile (**1**, 200 mM) with L-alanine (**2**$_A$, 200 mM) and L-N-acetyl cysteine (**6a**, 60 mM) at pH 7.0, room temperature.

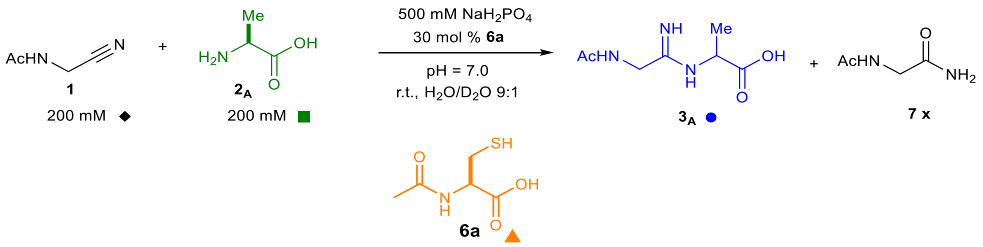

Entry	time / h	**1** / %	**2**$_A$ / %	**3**$_A$ / %	**7** / %
1	1	96	107	0	3
2	7	90	104	6	6
3	12	83	100	10	9
4	25	69	93	18	16
5	37	57	86	23	21
6	49	48	81	28	26
7	61	41	78	32	29
8	73	34	74	35	33
9	97	25	70	39	38
10	121	18	66	43	42
11	145	13	64	45	45

Table S2. Yields over time for the reaction between N-acetyl glycine nitrile (**1**, 200 mM) with L-alanine (**2**$_A$, 200 mM) and L-N-acetyl cysteine (**6a**, 60 mM) in phosphate buffer (500 mM) at pH 7.0, room temperature.

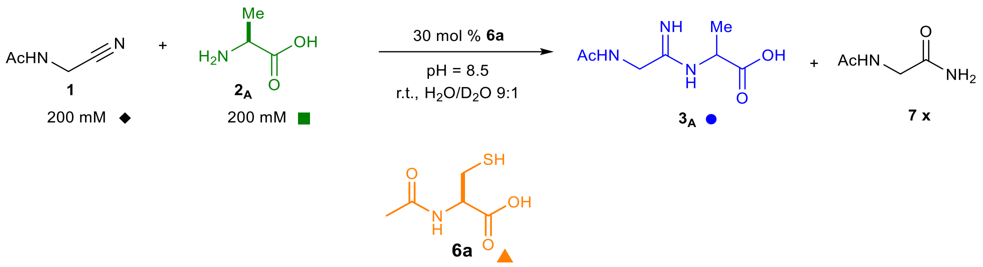

Entry	time / h	**1** / %	**2**$_A$ / %	**3**$_A$ / %	**7** / %
1	1	84	96	5	2
2	7	67	77	22	2
3	12	56	65	35	3
4	25	39	45	53	2
5	37	30	36	63	3
6	49	24	30	69	3
7	61	20	25	71	4
8	73	17	22	75	4
9	97	13	19	77	5
10	121	11	17	79	6
11	145	9	16	81	7

Table S3. Yields over time for the reaction between N-acetyl glycine nitrile (**1**, 200 mM) with L-alanine (**2**$_A$, 200 mM) and L-N-acetyl cysteine (**6a**, 60 mM) at pH 8.5, room temperature.

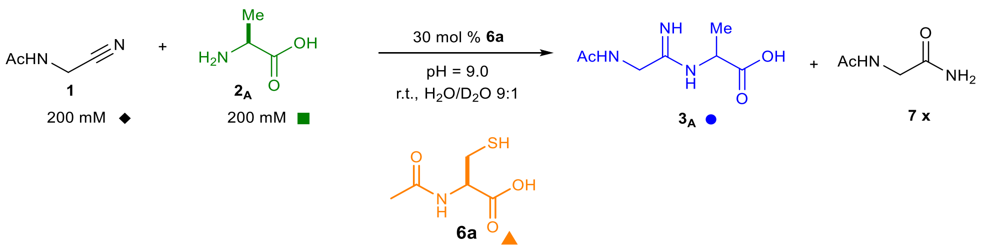

Entry	time / h	**1** / %	**2**$_A$ / %	**3**$_A$ / %	**7** / %
1	1	84	96	5	2
2	7	67	77	22	2
3	12	56	65	35	3
4	25	39	45	53	2
5	37	30	36	63	3
6	49	24	30	69	3
7	61	20	25	71	4
8	73	17	22	75	4
9	97	13	19	77	5
10	121	11	17	79	6
11	145	9	16	81	7

Table S4. Yields over time for the reaction between N-acetyl glycine nitrile (**1**, 200 mM) with L-alanine (**2**$_A$, 200 mM) and L-N-acetyl cysteine (**6a**, 60 mM) at pH 9.0, room temperature.

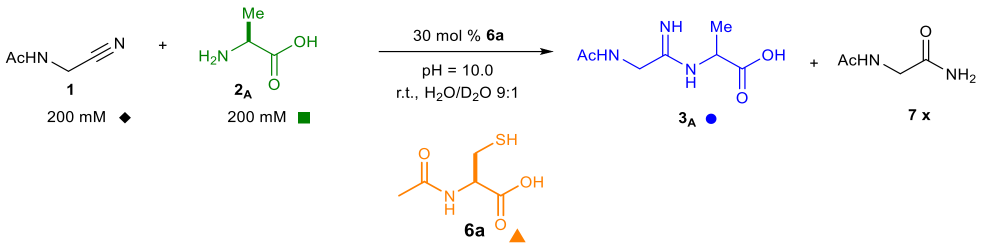

Entry	time / h	**1** / %	**2**$_A$ / %	**3**$_A$ / %	**7** / %
1	2	84	95	2	2
2	7	76	86	9	2
3	12	71	82	14	3
4	25	61	73	21	5
5	37	55	69	26	7
6	49	49	66	28	10
7	61	43	61	28	12
8	73	39	60	29	14
9	97	33	56	27	20
10	121	28	55	27	24

Table S5. Yields over time for the reaction between N-acetyl glycine nitrile (**1**, 200 mM) with L-alanine (**2**$_A$, 200 mM) and L-N-acetyl cysteine (**6a**, 60 mM) at pH 10.0, room temperature.

*Coupling of 1 with L-alanine 2_A catalyzed by **6b** at room temperature*

N-Acetylglycine nitrile (**1**, 200 mM), L-alanine (**2_A**, 220 mM), and 3-mercaptopropionic acid (**6b**, 30 mol%, 60 mM) and MSM (100 mM) as internal standard were dissolved in H_2O/D_2O (9:1, 0.8 mL). The pH of the solution was adjusted to the desired value with 4 M HCl/NaOH, the solution volume was increased to 1 mL and NMR spectra were obtained periodically.

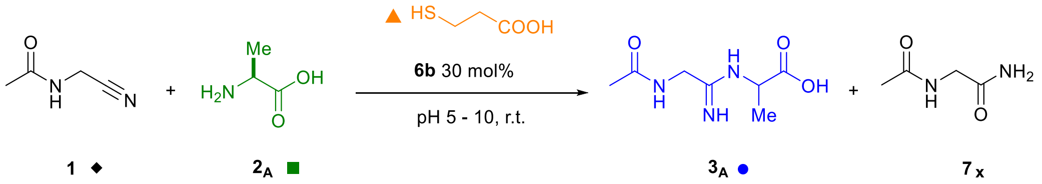

Entry	Time /h	pH = 5<br>3_A /%	pH = 7<br>3_A / %	pH = 7,<br>1 M Pi,<br>3_A / %	pH = 8.5<br>1 M borate,<br>3_A / %	pH = 9<br>1 M borate,<br>3_A / %	pH = 10<br>3_A / %
1	1	0	0	0	25	32	17
2	6	0	4	3	57	61	32
3	12	0	7	6	73	78	38
4	24	0	14	11	87	88	43
5	36	0	21	16	91	91	41
6	48	0	29	20	93	90	38
7	60	0	38	24	94	90	34
8	72	0	44	27	94	89	30
9	96	0	59	33	93	87	25
10	108	0	64	35	92	-	20
11	120	0	71	36	92	-	-
12	144	-	82	39.5	91	-	-
13	192	-	88	45.3	90	-	-
14	230	-	94	50	-	-	-

Table S6: Coupling of N-acetyl glycine nitrile (**1**) with L-alanine (**2_A**) catalyzed by 3-mercaptopropionic acid (**6b**) at various pH at room temperature

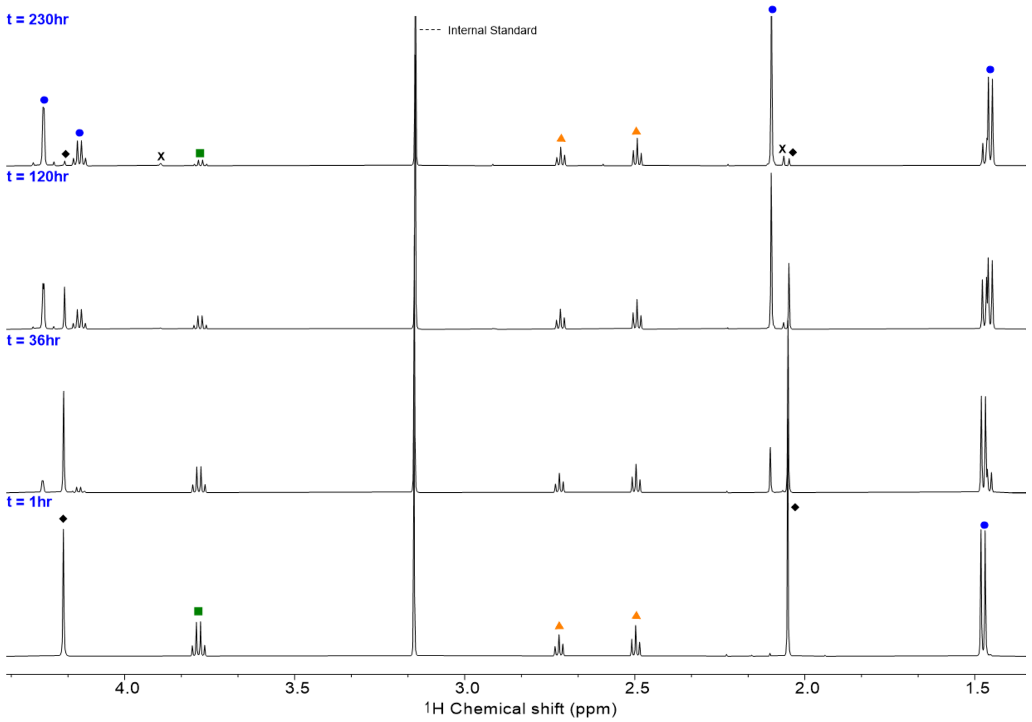

Figure S3: ^{1}H NMR (600 MHz, H_2O/D_2O 9:1, noesygppr1d, 1.4-4.5 ppm) spectrum to show the reaction of N-acetyl glycine nitrile (**1**, 200 mM) with L-alanine (**2_A**, 220 mM) and 3-mercaptopropanoic acid (**6b**, 60 mM), with MSM (100 mM) as an internal standard, at pH 7, room temperature.

Entry	Time / h	3_A / %
1	1	25
2	6	52
3	12	72
4	24	86
5	36	90
6	48	92
7	60	92

Table S7: Coupling of N-acetyl glycine nitrile (**1**) with L-alanine (**2A**) catalyzed by 3-mercaptopropionic acid (**6b**)at pH = 8.5, at room temperature

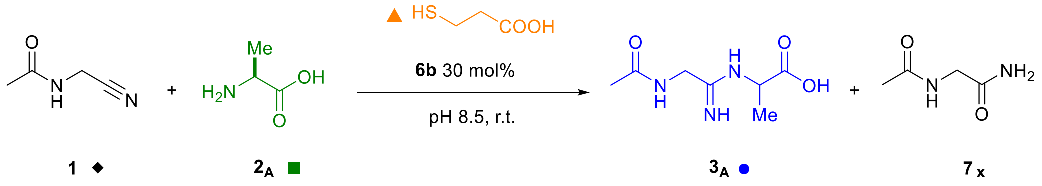

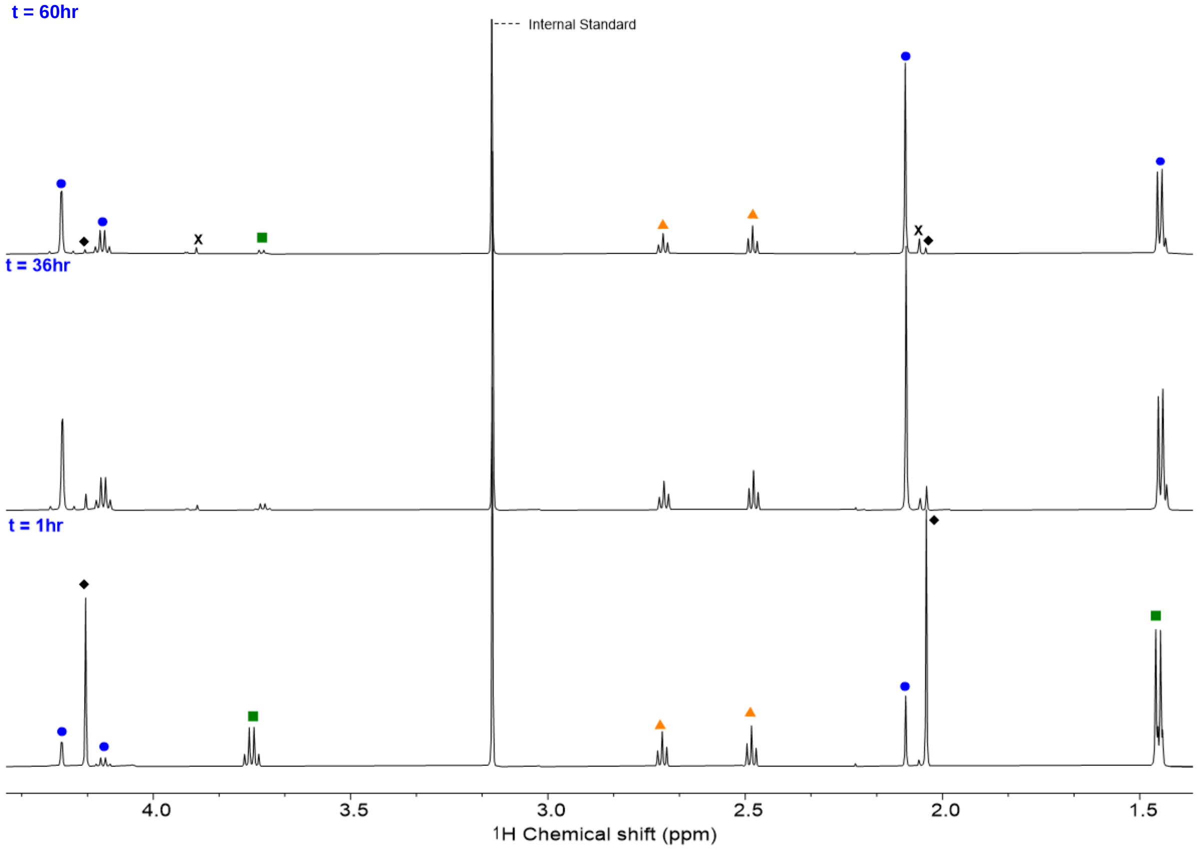

Figure S4: ^{1}H NMR (600 MHz, H_2O/D_2O 9:1, *noesygppr1d,* 1.4-4.5 ppm) spectrum to show the reaction of N-acetyl glycine nitrile (**1**, 200 mM) with L-alanine (**2A**, 220 mM) and 3-mercaptopropanoic acid (**6b**, 60 mM), with MSM (100 mM) as an internal standard, at pH 8.5, room temperature.

*Coupling of 1 with L-alanine **2_A** catalyzed by **6c** at room temperature*

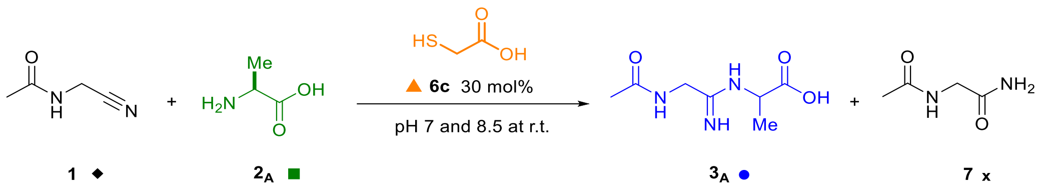

N-Acetylglycine nitrile (**1**, 200 mM), L-alanine (**2_A**, 220 mM), and thioglycolic acid (**6c**, 30 mol%, 60 mM) and MSM (4 mM) as internal standard were dissolved in H_2O/D_2O (9:1, 0.8 mL). The pH of the solution was adjusted to the desired value with 4 M HCl/NaOH, the solution volume was increased to 1 mL and NMR spectra were obtained periodically.

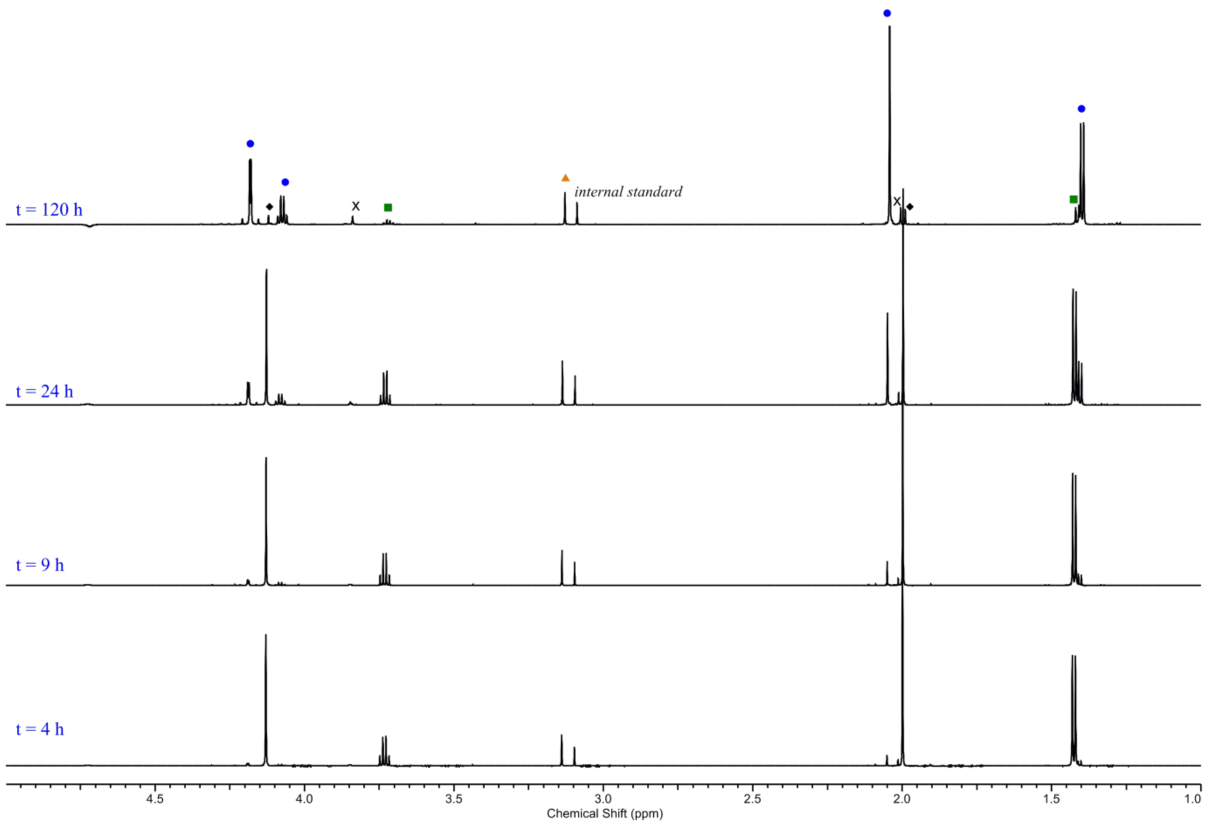

Figure S5: ^{1}H NMR (700 MHz, H_2O/D_2O 9:1, *noesygppr1d,* 1.0-5.0 ppm) spectrum to show the reaction of N-acetyl glycine nitrile (**1**, 200 mM) with L-alanine (**2_A**, 200 mM) and thioglycolic acid (**6c**, 60 mM), with MSM (4 mM) as an internal standard, at pH 7.0, room temperature.

Entry	time / h	**1** / %	**2_A** / %	**3_A** / %	**7** / %
1	4	92	92	5	3
2	9	88	88	11	3
3	15	81	82	18	3
4	26	67	70	33	4
5	36	57	61	44	4
6	48	43	48	59	5
7	74	20	26	82	6
8	120	8	14	90	7

Table S8. Yields over time for the reaction between N-acetyl glycine nitrile (**1**, 200 mM) with L-alanine (**2_A**, 200 mM) and thioglycolic acid (**6c**, 60 mM) at pH 7.0, room temperature.

N-Acetylglycine nitrile (**1**, 200 mM), L-alanine (**2**$_A$, 220 mM), and thioglycolic acid (**6c**, 30 mol%, 60 mM) and MSM (100 mM) as internal standard were dissolved in H_2O/D_2O (9:1, 0.8 mL). The pH of the solution was adjusted to the desired value with 4 M HCl/NaOH, the solution volume was increased to 1 mL and NMR spectra were obtained periodically.

Entry	Time / h	**pH = 5.3 3$_A$ / %**	**pH = 8.5, 3$_A$ / %**	**pH = 8.5 1 M borate, 3$_A$ / %**	**pH = 9.5, 3$_A$ / %**	**pH = 10 3$_A$ / %**
1	1	0	21	23.5	-	12
2	12	0	71	74	65	42.3
3	20	0	80	-	-	-
4	24	0	83	85	68	47
5	30	0	84.5	88	-	-
6	36	0	85	89	67.3	-
7	48	0	87	92	62	43.3
8	60	0	88.5	93	58	-
9	72	0	87	93	56	36.5
10	96	0	86	91	50	28
11	120	0	84.8	90	46	24.6

Table S9. Yields over time for the reaction between N-acetyl glycine nitrile (**1**, 200 mM) with L-alanine (**2**$_A$, 200 mM) and thioglycolic acid (**6c**, 60 mM) at various pH, room temperature.

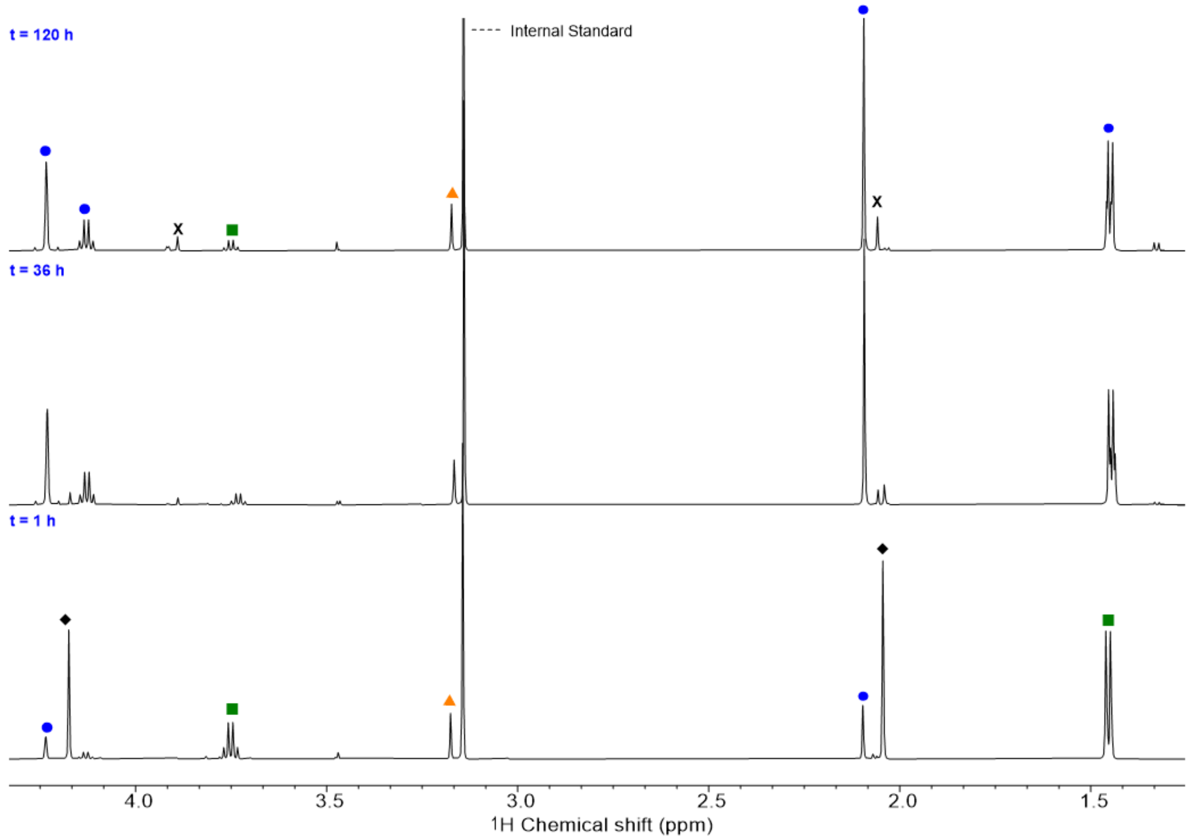

Figure S6: ^{1}H NMR (700 MHz, H_2O/D_2O 9:1, *noesygppr1d,* 1.3-4.5 ppm) spectrum to show the reaction of N-acetyl glycine nitrile (**1**, 200 mM) with L-alanine (**2**$_A$, 200 mM) and thioglycolic acid (**6c**, 60 mM), with MSM (100 mM) as an internal standard, at pH 8.5, room temperature.

Coupling of 1 with L-alanine ***2***

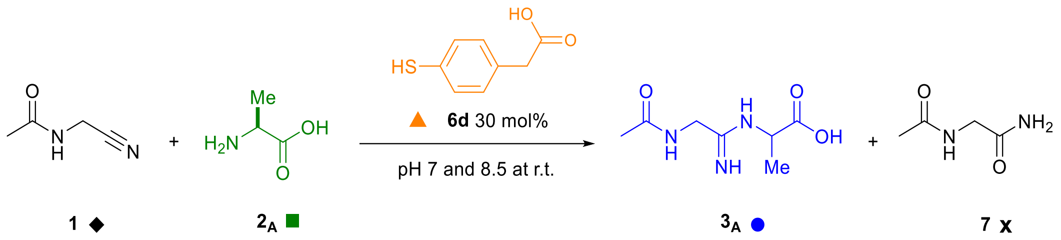

N-Acetylglycine nitrile (**1**, 200 mM), L-alanine (**2A**, 220 mM), and 4-mercaptophenylacetic acid (**6d**, 30 mol%, 60 mM) and MSM (100 mM) as an internal standard were dissolved in H_2O/D_2O (9:1, 0.8 mL). The pH of the solution was adjusted to the desired value with 4 M HCl/NaOH, the solution volume was increased to 1 mL and NMR spectra were obtained periodically.

Entry	Time / h	pH = 7 3A / %	pH =8.5 3A / %
1	1	0	0
2	12	7	2
3	24	12	3
4	36	17	4
5	48	21	7
7	72	30	9
8	96	36	12
9	120	43	13
10	144	48	16
11	168	50	18

Table S10: Coupling of N-acetyl glycine nitrile (**1**) with L-alanine (**2A**) catalyzed by 4-mercaptophenylacetic acid (**6d**) at pH = 7 and pH = 8.5 at room temperature

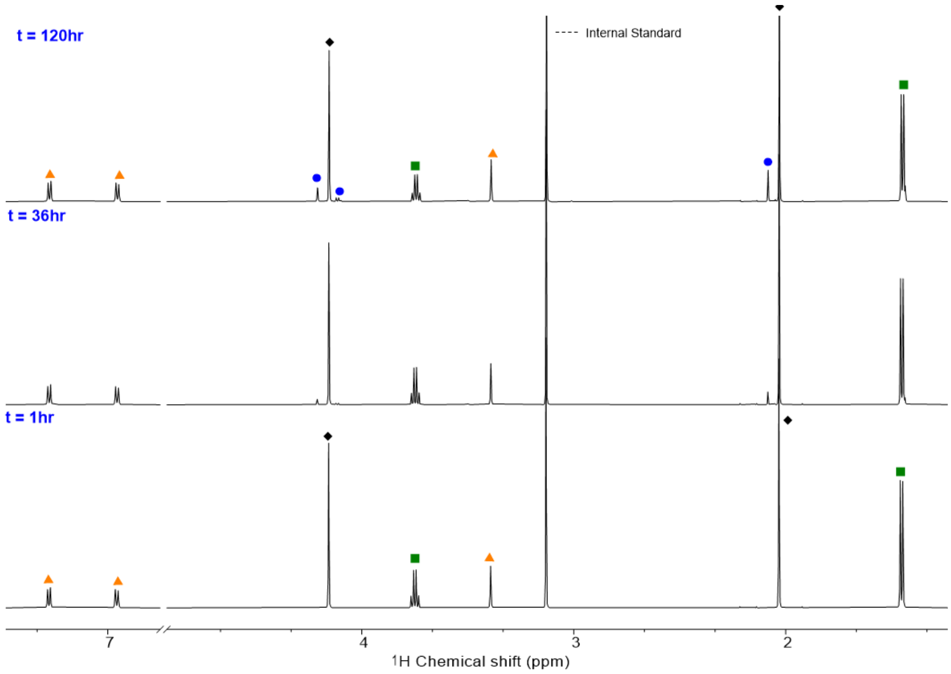

Figure S7: ^{1}H NMR (600 MHz, H_2O/D_2O 9:1, noesygppr1d, 1.1-7.5 ppm) spectrum to show the reaction of N-acetyl glycine nitrile (**1**, 200 mM) with L-alanine (**2A**, 220 mM) and 4-mercaptophenylacetic acid (**6d**, 60 mM), with MSM (100 mM) as an internal standard, at pH 8.5, room temperature.

*Coupling of 1 with L-alanine **2A** catalyzed by **6e** at room temperature*

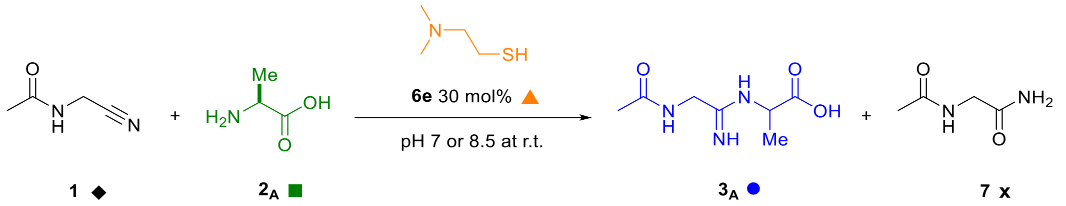

N-Acetylglycine nitrile (**1**, 200 mM), L-alanine (**2A**, 220 mM), and *N,N*-dimethyl cysteamine (**6e**, 30 mol%, 60 mM) and MSM (100 mM) as an internal standard were dissolved in H_2O/D_2O (9:1, 0.8 mL). The pH of the solution was adjusted to the desired value with 4 M HCl/NaOH, the solution volume was increased to 1 mL and NMR spectra were obtained periodically.

Entry	Time //h	pH = 7<br>3A /%	pH = 8.5<br>3A /%
1	1	2	10
2	12	12	19.3
3	24	22	32
4	36	28	41
5	48	35	50
7	72	41	57
8	96	48	61
9	120	52	65

Table S11: Coupling of N-acetyl glycine nitrile (**1**) with L-alanine (**2A**) catalyzed by *N,N*-dimethyl cysteamine (**6e**) at pH = 7 and pH = 8.5 at room temperature

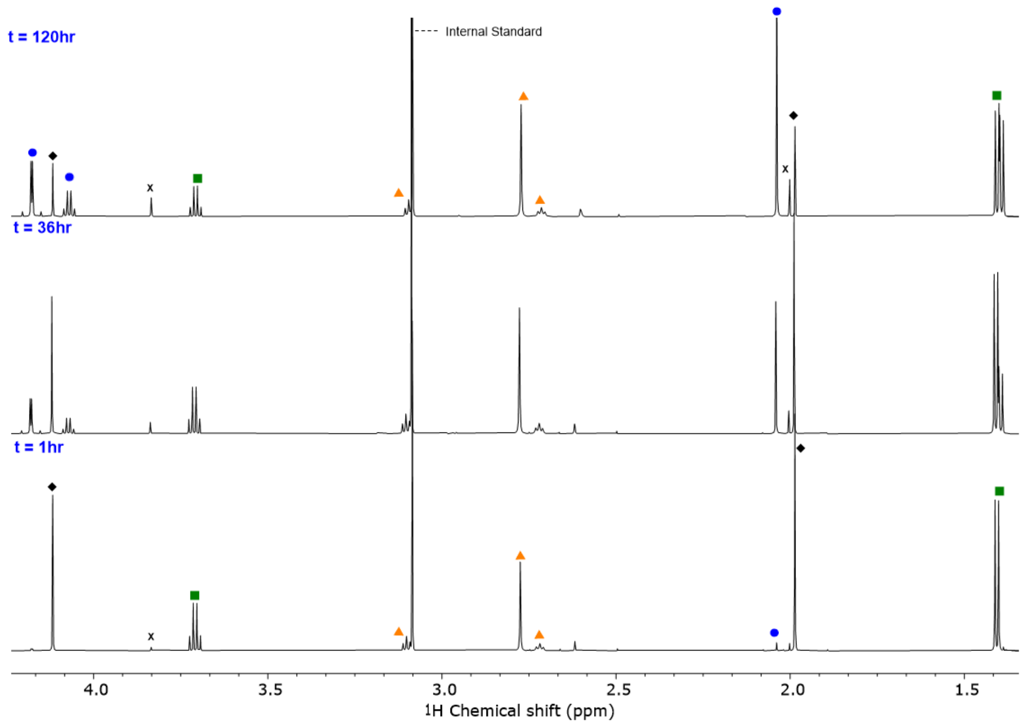

Figure S8: ^1H NMR (600 MHz, H_2O/D_2O 9:1, noesygppr1d, 1.4-4.2 ppm) spectrum to show the reaction of N-acetyl glycine nitrile (**1**, 200 mM) with L-alanine (**2A**, 220 mM) and *N,N*-dimethyl cysteamine (**6e**, 60 mM), with MSM (100 mM) as an internal standard, at pH 8.5, room temperature.

*Coupling of 1 with L-alanine **2A** catalyzed by **6f** at room temperature*

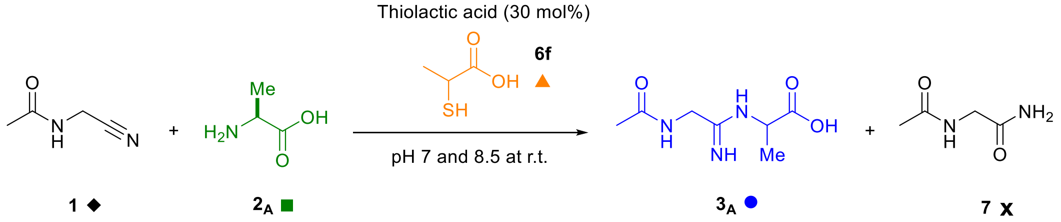

N-Acetylglycine nitrile (**1**, 200 mM), L-alanine (**2A**, 220 mM), and thiolactic acid (**6f**, 30 mol%, 60 mM) and MSM (100 mM) as an internal standard were dissolved in H_2O/D_2O (9:1, 0.8 mL). The pH of the solution was adjusted to the desired value with 4 M HCl/NaOH, the solution volume was increased to 1 mL and NMR spectra were obtained periodically.

Entry	Time //h	**3A** /%
1	1	1
2	12	4.3
3	24	8.3
4	36	11
5	48	14
7	72	20
8	96	25
9	120	29

Table S12. Coupling of N-acetyl glycine nitrile (**1**) with L-alanine (**2A**) catalyzed by 2-mercaptopropionic acid (**6f**) at pH = 7, at room temperature

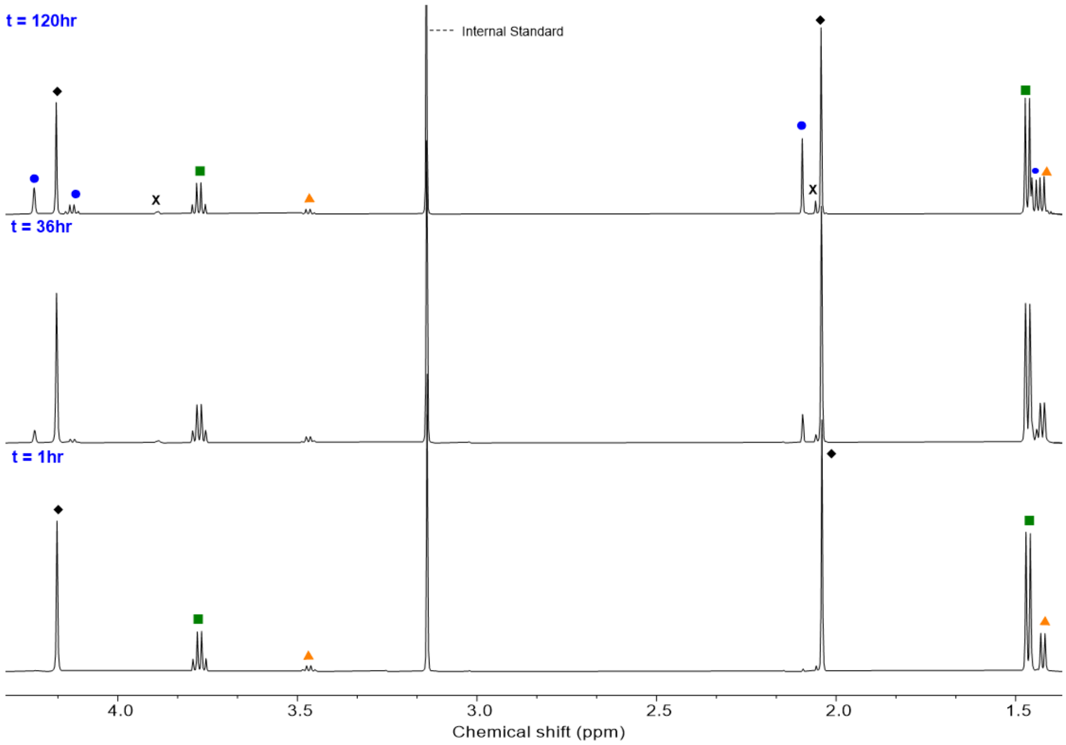

Figure S9: ^{1}H NMR (600 MHz, H_2O/D_2O 9:1, noesygppr1d, 1.4-4.3 ppm) spectrum to show the reaction of N-acetyl glycine nitrile (**1**, 200 mM) with L-alanine (**2A**, 220 mM) and 2-mercaptopropionic acid (**6f**, 60 mM), with MSM (100 mM) as an internal standard at pH 7, room temperature.

Entry	Time /h	3_A /%
1	1	8
2	12	34
3	24	49
4	36	60
5	48	67
7	72	73
8	96	77
9	120	79

Table S13: Coupling of N-acetyl glycine nitrile (**1**) with L-alanine (**2A**) catalyzed by 2-mercaptopropionic acid (**6f**) at pH = 8.5, at room temperature

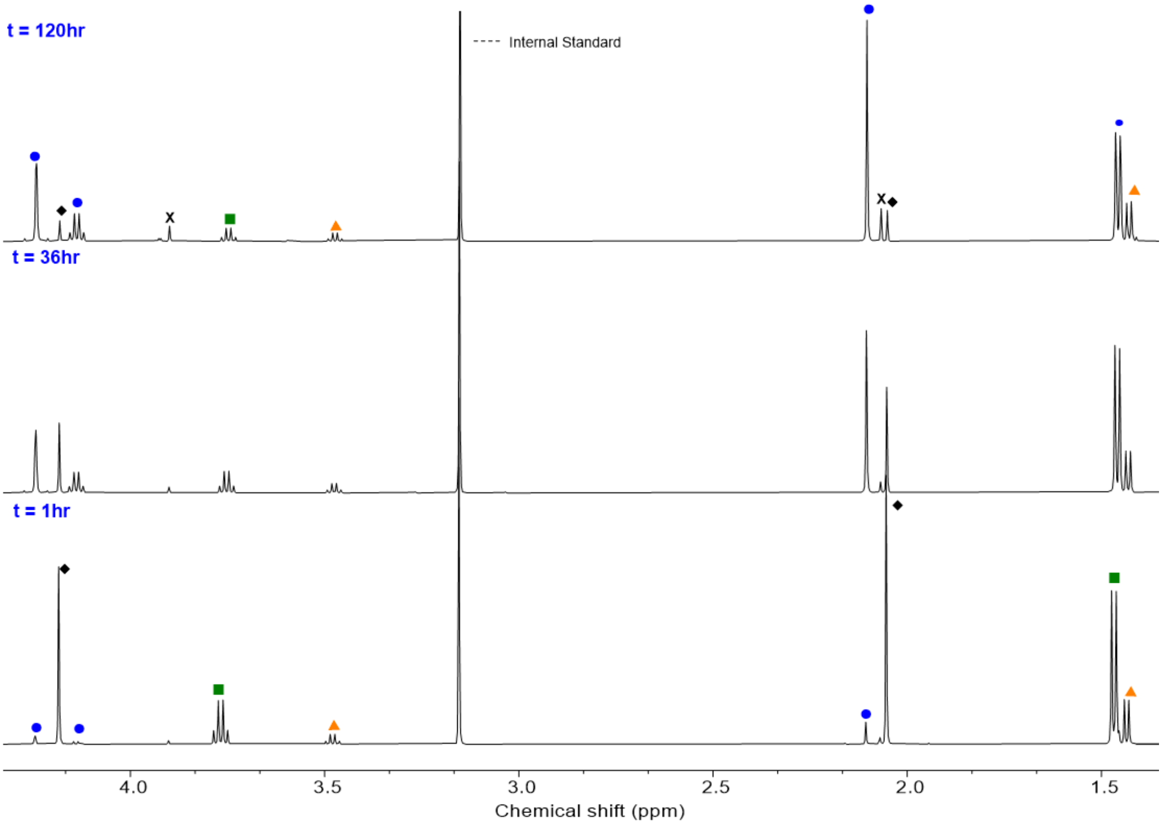

Figure S10: ^{1}H NMR (600 MHz, H_2O/D_2O 9:1, noesygppr1d, 1.4-4.3 ppm) spectrum to show the reaction of N-acetyl glycine nitrile (**1**, 200 mM) with L-alanine (**2A**, 220 mM) and 2-mercaptopropionoic acid (**6f**, 60 mM), with MSM (100 mM) as an internal standard, at pH 8.5, room temperature.

*Coupling of 1 with L-alanine **2A** catalyzed by **6g** at room temperature*

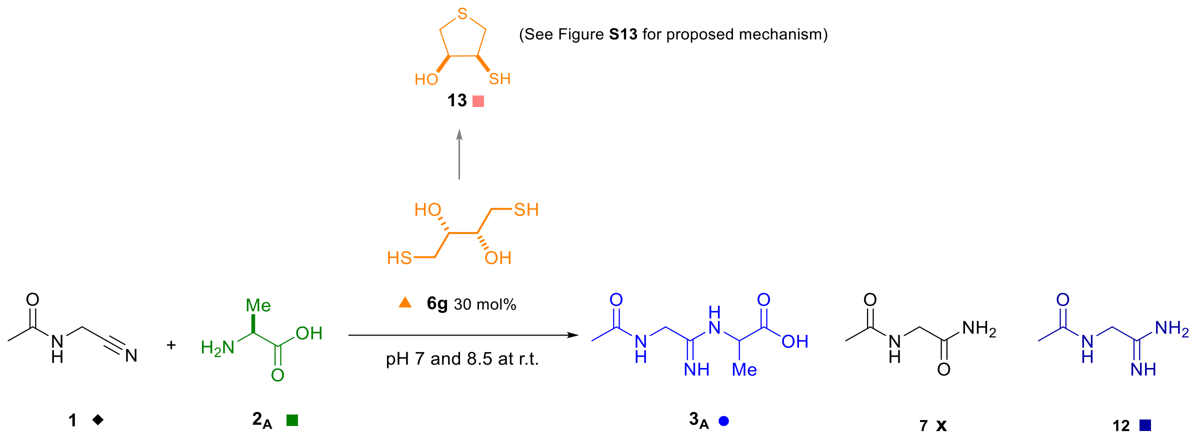

N-Acetylglycine nitrile (**1**, 200 mM), L-alanine (**2A**, 220 mM), and dithiothreitol (**6g**, 30 mol%, 60 mM) and MSM (100 mM) as internal standard were dissolved in H_2O/D_2O (9:1, 0.8 mL). The pH of the solution was adjusted to the desired value with 4 M HCl/NaOH, the solution volume was increased to 1 mL and NMR spectra were obtained periodically.

^1H NMR (600 MHz, H_2O/D_2O 9:1) *cis-4-Hydroxy-3-mercaptotetrahydrothiophene*, **13** (■): 2.77 (1H, t, *J* = 10.3 Hz, -SC*H*HCHSH), 2.87 (1H, dd, *J* = 11.7, 2.8 Hz, -SC*H*HCHOH), 3.09 (1H, dd, *J* = 11.7, 2.8 Hz, -SCH*H*CHOH), 3.16 (1H, dd, *J* = 10.3, 6.6 Hz, -SCH*H*CHSH), 3.38 (1H, ddd, *J* = 10.0, 6.8, 3.3 Hz, -SCHHC*H*SH), 4.41 – 4.34 (1H, m, -SCHHC*H*OH). The data is consistent with literature characterisation of **15.**[2]

Entry	Time /h	**3A / %**	**7 / %**	**12 / %**
1	6	35	10	5
2	12	50	15	8
3	24	58	19	9
4	36	60	20	9.5
5	72	65	22	9.5
7	96	68	23	7.6
8	120	68	23	7.6

Table S14. Coupling of N-acetyl glycine nitrile (**1**) with L-alanine (**2A**) catalyzed by dithiothreitol (**6g**) at pH = 7, at room temperature

Entry	Time /h	**3A / %**	**7 / %**	**10 / %**
1	1	12	4.5	0
2	12	50	17	6
3	24	61	21	7
4	36	65	23	6
5	72	68	24	6
7	96	68	24	6.3
8	120	69	25	6

Table S15. Coupling of N-acetyl glycine nitrile (**1**) with alanine (**2A**) catalyzed by dithiothreitol (**6g**) at pH = 8.5, at room temperature

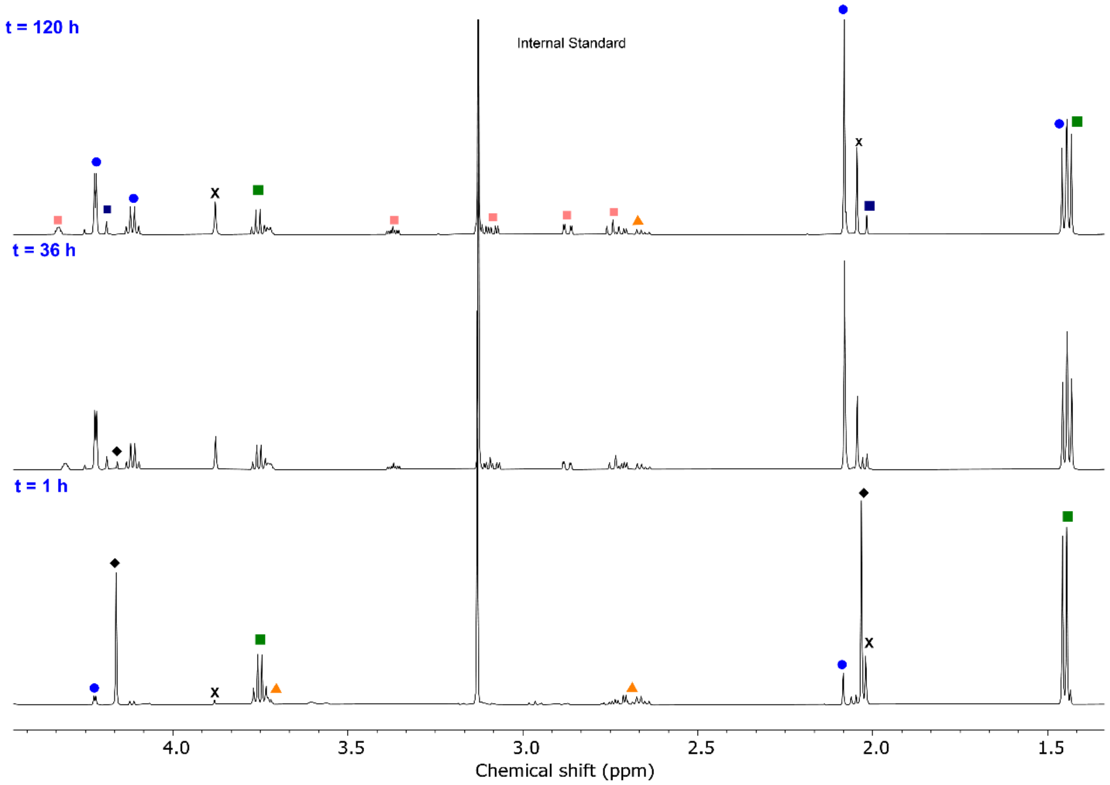

Figure S11: ^{1}H NMR (600 MHz, H_2O/D_2O 9:1, noesygppr1d, 1.4-4.3 ppm) spectrum to show the reaction of N-acetyl glycine nitrile (**1**, 200 mM) with L-alanine (**2**$_{\mathbf{A}}$, 240 mM) and dithiothreitol (**6g**, 60 mM), with MSM (100 mM) as an internal standard, at pH 8.5, room temperature.

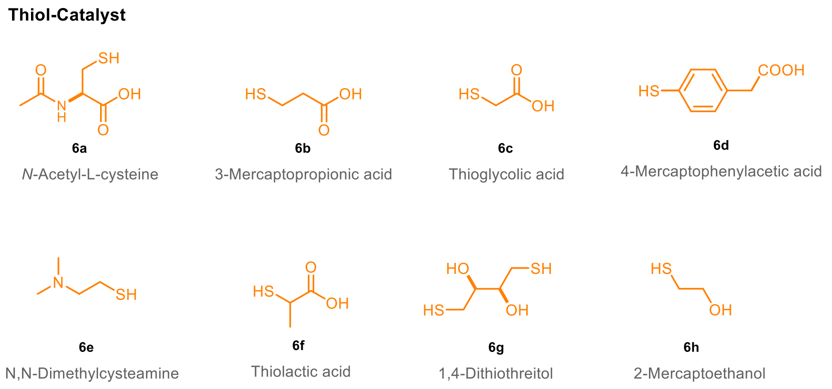

Entry	thiol catalyst	pH	Time to max yield / h	**3**$_A$ / %	**7** / %
1	**6a**	7	196	84	5
2	**6b**	7	196	88	4
3	**6c**	7	120	92	4
4	**6d**	7	168	50	3
5	**6e**	7	120	52	23
6	**6f**	7	120	29	5
7	**6g**	7	36	70	19
8	**6a**	8.5	120	84	7
9	**6b**	8.5	48	92	4
10	**6c**	8.5	48	87	4
11	**6d**	8.5	168	18	2
12	**6e**	8.5	120	65	11
13	**6f**	8.5	96	78	7
14	**6g**	8.5	48	70	23

Table S16. Comparison of final yields for coupling of **1** and **2**$_A$ catalyzed by different thiols.

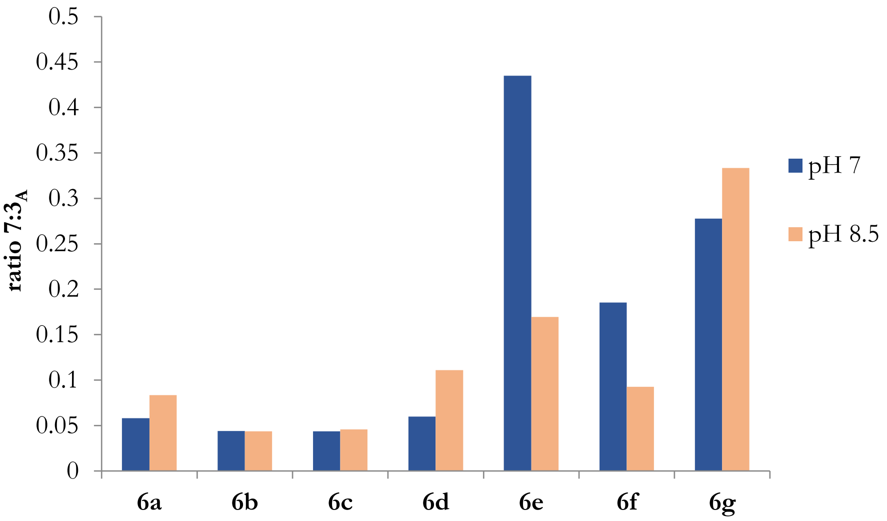

Figure S12: The effect of different thiol catalysts on selectivity in the reaction between **1** and **2**$_A$. Ratios of hydration:ligation are taken at the point of maximum yield for each reaction (see Table S16).

With **6g** as catalyst, despite showing fast and relatively high yielding formation of **3**$_A$, an unusually high yield of the hydration product **7** was observed, alongside disappearance of the ^{1}H NMR signals for **6g** at 2.7 ppm. The product

derived from **6g** is consistent with literature data for substituted tetrahydrothiophene **13**. We propose that formation of this species occurs alongside hydration, via a mechanism such as the one shown in figure **S12** which proceeds through thioimidate formation, S-to-O acyl transfer, substitution of the resulting isoamide via formation of a 3-membered thiirane formation adjacent thiol, and finally nucleophilic attack by the second thiol to form a more stable 5-membered ring.

To probe this mechanism further we investigated a simpler thiol, 2-mercaptoethanol **6h**. This thiol has the same 1,2 thiol-alcohol substitution pattern which should allow it to form a thiirane via this mechanism, but lacks the second thiol so thiirane should be visible by NMR.

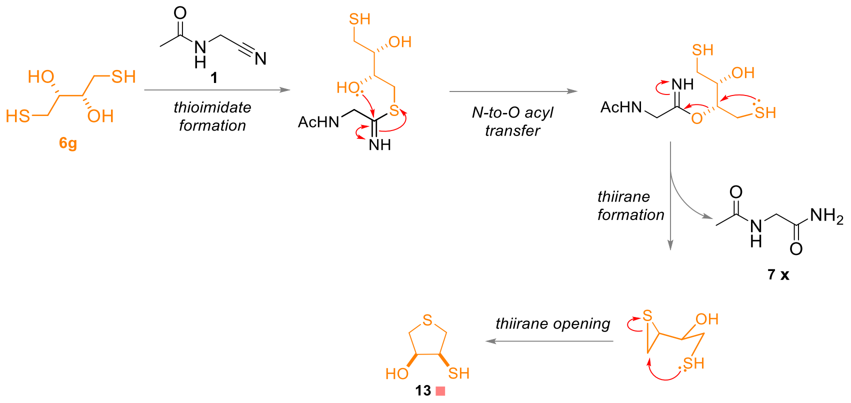

Figure S13. Proposed mechanism for dithiothreitol **6g**-mediated hydration N-acetyl Glycine nitrile **1** leading to formation of **13** and **7**

*Coupling of 1 with L-alanine **2A** catalyzed by **6h** at room temperature*

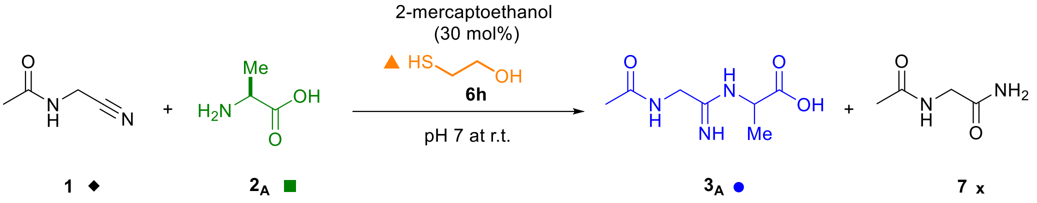

N-Acetylglycine nitrile (**1**, 200 mM), L-alanine (**2A**, 220 mM), and 2-mercaptoethanol (**6h**, 30 mol%, 60 mM) were dissolved in H_2O/D_2O (9:1, 0.8 mL) along with MSM (14 mM) as internal standard. The pH of the solution was adjusted to the desired value with 4 M HCl/NaOH, the solution volume was increased to 1 mL and NMR spectra were obtained periodically.

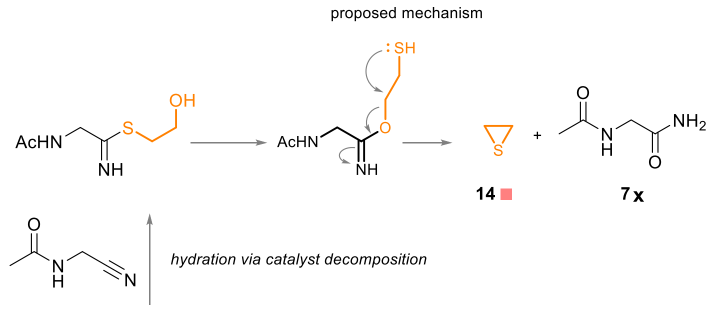

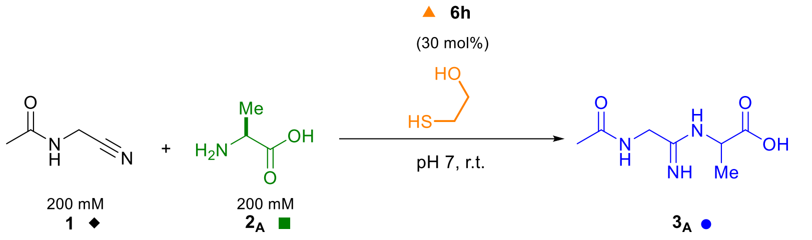

Entry	time / h	**1** / %	**3A** / %	**7** / %
1	1	89	0	5
2	5	82	1	12
3	8	77	1	17
4	16	67	2	26
5[a]	***24***	***62***	***3***	***30***
6	36	58	6	31
7	48	57	7	31
8	72	53	10	31
9	120	52	12	32

Table S17. Yields over time for the reaction between N-acetyl glycine nitrile (**1**, 200 mM) with L-alanine (**2A**, 200 mM) and 2-mercaptoethanol (**6h**, 60 mM) at pH 7.0, room temperature. [a] At this time **6h** was no longer visible by ^{1}H NMR (see Figure S14)

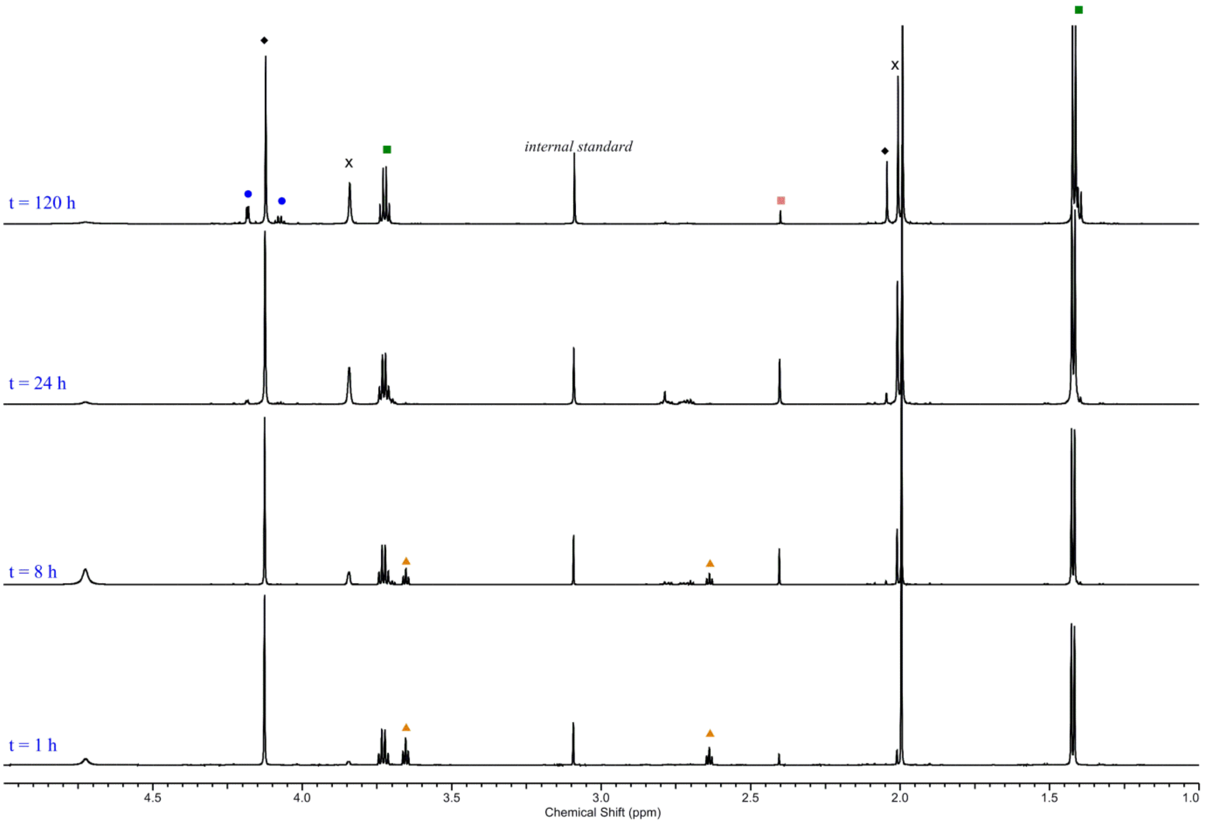

Figure S14: ^{1}H NMR (700 MHz, H_2O/D_2O 9:1, noesygppr1d, 1.0-5.0 ppm) spectrum to show the reaction of N-acetyl glycine nitrile (**1**, 200 mM) with L-alanine (**2$_A$**, 220 mM) and 2-mercaptoethanol (**6h**, 60 mM) with MSM (7 mM) as an internal standard, at pH 7.0, room temperature.

6h is not an effective catalyst for the coupling between **1** and **2$_A$** at pH 7. Instead, **6h** promotes hydration of **1** to yield **7**. **6h** is completely consumed (>99% by NMR spectroscopy) after 24 h. The formation of thiirane **14** (2.4 ppm) is observed, alongside further thiol-derived signals at 2.6-2.8 and 3.7-3.8 ppm. After full consumption of **6h** (24 h), ligation between **1** and **2$_A$** occurs, suggesting that the thiol-derived products of **6h** degradation are competent catalysts (see Table S6, entries 5-9).

To further demonstrate that hydration of **1** is linked to decomposition of **6h** via formation of thiirane **14** we investigated the reaction between **6h** and **1**.

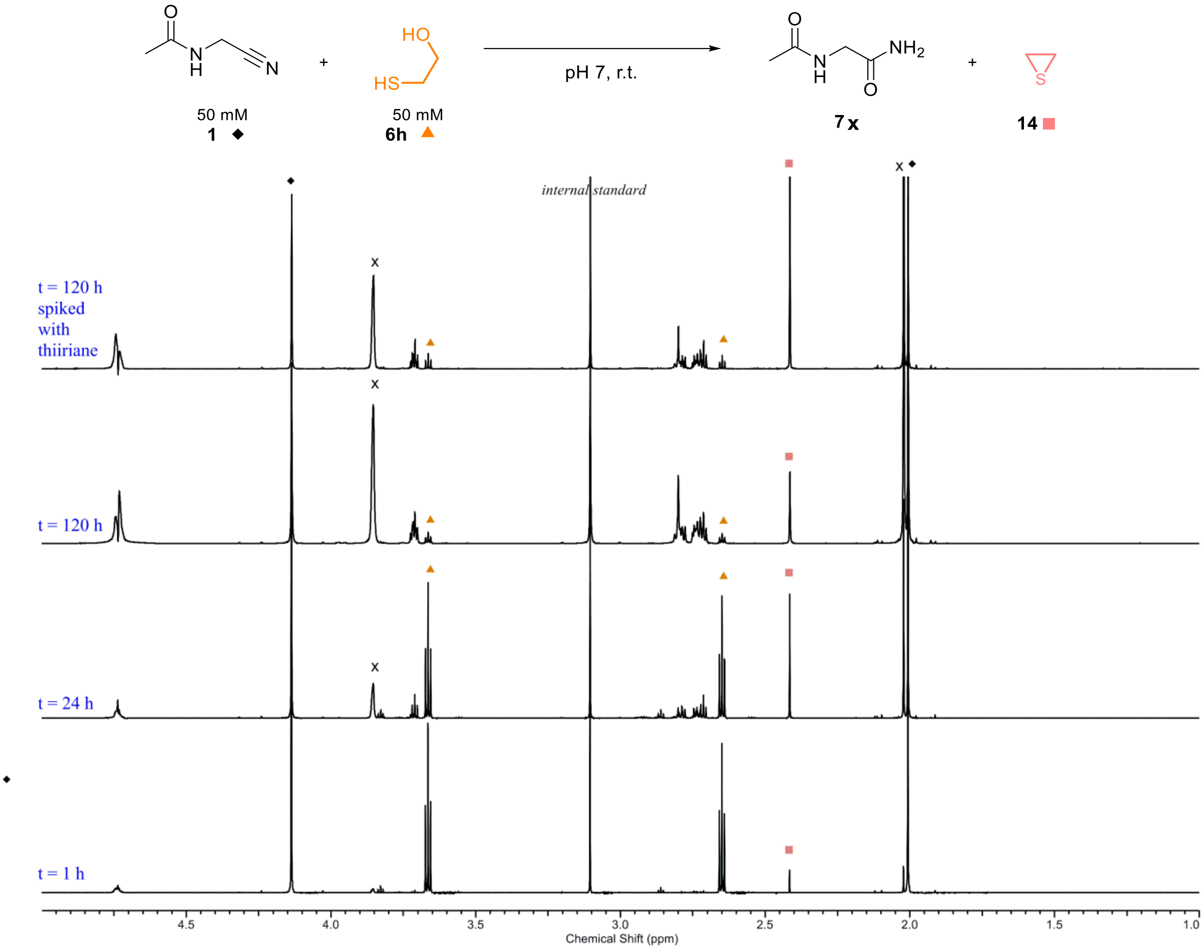

Figure S15: ^{1}H NMR (700 MHz, H_2O/D_2O 9:1, noesygppr1d, 1.0-5.0 ppm) spectrum to show the reaction of N-acetyl glycine nitrile (**1**, 50 mM) with 2-mercaptoethanol (**6h**, 50 mM) with MSM (7 mM) as an internal standard, at pH 7.0, room temperature. Formation of thiirane **14** was confirmed by spiking with an authentic sample (10 mM).

6h and **1** react to form the hydration product **7** and thiirane **14**, alongside further thiol-derived products that seem to derive from the addition of **6h** to **14**.

*Coupling of N-acetylglycine nitrile **1** with a-amino acids **2** at pH 8.5 and room temperature*

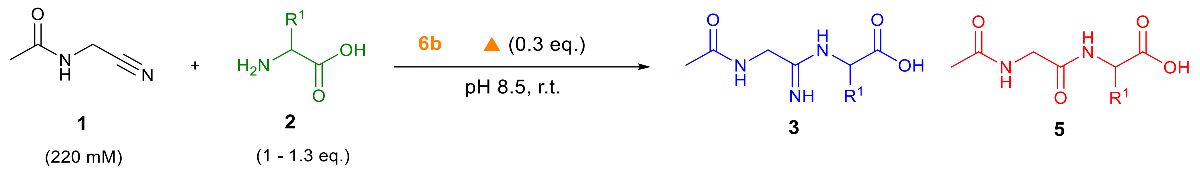

Amino acid 2	Yield /%		HRMS-ESI for amidines 3		
	3 ●	5 ▼	Formula	Theoretical	Found
Gly, $\mathbf{2_G}$	>95%	-----	$C_6H_{12}N_3O_3$ [M+H]$^+$	174.0874	174.0874
L-Ala, $\mathbf{2_A}$	87%	-----	$C_7H_{14}N_3O_3$ [M+H]$^+$	188.1030	188.1027
L-Arg[a], $\mathbf{2_R}$	91%	-----	$C_{10}H_{21}N_6O_3$ [M+H]$^+$	273.1670	273.1669
L-Asn[a], $\mathbf{2_N}$	40%[b]	53%	$C_8H_{14}N_3O_5$ [M+H]$^+$	232.0928	232.0933
L-Asp, $\mathbf{2_D}$	91%	-----	$C_8H_{14}N_3O_5$ [M+H]$^+$	232.0928	232.0931
L-Gln[a], $\mathbf{2_Q}$	90%	-----	$C_9H_{17}N_4O_4$ [M+H]$^+$	245.1245	245.1244
L-Glu, $\mathbf{2_E}$	90%	-----	$C_9H_{16}N_3O_5$ [M+H]$^+$	246.1084	246.1086
L-His, $\mathbf{2_H}$	93%	-----	$C_{10}H_{16}N_5O_3$ [M+H]$^+$	254.1248	254.1249
L-Ile[a], $\mathbf{2_I}$	86%	-----	$C_{10}H_{20}N_3O_3$ [M+H]$^+$	230.1499	230.1498
L-Leu, $\mathbf{2_L}$	84%	-----	$C_{10}H_{20}N_3O_3$ [M+H]$^+$	230.1499	230.1498
L-Lys[a1], $\mathbf{2_K}$	>90%[c]	-----	$C_{10}H_{21}N_4O_3$ [M+H]$^+$	245.1068	245.1067
DL-Met, $\mathbf{2_M}$	89%	-----	$C_9H_{18}N_3O_3S$ [M+H]$^+$	248.1063	248.1064
L-Phe, $\mathbf{2_F}$	86%	-----	$C_{13}H_{18}N_3O_3$ [M+H]$^+$	264.1343	264.1343
L-Pro, $\mathbf{2_P}$	83%	-----	$C_9H_{16}N_3O_3$ [M+H]$^+$	214.1187	214.1196
L-Ser, $\mathbf{2_S}$	-----	95%[d]	$C_7H_{12}N_2O_5$ [M+Na]$^+$	227.0638	227.0643
L-Thr[a], $\mathbf{2_T}$	-----	93%[e]	$C_8H_{15}N_2O_5$ [M+H]$^+$	219.0976	219.0979
L-Trp[a], $\mathbf{2_W}$	83%[f]	-----	$C_{15}H_{19}N_4O_3$ [M+H]$^+$	303.1452	303.1451
L-Val[a], $\mathbf{2_V}$	93%	-----	$C_9H_{18}N_3O_3$ [M+H]$^+$	216.1343	216.1339

Table S18: Yields and ESI-HRMS data for 3-mercaptopropanoic acid (**6b**, 60 mM) ▲ catalyzed formation of amidine **3** ● and peptide **5** ▼ from the coupling of N-acetyl glycine nitrile (**1,** 200 mM) with amino acid ■ (**2**, 1.0 - 1.3 equiv.) at pH 8.5, room temperature, after 24 h, unless stated otherwise.

[a] Valine $\mathbf{2_V}$, isoleucine $\mathbf{2_I}$, arginine $\mathbf{2_R}$, lysine $\mathbf{2_K}$, glutamine $\mathbf{2_Q}$, tryptophan $\mathbf{2_W}$, threonine $\mathbf{2_T}$, and asparagine $\mathbf{2_N}$ were poorly soluble in water, therefore each was dissolved with NaOH (1 equiv.) and then adjusted to pH 9 with HCl. An aliquot of pH 9 amino acid solution was used in each ligation reaction. The ligation reaction pH was adjusted to the desired pH at t = 0.

[b] The reaction progress was monitored for 5 days to ensure that all in situ formed intermediates were hydrolysed to $\mathbf{5_N}$ and $\mathbf{3_D}$. **8** is observed to form from $\mathbf{3_N}$, and was detected in the first NMR acquired (t = 12 h). **Figure S22** depicts the progression of the reaction.

[c] Combined yield for the N^2, N^6, and N^2,N^6-bis-acyl coupling products reported: (N^2-(Ac-Gly^N)-Lys-OH, plus N^2,N^6-bis(Ac-Gly^N)-Lys-OH (62%) and N^6-(Ac-Gly^N)-Lys-OH plus N^2,N^6-bis(Ac-Gly^N)-Lys-OH (32%). **Figure S37**

[d] An intermediate oxazole, 2-(acetamidomethyl)-4,5-dihydrooxazole-4-carboxylic acid, $\mathbf{9_S}$ (55% at 24 h) was observed, after complete consumption of **1**, the reaction mixture was heated at 60°C for 12 h, and complete conversion of $\mathbf{9_S}$ to $\mathbf{5_S}$ was observed. **Figure S45**.

[e] An intermediate oxazole, (2-(acetamidomethyl)-5-methyl-4,5-dihydrooxazole-4-carboxylic acid $\mathbf{9_T}$ (67% at 24 h) was observed. The reaction mixture was incubated at 60°C for 12 h and hydrolysis of $\mathbf{9_T}$ to $\mathbf{5_T}$ was observed. **Figure S49.**

[f] L-Tryptophan $\mathbf{2_W}$ exhibits low solubility in water, therefore after dissolving all the substrates reaction mixture was heated for 10 mins (temperature approx. ~60°C). Afterwards reaction progress was followed by NMR at room temperature. No further attempts were made to optimise $\mathbf{2_W}$ coupling.

*Coupling of **1** with L-alanine **2_A** at pH 8.5 and room temperature*

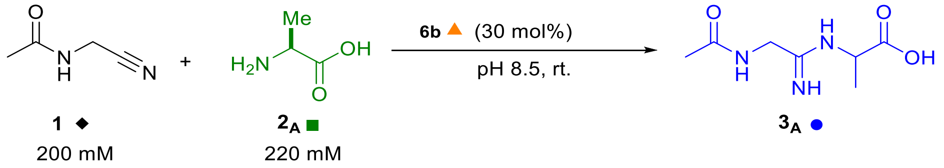

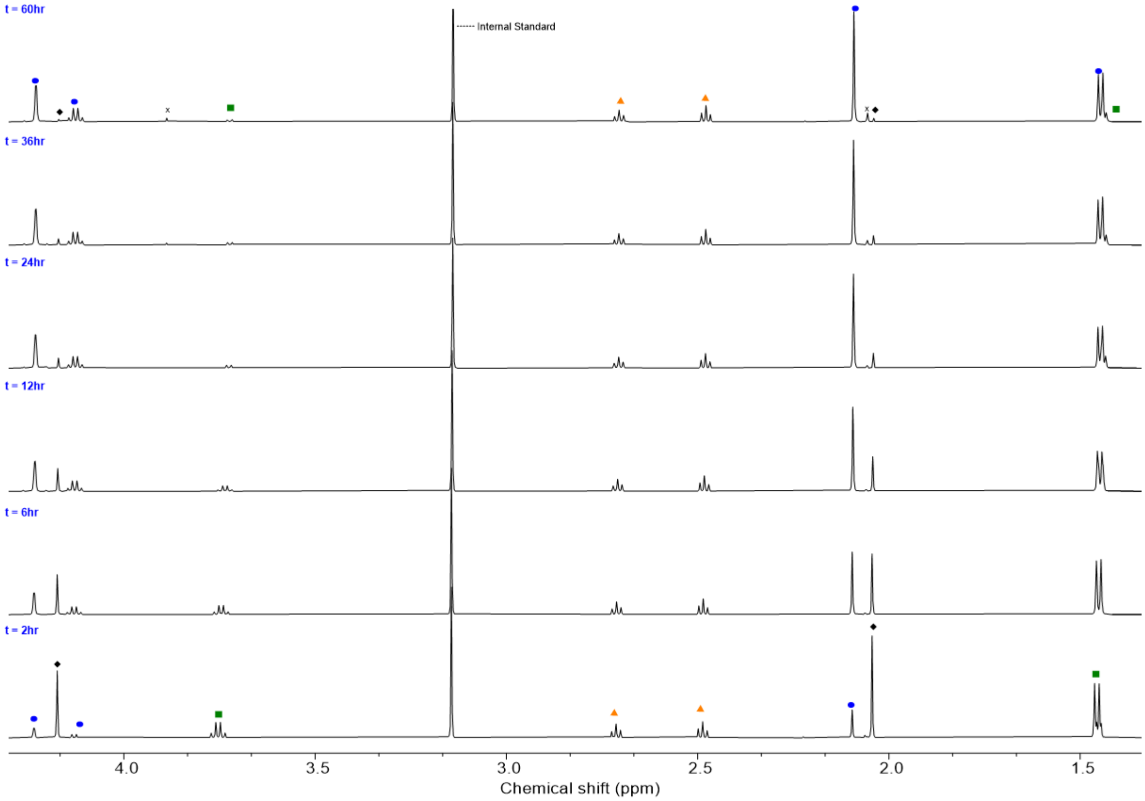

Figure S16: ^{1}H NMR (600 MHz, H_2O/D_2O 9:1, noesygppr1d, 1.4-4.3 ppm) spectrum to show the reaction of N-acetyl glycine nitrile (**1**, 200 mM) with L-alanine (**2_A**, 220 mM) and 3-mercaptopropanoic acid (**6b**, 60 mM), with MSM (100 mM) as an internal standard, at pH 8.5, room temperature.

^{1}H NMR (600 MHz, H_2O/D_2O 9:1) *(2-acetamido-1-iminoethyl)-L-alanine*, **3_A** (●): δ_H 4.26 (1H, AB, *J* = 17.2 Hz, AcNHCH*H*), 4.22 (1H, AB, *J* = 17.2 Hz, AcNHCH*H*), 4.11 (1H, q, *J* = 7.1 Hz, C*H*(CH_3)), 2.10 (3H, s, *H_3*C(CO)), 1.46 (3H, d, *J* = 7.2 Hz, CH(C*H_3*)); *L-alanine*, ***2_A*** (■): δ_H 3.78 (1H, q, *J* = 7.2 Hz, C*H*(CH_3)), 1.47 (3H, d, *J* = 7.2 Hz, CH(C*H_3*)); *N-acetylglycinamide*, **7** (✖) (partial assignment): δ_H 3.89 (2H, d, C*H_2*).

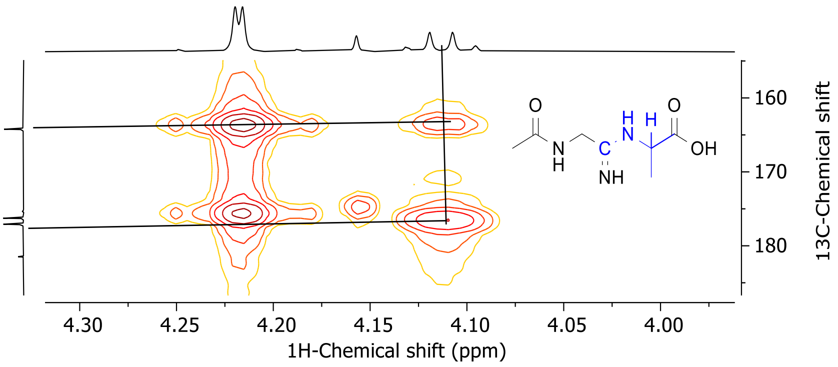

Figure S17: ^{1}H–^{13}C HMBC (^{1}H: 600 MHz [3.5-4.5 ppm], ^{13}C: 176 MHz [150-185 ppm], H_2O/D_2O 9:1) spectrum showing the diagnostic $^2J_{CH}$ and $^3J_{CH}$ coupling of **Ala**-αH-COOH in **3$_A$** at 4.11 ppm with two resonances at 177.5 and 164.5 ppm, which is characteristic of amidine bond formation of **2$_A$**.

*Coupling of **1** with L-aspartic acid **2D** at pH 8.5 and room temperature*

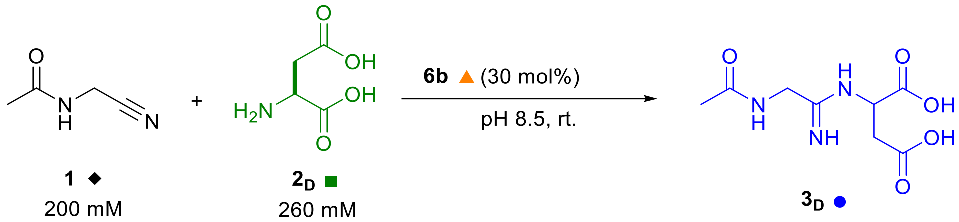

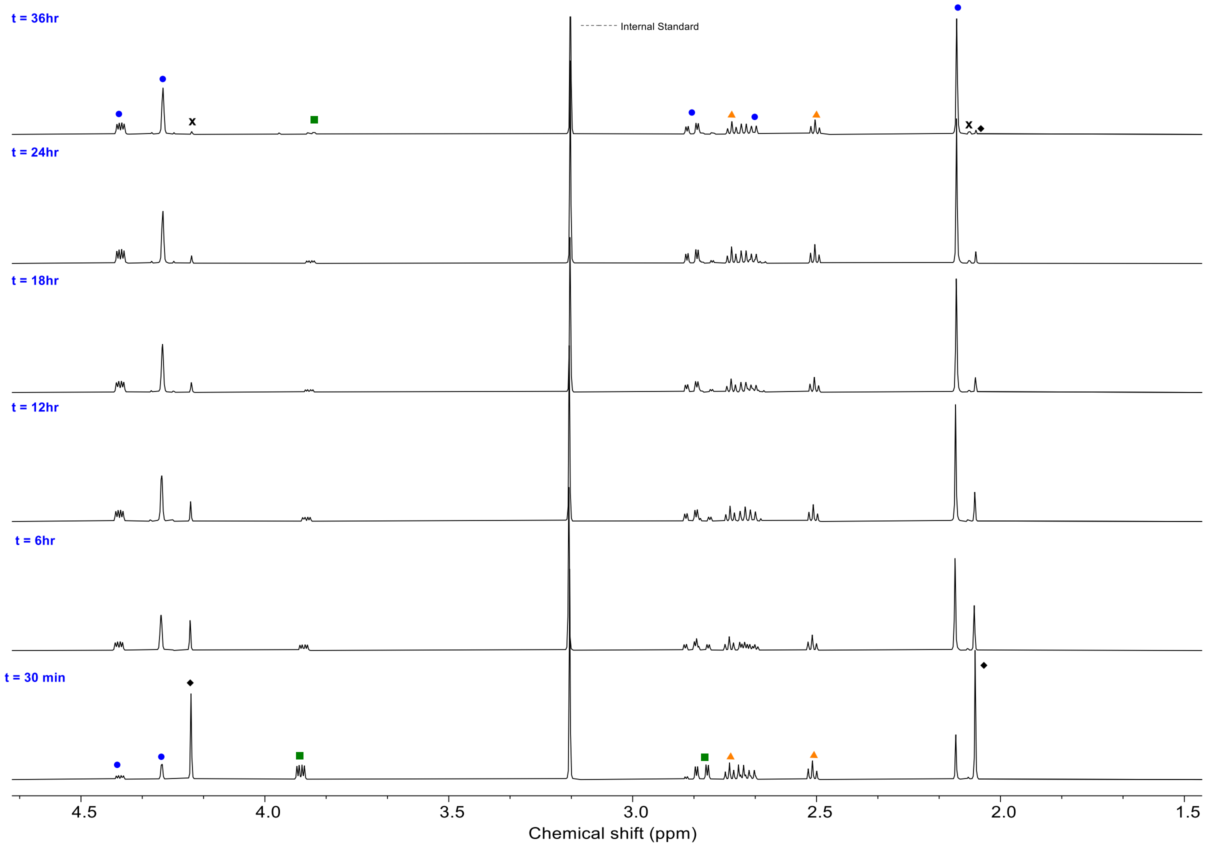

Figure S18: ^{1}H NMR (600 MHz, H_2O/D_2O 9:1, noesygppr1d, 1.0-5.0 ppm) spectrum to show the reaction of *N*-acetyl glycine nitrile (**1**, 200 mM) with L-aspartic acid (**2D**, 260 mM) and 3-mercaptopropanoic acid (**6b**, 60 mM) with MSM (100 mM) as an internal standard at pH 8.5 and room temperature.

^{1}H NMR (600 MHz, H_2O/D_2O 9:1, noesygppr1d) *(2-acetamido-1-iminoethyl)aspartic acid*, **3D** (●): δ_H 4.38 (1H, dd, *J* = 8.3, 3.7 Hz, Asp-αH-COOH), 4.26 (1H, AB, *J* = 17.3 Hz, AcNHCH*H*), 4.23 (1H, AB, *J* = 17.3 Hz, AcNHCH*H*), 2.84 (1H, ABX, *J* = 16.6, 3.7 Hz, CH(CH*H*COOH)COOH), 2.66 (1H , ABX, *J* = 16.6, 8.3 Hz, CH(CH*H*COOH)COOH), 2.10 (3H, s, *H*$_3$C(CO)); *L-aspartic acid*, **2D** (■): δ_H 3.87 (1H, dd, *J* = 9.0, 3.8 Hz, αH-COOH), 2.78 (1H, ABX, *J* = 17.2, 3.8 Hz, CH(CH*H*COOH)COOH), 2.62 (1H, dd, *J* = 17.1, 9.0 Hz, CH(CH*H*COOH)COOH); *N-acetylglycinamide*, **7** (✖): δ_H 3.94 (2H, s, C*H*$_2$).

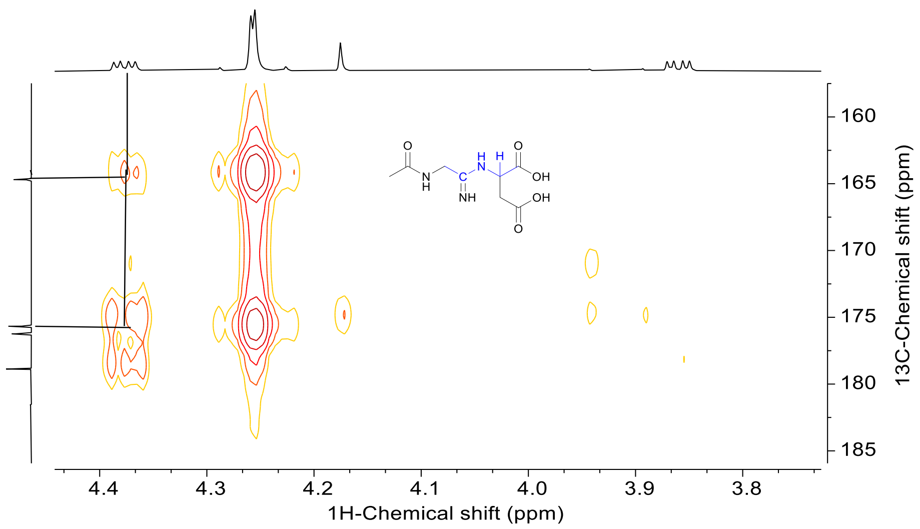

Figure S19: ${}^{1}H$–${}^{13}C$ HMBC (${}^{1}H$: 600 MHz [3.5-5.0 ppm], ${}^{13}C$: 176 MHz [160-185 ppm], H_2O/D_2O 9:1) spectrum showing the diagnostic ${}^{2}J_{CH}$ and ${}^{3}J_{CH}$ coupling of the **Asp**-αH-COOH in **3**$_{\mathbf{D}}$ at 4.37 ppm with two resonances at 175.6 and 165.4 ppm, which is characteristic of amidine bond formation of **2**$_{\mathbf{D}}$.

*Coupling of **1** with L-arginine **2**$_R$ at pH 8.5 and room temperature*

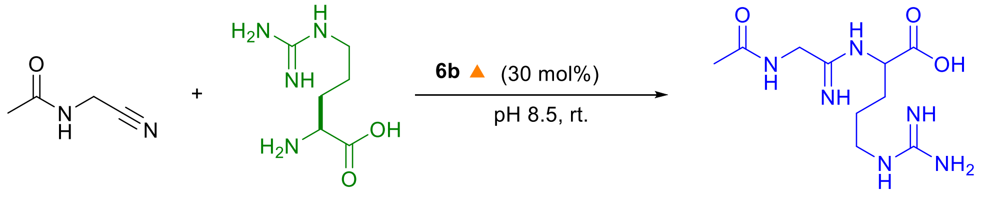

1 ◆ 200 mM   **2**$_R$ ■ 240 mM   **3**$_R$ ●

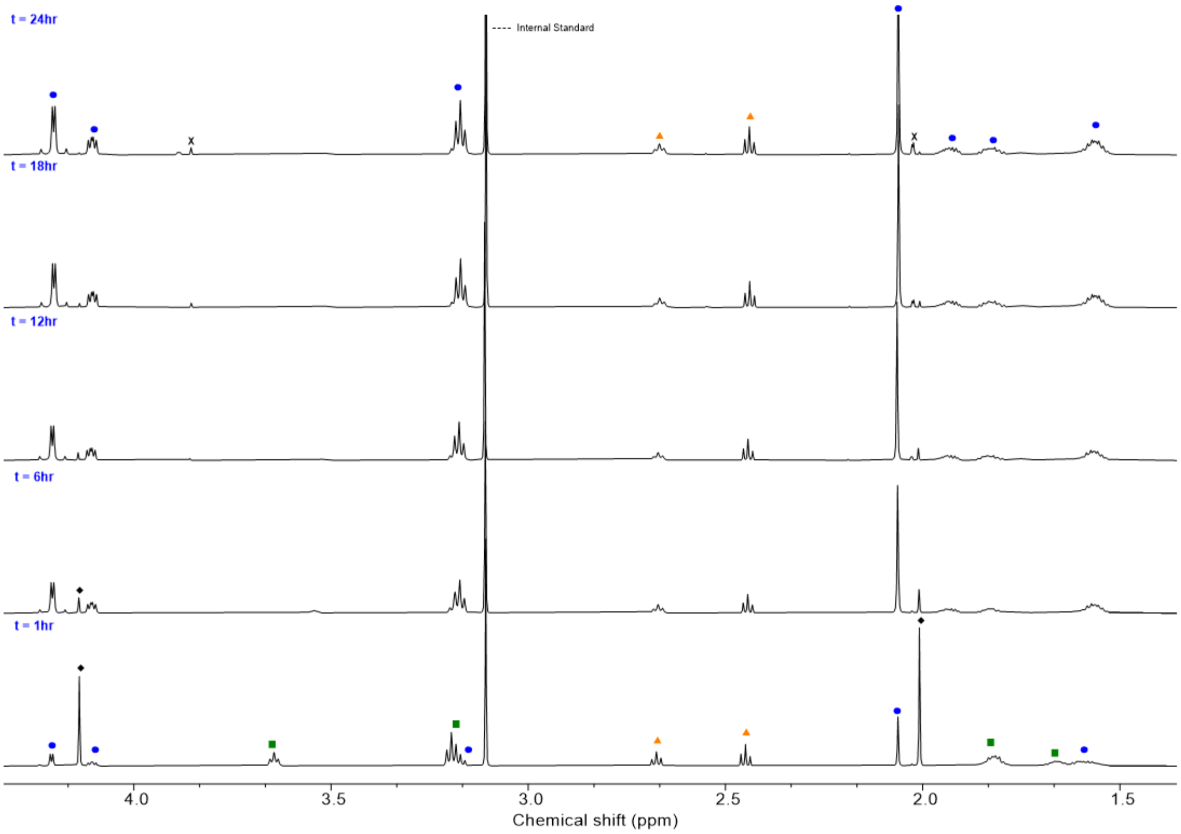

Figure S20: ^{1}H NMR (600 MHz, H_2O/D_2O 9:1, noesygppr1d, 1.0-5.0 ppm) spectrum to show the reaction of N-acetyl glycine nitrile (1, 200 mM) with L-arginine (2$_R$, 240 mM) 3-mercaptopropanoic acid (6b, 60 mM) with (100 mM) as an internal standard, at pH 8.5 and room temperature. ✖ = *N*-acetylglycinamide, 7.

^{1}H NMR (600 MHz, H_2O/D_2O 9:1, noesygppr1d) *(2-acetamido-1-iminoethyl)arginine*, **3**$_R$ (●) (partial assignment): δ_H 4.21 (1H, AB, *J* = 17.1 Hz, AcNHCH*H*), 4.19 (1H, AB, *J* = 17.1 Hz, AcNHCH*H*), 4.11 (1H, dd, *J* = 7.5, 4.7 Hz, Arg-αH-COOH), 3.17 (2H, t, *J* = 6.9 Hz, C*H*$_2$(guanidyl)), 2.06 (3H, s, *H*$_3$C(CO)), 1.94 (1H, m, C*H*HCH$_2$CH$_2$(guanidyl)), 1.83 (1H, m, CH*H*CH$_2$CH$_2$(guanidyl)), 1.57 (2H, m, CH$_2$C*H*$_2$CH$_2$(guanidyl)); *L-arginine*, **2**$_R$ (■) (partial assignment): δ_H 3.50 (1H, t, *J* = 5.6 Hz, αH-COOH), 3.17 (2H, t, *J* = 7.0 Hz, C*H*$_2$(guanidyl)); *N-acetylglycinamide*, **7** (✖) (partial assignment): δ_H 3.88 (2H, s, C*H*$_2$).

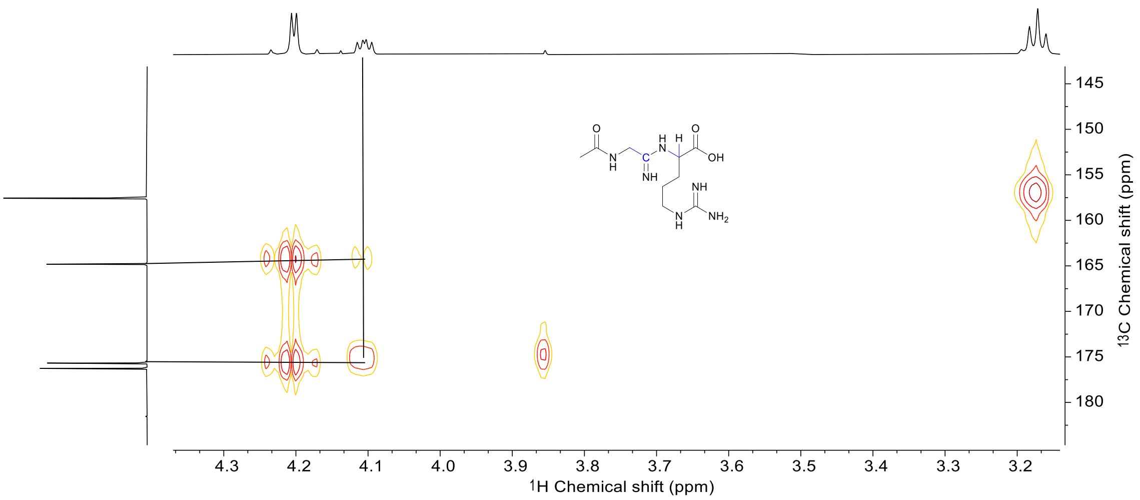

Figure S21: ^1H–^{13}C HMBC (^1H: 600 MHz [3.2-4.3 ppm], ^{13}C: 176 MHz [145-185 ppm], H_2O/D_2O 9:1) spectrum showing the diagnostic $^2J_{CH}$ and $^3J_{CH}$ coupling of **Arg**-αH-COOH in **3R** at 4.11 ppm with two resonances at 176.2 and 164.7 ppm, which is characteristic of amidine bond formation of **2R**.

*Coupling of **1** with L-asparagine **2**$_N$ at pH 8.5 and room temperature*

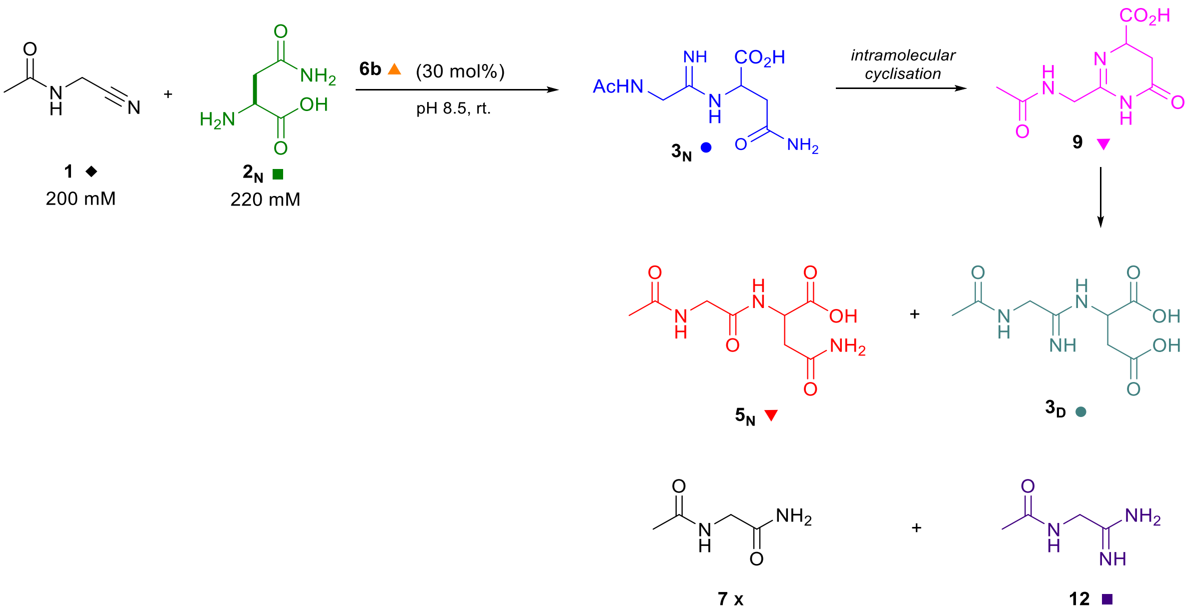

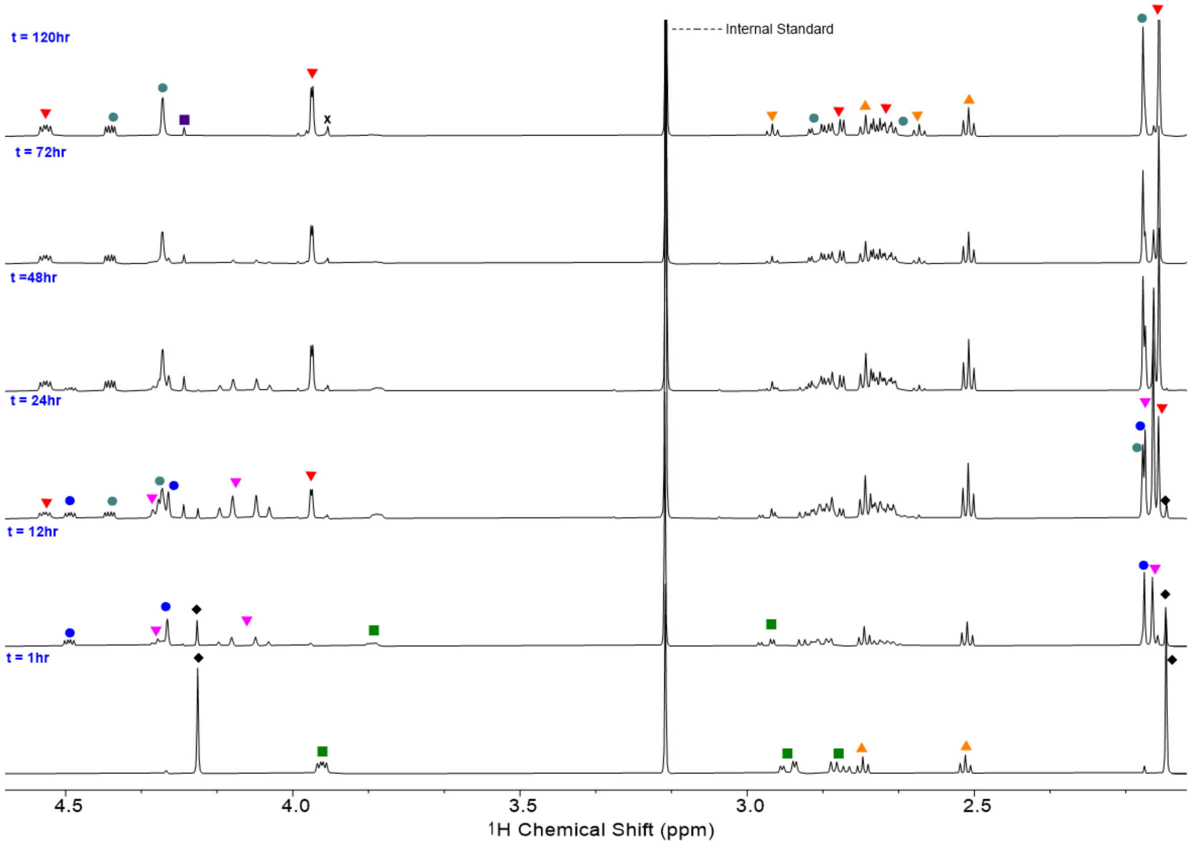

Figure S22: ^{1}H NMR (600 MHz, H_2O/D_2O 9:1, noesygppr1d, 1.0–5.0 ppm) spectrum to show the reaction of *N*-acetyl glycine nitrile (**1**, 200 mM) with L-asparagine (**2**$_N$, 220 mM) and 3-mercaptopropanoic acid (MPA, 60 mM) with MSM (100 mM) as an internal standard at pH 8.5 and room temperature. ✖= 7 ■= 12.

Partial assignment for dihydropyrimidone **9** (▼): δ_H 4.30 (1H, m, Asn-αH-COOH), 4.15 (1H, AB, J = 17.2 Hz, AcNHCH*H*), 4.07 (1H, AB, J = 17.2 Hz, AcNHC*H*H), 2.11 (3H, s, H_3C(CO)).

^{1}H NMR (600 MHz, H_2O/D_2O 9:1, noesygppr1d) *N-Acetylglycylasparagine*, **5_N** (▼): δ_H 4.54 (1H, dd, *J* = 8.5, 4.8 Hz, Asn-αH-COOH), 3.94 (1H, AB, *J* = 17.1 Hz, AcNHCH*H*), 3.91 (1H, AB, *J* = 17.1 Hz, AcNHCH*H*), 2.80 (1H, ABX, *J* = 15.2, 4.8 Hz, CH(C*H*HCONH$_2$)COOH), 2.72 - 2.77 (1H, m, CH(CH*H*CONH$_2$)COOH), 2.09 (3H, s, *H*$_3$C(CO)); *(2-acetamido-1-iminoethyl)aspartic acid*, **2_N** (●): δ_H 4.40 (1H, dd, *J* = 8.1, 3.8 Hz, Asp-αH-COOH)), 4.29 (1H, AB, *J* = 17.2 Hz, AcNHCH*H*), 4.24 (1H, AB, *J* = 17.2 Hz, AcNHCH*H*), 2.85 (1H, ABX, *J* = 16.7, 3.8 Hz, CH(CH*H*COOH)COOH), 2.71-2.67 (1H, m, CH(CH*H*COOH)COOH), 2.13 (3H, s, *H*$_3$C(CO)); *L-asparagine*, **2_N** (■) (partial assignment): δ_H 3.82 (1H, dd, *J* = 8.3, 4.6 Hz, αH-COOH); *N-acetylglycinamide*, **7** (✖):δ_H 3.92 (2H, s, C*H*$_2$). **12** (■) (partial assignment): δ_H 4.24 (2H, s, CH$_2$). Dihydropyrimidone **9** (▼): HRMS (ESI) m/z for $[C_8H_{12}N_3O_4]^+$: calcd 214.0822, found 214.0822.

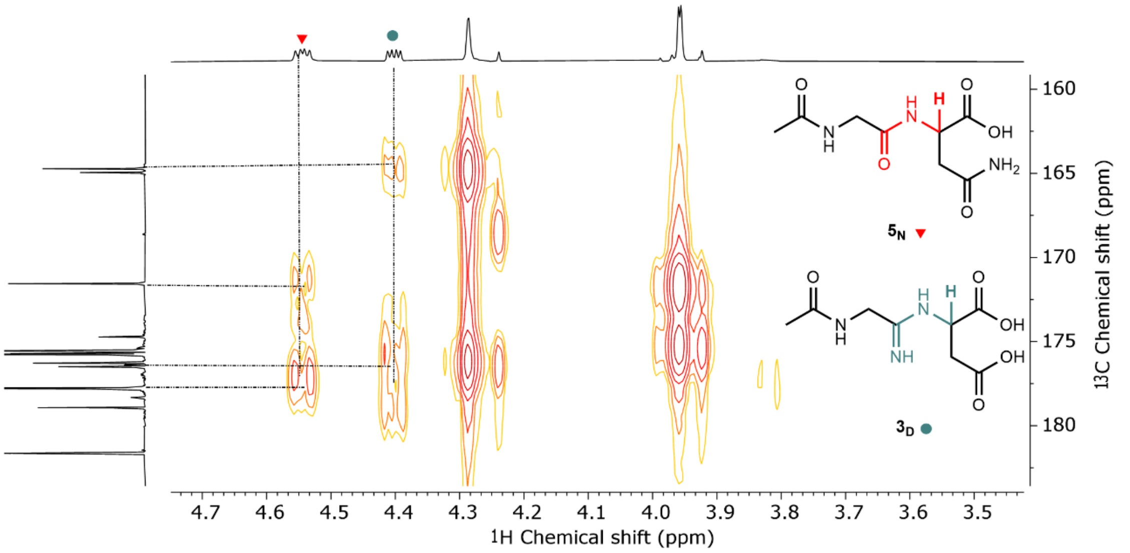

Figure S23: ^{1}H–^{13}C HMBC (^{1}H: 600 MHz [3.5-4.5 ppm], ^{13}C: 176 MHz [150-185 ppm], H_2O/D_2O 9:1) spectrum showing the diagnostic $^{2}J_{CH}$ and $^{3}J_{CH}$ coupling of the Asn-αH-COOH in 5_N at 4.54 ppm with two resonances at 177.9 and 171.5 ppm, which is characteristic of peptide bond formation of 2_N and the $^{2}J_{CH}$ and $^{3}J_{CH}$ coupling of Asp-αH-COOH in 3_D at 4.40 ppm with two resonances at 175.7 and 164.7 ppm, which is characteristic of peptide bond formation amidine of 2_D.

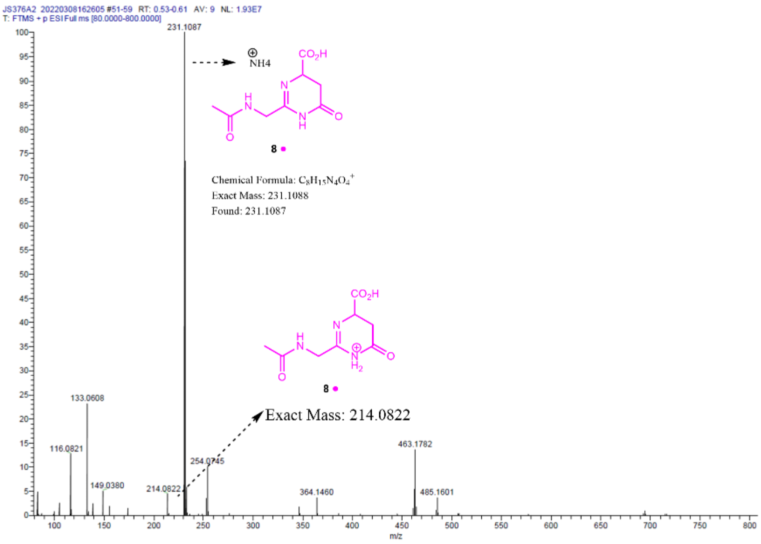

Figure S24: *HRMS (ESI) spectra for dihydropyrimidone intermediate 9.*

*Coupling of **1** with L-glutamic acid **2**$_E$ at pH 8.5 and room temperature*

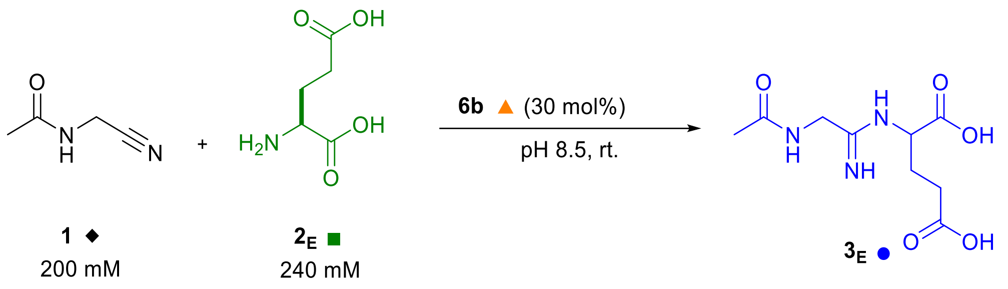

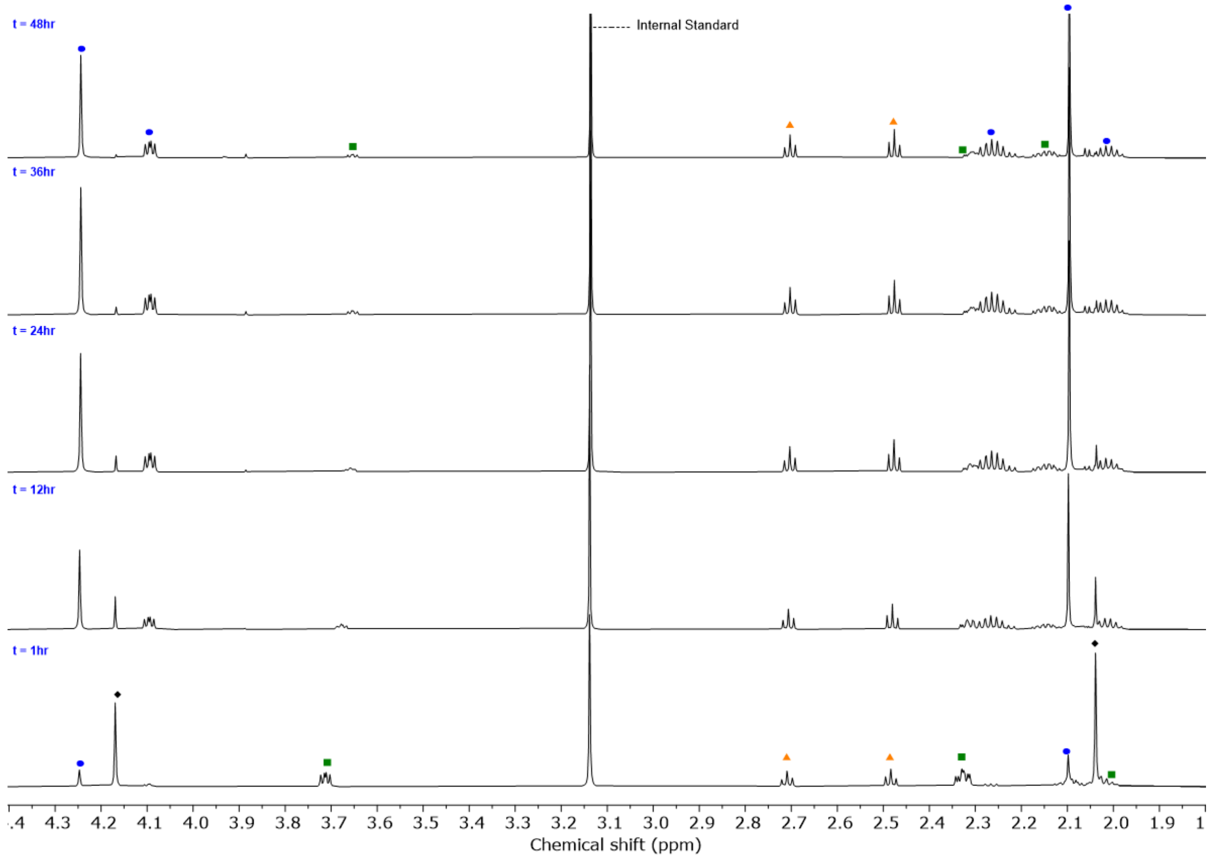

Figure S25: ^{1}H NMR (600 MHz, H_2O/D_2O 9:1, noesygppr1d, 1.8-4.4 ppm) spectrum to show the reaction of N-acetyl glycine nitrile (**1**, 200 mM) with L-glutamic acid (**2**$_E$, 240 mM) and 3-mercaptopropanoic acid (**6b**, 60 mM) with MSM (100 mM) as an internal standard at pH 8.5 and room temperature.

^{1}H NMR (600 MHz, H_2O/D_2O 9:1) *(2-acetamido-1-iminoethyl)glutamic acid*, **3**$_E$ (●): δ_H 4.24 (2H, s, AcNHC*H*$_2$), 4.09 (1H, dd, *J* = 7.5, 4.9 Hz, Glu-αH-COOH), 2.13-2.25 (3H, m, CH_2CH_2COOH), 2.10 (3H, s, *H*$_3$C(CO)), 2.06-1.97 (1H, m, CH_2CH_2COOH); *L-glutamic acid*, **2**$_E$ (■): δ_H 3.60 (1H, dd, *J* = 7.2, 5.2 Hz, αH-COOH), 2.31-2.22 (4H, m, CH_2CH_2COOH); *N-acetylglycinamide*, **7** (✖) (partial assignment): δ_H 3.93 (2H, d, *J* = 2.7 Hz, C*H*$_2$).

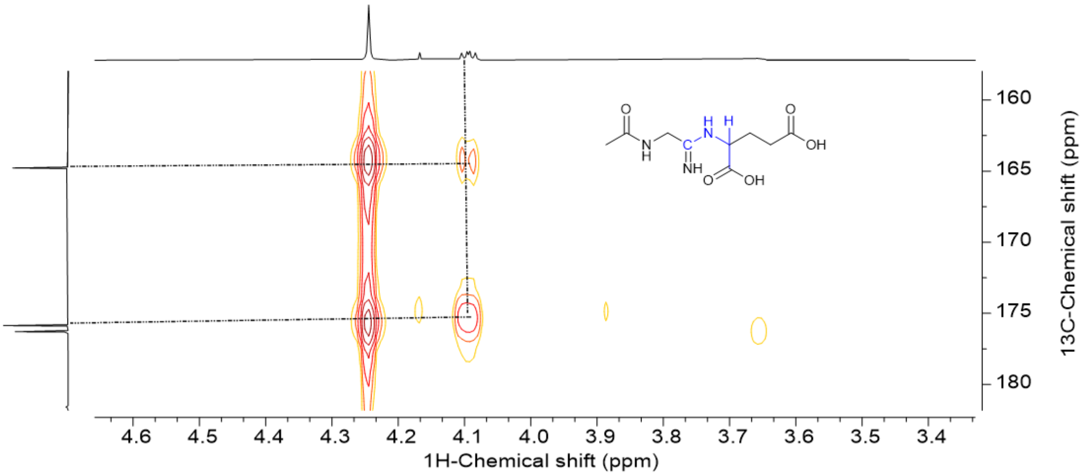

Figure S26: ^{1}H–^{13}C HMBC (^{1}H: 600 MHz [3.5-4.5 ppm], ^{13}C: 176 MHz [150-185 ppm], H_2O/D_2O 9:1) spectrum showing the diagnostic $^{2}J_{CH}$ and $^{3}J_{CH}$ coupling of the Glu-α***H***-COOH in 3_E at 4.09 ppm with two resonances at 175.8 and 164.9 ppm, which is characteristic of amidine bond formation of $\mathbf{2_E}$.

*Coupling of **1** with L-glutamine **2$_Q$** at pH 8.5 and room temperature*

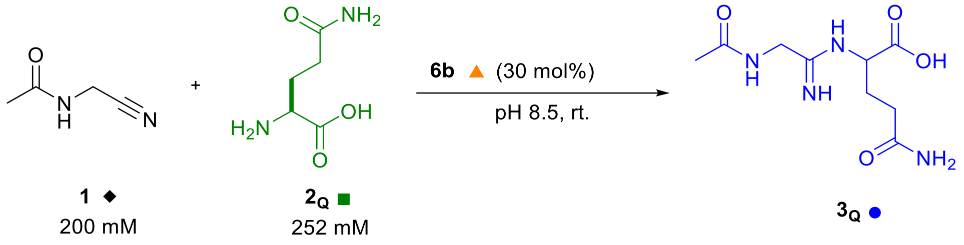

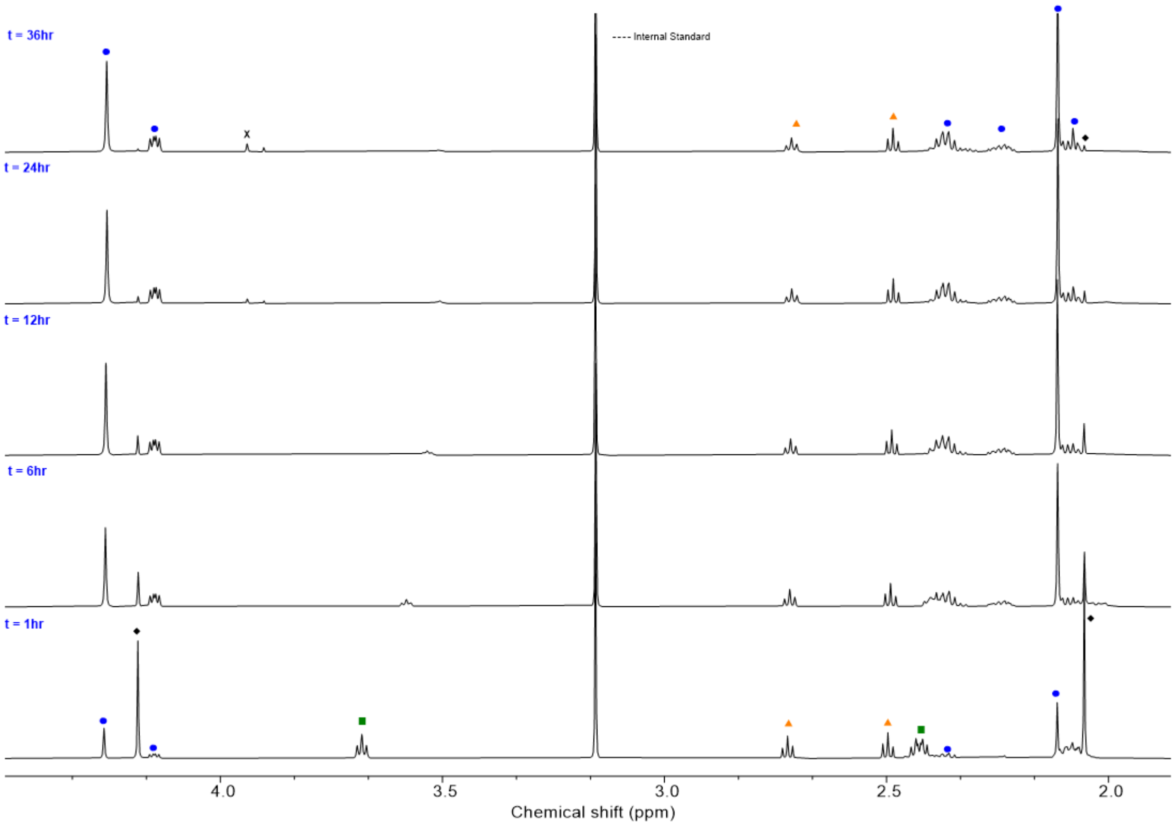

Figure S27: ^{1}H NMR (600 MHz, H_2O/D_2O 9:1, noesygppr1d, 1.8-4.5 ppm) spectrum to show the reaction of N-acetyl glycine nitrile (**1**, 200 mM) with L-glutamine (**2$_Q$**, 250 mM) and 3-mercaptopropanoic acid (**6b**, 60 mM) with MSM (100 mM) as an internal standard at pH 8.5 and room temperature.

^{1}H NMR (600 MHz, H_2O/D_2O 9:1, noesygppr1d) *(2-acetamido-1-iminoethyl)glutamine*, **3$_Q$** (●): δ_H 4.26 (2H, s, AcNHC*H*$_2$), 4.15 (1H, dd, *J* = 7.7, 4.7 Hz, Gln-α*H*-COOH), 2.40 - 2.33 (2H, m, $CH_2CH_2CONH_2$), 2.30 - 2.20 (1H, m, C*H*HCH$_2$CONH$_2$), 2.08 - 2.05 (1H, m, C*H*HCH$_2$CONH$_2$), 2.09 (3H, s, *H*$_3$C(CO)); *L-glutamine*, **2$_Q$** (■): δ_H 3.44 (1H, t, *J* = 6.3 Hz, αH-COOH), 2.25-2.19 (2H, m, C*H*$_2$CH$_2$CONH$_2$), 2.01-1.89 (2H, m, CH$_2$C*H*$_2$CONH$_2$); *N-acetylglycinamide*, **7** (✖) (partial assignment): δ_H 3.92 (2H, s, C*H*$_2$).

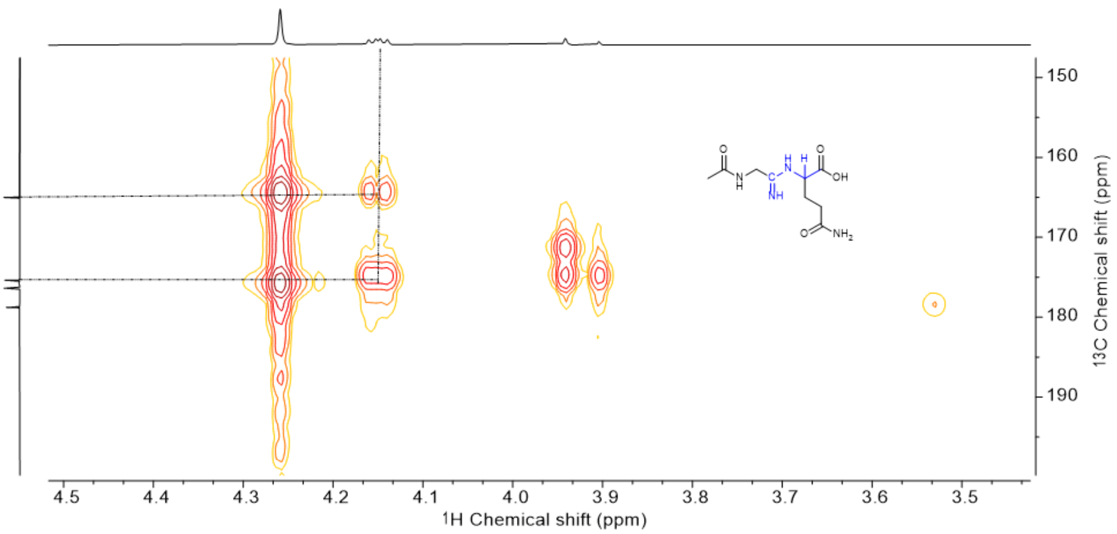

Figure S28: ^{1}H–^{13}C HMBC (^{1}H: 600 MHz [3.5-4.5 ppm], ^{13}C: 176 MHz [150-200 ppm], H_2O/D_2O 9:1) spectrum showing the diagnostic $^2J_{CH}$ and $^3J_{CH}$ coupling of the **Gln**-αH-COOH in $\mathbf{3_Q}$ at 4.14 ppm with two resonances at 175.4 and 164.2 ppm, which is characteristic of amidine bond formation of $\mathbf{2_Q}$.

*Coupling of **1** with glycine **2**$_G$ at pH 8.5 and room temperature*

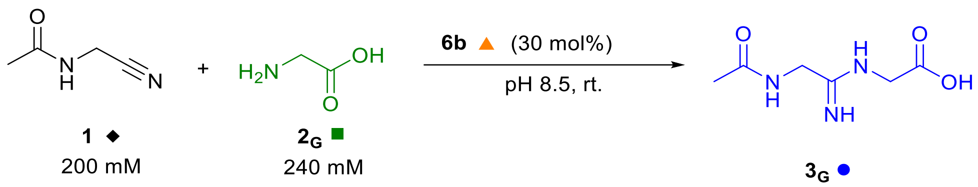

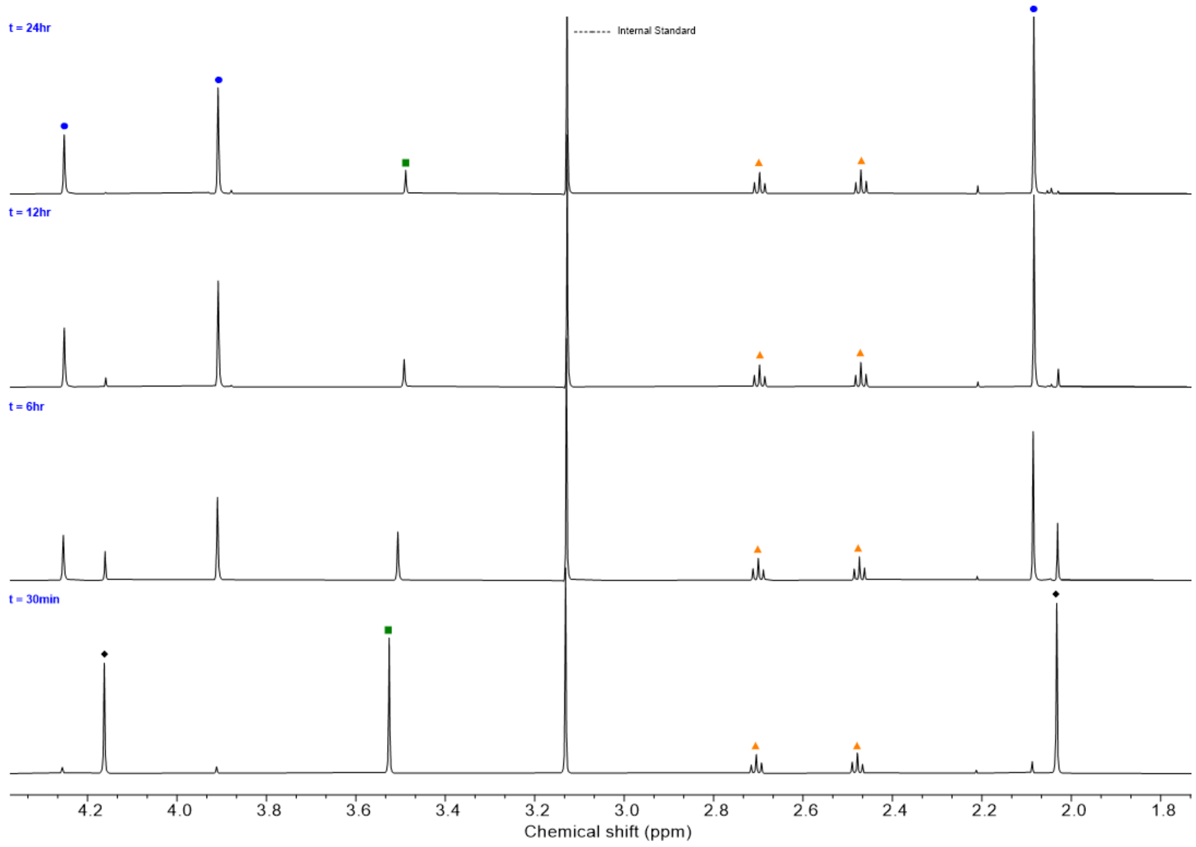

Figure S29: ^{1}H NMR (600 MHz, H_2O/D_2O 9:1, noesygppr1d, 1.7-4.3 ppm) spectrum to show the reaction of N-acetyl glycine nitrile (**1**, 200 mM) with glycine (**2**$_G$, 240 mM) and 3-mercaptopropanoic acid (**6b**, 60 mM) with MSM (100 mM) as an internal standard at pH 8.5 and room temperature.

^{1}H NMR (600 MHz, H_2O/D_2O) *(2-acetamido-1-iminoethyl)glycine*, **3**$_G$ (●): δ_H 4.25 (2H, s, AcNHC*H*$_2$), 3.91 (2H, s, C*H*$_2$COOH), 2.08 (3H, s, *H*$_3$C(CO)); *glycine*, **2**$_G$ (■): δ_H 3.49 (2H, s, C*H*$_2$).

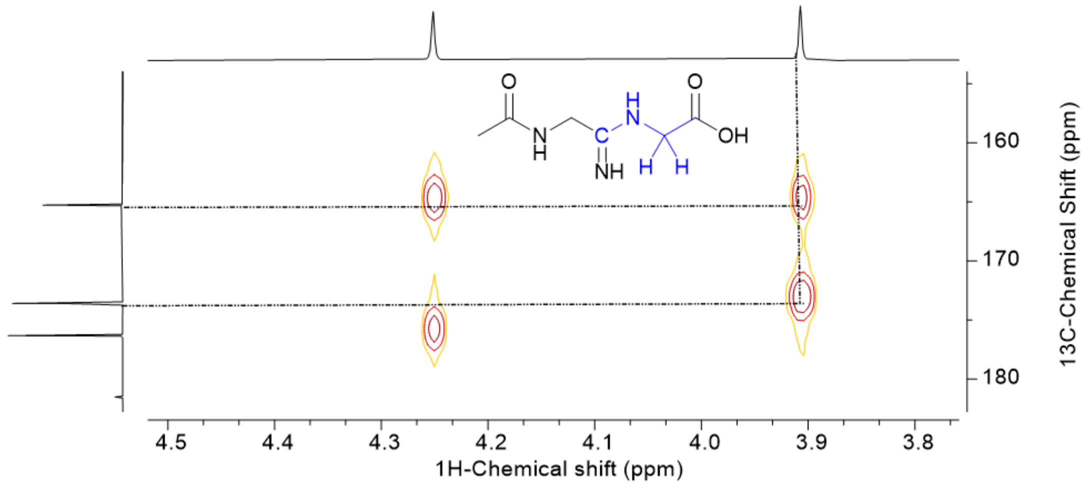

Figure S30: ${}^1H$–${}^{13}C$ HMBC (${}^1H$: 600 MHz [3.80–4.50 ppm], ${}^{13}C$: 176 MHz [150-185 ppm], H_2O/D_2O 9:1) spectrum showing the diagnostic ${}^2J_{CH}$ and ${}^3J_{CH}$ coupling of **Gly**-αH-COOH in $\mathbf{3_G}$ at 3.91 ppm with two resonances at 175.4 and 164.6 ppm, which is characteristic of amidine bond formation of **Gly**.

*Coupling of **1** with L-histidine **2_H** at pH 8.5 and room temperature*

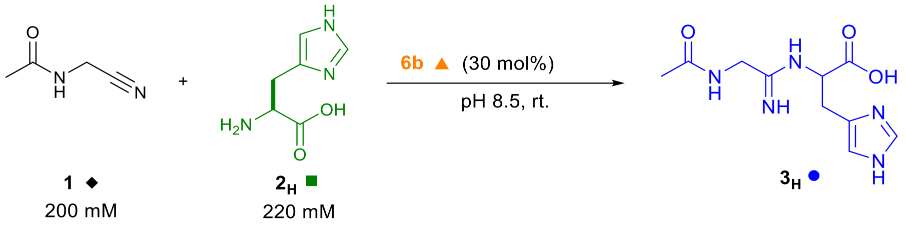

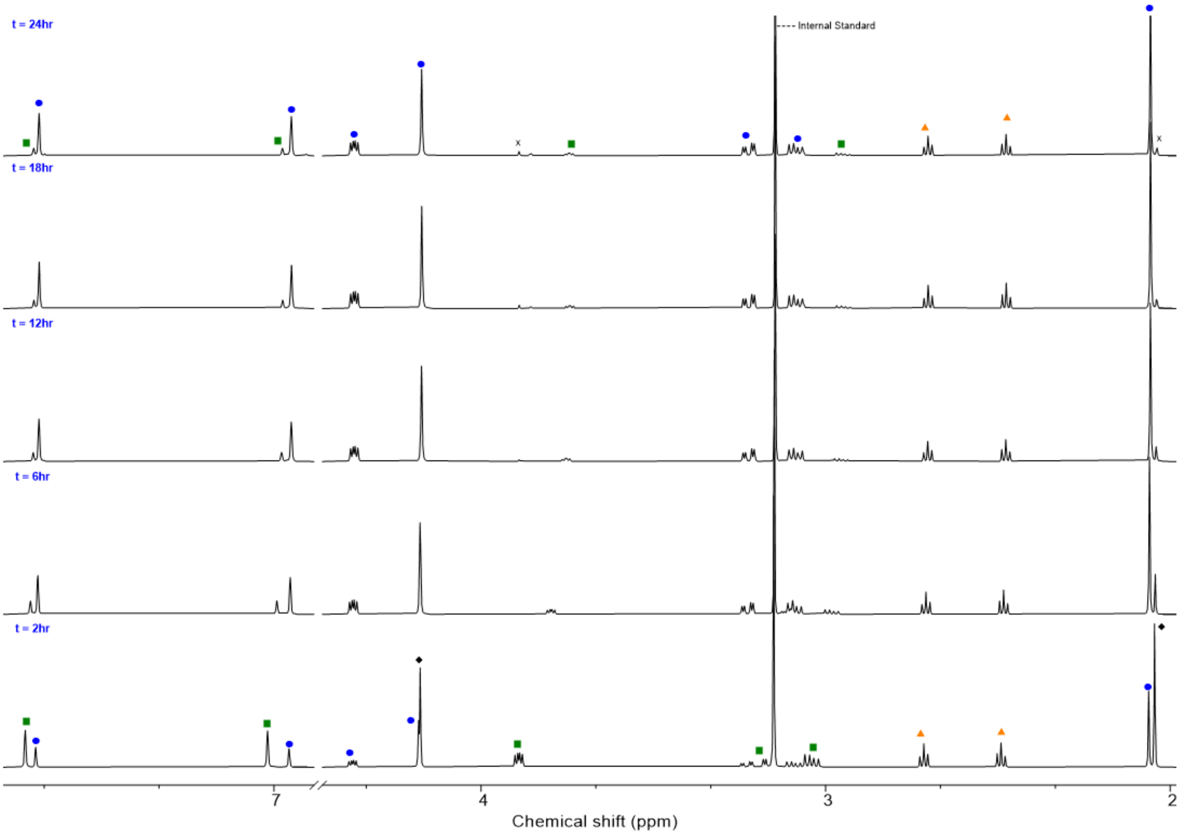

Figure S31: ^{1}H NMR (600 MHz, H_2O/D_2O 9:1, noesygppr1d, 2.0-5.0 ppm) spectrum to show the reaction of N-acetyl glycine nitrile (**1**, 200 mM) with L-histidine (**2_H**, 220 mM) and 3-mercaptopropanoic acid (**6b**, 60 mM) with MSM (100 mM) as an internal standard at pH 8.5 and room temperature.

^{1}H NMR (600 MHz, H_2O/D_2O 9:1, noesygppr1d) *(2-acetamido-1-iminoethyl)histidine*, **3_H** (●): δ_H 7.64 (1H, s, Ar*H*), 6.91 (1H, s, Ar*H*), 4.33 (1H, dd, *J* = 8.1, 4.5 Hz, His-αH-COOH), 4.17 (2H, s, AcNHC*H*2), 3.22 (1H, ABX, *J* = 15.1, 4.5 Hz, CH(CH*H*Ar)), 3.09 (1H, ABX, *J* = 15.1, 8.1 Hz, CH(CH*H*Ar)), 2.06 (3H, s, *H*3C(CO)); *L-histidine*, **2_H** (■): δ_H 7.70 (1H, s, Ar*H*), 6.98 (1H, s, Ar*H*), 3.74 (1H, ABX, *J* = 8.0, 4.9 Hz, αH-COOH), 2.91-2.87 (1H, m, CH(CH*H*Ar)), 2.95 (1H, dd, *J* = 15.1, 8.0 Hz, CH(CH*H*Ar)); *N-acetylglycinamide*, **7** (✖) (partial assignment): δ_H 3.89 (2H, s, C*H*2).

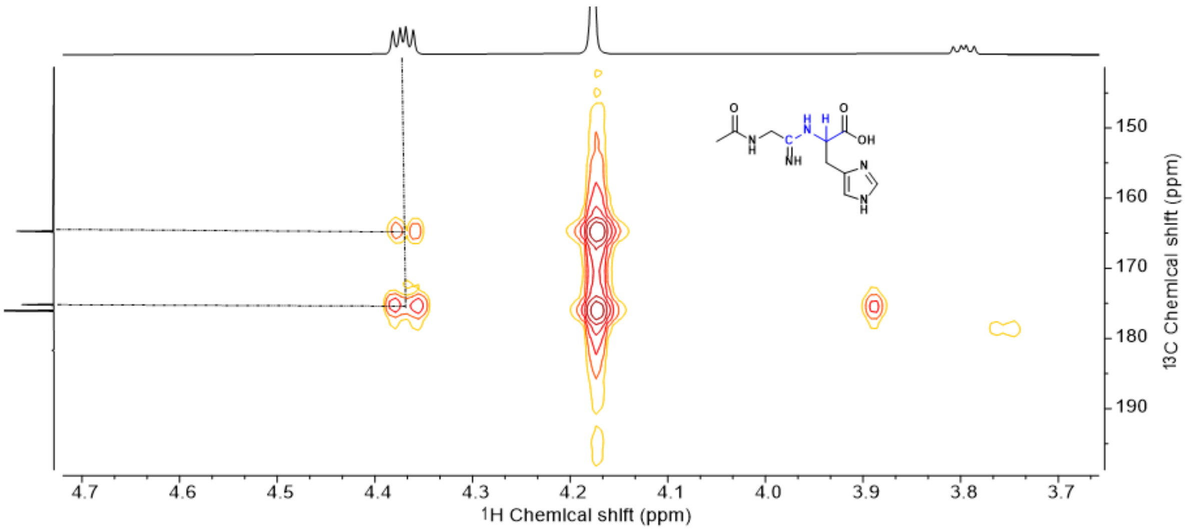

Figure S32: ^1H–^{13}C HMBC (^1H: 600 MHz [3.5–4.5 ppm], ^{13}C: 176 MHz [110–185 ppm], H_2O/D_2O 9:1) spectrum showing the diagnostic $^2J_{CH}$ and $^3J_{CH}$ coupling of the **His**-αH-COOH in $\mathbf{3_H}$ at 4.33 ppm with two resonances at 175.2 and 164.7 ppm, which is characteristic of amidine bond formation of $\mathbf{2_H}$.

*Coupling of **1** with L-isoleucine **2_I** at pH 8.5 and room temperature*

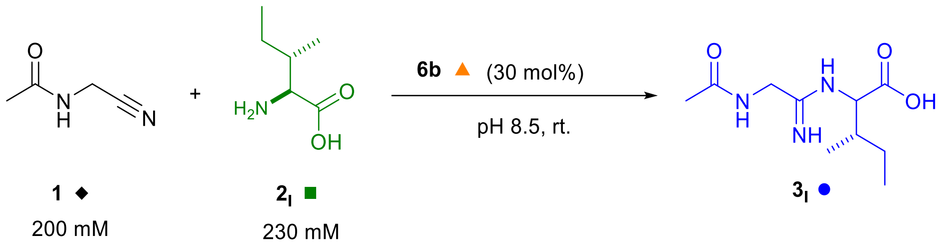

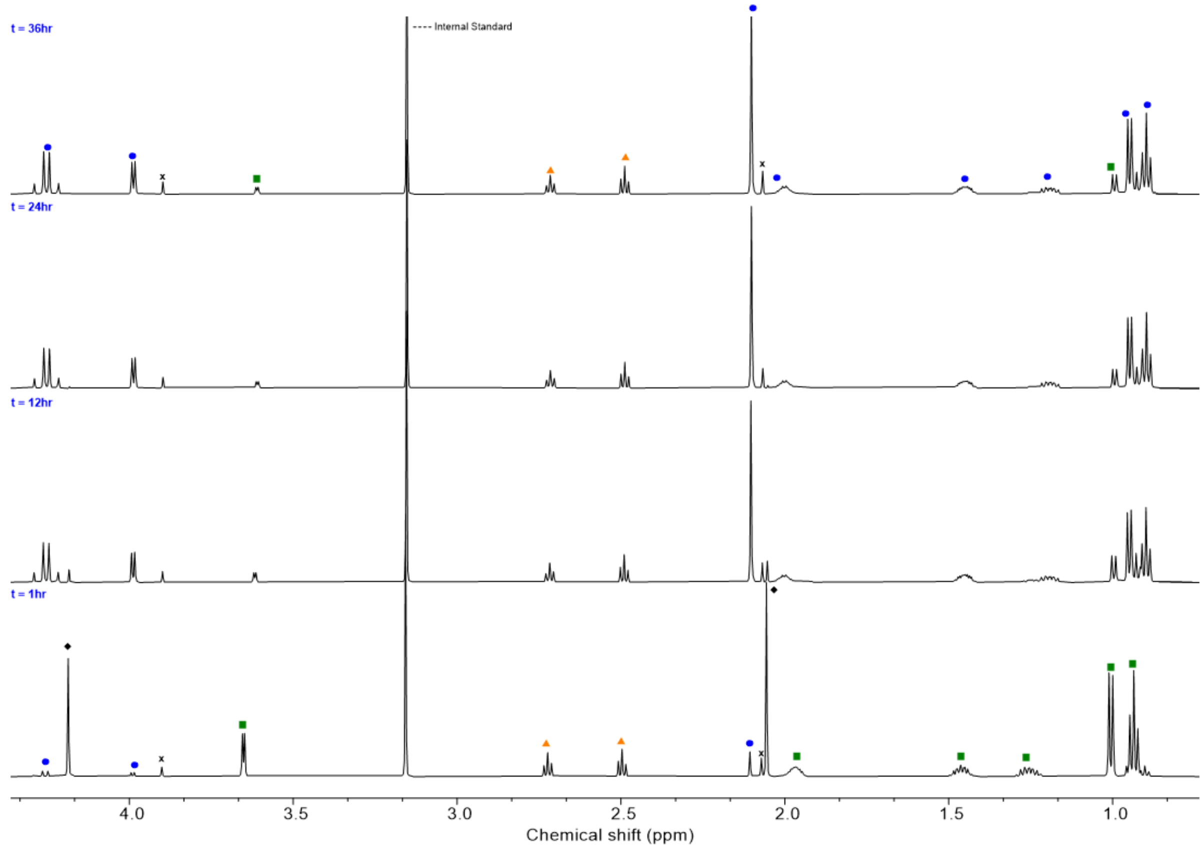

Figure S33: ^{1}H NMR (600 MHz, H_2O/D_2O 9:1, noesygppr1d, 0.5–4.2 ppm) spectrum to show the reaction of N-acetyl glycine nitrile (**1**, 200 mM) with L-isoleucine (**2**$_I$, 230 mM) and 3-mercaptopropanoic acid (**6b**, 60 mM) with MSM (100 mM) as an internal standard at pH 8.5 and room temperature.

^{1}H NMR (600 MHz, H_2O/D_2O 9:1, noesygppr1d) *(2-acetamido-1-iminoethyl)isoleucine*, **3**$_I$ (●) (partial assignment): δ_H 4.28 (1H, AB, *J* = 17.1 Hz, AcNHCH*H*), 4.23 (1H, AB, *J* = 17.1 Hz, AcNHC*H*H), 3.99 (1H, d, *J* = 5.8 Hz, Ile-αH-COOH), 2.08 (3H, s, *H*$_3$C(CO)), 2.04-1.97 (m, 1H, Ile-βC*H*(CH$_3$)CH$_2$CH$_3$), 1.50-1.40 (m, 1H, Ile-CH(CH$_3$)C*H*HCH$_3$), 1.26-1.14 (m, 1H, Ile-CH(CH$_3$)CH*H*CH$_3$), 0.95 (3H, d, *J = 6.9 Hz*, Ile-βCH(C*H*$_3$)CH$_2$CH$_3$), 0.90 (3H, t, *J = 7.4 Hz*, Ile-CH(CH$_3$)CH$_2$C*H*$_3$); *L-isoleucine*, **2**$_I$ (■) (partial assignment): δ_H 3.61 (1H, d, *J* = 4.1 Hz, αH-COOH); *N-acetylglycinamide*, **7** (✖) (partial assignment): δ_H 3.90 (2H, s, C*H*$_2$).

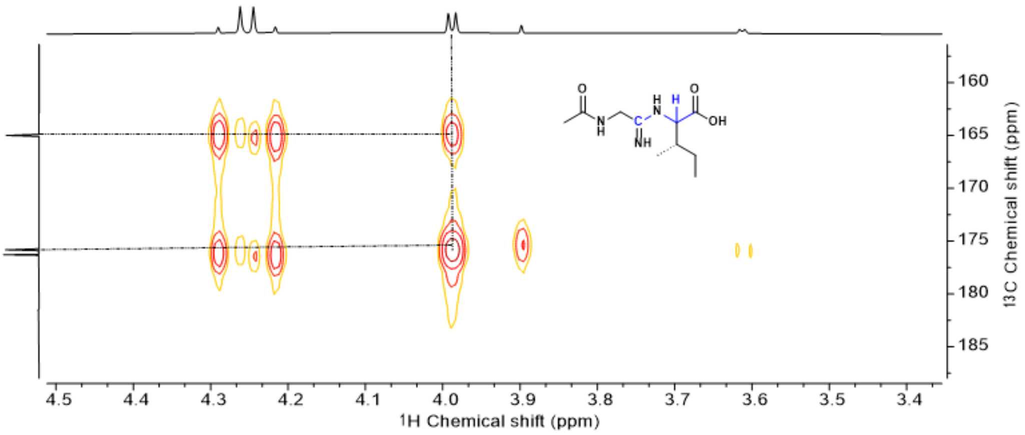

Figure S34: ^{1}H–^{13}C HMBC (^{1}H: 600 MHz [3.4–4.5 ppm], 13C: 176 MHz [150–185 ppm], H_2O/D_2O 9:1) spectrum showing the diagnostic $^{2}J_{CH}$ and $^{3}J_{CH}$ coupling of the **Ile**-αH-COOH in **3I** at 4.25 ppm with two resonances at 175.7 and 164.7 ppm, which is characteristic of amidine bond formation of **2I**.

*Coupling of **1** with L-leucine **2**$_L$ at pH 8.5 and room temperature*

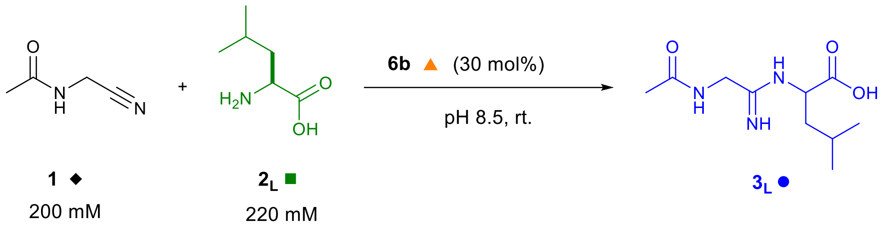

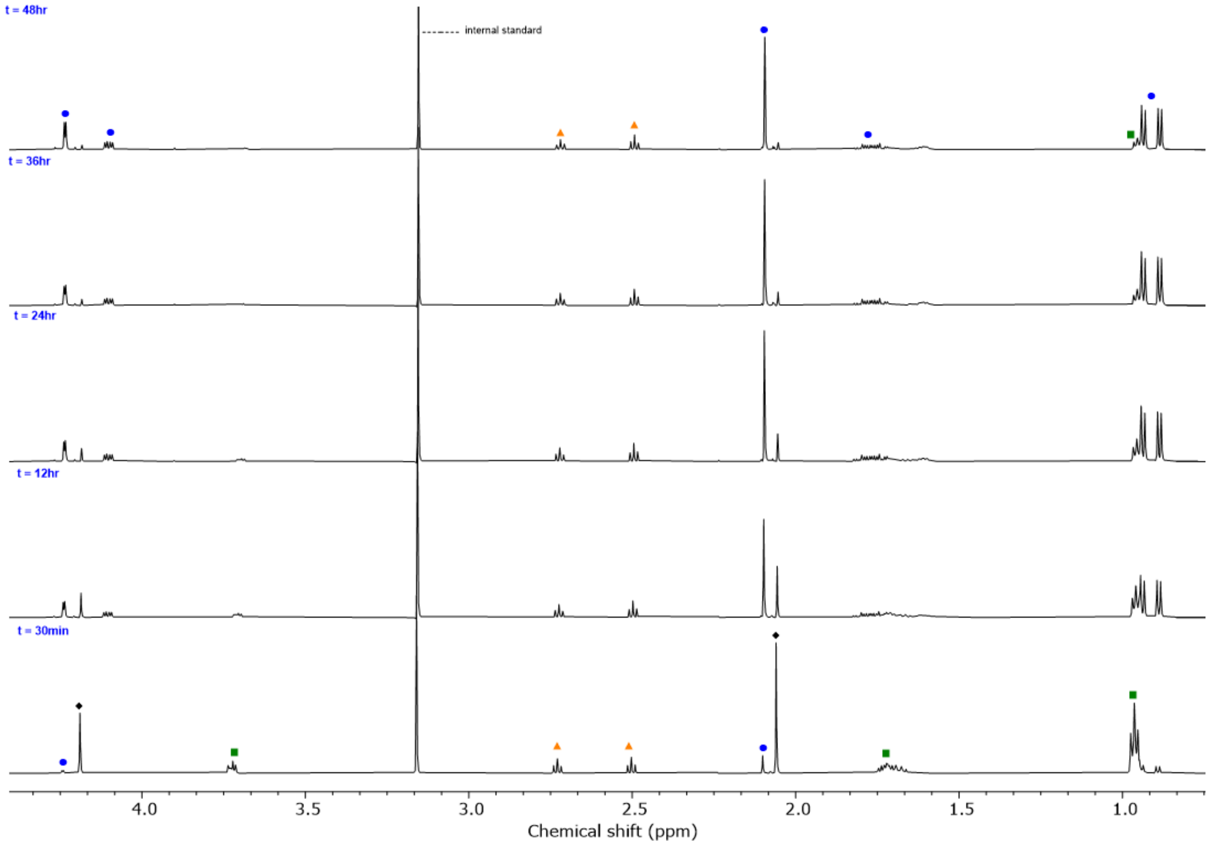

Figure S35: ^{1}H NMR (600 MHz, H_2O/D_2O 9:1, noesygppr1d, 0.5-5.0 ppm) spectrum to show the reaction of N-acetyl glycine nitrile (**1**, 200 mM) with L-leucine (**2**$_L$, 220 mM) and 3-mercaptopropanoic acid (**6b**, 60 mM) with MSM (100 mM) as an internal standard at pH 8.5 and room temperature. ✖ = 7.

^{1}H NMR (600 MHz, H_2O/D_2O 9:1, noesygppr1d) *(2-acetamido-1-iminoethyl)leucine*, **3**$_L$ (●) (partial assignment): δ_H 4.24 (1H, AB, *J* = 17.1 Hz, AcNHCH*H*), 4.20 (1H, AB, *J* = 17.1 Hz, AcNHC*H*H), 4.10 (1H, dd, *J* = 9.8, 4.3 Hz, Leu-αH-COOH), 2.07 (3H, s, *H*$_3$C(CO)), 1.69-1.84 (2H, m; Leu-C*H*$_2$), 1.58-1.68 (1H, m; Leu-C*H*-(CH$_3$)$_2$), 0.94 (3H, d, *J* = 6.7 Hz, Leu-(C*H*$_3$)), 0.89 (3H, d, *J* = 6.5 Hz, Leu-(C*H*$_3$)) *L-leucine*, **2**$_L$ (■) (partial assignment): δ_H 3.62 (1H, dd, *J* = 8.8, 4.9 Hz, αH-COOH); *N-Acetylglycinamide*, **7** (✖) (partial assignment): δ_H 3.89 (2H, s, C*H*$_2$).

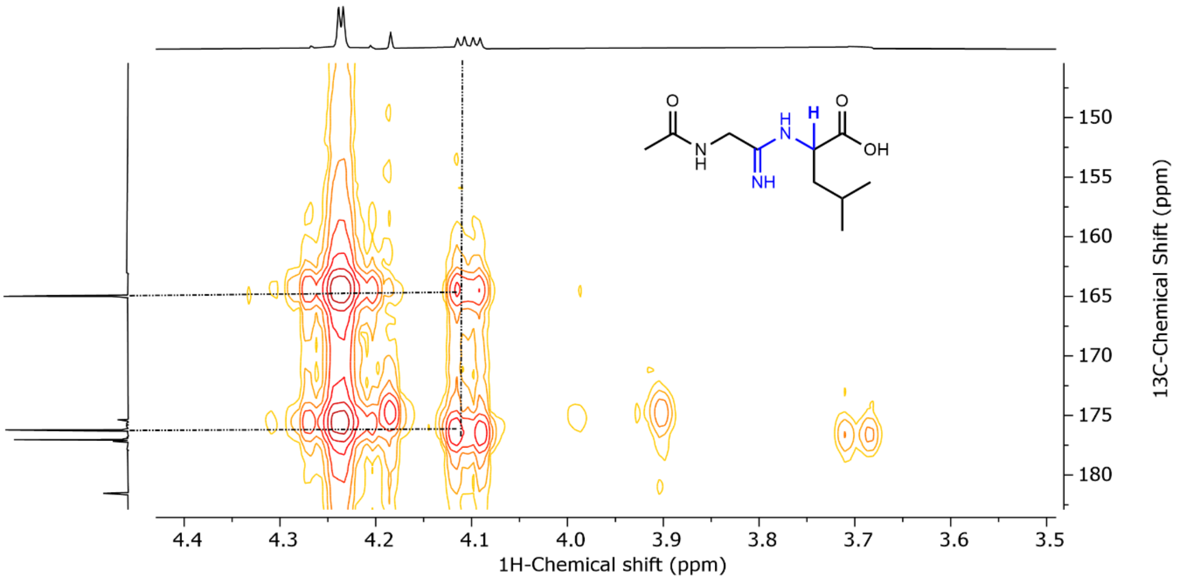

Figure S36: ^{1}H–^{13}C HMBC (^{1}H: 600 MHz [3.5-4.5 ppm], ^{13}C: 176 MHz [150-185 ppm], H_2O/D_2O 9:1) spectrum showing the diagnostic $^2J_{CH}$ and $^3J_{CH}$ coupling of the **Leu**-αH-COOH in **3**$_L$ at 4.08 ppm with two resonances at 175.6 and 164.6 ppm, which is characteristic of amidine bond formation of **2**$_L$.

*Coupling of **1** with L-lysine **2**$_K$ at pH 8.5 and room temperature*

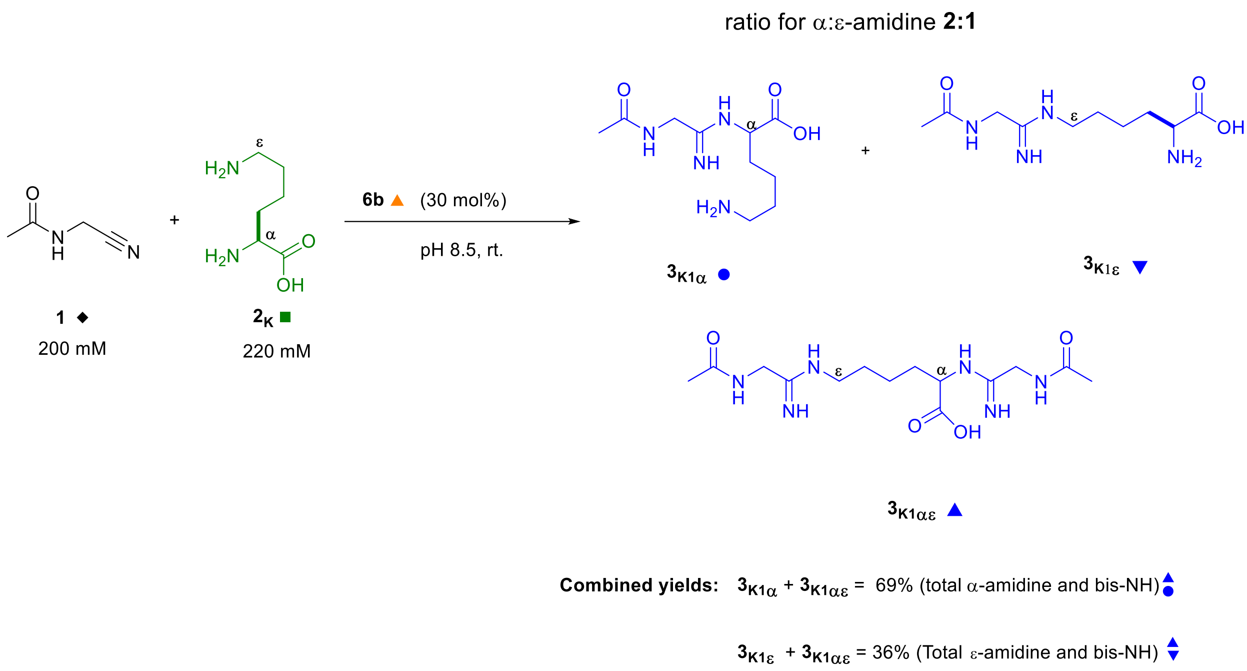

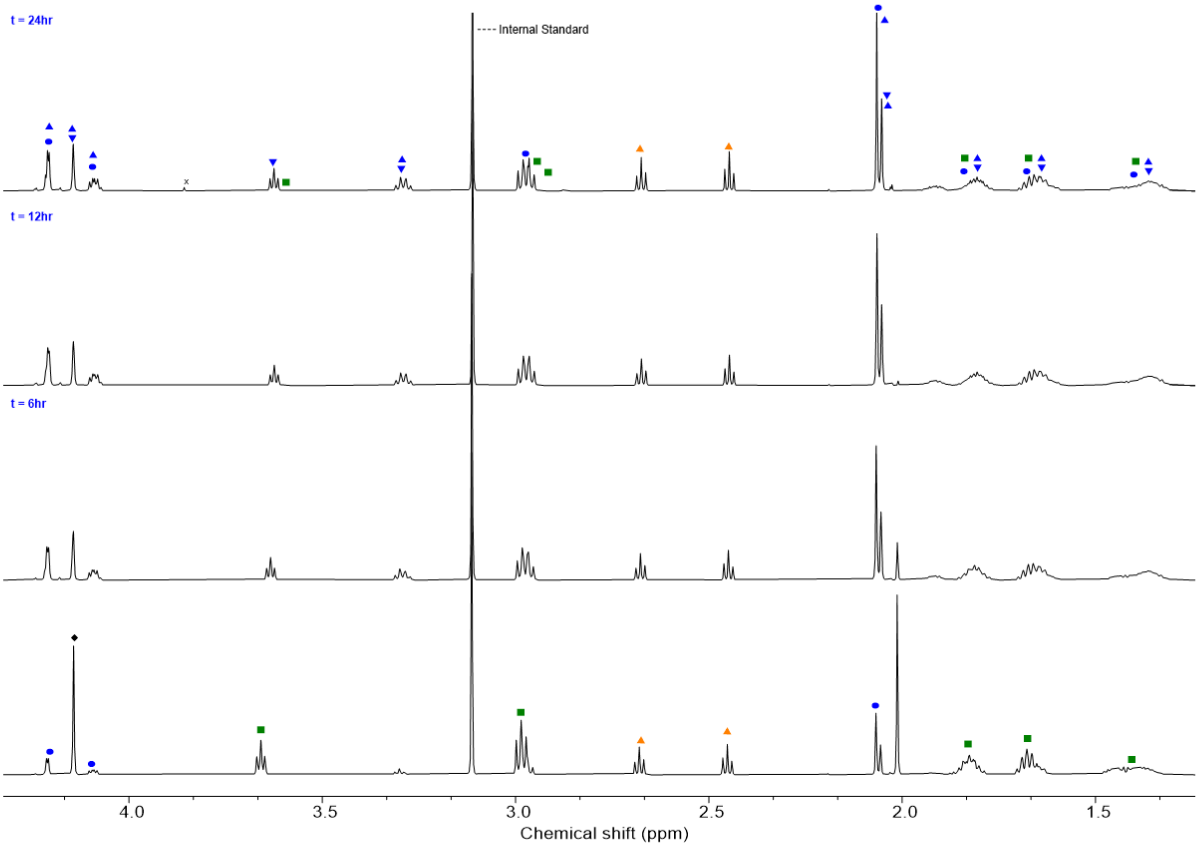

Figure S37: ^{1}H NMR (600 MHz, H_2O/D_2O 9:1, noesygppr1d, 0.5-5.0 ppm) spectrum to show the reaction of N-acetyl glycine nitrile (**1**, 200 mM) with L-lysine (**2**$_K$, 250 mM) and 3-mercaptopropanoic acid (**6b**, 60 mM) with MSM (100 mM) as an internal standard at pH 8.5 and room temperature.

^{1}H NMR (600 MHz, H_2O/D_2O 9:1, noesygppr1d):

N²-(2-acetamido-1-iminoethyl)lysine, ***3$_{K1\alpha}$*** (●) (partial assignment): δ_H 4.21 – 4.17 (2H, m, dd, overlapped, *N*²-AcNHC*HH*), 4.09 (1H, dd, *J* = 7.5, 4.7 Hz, Lys-αH-COOH), 2.93 – 3.01 (2H, m, *N*⁶-NH₂C*H*₂CH₂), 2.07 (3H, s, *N*²-*H*₃C(CO)).

N²,N⁶*-di(2-acetamido-1-iminoethyl)lysine*, ***3$_{K1\alpha\varepsilon}$*** (▲) (partial assignment): δ_H 4.25 – 4.14 (2H, m, *N*²-AcNHC*HH*), 4.11-4.07 (1H, m, overlapped, Lys-αH-COOH), 3.37 – 3.23 (2H, m, *N*⁶-C*H*₂CH₂), 2.07 (3H, s, *N*²-*H*₃C(CO)), 2.03 (3H, s, *N*⁶-*H*₃C(CO)).

N⁶*-(2-acetamido-1-iminoethyl)lysine*, ***3$_{K1\varepsilon}$***(▼) (partial assignment): δ_H 4.14 (2H, s, *N*⁶-AcNHC*H*₂), 3.63 (1H, t, *J* = 6.1 Hz, Lys-αH-COOH), 3.37-3.23 (2H, m, *N*⁶-C*H*₂CH₂), 2.03 (3H, s, *N*⁶-*H*₃C(CO)); *L-lysine*, **Lys** (■) (partial assignment): δ_H 3.63 (1H, t, *J* = 6.1 Hz, Lys-αH-COOH), 2.95 – 3.01 (2H, t, *J* = 7.9 Hz, *N*⁶-NH₂C*H*₂CH₂); *N-Acetylglycinamide*, **7** (✖) (partial assignment): δ_H 3.86 (2H, s, C*H*₂).

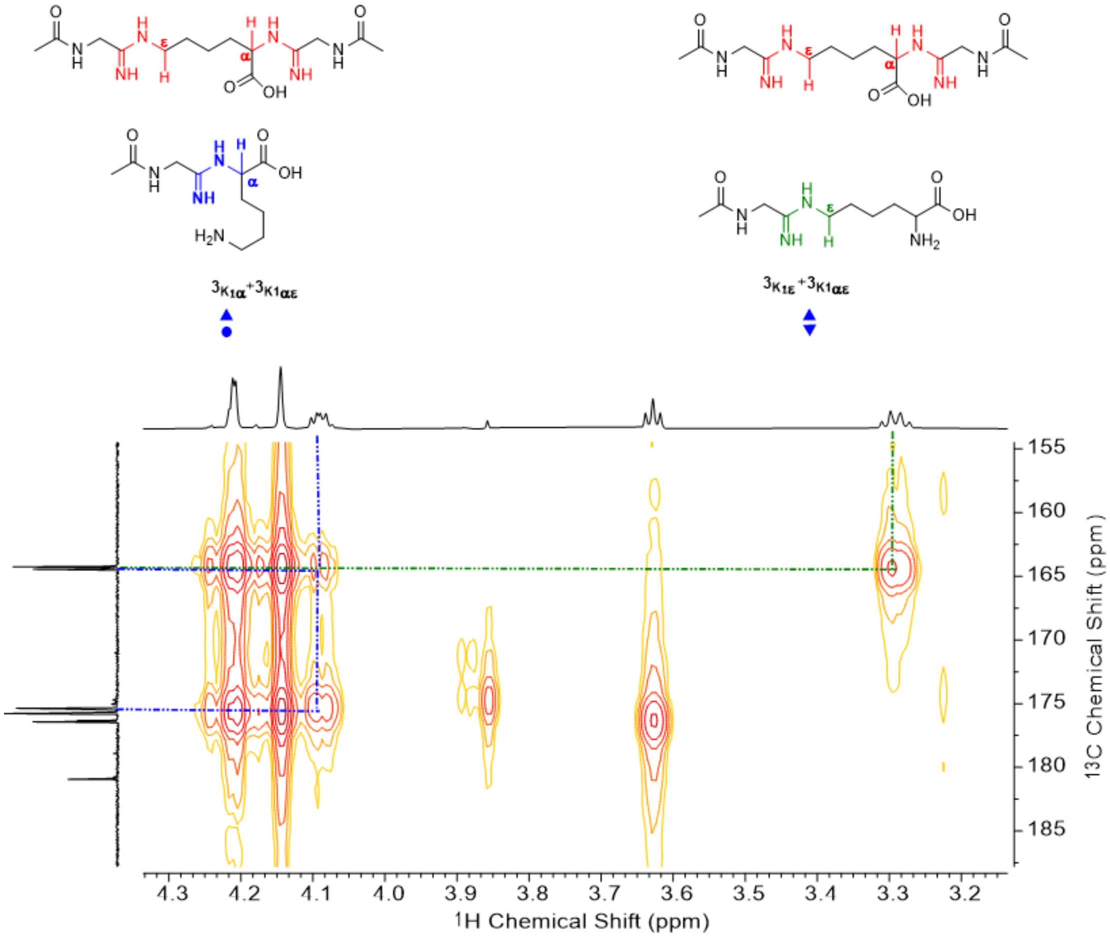

Figure S38: ^{1}H–^{13}C HMBC (^{1}H: 600 MHz [2.0–4.5 ppm], ^{13}C: 176 MHz [150–185 ppm], H_2O/D_2O 9:1) spectrum showing the diagnostic $^{2}J_{CH}$ and $^{3}J_{CH}$ coupling of the lysyl-αH-COOH and lysyl-N⁶-CH₂ in *3$_{K1\alpha\varepsilon}$*(●), *3$_{K1\alpha\varepsilon}$*(▲), *3$_{K2}$* (▼) which are characteristic of amidine bond formations of 2$_K$.

*Coupling of **1** with DL-methionine **2_M** at pH 8.5 and room temperature*

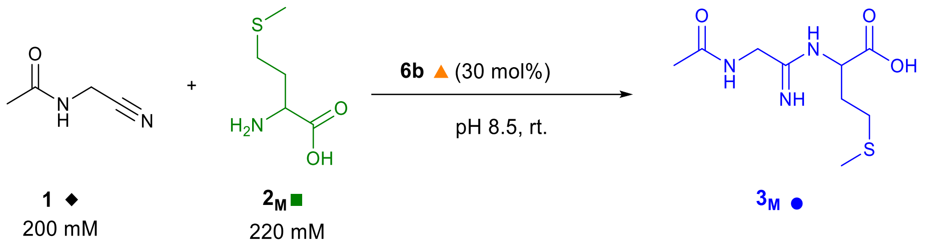

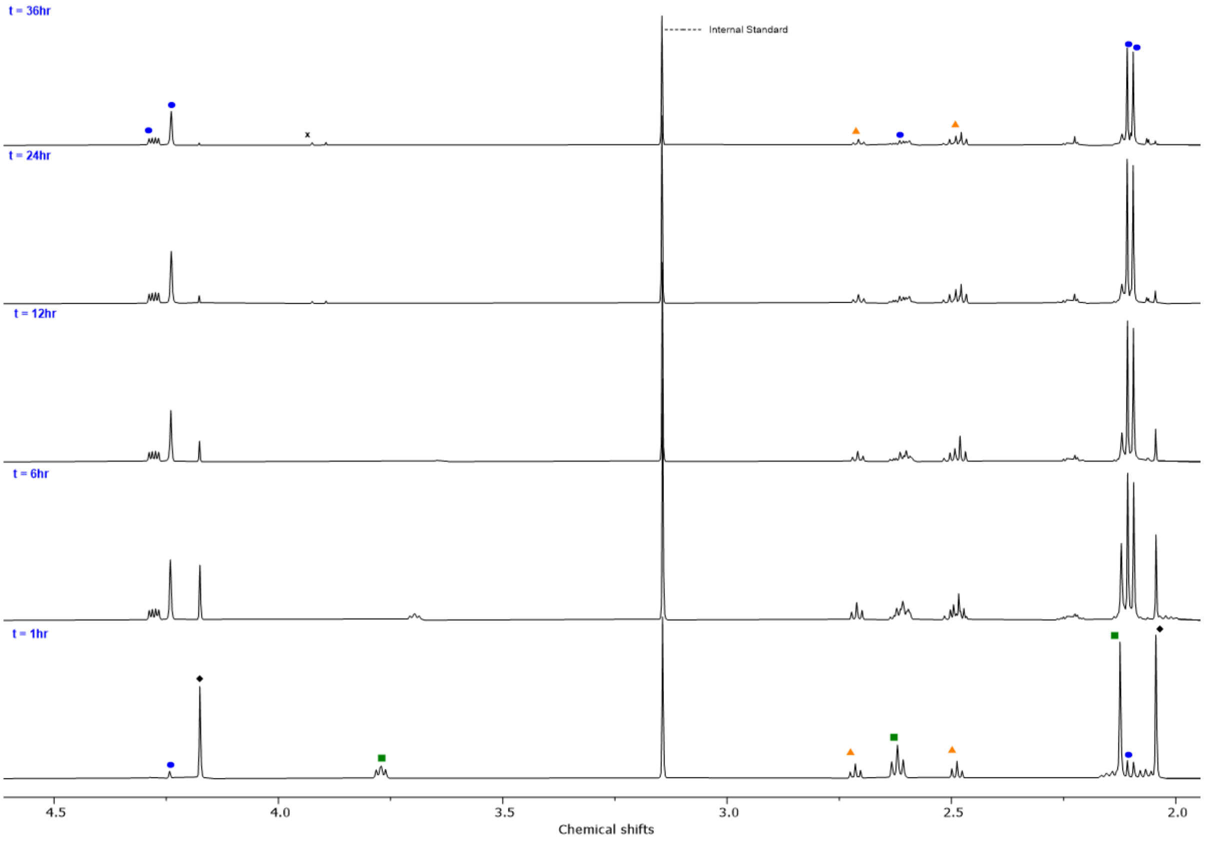

Figure S39: ^{1}H NMR (600 MHz, H_2O/D_2O 9:1, noesygppr1d, 1.9-4.6 ppm) spectrum to show the reaction of N-acetyl glycine nitrile (**1**, 200 mM) with DL-methionine (**2_M**, 220 mM) and 3-mercaptopropanoic acid (**6b**, 60 mM) with MSM (100 mM) as an internal standard pH 8.5 and room temperature.

^{1}H NMR (600 MHz, H_2O/D_2O 9:1, noesygppr1d) *(2-acetamido-1-iminoethyl)methionine*, **3_M** (●) (partial assignment): δ_H 4.28 (1H, dd, J = 8.5, 4.1 Hz, Met-αH-COOH), 4.24 (2H, s, AcNHCH_2), 2.63-2.58 (2H, m, CH_2SCH_3), 2.10 (3H, s, CH_2SCH_3), 2.09 (3H, s, H_3C(CO)); *DL-methionine*, **2_M** (■) (partial assignment): δ_H 3.56 (1H, br. t., J = 5.9 Hz, αH-COOH), 2.49 (2H, m, CH_2SCH_3), 2.11 (3H, s, SCH_3); *N-Acetylglycinamide*, **7** (✖) (partial assignment): δ_H 3.89 (2H, s, CH_2).

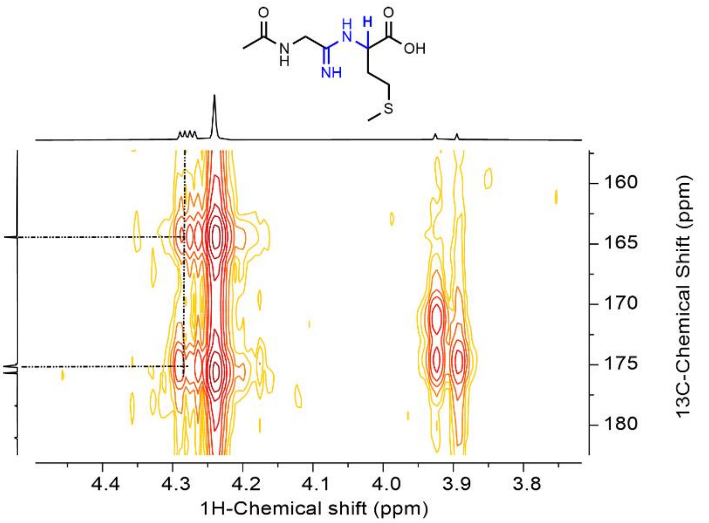

Figure S40: ^1H–^{13}C HMBC (^1H: 600 MHz [3.5-4.5 ppm], ^{13}C: 176 MHz [150-185 ppm], H_2O/D_2O 9:1) spectrum showing the diagnostic $^2J_{CH}$ and $^3J_{CH}$ coupling of the **Met**-αH-COOH in $\mathbf{3_M}$ at 4.28 ppm with two resonances at 176.7 and 165.5 ppm, which is characteristic of amidine bond formation of $\mathbf{2_M}$.

*Coupling of **1** with L-phenylalanine **2_F** at pH 8.5 and room temperature*

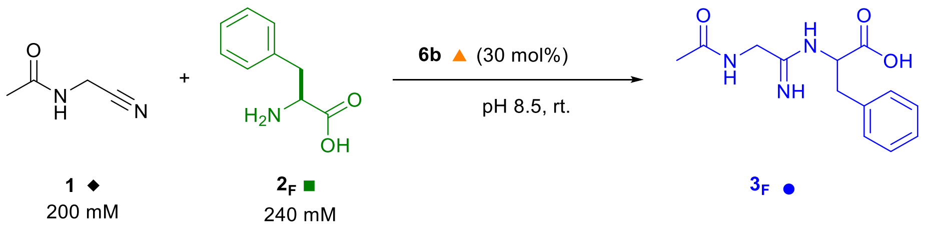

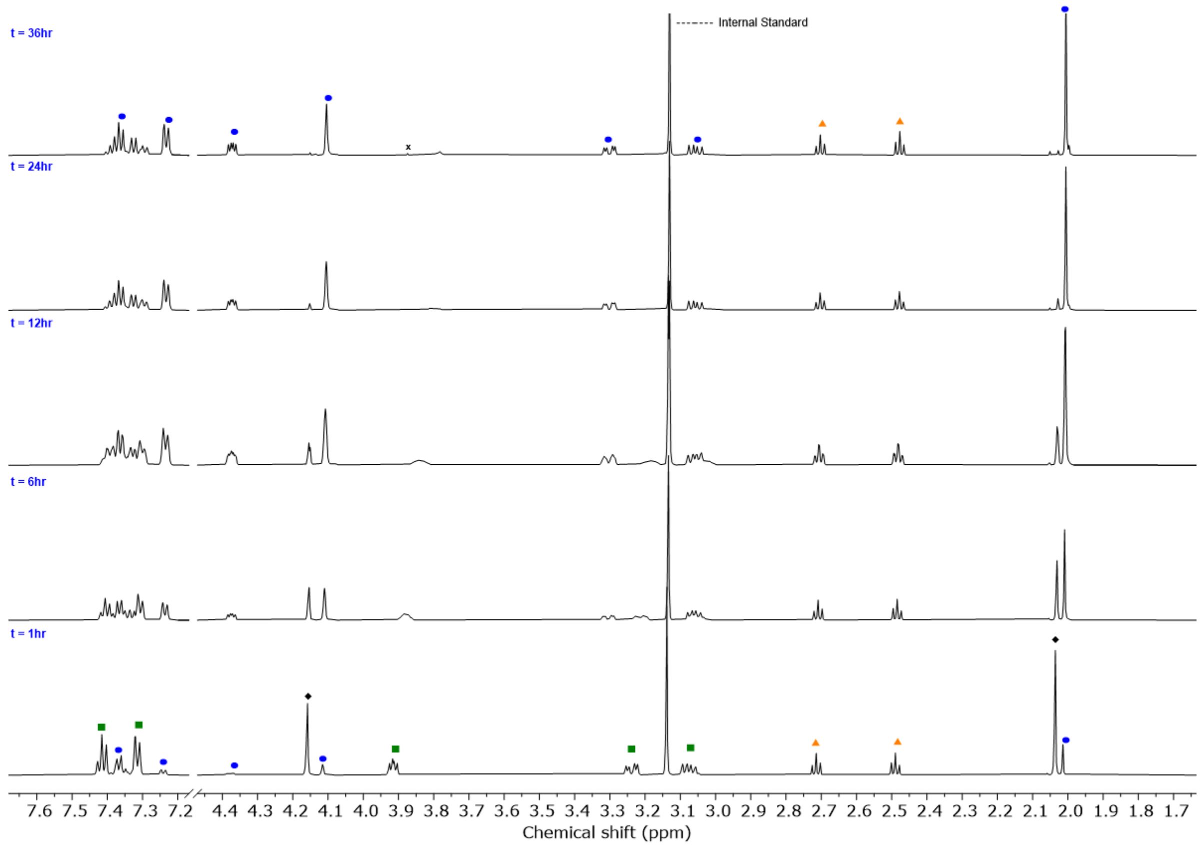

Figure S41: ^{1}H NMR (600 MHz, H_2O/D_2O 9:1, noesygppr1d, 1.6-7.7 ppm) spectrum to show the reaction of N-acetyl glycine nitrile (**1**, 200 mM) with L-phenylalanine (**2_F**, 240 mM) and 3-mercaptopropanoic acid (**6b**, 60 mM) with MSM (100 mM) at pH 8.5 and room temperature.

^{1}H NMR (600 MHz, H_2O/D_2O 9:1, noesygppr1d) *(2-acetamido-1-iminoethyl)phenylalanine*, **3_F** (●) (partial assignment): δ_H 7.41-7.34 (2H, m, Ar*H*), 7.33-7.27 (1H, m, Ar*H*), 7.22 (2H, d, *J* = 8.5 Hz, Ar*H*), 4.37 (1H, ABX, ddd, *J* = 8.1, 4.4, 1.4 Hz, Phe-αH-COOH), 4.10 (1H, AB, *J* = 17.3 Hz, AcNHCH*H*), 4.08 (1H, AB, *J* = 17.3 Hz, AcNHCH*H*), 3.30 (1H, ABX, *J* = 14.2, 4.4 Hz, CHCH*H*Ph), 3.06 (1H, ABX, *J* = 14.2, 8.1 Hz, CHCH*H*Ph), 2.00 (3H, s, H_3C(CO)); **2_F** (■) (partial assignment): δ_H 7.38-7.34 (3H, m, Ar*H*), 7.28 (2H, d, *J* = 7.2 Hz, Ar*H*), 3.68 (1H, br. t, *J* = 6.2 Hz, αH-COOH); *N-acetylglycinamide*, **7** (✖) (partial assignment): δ_H 3.87 (2H, s, CH_2).

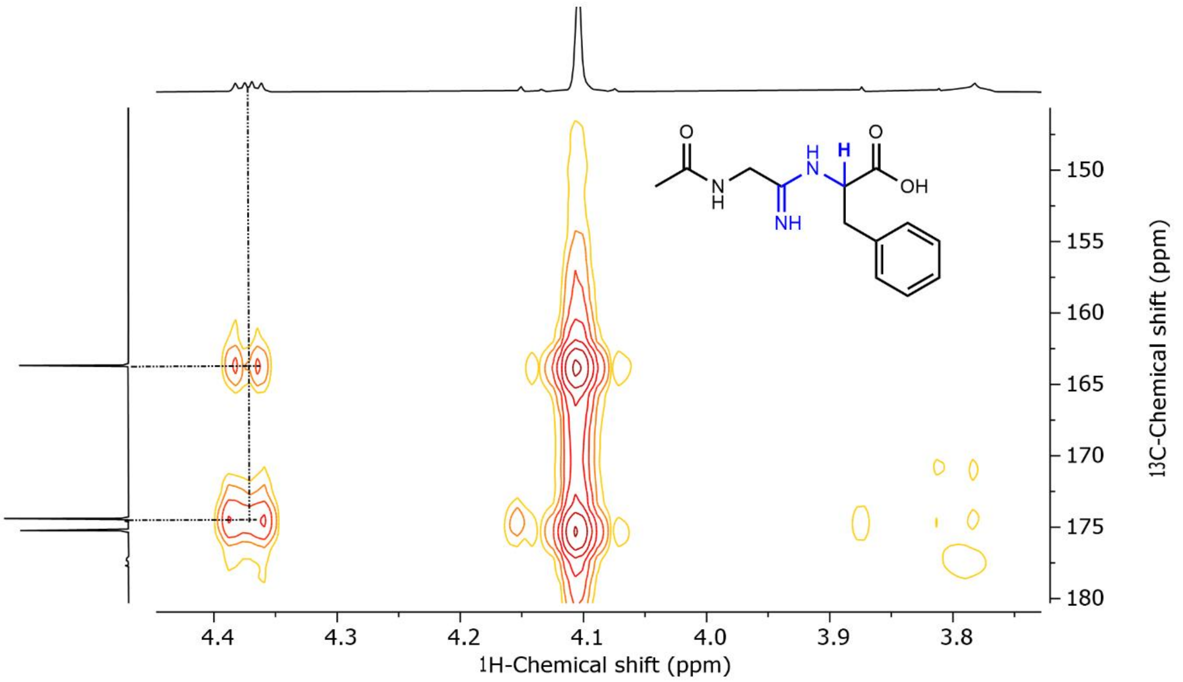

Figure S42: ^{1}H–^{13}C HMBC (^{1}H: 600 MHz [3.5-4.5 ppm], ^{13}C: 176 MHz [150-185 ppm], H_2O/D_2O 9:1) spectrum showing the diagnostic $^{2}J_{CH}$ and $^{3}J_{CH}$ coupling of the **Phe**-αH-COOH in **3**$_{\mathbf{F}}$ at 4.37 ppm with two resonances at 175.5 and 164.6 ppm, which is characteristic of amidine bond formation of **2**$_{\mathbf{F}}$.

*Coupling of **1** with L-proline **2P** at pH 8.5 and room temperature*

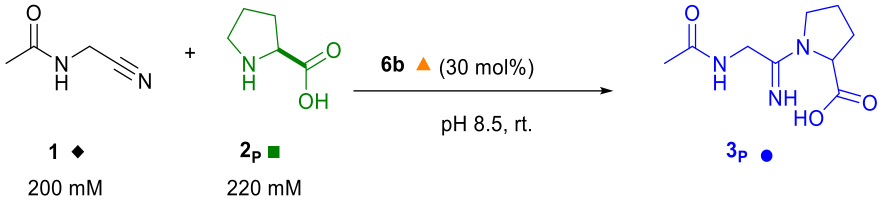

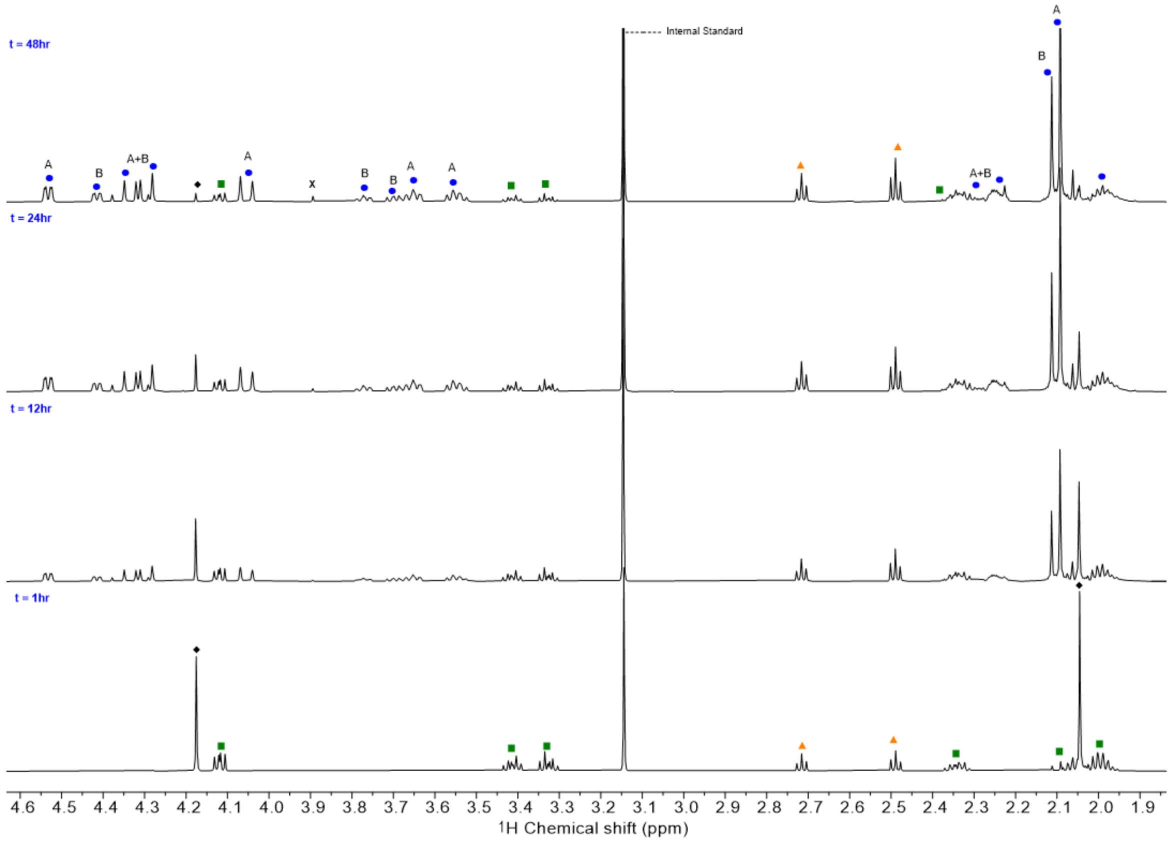

Figure S43: ^{1}H NMR (600 MHz, H_2O/D_2O 9:1, noesygppr1d, 1.8-4.6 ppm) spectrum to show the reaction of N-acetyl glycine nitrile (**1**, 200 mM) with L-proline (**2P**, 230 mM) and 3-mercaptopropanoic acid (**6b**, 60 mM) with MSM (100 mM) as an internal standard at pH 8.5 and room temperature.

^{1}H NMR (600 MHz, H_2O/D_2O 9:1, noesygppr1d) *(2-acetamido-1-iminoethyl)proline*, mixture of rotamers [A:B, 63:37], **3P** (*major rotamer*: A, ●) (partial assignment): δ_H 4.53 (1H, dd, *J* = 8.8, 2.5 Hz, Pro-αH-COOH), 4.29 (1H, AB, *J* = 17.3 Hz, AcNHCH*H*), 4.05 (1H, AB, *J* = 17.3 Hz, AcNHCH*H*), 3.67-3.63 (1H, m, NCH*H*CH_2CH_2), 3.56-3.52 (1H, m, NCH*H*CH_2CH_2), 2.08 (3H, s, H_3C(CO)), (*minor rotamer*: B, ●) (partial assignment): δ_H 4.40 (1H, dd, *J* = 8.5 2.5 Hz, Pro-αH-COOH), 4.36 (1H, AB, *J* = 17.5 Hz, AcNHCH*H*), 4.29 (1H, AB, *J* = 17.5 Hz, AcNHCH*H*), 3.78-3.75 (1H, m, NCH*H*CH_2CH_2), 3.71-3.68 (1H, m, NCH*H*CH_2CH_2), 2.10 (3H, s, H_3C(CO)); *L-proline*, **2P** (■) (partial assignment): δ_H 4.10 (1H, dd, *J* = 8.9, 6.6 Hz, αH-COOH), 3.40 (1H, dt, *J* = 11.6, 7.0 Hz, HNCH*H*CH_2CH_2), 3.31 (1H, dt, *J* = 11.6, 7.0 Hz, HNCH*H*CH_2CH_2); *N-acetylglycinamide*, **7** (✖) (partial assignment): δ_H 3.89 (2H, s, CH_2).

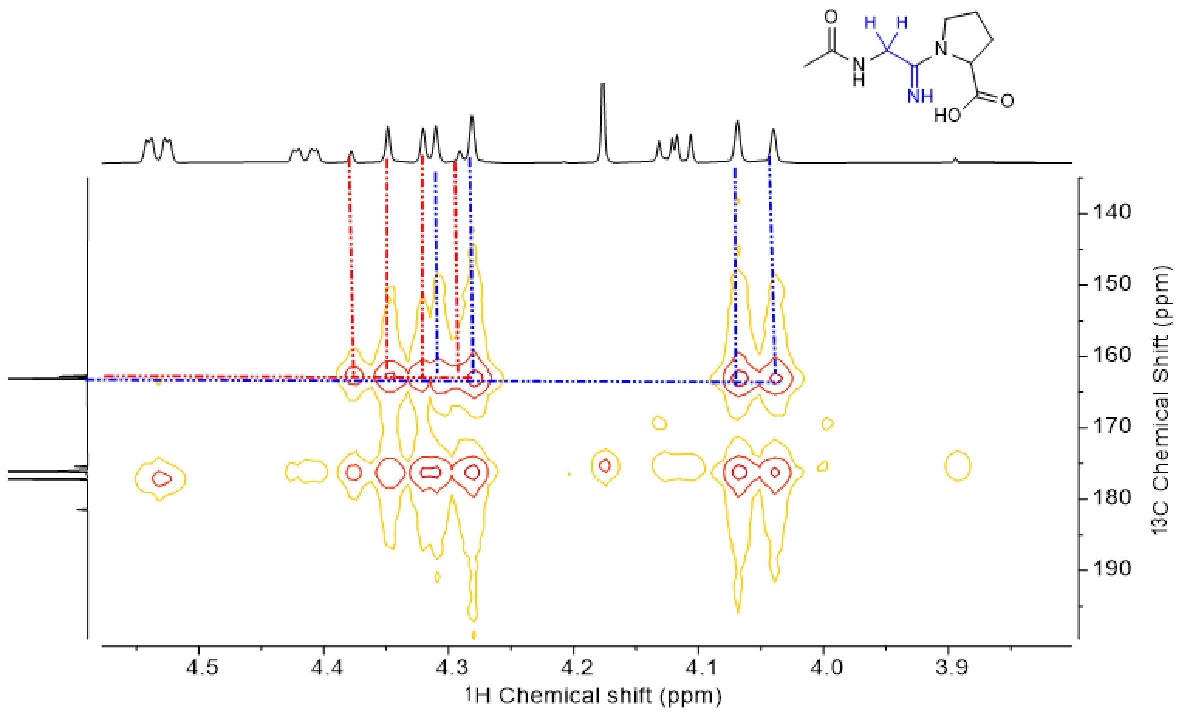

Figure S44: . ^1H–^{13}C HMBC (^1H: 600 MHz [3.5-4.5 ppm], ^{13}C: 176 MHz [150-180 ppm], H_2O/D_2O 9:1) spectrum showing the diagnostic $^2J_{CH}$ coupling of the Gly-CH_2 AB systems in both rotamers of **3P** at 4.37 and 4.34, 4.31 and 4.28, 4.30 and 4.29, and 4.06 and 4.03 ppm with two resonances at 163.2 and 162.7 ppm, which is characteristic of amidine bond formation of **2P**.

*Coupling of **1** with L-serine **2**$_S$ at pH 8.5 and room temperature*

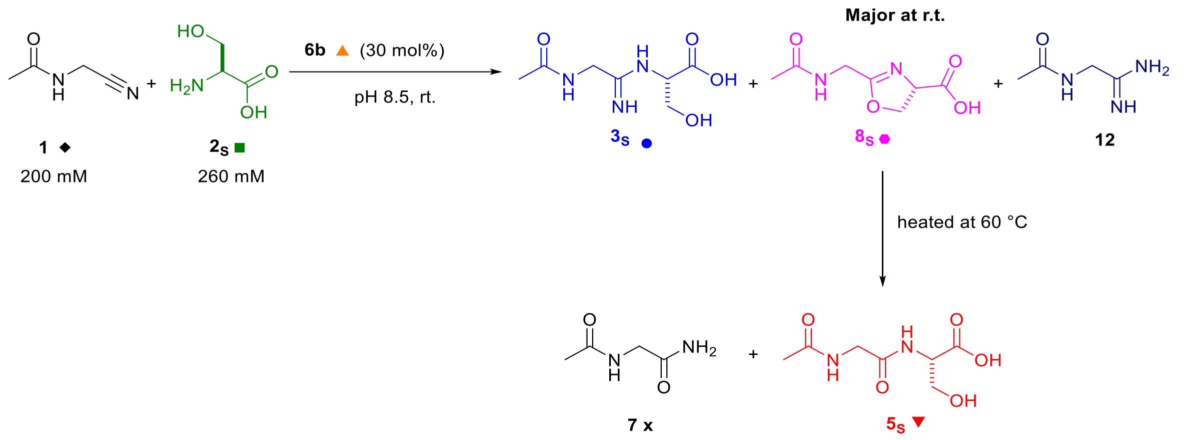

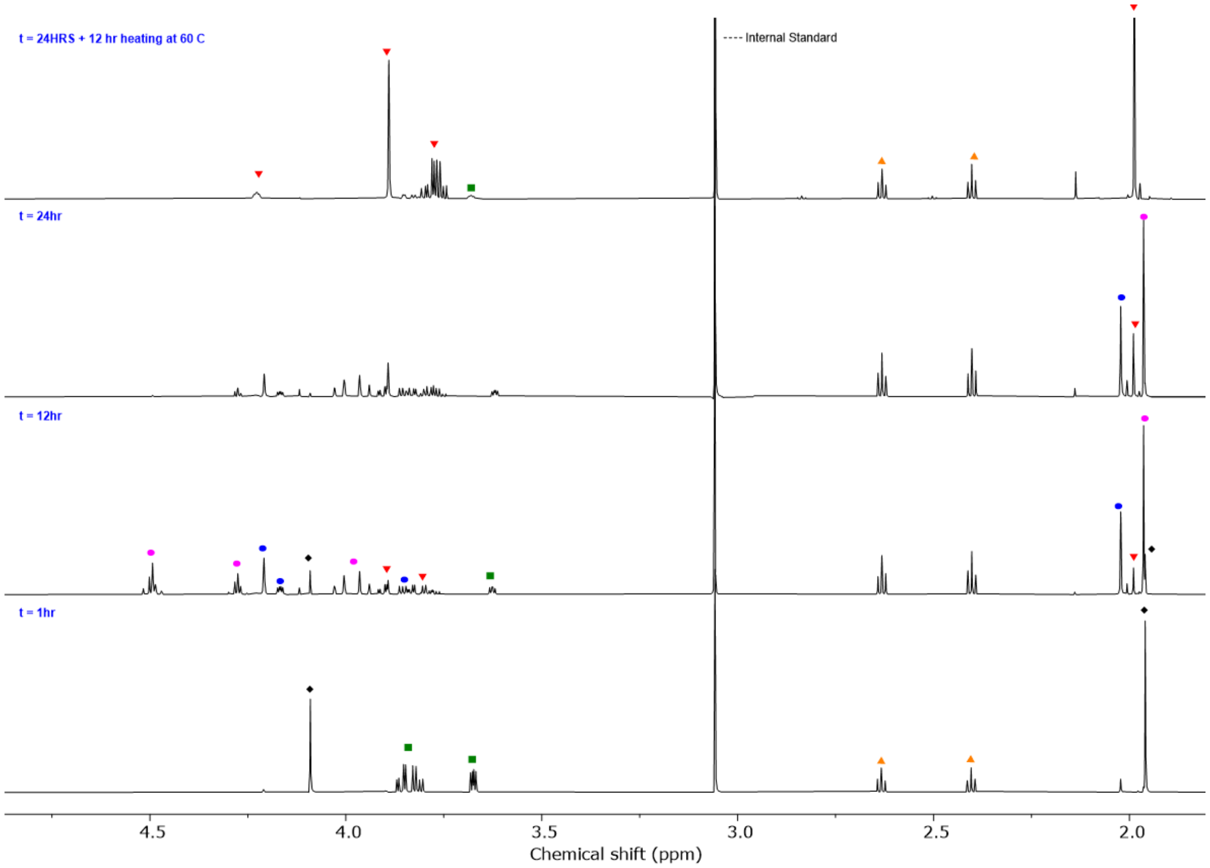

Figure S45: ^{1}H NMR (600 MHz, H_2O/D_2O 9:1, noesygppr1d, 1.8-5.0 ppm) spectrum to show the reaction of N-acetyl glycine nitrile (**1**, 200 mM) with L-serine (**2**$_S$, 260 mM) and 3-mercaptopropanoic acid (**6b**, 60 mM) with MSM (100 mM) as an internal standard at pH 8.5 and room temperature.

After 24 h the following species were observed:

^{1}H NMR (700 MHz, H_2O/D_2O 9:1, noesygppr1d): partial assignment for (2-acetamido-1-iminoethyl)serine, **3**$_S$ (●): δ_H 4.24 (2H, s, br., AcNHC*H*$_2$), 4.20 (1H, ABX, *J* = 5.8, 3.4 Hz, Ser-α*H*-COOH), 4.00-3.97 (1H, obs., CHCH*H*OH), 3.94 (1H, ABX, *J* = 11.9, 3.4 Hz, CHC*H*HOH), 2.02 (3H, s, H_3C(CO)); partial assignment for (2-(acetamidomethyl)-4,5-dihydrooxazole-4-carboxylic acid **8**$_S$ (●): δ_H 4.60-4.48 (2H, m, C*H*HCHCOOH), 4.31 (1H, dd, *J* = 5.5, 4.8 Hz,

CH*H*CHCOOH), 4.05 (1H, d, *J* = 17.3, 1.4 Hz, AcNHCH*H*), 3.99 (1H, AB, *J* = 17.3 Hz, 1.4 Hz, AcNHC*H*H), 2.00 (3H, s, $H_3C(CO)$).

After complete consumption of **1** the reaction was heated at 60°C for 16 h, which resulted in the formation of **5S**.

^1H NMR (700 MHz, H_2O/D_2O 9:1, noesygppr1d): *N-Acetylglycylserine*, **5S** (▼): δ_H 4.27 (1H, t, *J* = 4.7 Hz, Ser-αH-COOH), 3.93 (2H, s, AcNHCH_2), 3.89-3.61 (2H, m, CHCH_2OH), 2.03 (3H, s, $H_3C(CO)$); *L-serine*, **2S** (■): δ_H 3.92-3.86 (2H, m, CHCH_2OH), 3.73-3.70 (1H, br. t, *J* = 4.4 Hz, αH-COOH); *N-acetylglycinamide*, **7** (✖): (partial assignment) δ_H 3.85 (2H, s, CH_2).

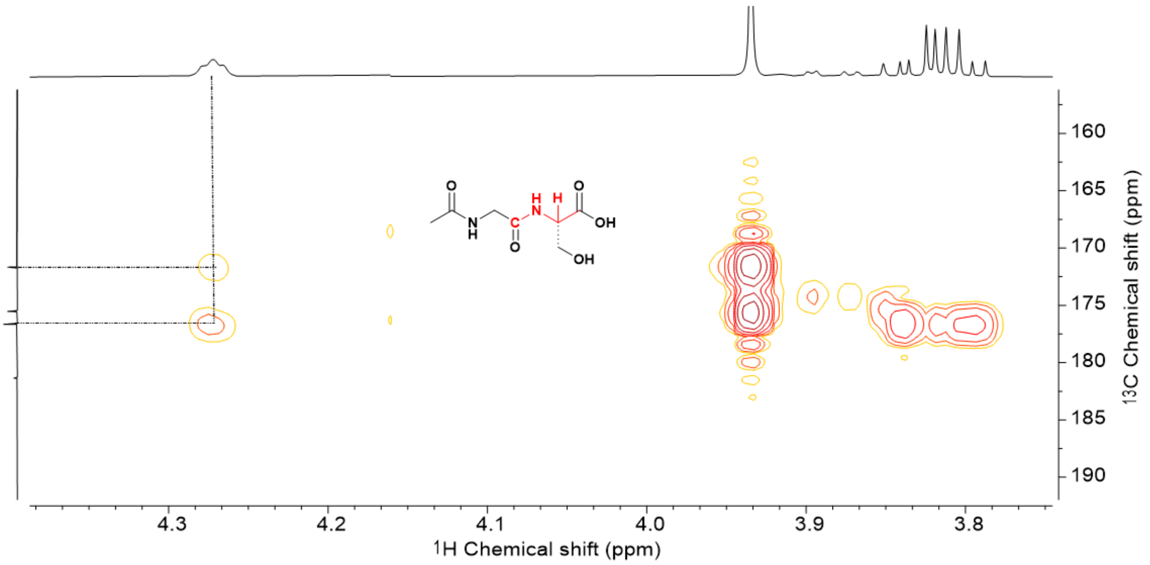

Figure S46: ^1H–^{13}C HMBC (^1H: 700 MHz [3.7–4.4 ppm], ^{13}C: 176 MHz [150-185 ppm], H_2O/D_2O 9:1) spectrum showing the diagnostic $^2J_{CH}$ and $^3J_{CH}$ coupling of the **Ser**-αH-COOH in **5S** at 4.27 ppm with two resonances at 176.6 and 171.7 ppm, which is characteristic of amide bond formation of **2S**.

*Coupling of **1** with L-tryptophan **2**$_W$ at pH 8.5 and room temperature*

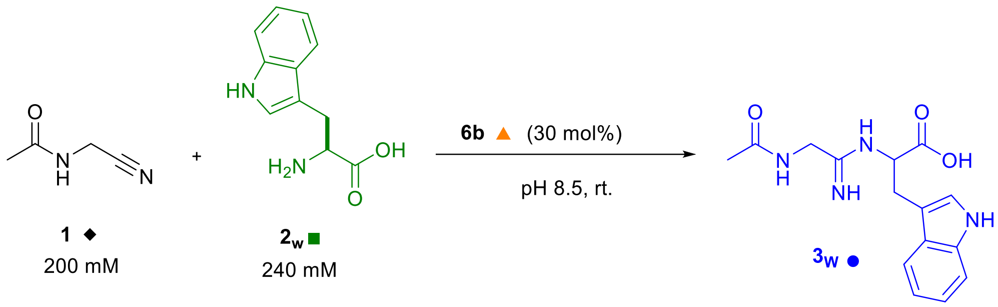

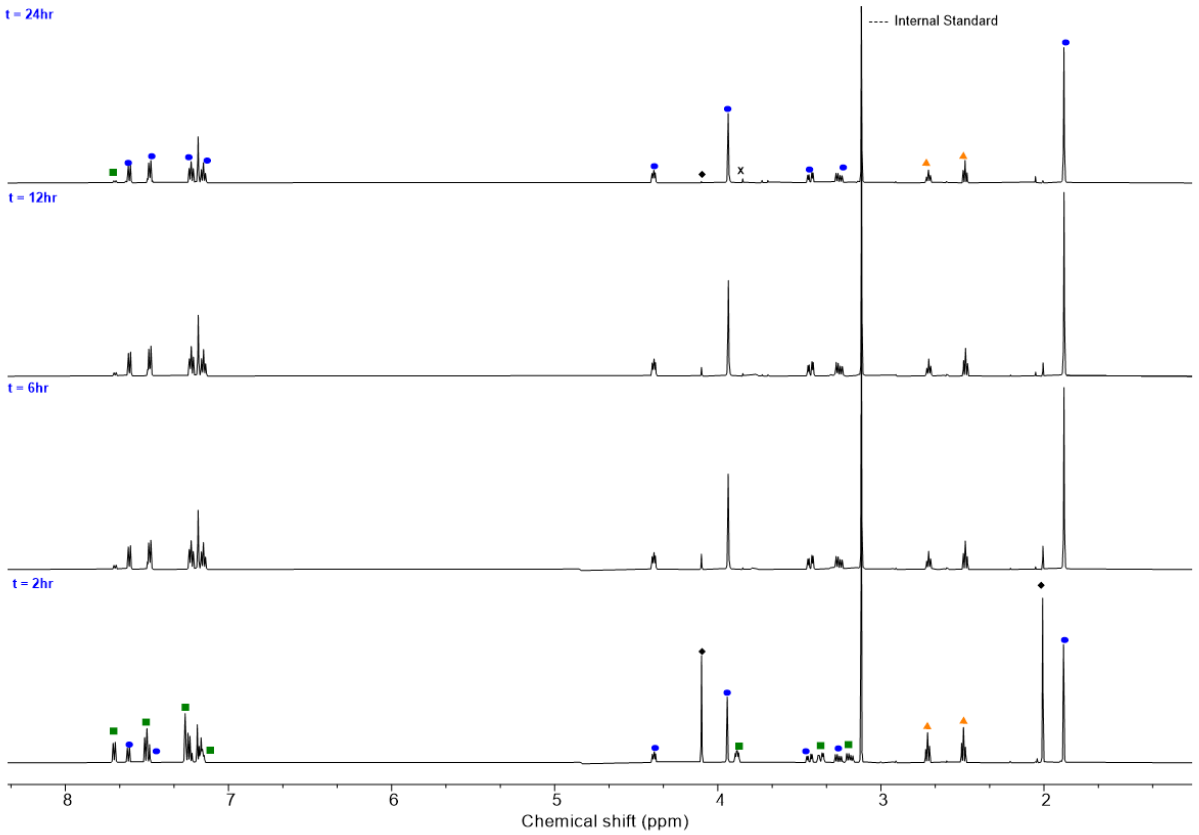

Figure S47: ^{1}H NMR (600 MHz, H_2O/D_2O 9:1, noesygppr1d, 1.0-8.0 ppm) spectrum to show the reaction of N-acetyl glycine nitrile (**1**, 200 mM) with L-tryptophan (**2**$_W$, 238 mM) and 3-mercaptopropanoic acid (**6b** 60 mM) with MSM (100 mM) as an internal standard at pH 8.5 and room temperature.

^{1}H NMR (600 MHz, H_2O/D_2O 9:1, noesygppr1d) *(2-acetamido-1-iminoethyl)tryptophan*, **3**$_W$ (●): δ_H 7.65 (1H, d, *J* = 8.3 Hz, Ar*H*), 7.48 (1H, d, *J* = 8.6 Hz, Ar*H*), 7.21-7.25 (1H, m, Ar*H*), 7.19 (1H, br, Ar*H*), 7.15 (1H, t, *J* = 7.5 Hz, Ar*H*), 4.39 (1H, ABX, *J* = 7.3, 4.7 Hz, Trp-αH-COOH), 3.94 (2H, s, AcNHC*H*$_2$), 3.43 (1H, ABX, *J* = 15.1, 4.7 Hz, CHC*H*HAr), 3.26 (1H, ABX, *J* = 15.1, 7.3 Hz, CHCH*H*Ar), 1.88 (3H, s, *H*$_3$C(CO)); *L-tryptophan*, **2**$_W$ (■): δ_H 7.67 (1H, d, *J* = 8.1 Hz, Ar*H*), 7.46 (1H, d, *J* = 8.1 Hz, Ar*H*), 7.22 (1H, s, Ar*H*), 7.21-7.19 (1H, m, Ar*H*), 7.14-7.11 (1H, m, Ar*H*), 3.83 (1H, ABX, *J* = 7.7, 4.9 Hz, αH-COOH), 3.32 (1H, dd, *J* = 15.1, 4.9 Hz, CHC*H*HAr), 3.17 (1H, ABX, *J* = 15.1, 7.7 Hz, CHCH*H*Ar); *N-acetylglycinamide*, **7** (✖) (partial assignment): δ_H 3.84 (2H, s, C*H*$_2$).

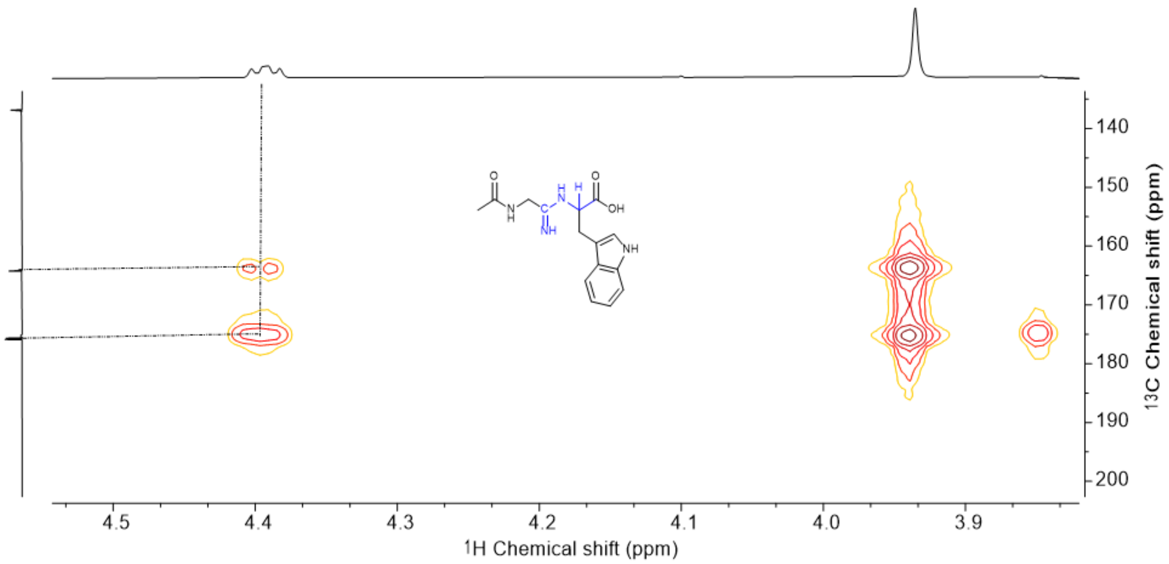

Figure S48: ^1H–^{13}C HMBC (^1H: 600 MHz [3.7–5.0 ppm], ^{13}C: 176 MHz [140–200 ppm], H_2O/D_2O 9:1) spectrum showing the diagnostic $^2J_{CH}$ and $^3J_{CH}$ coupling of the **Trp**-αH-COOH in **3w** at 4.39 ppm with two resonances at 175.7 and 164.2 ppm, which is characteristic of amidine bond formation of **2w**.

*Coupling of **1** with L-threonine **2_T** at pH 8.5 and room temperature*

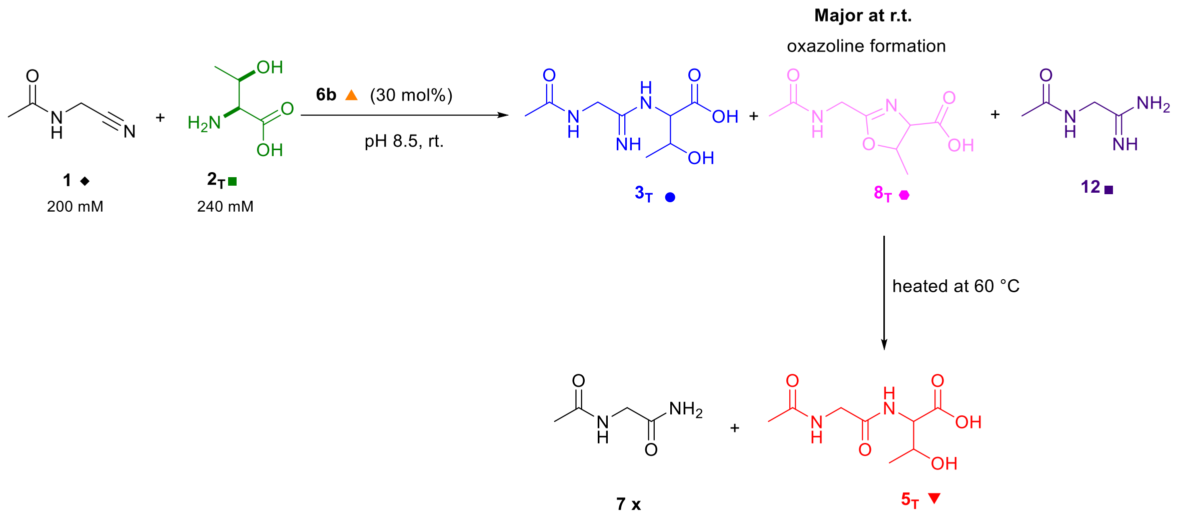

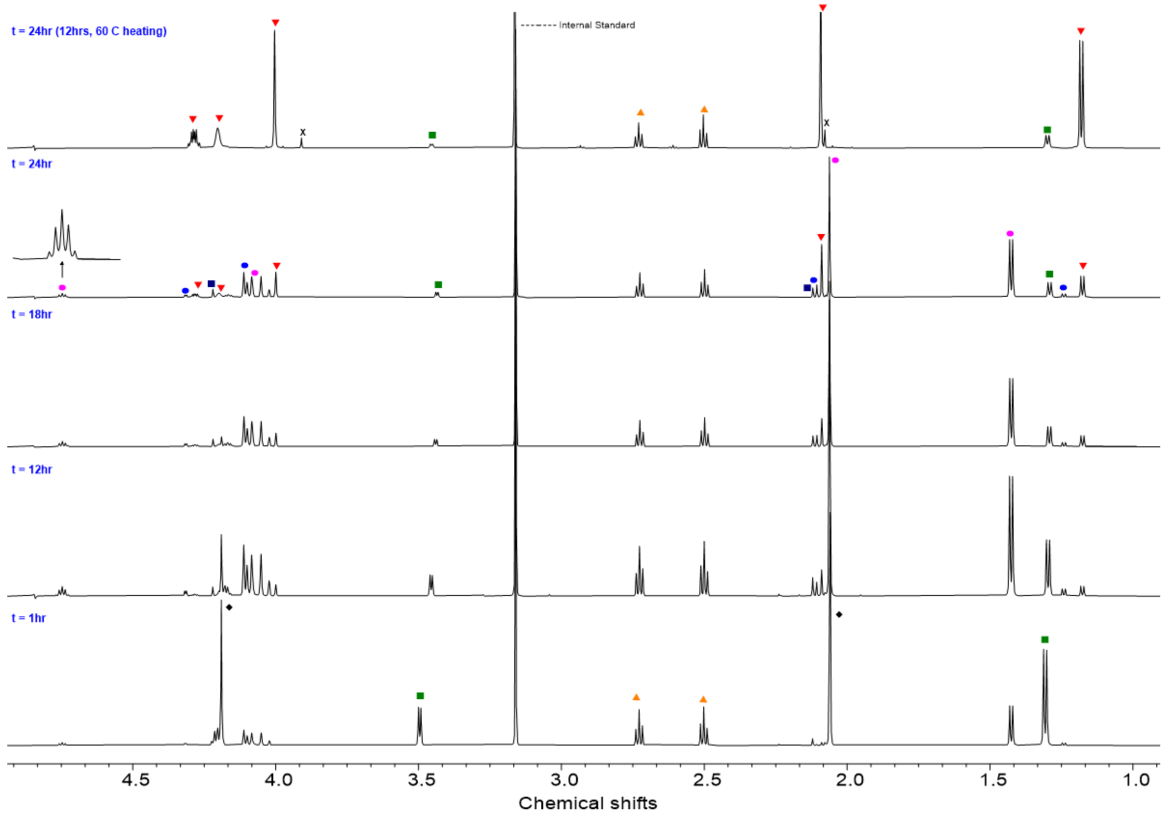

Figure S49: ^{1}H NMR (600 MHz, H_2O/D_2O 9:1, noesygppr1d, 1.0–4.7 ppm) spectrum to show the reaction of N-acetyl glycine nitrile (**1**, 200 mM) with L-threonine (**2_T**, 240 mM) and 3-Mercaptopropanoic acid (**6b**, 60 mM) with MSM (100 mM) as an internal standard at pH 9 and room temperature.

After 24 h following species were observed:

2-(Acetamidomethyl)-5-methyl-4,5-dihydrooxazole-4-carboxylic acid, **8_T** (●) (partial assignment - $OCHCH_3$ resonance partially supressed due to proximity to the HOD peak), δ_H 4.75 (1H, p, J = 6.3 Hz, OC*H*CH_3), 4.09 (1H, AB, J = 17.4 Hz, AcNHCH*H*), 4.03 (1H, AB, J = 17.4 Hz, AcNHC*H*H), 2.06 (3H, s, *H*$_3$C(CO)), 1.43 (3H, d, J = 6.3 Hz, HOCHC*H*$_3$); **3_T** (●) δ_H 4.40 (1H, qd, J = 6.4, 3.7 Hz, HOCHCH_3), 4.32 (1H, br, Thr-α*H*-COOH) and **12** (■) (partial assignment): δ_H 4.24 (2H, s, CH_2).

After complete consumption of **1** the reaction was heated at 60°C for 16 h, which resulted in formation of **5_T**.

^{1}H NMR (600 MHz, H_2O/D_2O 9:1, noesygppr1d) *N-Acetylglycylthreonine*, **5_T** (▼): δ_H 4.29 (1H, qd, J = 6.4, 3.7 Hz, HOC*H*CH3), 4.20 (1H, br, Thr-αH-COOH), 4.03 (1H, AB, J = 17.3 Hz, AcNHCH*H*), 4.00 (1H, AB, J = 17.3 Hz, AcNHCH*H*), 2.09 (3H, s, *H*3C(CO)), 1.18 (3H, d, J = 6.5 Hz, HOCHC*H*3); *L-threonine*, **2_T** (■): 3.45 (1H, d, J = 4.8 Hz, αH-COOH), 1.30 (3H, d, J = 6.6 Hz, HOCHC*H*3); *N-acetylglycinamide*, **7** (✖) (partial assignment): δ_H 3.91 (2H, s, C*H*2), 2.08 (3H, s, *H*3C(CO)).

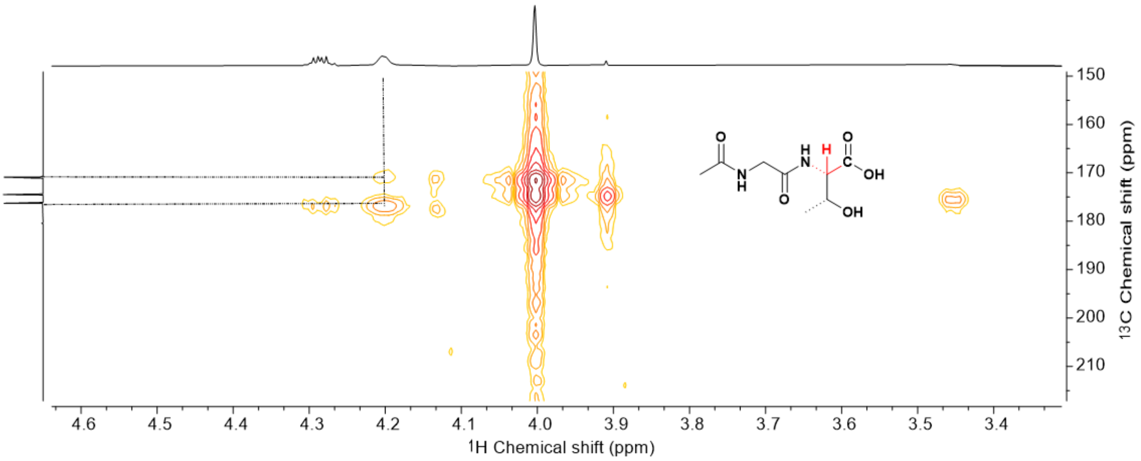

Figure S50: ^{1}H–^{13}C HMBC (^{1}H: 700 MHz [3.4-4.6 ppm], ^{13}C: 176 MHz [150–185 ppm], H_2O/D_2O 9:1) spectrum showing the diagnostic $^2J_{CH}$ and $^3J_{CH}$ coupling of the Thr-αH-COOH in 5_T at 4.19 ppm with two resonances at 177.4 and 171.5 ppm, which is characteristic of amide bond formation of 2_T.

*Coupling of **1** with L-valine **2**$_V$ at pH 8.5 and room temperature*

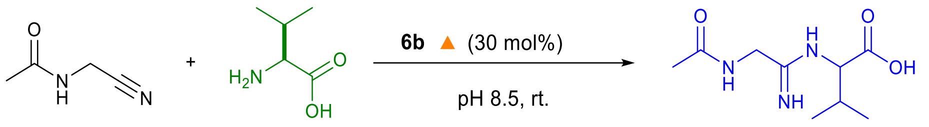

1 ◆ 200 mM **2**$_V$ ■ 220 mM **3**$_V$ ●

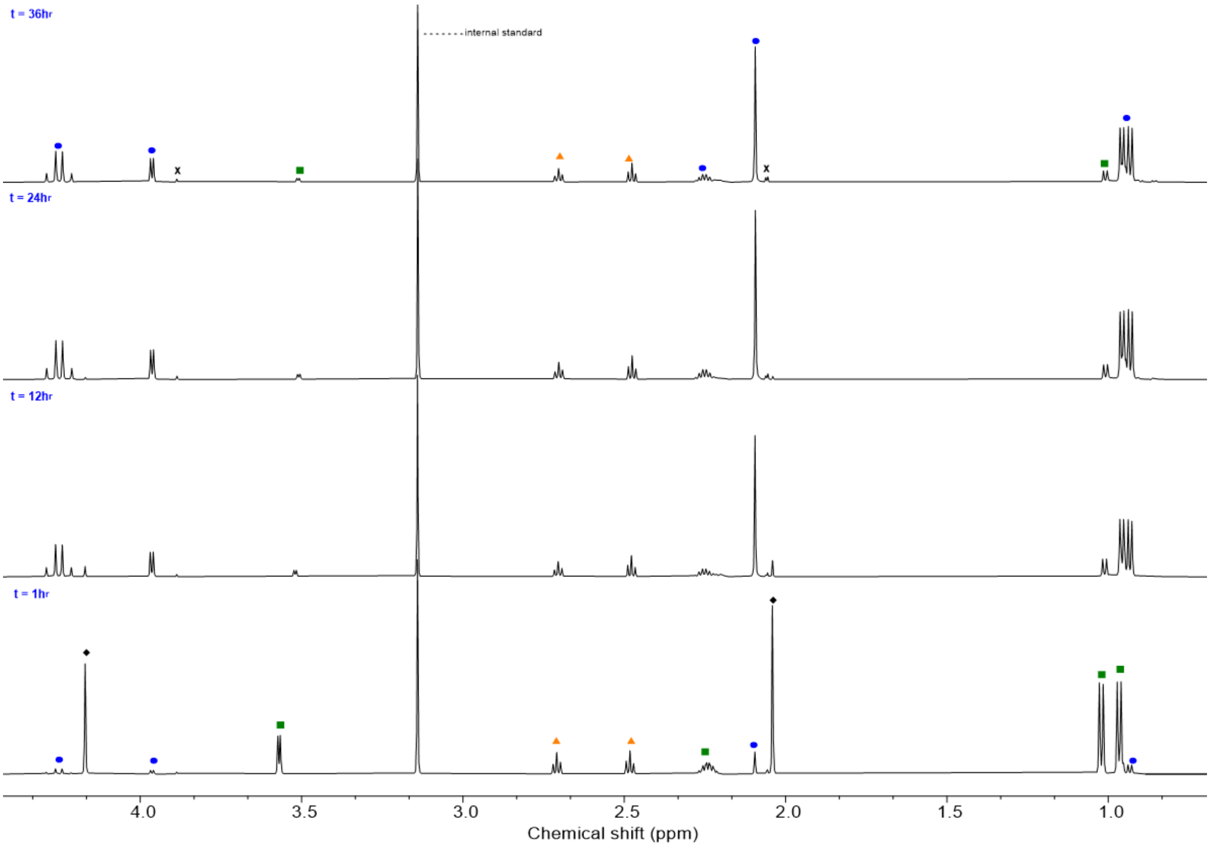

Figure S51: ^{1}H NMR (600 MHz, H_2O/D_2O 9:1, noesygppr1d, 0.8–5.0 ppm) spectrum to show the reaction of N-acetyl glycine nitrile (**1**, 200 mM) with L-valine (**2**$_V$, 220 mM) and 3-mercaptopropanoic acid (**6b**, 60 mM) with MSM (100 mM) as an internal standard at pH 8.5 and room temperature.

^{1}H NMR (600 MHz, H_2O/D_2O 9:1, noesygppr1d) *(2-acetamido-1-iminoethyl)valine*, **3**$_V$ (●): δ_H 4.28 (1H, AB, *J* = 17.1 Hz, AcNHCH*H*), 4.23 (1H, AB, *J* = 17.1 Hz, AcNHCH*H*), 3.97 (1H, d, *J* = 5.5 Hz, Val-αH-COOH), 2.31-2.24 (1H, m, H_3CCHCH_3), 2.10 (3H, s, $H_3C(CO)$), 0.95 (3H, d, *J* = 6.7 Hz, CH_3), 0.92 (3H, d, *J* = 7.0 Hz, CH_3); *L-valine*, **2**$_V$ (■): δ_H 3.53 (1H, br. d. *J* = 4.4 Hz, αH-COOH), 2.17-2.12 (1H, m, H_3CCHCH_3), 1.02 (3H, d, *J* = 7.0 Hz, CH_3), 0.93 (3H, d, *J* = 7.0 Hz, CH_3); *N-acetylglycinamide*, **7** (✖) (partial assignment): δ_H 3.90 (2H, s, CH_2), 2.06 (3H, s, CH_3).

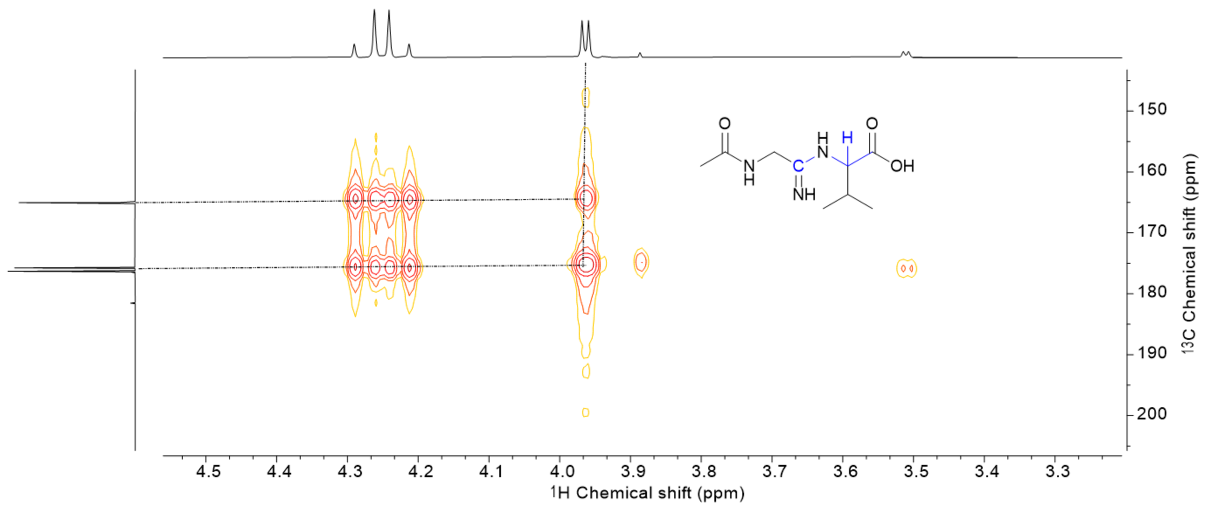

Figure S52: ^{1}H–^{13}C HMBC (^{1}H: 600 MHz [3.2-4.5 ppm], ^{13}C: 176 MHz [150–185 ppm], H_2O/D_2O 9:1) spectrum showing the diagnostic $^2J_{CH}$ and $^3J_{CH}$ coupling of the **Val**-αH-COOH in **3v** at 3.94 ppm with two resonances at 175.8 and 164.9 ppm, which is characteristic of amidine bond formation.

*Competitive coupling of **1** with glycine **2G** and ammonium chloride*

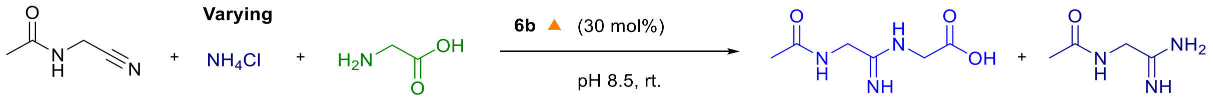

1 ◆		2_G ■	3_G ●	12 ■
200 mM	400 mM	200 mM		
200 mM	1 M	200 mM		
200 mM	2 M	200 mM		

Entry	Nucleophile stoichiometry (equiv.)		**Selectivity** **3G(●) : 12(■)** /% time / 24 h
	Gly-OH (■)	NH₄Cl	
1	1	2	5.5: 1
2	1	5	2.6: 1
3	1	10	1.2: 1

Table S19: Ratio for the formation of 3_G ● and 12 ■

Reaction conditions are as following 3-mercaptopropanoic acid (**6b**, 60 mM) ▲ catalyzed formation of 3_G ● and 12 ■ from the coupling of N-acetyl glycine nitrile (**1**, 200 mM) with glycine ■ (**2G**, 1 equiv.) and ammonium chloride (2 equiv., 5 equiv. and 10 equiv.) at pH 8.5, room temperature, after 12 h, unless stated otherwise.

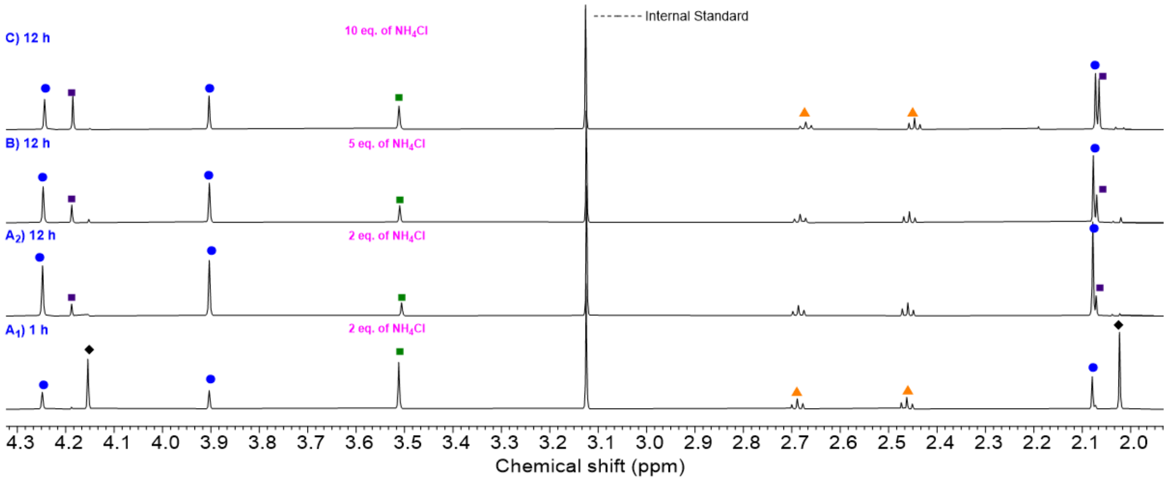

Figure S53: ^{1}H NMR (600 MHz, H_2O/D_2O 9:1, noesygppr1d, 1.0-5.0 ppm) spectrum to show the reaction of N-acetyl glycine nitrile (**1**, 200 mM) with glycine (**2**$_G$, 200 mM), ammonium chloride (NH_4Cl, 2 equiv., 5 equiv. and 10 equiv.), and 3-mercaptopropanoic acid (**6b**, 60 mM) with MSM (100 mM) as an internal standard after 12 h at pH 8.5. A$_1$) ^{1}H spectrum shows reaction of **1** with 2 equiv. of NH_4Cl and 1 equiv. of **2**$_G$ after 1 hrs, A$_2$) reaction of **1** with 2 equiv. of NH_4Cl and 1 equiv. of **2**$_G$ after 12 hrs B) reaction of **1** with 5 equiv. of NH_4Cl and 1 equiv. of **2**$_G$ after 12 hrs C) reaction of **1** with 10 equiv. of NH_4Cl and 1 equiv. of **2**$_G$ after 12 hrs.

^{1}H NMR (600 MHz, H_2O/D_2O 9:1, noesygppr1d) *(2-acetamido-1-iminoethyl)glycine*, **3**$_G$ (●): δ_H 4.26 (2H, s, AcNHC*H*$_2$), 3.91 (2H, s, Gly-αH-COOH), 2.09 (3H, s, *H*$_3$C(CO)); *2-(2-acetamidoacetimidamido)acetamide*, 1**2** (■): δ_H 4.19 (2H, s, AcNHC*H*$_2$), 2.08 (3H, s, *H*$_3$C(CO)); *glycine*, **2**$_G$ (■): δ_H 3.51 (2H, s, C*H*$_2$).

*Coupling of N-acetylglycine nitrile **1** with a-amino amides **2'** at pH 8.5 and room temperature*

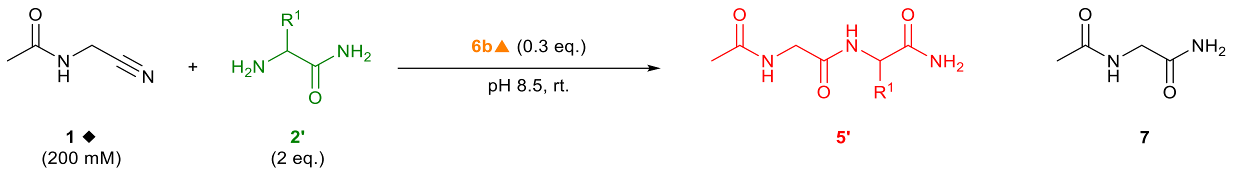

Amino amide	time / h	Yield /%		HRMS FOR 5'		
		5'	7	Formula	Theoretical	Found
Glycinamide (**2**$_G$**'**)	144	50	5	$[C_6H_{11}N_3O_3+Na]^+$	196.0693	196.0690 (ESI pos.)
L-Alaninamide (**2**$_A$**'**)	144	76	8	$[C_7H_{13}N_3O_3+H]^+$	188.1030	188.1030 (ESI pos.)
L-Arginine amide (**2**$_R$**'**)	120	80	18	$[C_{10}H_{15}N_3O_5+H]^+$	273.1670	273.1669 (ESI pos.)
L-Asparagine amide (**2**$_N$**'**)	240	50	45	$[C_8H_{14}N_4O_4+Na]^+$	253.0907	253.0910 (ESI pos.)
L-Aspartate amide (**2**$_D$**'**)	144	83	17	$[C_8H_{13}N_3O_5+Na]^+$	254.0747	254.0749 (ESI pos.)
L-Glutamine amide (**2**$_Q$**'**)	120	66	19	$[C_9H_{16}N_4O_4+H]^+$	245.1244	245.1243 (ESI pos.)
L-Glutamate amide (**2**$_E$**'**)	120	84	15	$[C_9H_{15}N_3O_5+Na]^+$	268.0904	268.0905 (ESI pos.)
L-Histidine amide (**2**$_H$**'**)	125	84	13	$[C_{10}H_{15}N_5O_3+H]^+$	254.1248	254.1246 (ESI pos.)
L-Isoleucinamide (**2**$_I$**'**)	168	86	8	$[C_{10}H_{19}N_3O_3+H]^+$	230.1499	230.1496 (ESI pos.)
L-Leucinamide (**2**$_L$**'**)	144	81	12	$[C_{10}H_{19}N_3O_3+H]^+$	230.1499	230.1499 (ESI pos.)
L-Lysinamide (**2**$_K$**'**)	120	42	16	$[C_{10}H_{12}N_4O_3+H]^+$	245.1608	245.1608 (ESI pos.)
(DL)-Methioninamide (**2**$_M$**'**)	120	63	22	$[C_9H_{17}N_3O_3+Na]^+$	270.0883	270.0887 (ESI pos.)
L-Phenylalaninamide (**2**$_F$**'**)	125	73	23	$[C_{13}H_{17}N_3O_3+H]^+$	264.1343	164.1341 (ESI pos.)
L-Prolinamide (**2**$_P$**'**)	144	85	6	$[C_9H_{15}N_3O_3+H]^+$	214.1186	214.1185 (ESI pos.)
L-Serinamide[a] (**2**$_S$**'**)	36	74		$[C_7H_{13}N_3O_4+H]^+$	204.0979	204.0981 (ESI pos.)
L-Threoninamide[a] (**2**$_T$**'**)	36	85		$[C_8H_{15}N_3O_4+H]^+$	218.1135	218.1134 (ESI pos.)
L-Tryptophan amide (**2**$_W$**'**)	144	82	14	$[C_{15}H_{18}N_4O_3-H]^-$	301.1306	301.1306 (ESI neg.)
L-Valinamide (**2**$_V$**'**)	132	90	10	$[C_9H_{17}N_3O_3+H]^+$	216.1343	216.1342 (ESI pos.)
L-Tyrosine amide (**2**$_Y$**'**)	97	85	10	$[C_{13}H_{17}N_3O_4-H]^-$	278.1146	278.1147(ESI neg.)

Table S20: Yields and ESI-HRMS data for 3-mercaptopropanoic acid (**6b**, 60 mM) ▲ catalyzed formation of peptide **5'** ▼ from the coupling of *N*-acetyl glycine nitrile (**1**, 200 mM) with aminoamide ■ (**2'**, 2.0 equiv.) at pH 8.5, room temperature, unless stated otherwise. Two equiv. of amino amide were used due to the low pKa of these compounds.

[a] pH 7, 60 °C

*Coupling of N-acetylglycine nitrile **1** with L-alaninamide **2$_A$'** at pH 8.5 and room temperature*

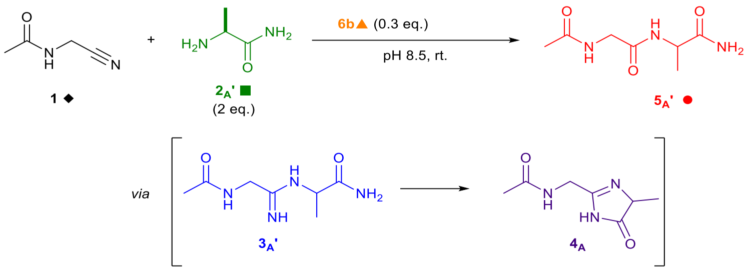

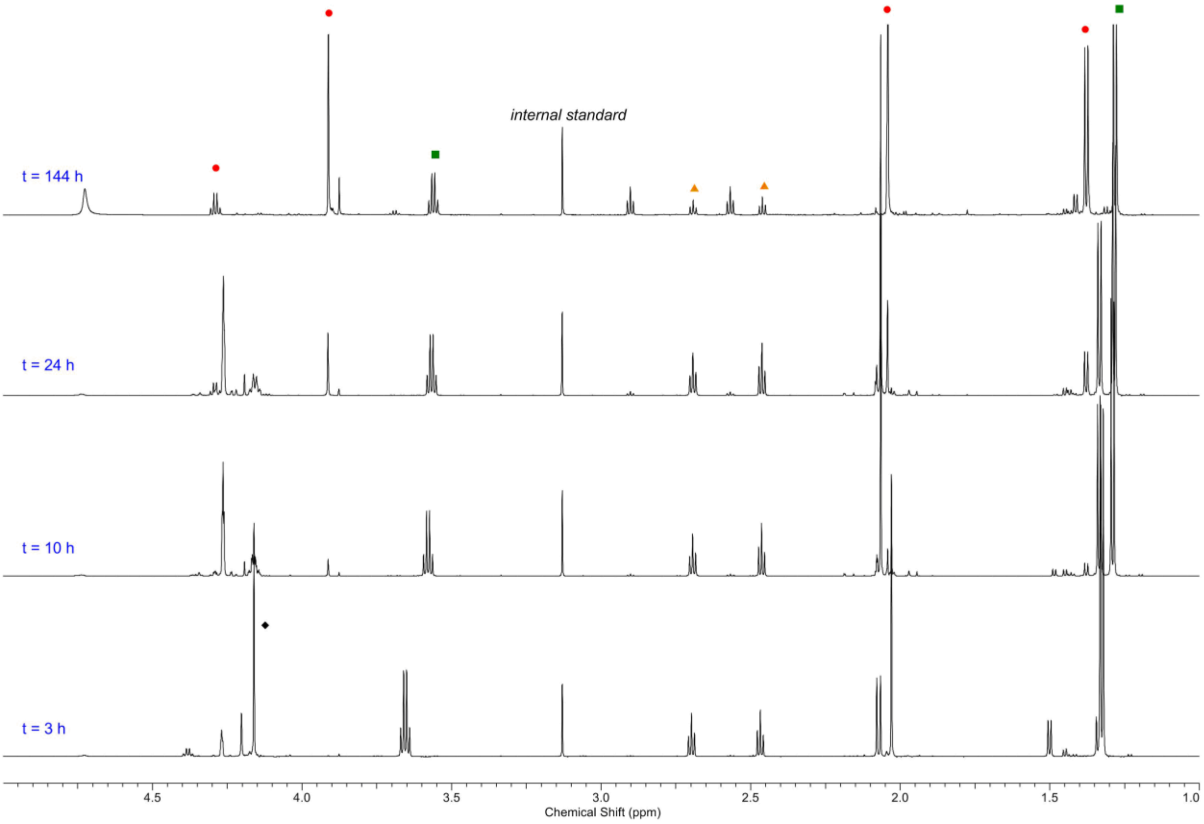

Figure S54: ^{1}H NMR (700 MHz, H_2O/D_2O 9:1, noesygppr1d, 1.0-5.0 ppm) spectrum to show the reaction of N-acetyl glycine nitrile (**1**, 200 mM) with L-alaninamide (**2$_A$'**, 400 mM) and 3-mercaptopropanoic acid (**6b**, 60 mM), with MSM (7 mM) as an internal standard, at pH 8.5, room temperature.

^{1}H NMR (700 MHz, H_2O/D_2O 9:1) **5$_A$'** (●): δ_H 4.29 (1H, q, *J* = 7.3 Hz, C***H***(CH$_3$)) 3.91 (2H, s, C***H$_2$***CONH)), 2.04 (3H, s, ***H$_3$***C(CO)), 1.37 (3H, d, *J* = 7.3 Hz, CH(C*H$_3$*)); *L-alaninamide*, **2$_A$'** (■): δ_H 3.56 (1H, q, *J* = 7.2 Hz, C*H*(CH$_3$)), 1.29 (3H, d, *J* = 7.2 Hz, CH(C*H$_3$*)); *N-acetylglycinamide*, **7** (✖) (partial assignment): δ_H 3.89 (2H, d, C*H$_2$*).

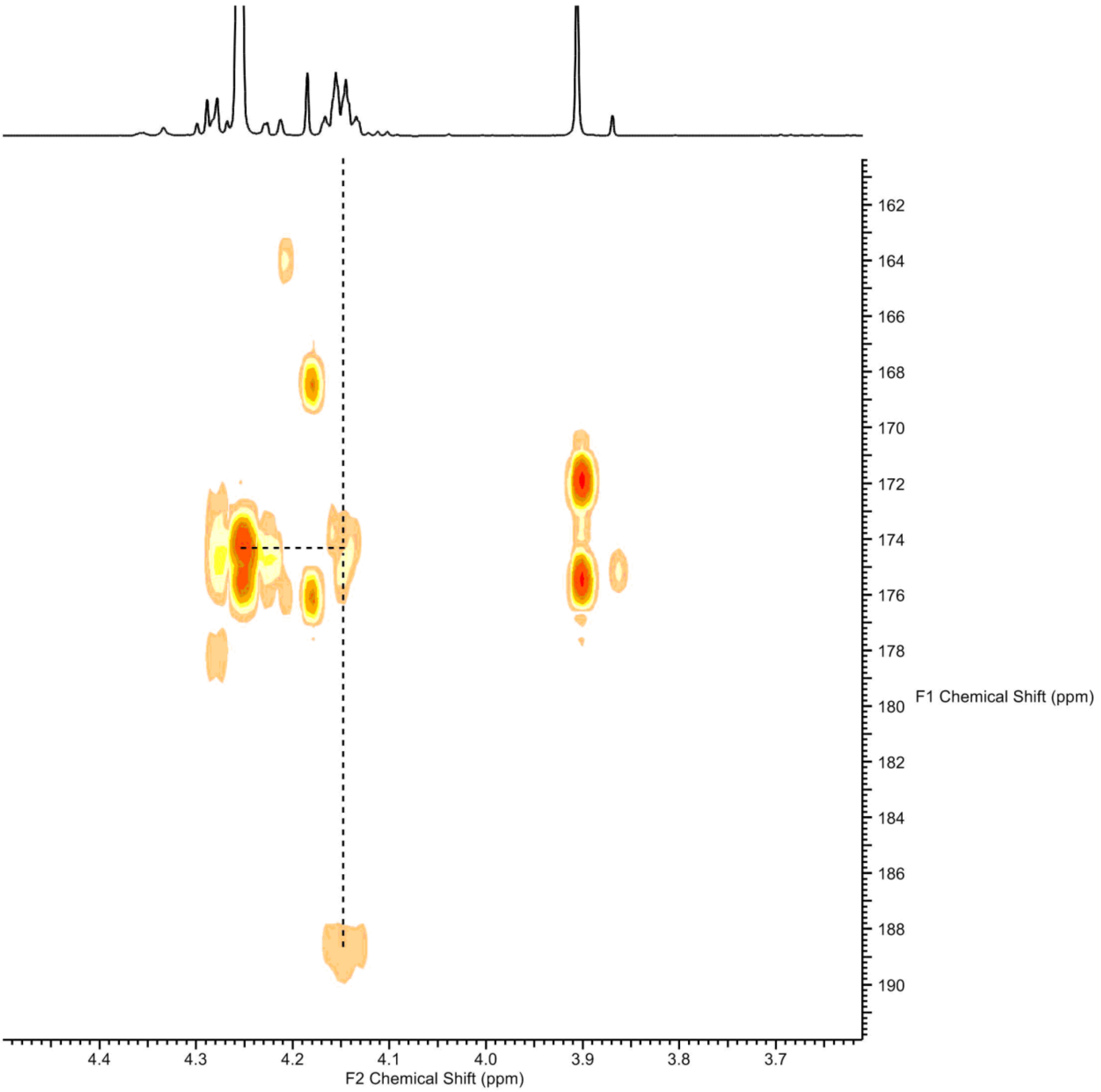

Figure S55: ^{1}H–^{13}C HMBC (^{1}H: 700 MHz [3.6-4.5 ppm], ^{13}C: 176 MHz [160-192 ppm], H_2O/D_2O 9:1) spectrum for the coupling of **1** and **2_A'** catalyzed by 3-mercaptopropionic acid (**6b**) at pH 8.5 after 24 h, showing the diagnostic $^{2}J_{CH}$ and $^{3}J_{CH}$ coupling of **Ala**-αH in **4_A** at 4.15 ppm with two resonances at 174.5 and 188.7 ppm, which is characteristic of imidazolone formation.

*Coupling of N-acetylglycine nitrile **1** with L-aspartic acid amide **2$_D$'** at pH 8.5 and room temperature*

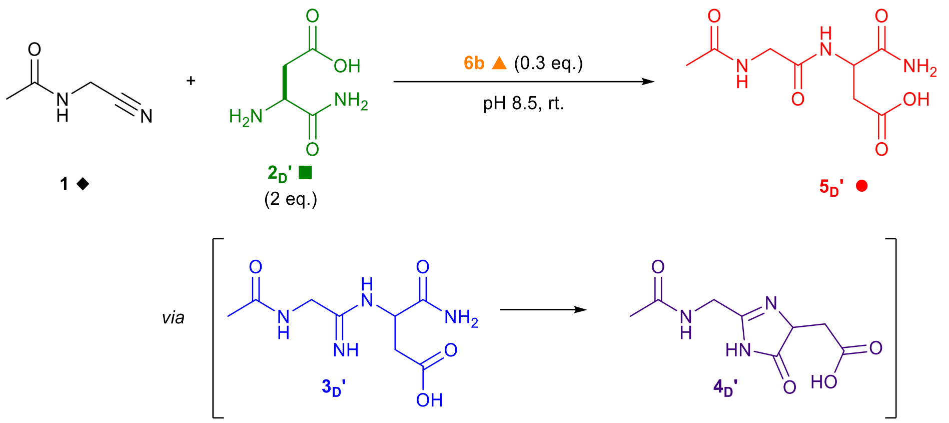

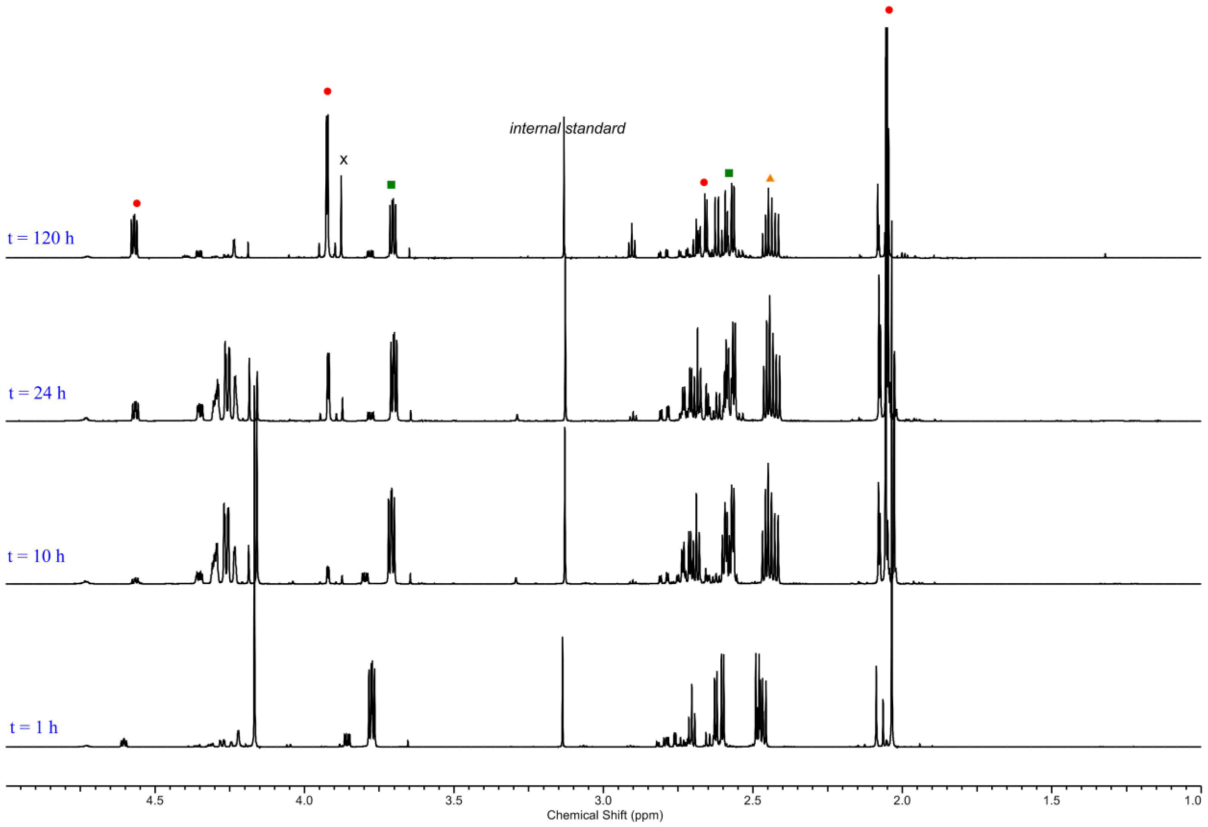

Figure S56: ^{1}H NMR (700 MHz, H_2O/D_2O 9:1, noesygppr1d, 1.0-5.0 ppm) spectrum to show the reaction of *N*-acetyl glycine nitrile (**1**, 200 mM) with L-aspartic acid amide (**2$_D$'**, 260 mM) and 3-mercaptopropanoic acid (**6b**, 60 mM) with MSM (7 mM) as an internal standard after 24 h at pH 8.5 and room temperature.

^{1}H NMR (700 MHz, H_2O/D_2O 9:1, noesygppr1d) **5$_D$'** (●): δ_H 4.57 (1H, dd, *J* = 7.8, 5.2 Hz, Asp-α***H***-CONH$_2$), 3.94 (1H, *J* = 17.1 Hz, AcNHC***H***H), 3.90 (1H, *J* = 17.1 Hz, AcNHCH***H***), 2.67 (ABX, *J* = 15.9, 5.2 Hz, CH(CH***H***COOH)CONH$_2$), 2.61 (ABX, *J* = 15.9, 7.8 Hz, CH(CH***H***COOH)CONH$_2$), 2.05 (3H, s, ***H***$_3$C(CO)); *L-aspartic acid amide*, **2$_D$'** (■): δ_H 3.70 (1H, dd, *J* = 8.0, 5.5 Hz, ***αH***-COOH), 2.60 (1H, ABX, *J* = 15.8, 5.5 Hz, CH(CH***H***COOH)CONH$_2$), 2.46 (1H, ABX, *J* = 15.8, 8.0 Hz, CH(CH***H***COOH)CO NH$_2$); *N-acetylglycinamide*, **7** (✖): δ_H 3.90 (2H, s, C*H*$_2$).

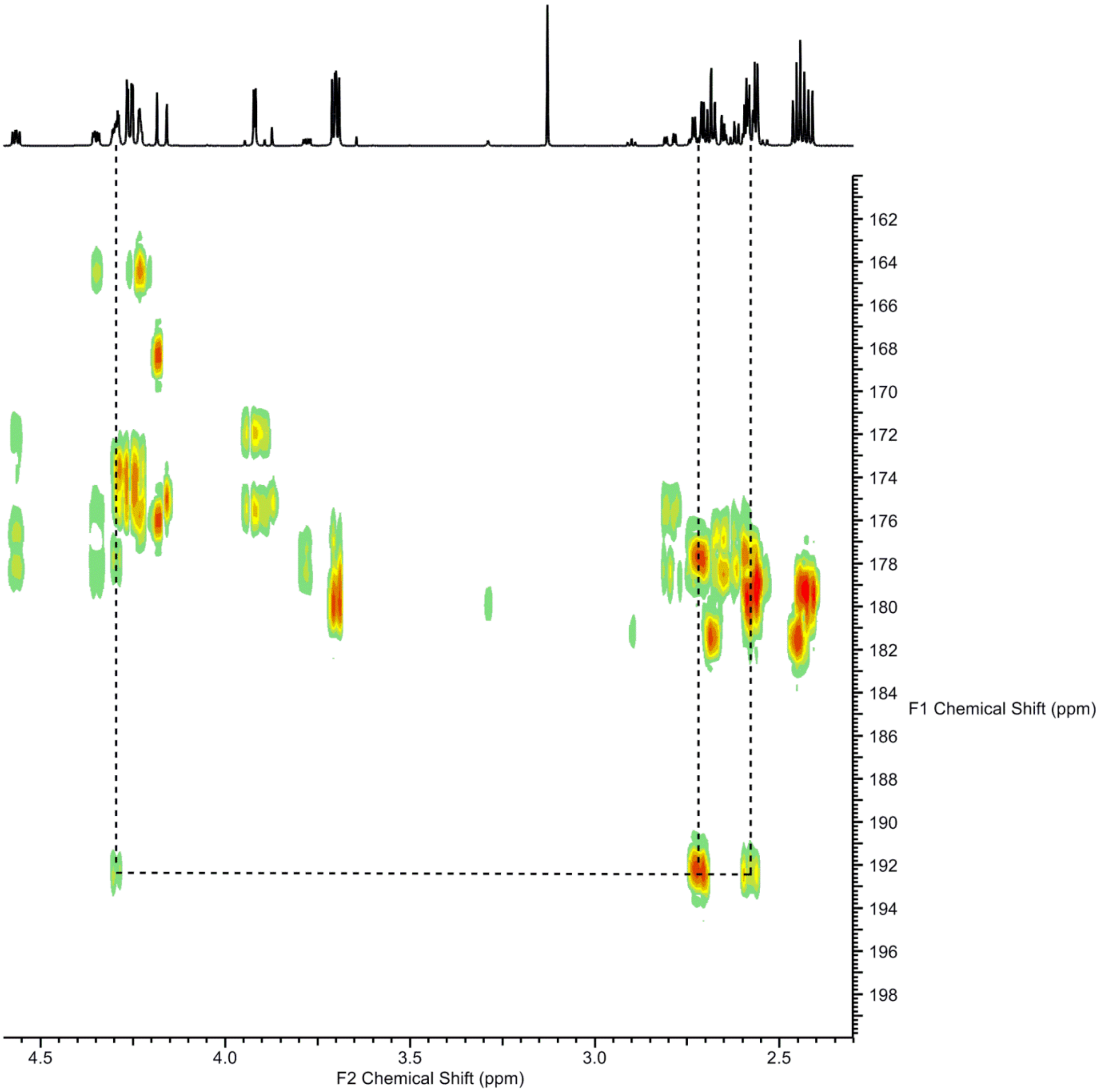

Figure S57: ^1H–^{13}C HMBC (^1H: 700 MHz [2.4-4.5 ppm], ^{13}C: 176 MHz [160-200 ppm], H_2O/D_2O 9:1) spectrum for the coupling of **1** and **2**$_D$**'** catalyzed by 3-mercaptopropionic acid (**6b**) at pH 8.5 after 24 h, showing the diagnostic coupling of the imidazolone amide signal in **4**$_D$ at 192.2 ppm with signals at 4.30 ppm (**Asp**-αH, $^2J_{CH}$), 2.72 and 2.58 ppm (**Asp**-βH, $^3J_{CH}$), which is characteristic of imidazolone formation.

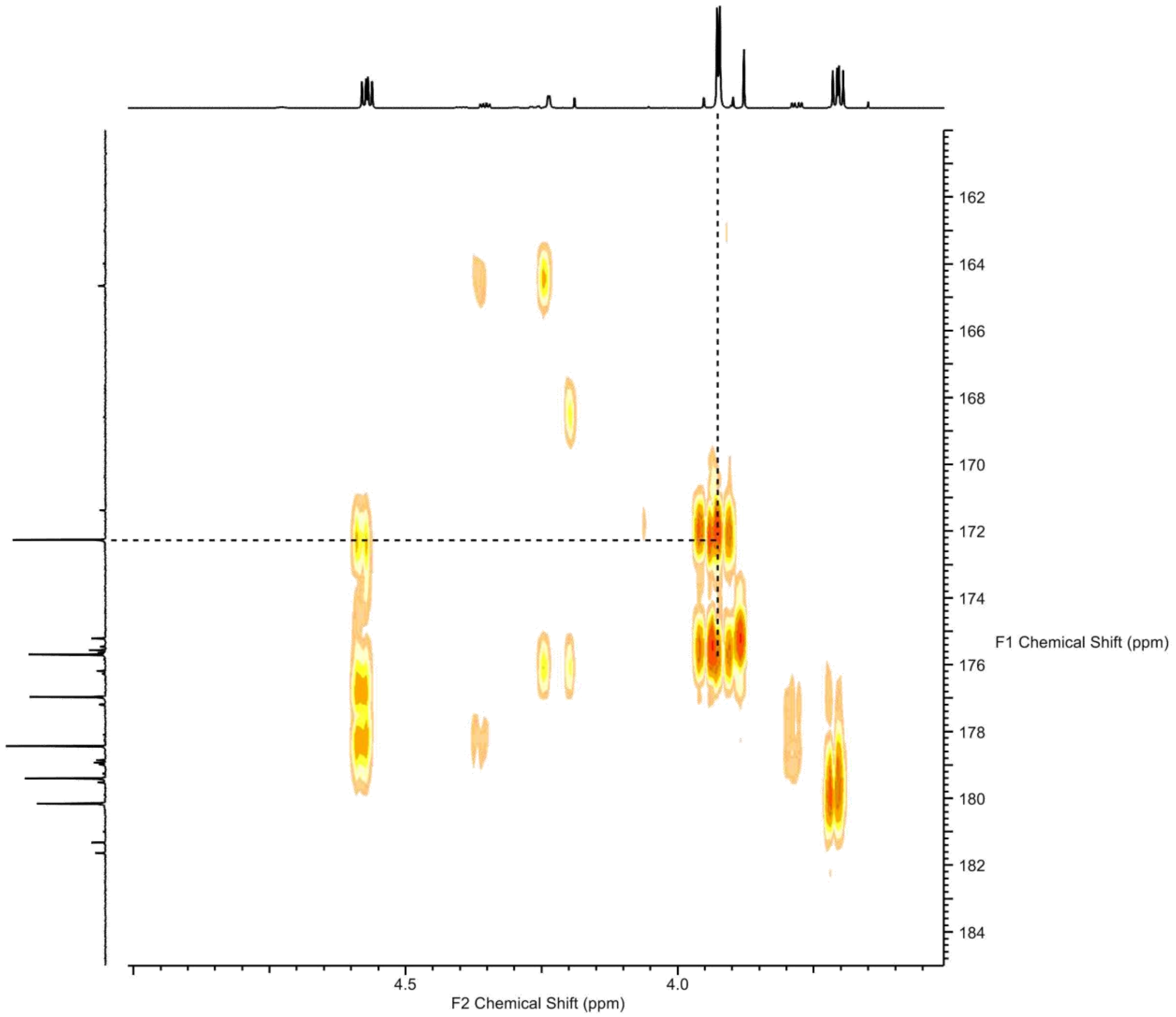

Figure S58: ^{1}H–^{13}C HMBC (^{1}H: 700 MHz [3.5-5.0 ppm], ^{13}C: 176 MHz [160-185 ppm], H_2O/D_2O 9:1) spectrum showing the diagnostic $^2J_{CH}$ and $^3J_{CH}$ coupling of the **Asp**-αH-CONH$_2$ in **5$_D$'** at 4.57 ppm with three resonances at 178.4, 177.0 and 172.3 ppm, characteristic of peptide formation.

*Coupling of N-acetylglycine nitrile **1** with L-arginine amide **2***

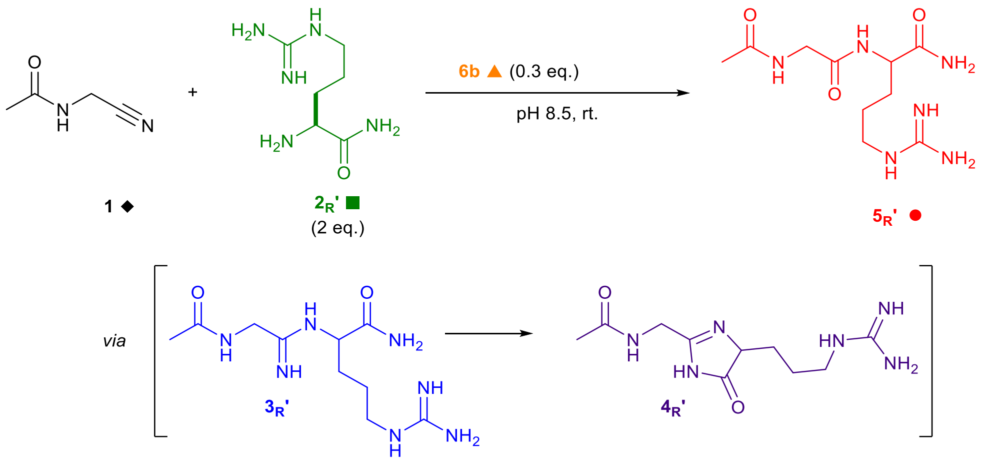

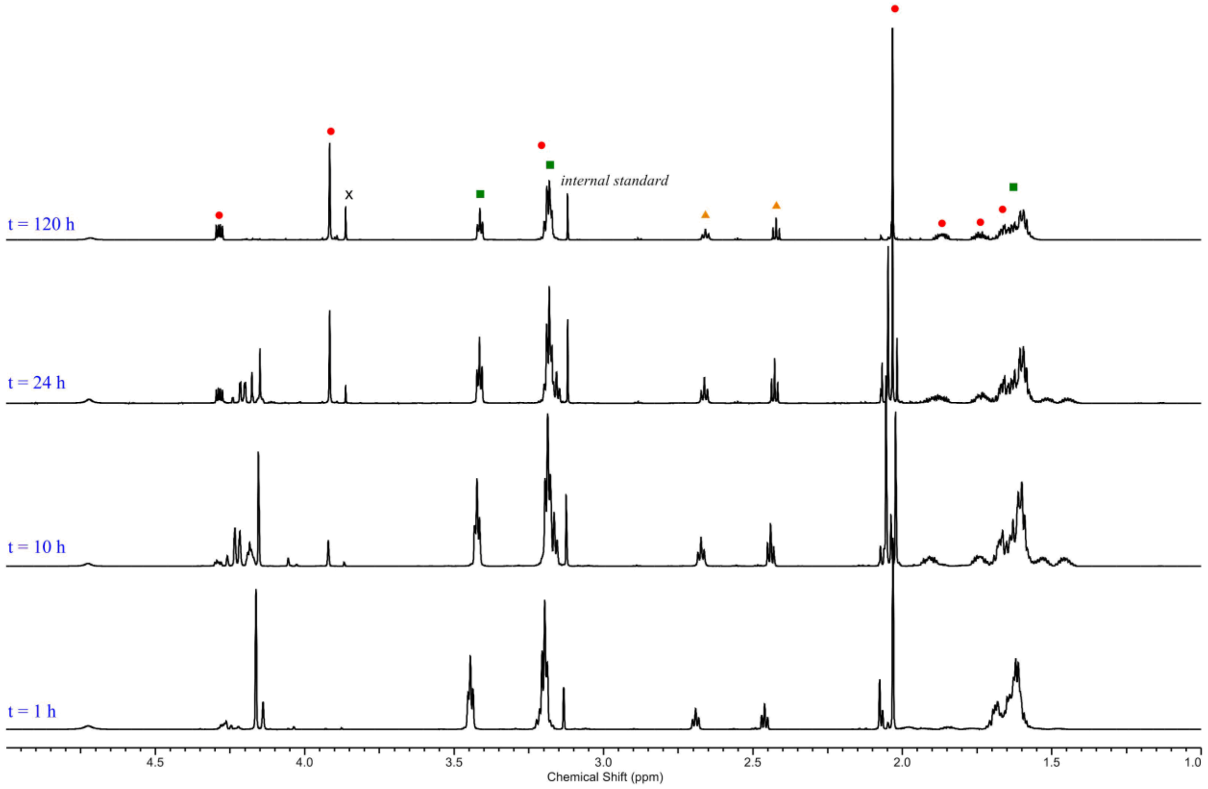

Figure S59: ^{1}H NMR (700 MHz, H_2O/D_2O 9:1, noesygppr1d, 1.0-5.0 ppm) spectrum to show the reaction of N-acetyl glycine nitrile (**1**, 200 mM) with L-arginine amide (**2_R'**, 400 mM) 3-mercaptopropanoic acid (**6b**, 60 mM) with MSM (30 mM) as an internal standard at pH 8.5 and room temperature. ✖ = N-acetylglycinamide, **7**.

^{1}H NMR (700 MHz, H_2O/D_2O 9:1, noesygppr1d) *(2-acetamido-1-iminoethyl)arginine*, **5_R'** (●): δ_H 4.32 (1H, dd, J = 9.3, 5.1 Hz, Arg-***αH***-COOH), 3.95 (1H, app. s AcNHC***H₂***), 3.22 (overlapped, C*H*$_2$(guanidyl)), 2.07 (3H, s, ***H***$_3$C(CO)), 1.90 (1H, m, C**H**HCH$_2$CH$_2$(guanidyl)), 1.77 (1H, m, CH***H***CH$_2$CH$_2$(guanidyl)), 1.57 (overlap, CH$_2$C*H$_2$*CH$_2$(guanidyl)); *L-arginine amide*, **2_R'** (■) (partial assignment): δ_H 3.45 (1H, t, J = 6.1 Hz, α***H***-COOH), 3.22 (overlap C*H*$_2$(guanidyl)); *N-Acetylglycinamide*, **7** (✖) (partial assignment): δ_H 3.90 (2H, s, C*H*$_2$).

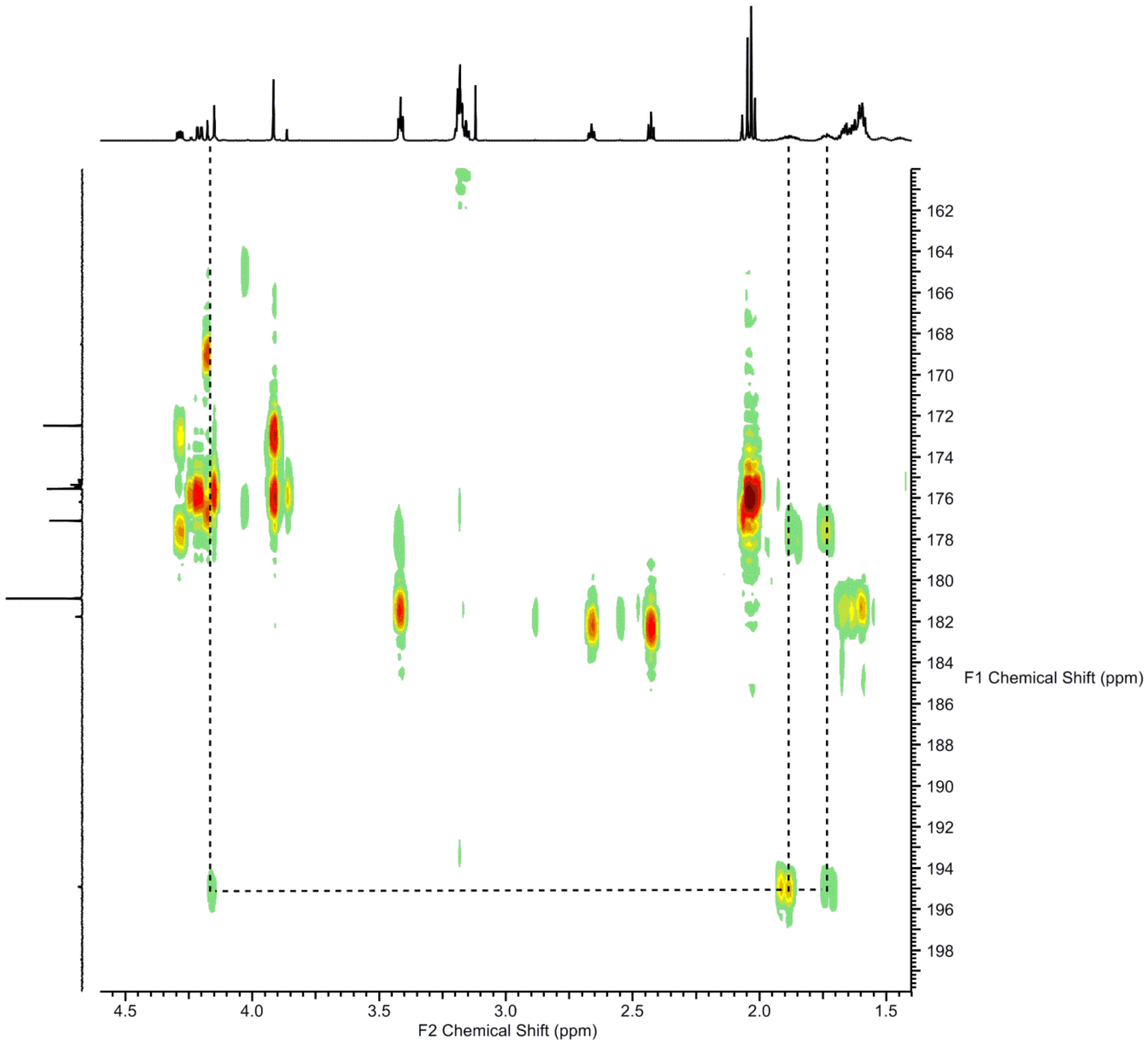

Figure S60: ^{1}H–^{13}C HMBC (^{1}H: 700 MHz [1.4-4.6 ppm], ^{13}C: 176 MHz [160-200 ppm], H_2O/D_2O 9:1) spectrum for the coupling of **1** and **2R'** catalyzed by 3-mercaptopropionic acid (**6b**) at pH 8.5 after 24 h, showing the diagnostic coupling of the imidazolone amide signal in **4R** at 194.8 ppm with signals at 4.17 ppm (**Arg**-αH, $^{2}J_{CH}$), 1.90 and 1.72 ppm (**Arg**-βH, $^{3}J_{CH}$), which is characteristic of imidazolone formation.

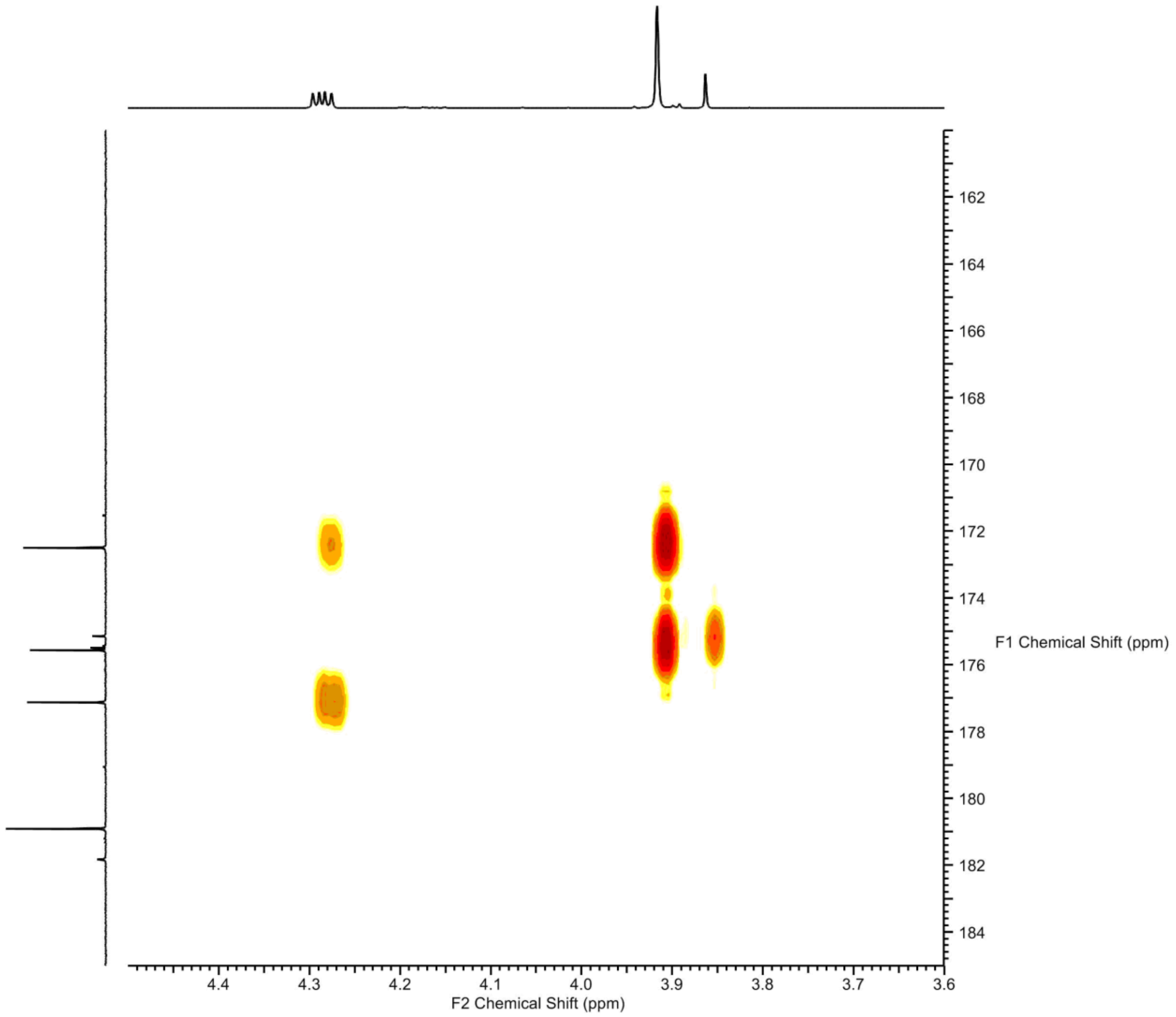

Figure S61: ^1H–^{13}C HMBC (^1H: 700 MHz [3.6-4.5 ppm], ^{13}C: 176 MHz [160-185 ppm], H_2O/D_2O 9:1) spectrum showing the diagnostic $^2J_{CH}$ and $^3J_{CH}$ coupling of **Arg**-αH in **5_R'** at 4.32 ppm with two resonances at 177.1 and 172.5 ppm.

*Coupling of N-acetylglycine nitrile **1** with L-asparagine amide **2$_N$'** at pH 8.5 and room temperature*

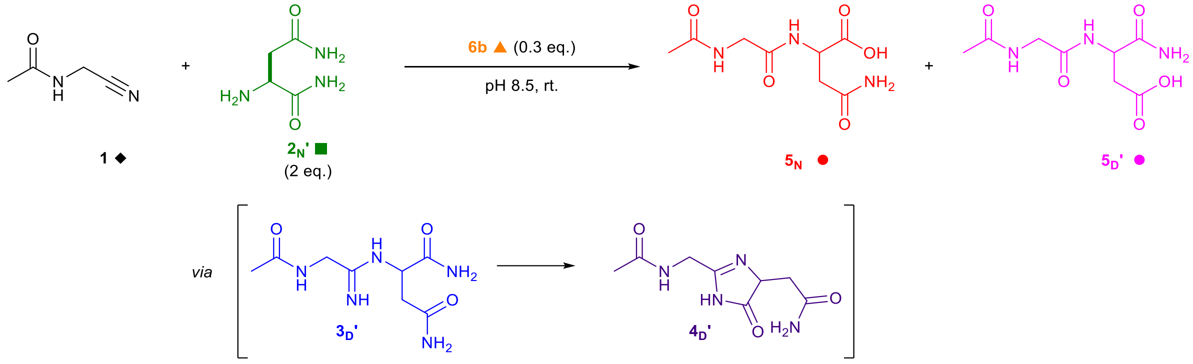

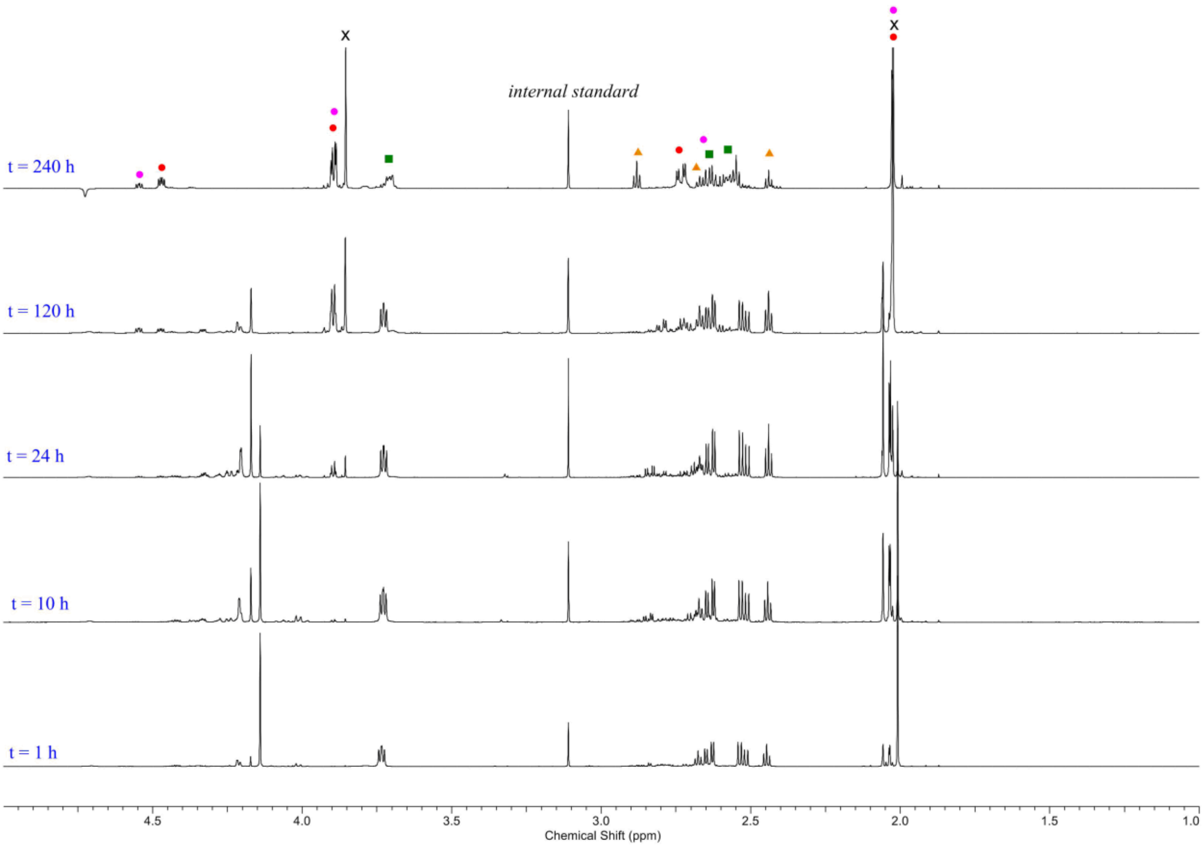

Figure S62: ^{1}H NMR (700 MHz, H_2O/D_2O 9:1, noesygppr1d, 1.0–5.0 ppm) spectrum to show the reaction of *N*-acetyl glycine nitrile (**1**, 200 mM) with L-asparagine amide (**2$_N$'**, 400 mM) and 3-mercaptopropanoic acid (6b, 60 mM) with MSM (7 mM) as an internal standard at pH 8.5 and room temperature. ✖ = N-acetyl glycinamide **7**

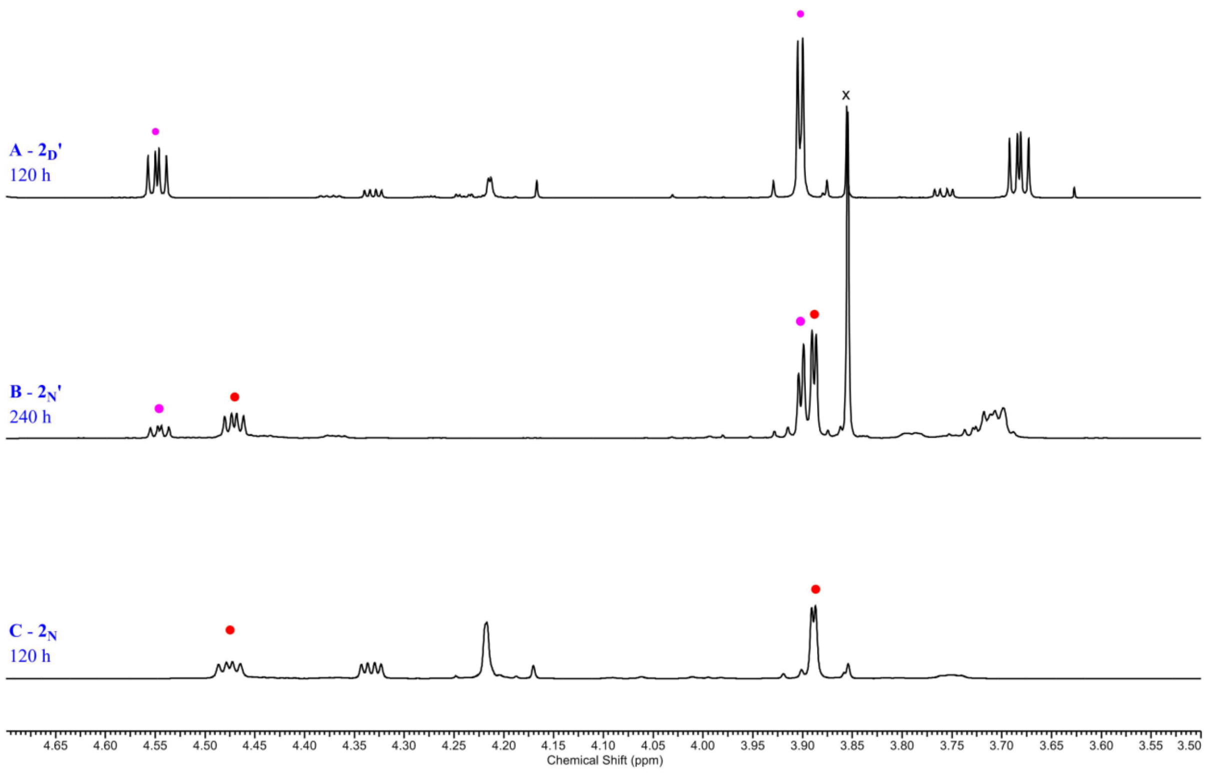

Figure S63: ^{1}H NMR (700 MHz, H_2O/D_2O 9:1, noesygppr1d, 3.5–4.7 ppm) spectrum to show the reaction of *N*-acetyl glycine nitrile (**1**, 200 mM) at pH 8.5 and room temperature with 3-mercaptopropanoic acid (**6b**, 60 mM) and **A** L-asparagine (**2_N**, 240 mM, 120 h), **B** L-asparagine amide (**2_N'**, 400 mM, 240 h) or **C** L-aspartic acid amide (**2_D'**, 400 mM, 120 h).

For characterisation see entries for **2_N** (p 33) and **2_D'** (p 66).

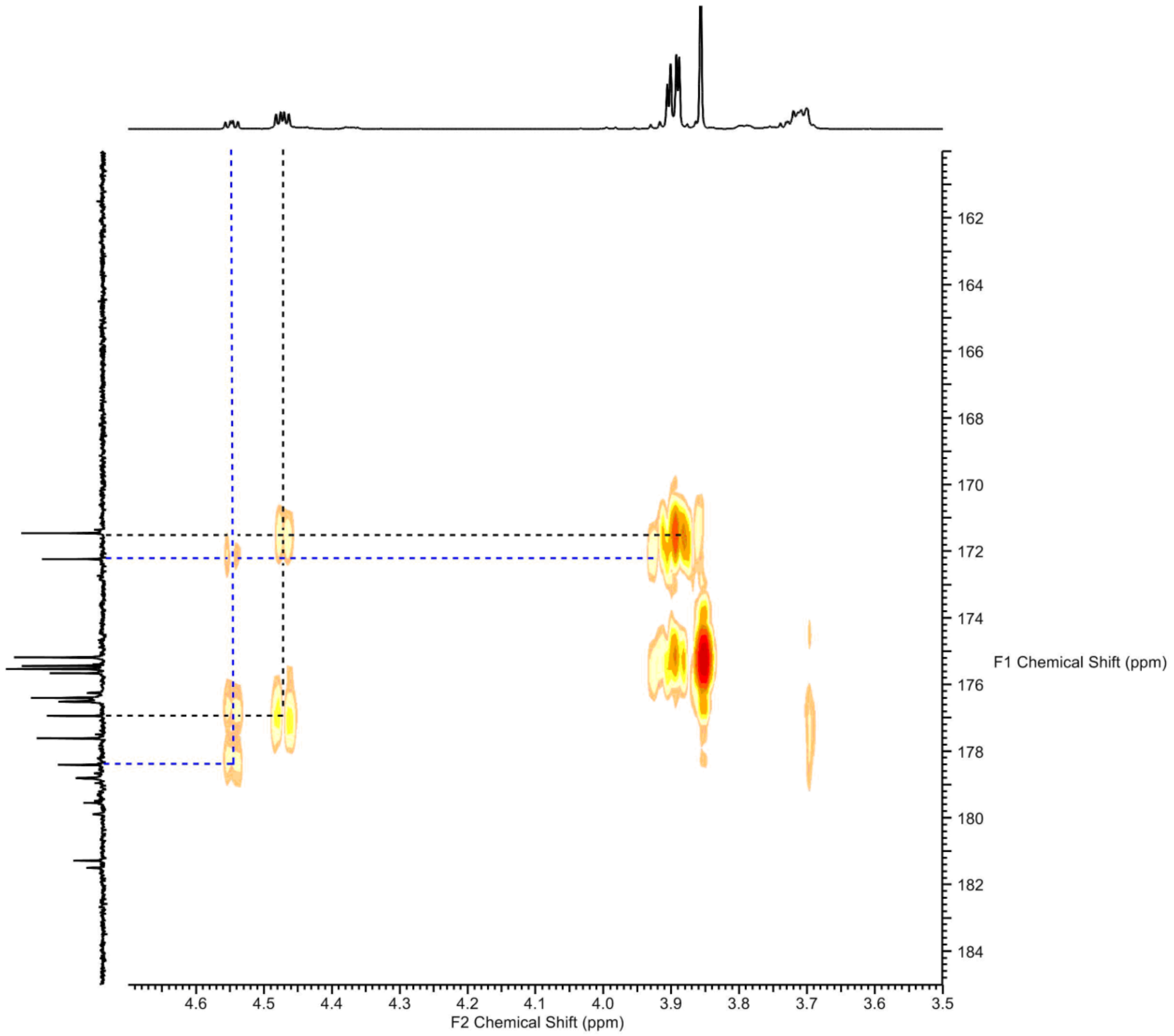

Figure S64: ^1H–^{13}C HMBC (^1H: 700 MHz [3.5-4.7 ppm], ^{13}C: 176 MHz [160-185 ppm], H_2O/D_2O 9:1) spectrum showing the diagnostic $^2J_{CH}$ and $^3J_{CH}$ coupling of the Asn-αH- COOH in **5$_N$** at 4.54 ppm with three resonances at 178.4, 176.9 and 172.2 ppm, which is characteristic of peptide bond formation of Asn, and the $^2J_{CH}$ and $^3J_{CH}$ coupling of Asp-αH-CONH$_2$ in **5$_D$'** at 4.46 ppm with two resonances at 176.9 and 171.5 ppm, which is characteristic of peptide bond formation of Asp.

*Coupling of N-acetylglycine nitrile **1** with L-glutamic acid amide **2_E'** at pH 8.5 and room temperature*

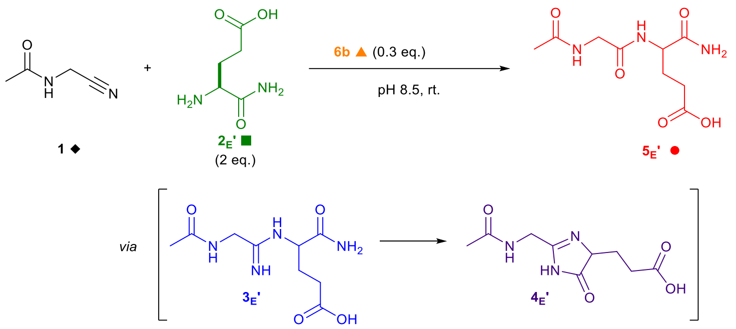

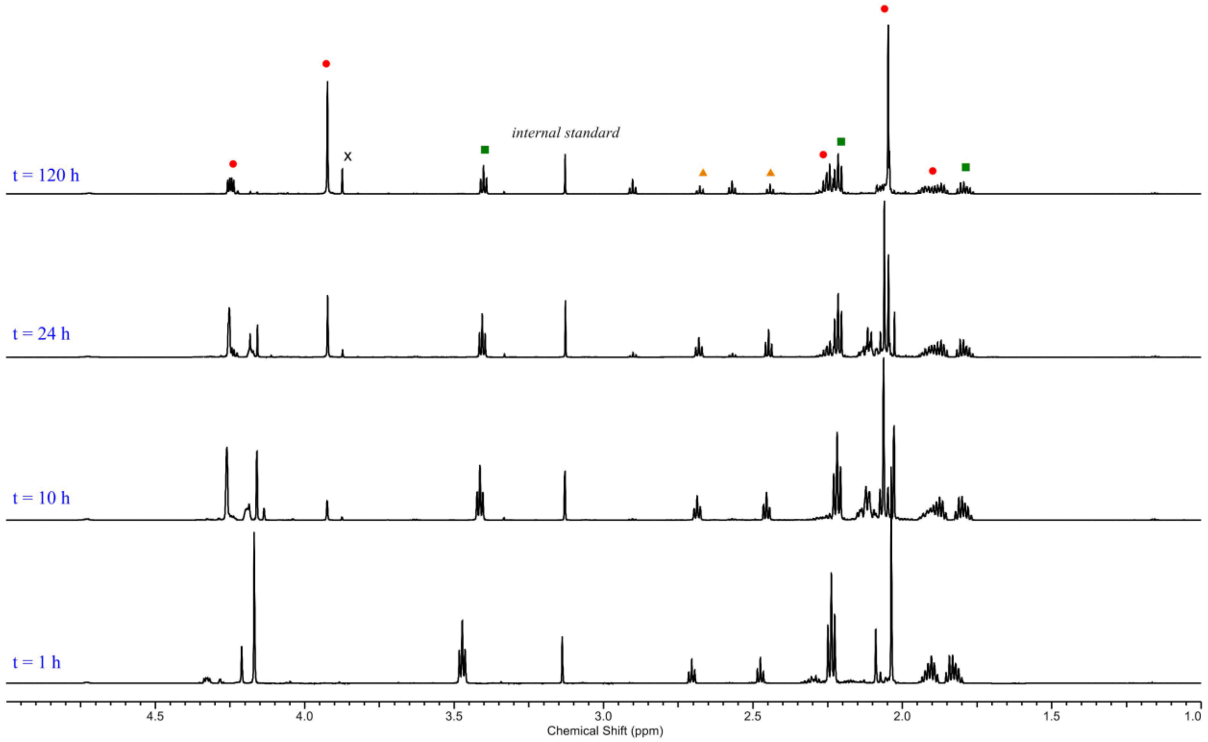

Figure S65: ^{1}H NMR (700 MHz, H_2O/D_2O 9:1 noesygppr1d, 1.0–5.0 ppm) spectrum to show the reaction of N-acetyl glycine nitrile (**1**, 200 mM) with L-glutamic acid amide (**2_E'**, 220 mM) and 3-mercaptopropanoic acid (**6b**, 60 mM) with MSM (7 mM) as an internal standard at pH 8.5 and room temperature.✖ = N-Acetyl glycinamide **7**

^{1}H NMR (700 MHz, H_2O/D_2O 9:1) **5_E'** (●): δ_H 4.27 (1H, dd, *J* = 9.2, 4.9 Hz, Glu-***αH***-CONH$_2$), 3.95 (2H, s, AcNHC*H*$_2$), 2.29-2.23 (m, CH$_2$C***H***$_2$COOH), 2.08 (3H, s, *H*$_3$C(CO)), 2.10-2.06 (m, C***H***HCH$_2$COOH), 1.98-1.91 (m, CH***H***CH$_2$COOH); *L-glutamic acid amide*, 2_E' (■): δ_H 3.43 (1H, t, *J* = 6.72 Hz, ***αH***-CONH$_2$), 2.24 (2H, t, J = 8.0 Hz, CH$_2$C*H*$_2$COOH), 1.93-1.88 (m, CH***H***CH$_2$COOH), 1.84-1.79 (1H, m, C***H***HCH$_2$COOH); *N-acetylglycinamide*, **7** (✖) (partial assignment): δ_H 3.90 (2H, s, C*H*$_2$).

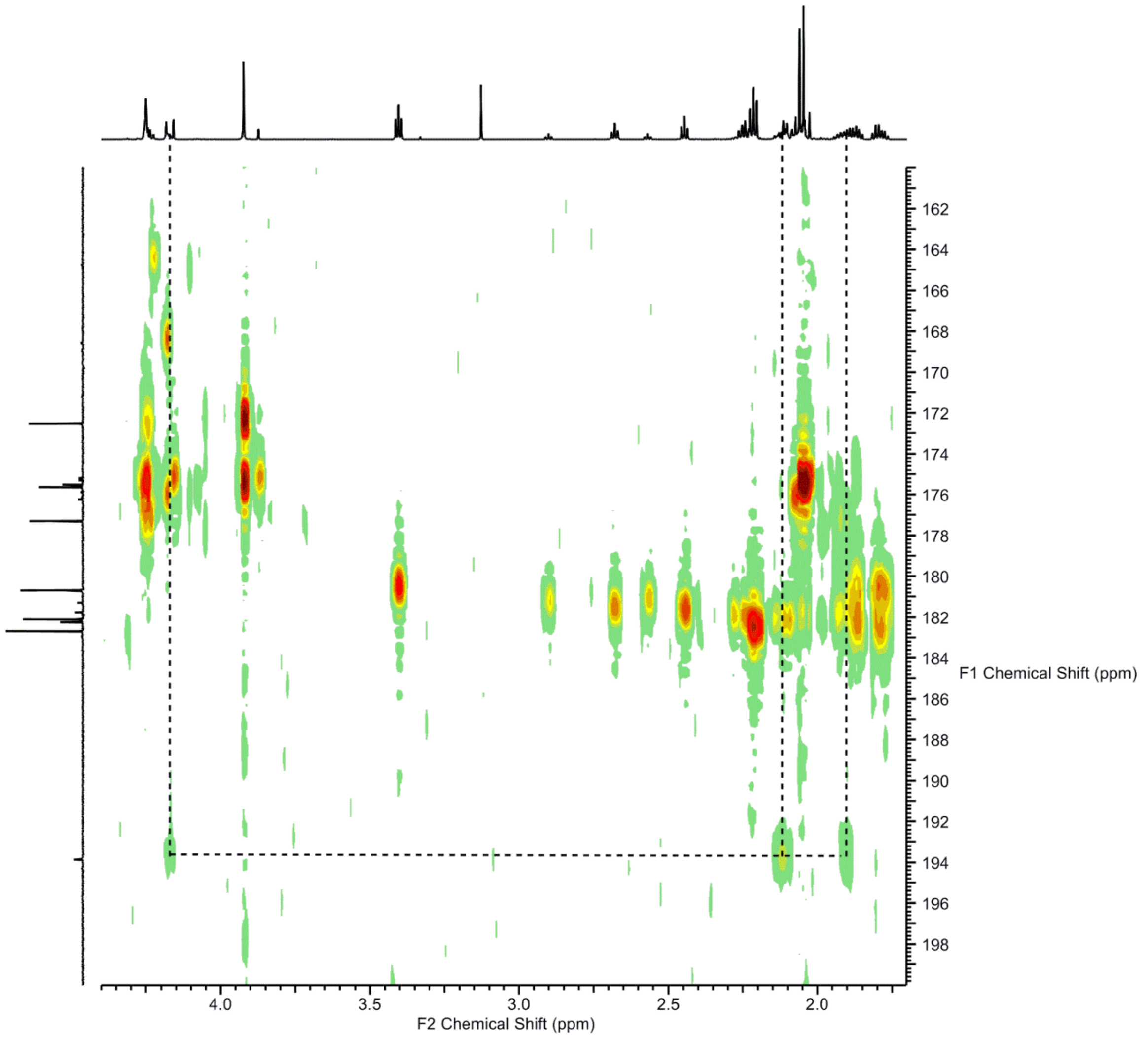

Figure S66: ^{1}H–^{13}C HMBC (^{1}H: 700 MHz [1.7-4.4 ppm], ^{13}C: 176 MHz [160-200 ppm], H_2O/D_2O 9:1) spectrum for the coupling of **1** and **2**$_{\mathbf{E}}$**'** catalyzed by 3-mercaptopropionic acid (**6b**) at pH 8.5 after 48 h, showing the diagnostic coupling of the imidazolone amide signal in **4**$_{\mathbf{E}}$ at 193.8 ppm with signals at 4.17 ppm (**Glu**-αH, $^{2}J_{CH}$), 2.12 and 1.90 ppm (**Glu**-βH, $^{3}J_{CH}$), which is characteristic of imidazolone formation.

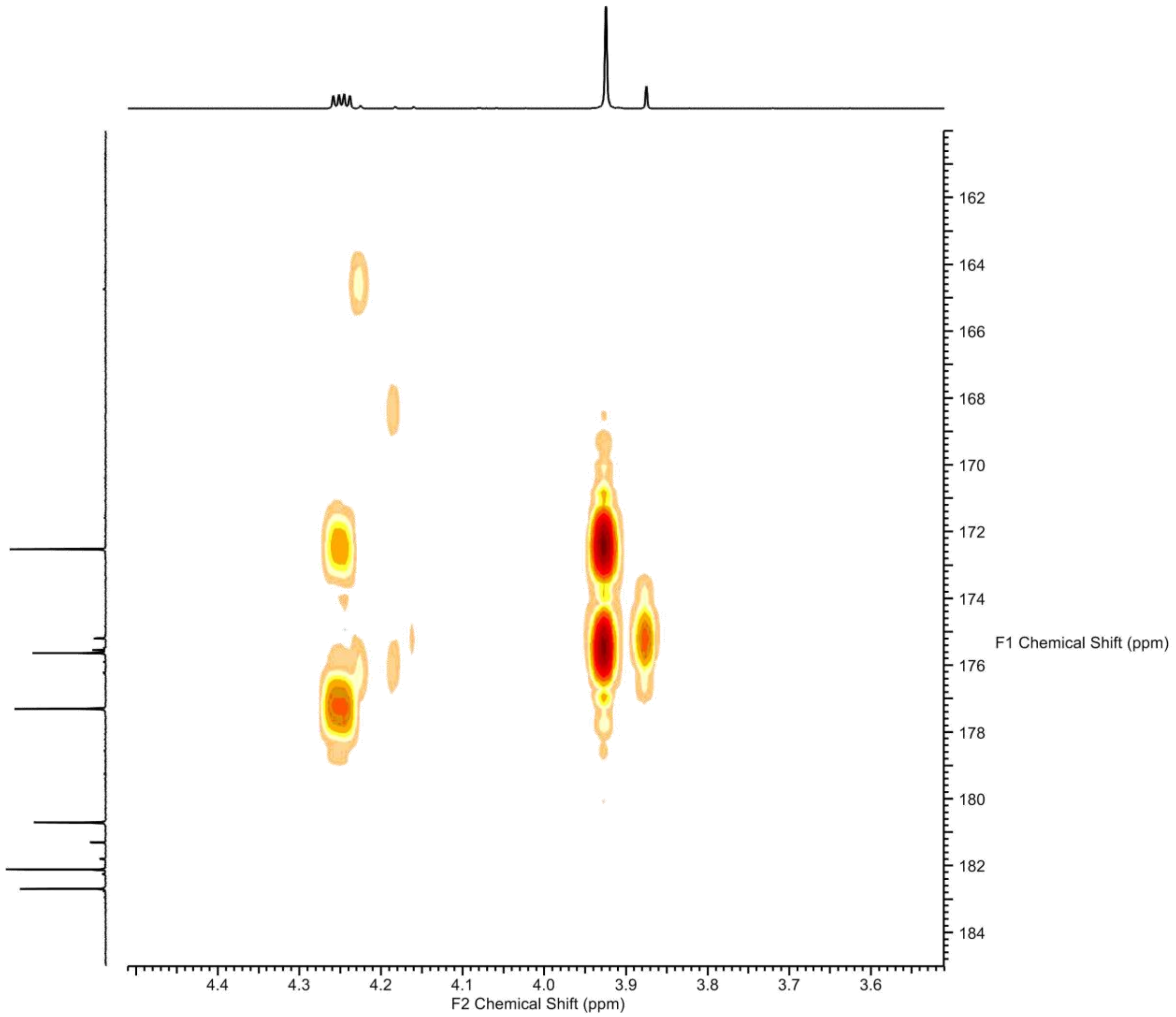

Figure S67: ^1H–^{13}C HMBC (^1H: 700 MHz [3.5-4.5 ppm], ^{13}C: 176 MHz [150-185 ppm], H_2O/D_2O 9:1) spectrum showing the diagnostic $^2J_{CH}$ and $^3J_{CH}$ coupling of the Glu-αH in 5_E' at 4.27 ppm with two resonances at 177.2 and 172.5 ppm.

*Coupling of N-acetylglycine nitrile **1** with L-glutamine amide **2$_Q$'** at pH 8.5 and room temperature*

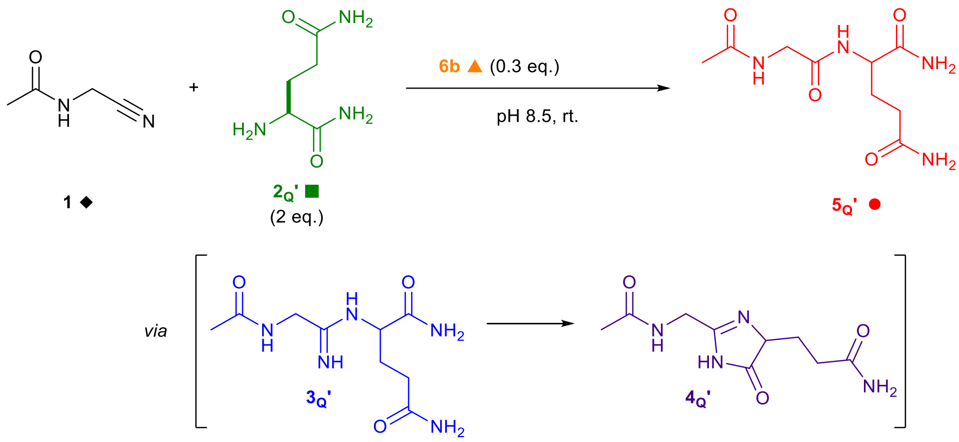

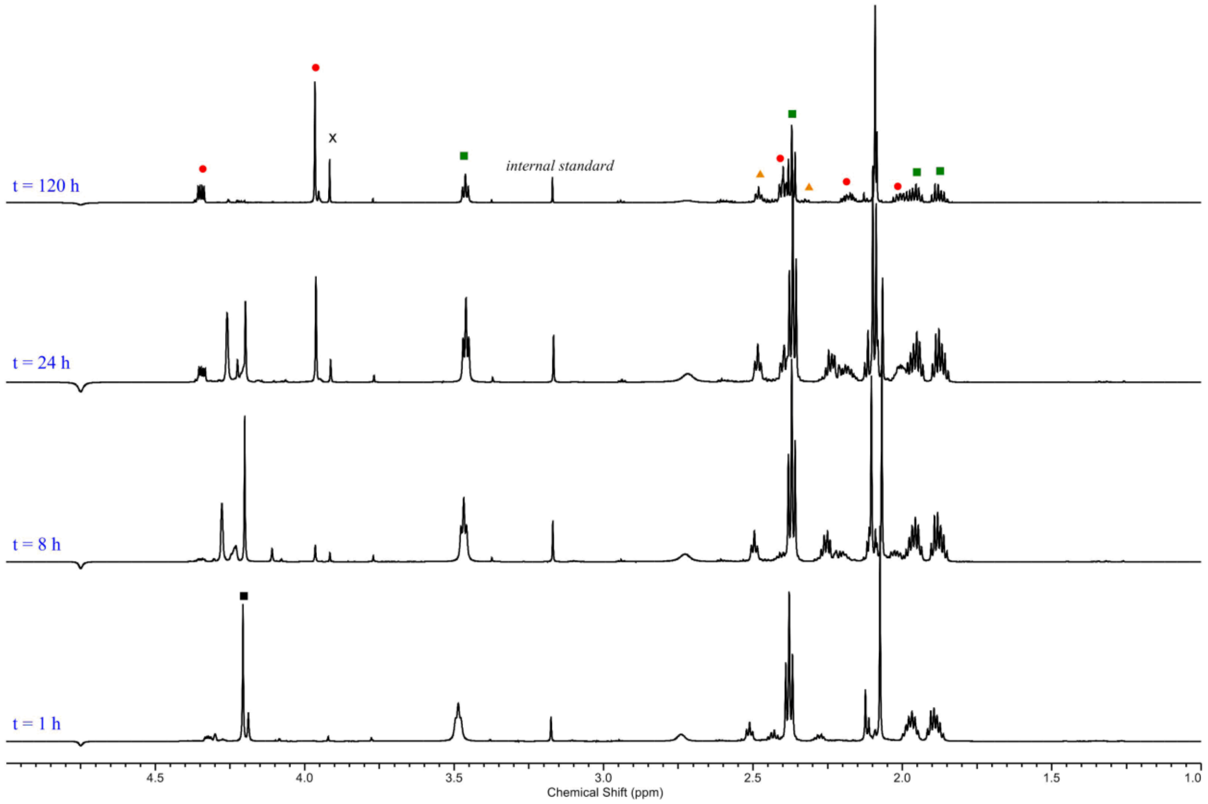

Figure S68: ^{1}H NMR (700 MHz, H_2O/D_2O 9:1, noesygppr1d, 1.0-5.0 ppm) spectrum to show the reaction of N-acetyl glycine nitrile (**1**, 200 mM) with L-glutamine amide (**2$_Q$'**, 400 mM) and 3-mercaptopropanoic acid (**6b**, 60 mM) with MSM (7 mM) as an internal standard at pH 8.5 and room temperature, ✖ = **7**.

^{1}H NMR (700 MHz, H_2O/D_2O 9:1, noesygppr1d) **5$_Q$'**, (●): δ_H 4.35 (1H, dd, *J* = 9.7, 4.7 Hz Gln-α*H*-COOH), 3.97 (2H, s, AcNHC*H*$_2$), 2.40 (2H, app. td, 7.4, 2.1 Hz C*H*$_2$CH$_2$CONH$_2$), 2.21 - 2.15 (1H, m, C***H***HCH$_2$CONH$_2$), 2.09 (3H, s, *H*$_3$C(CO)), 2.03 – 1.97 (1H, m, CH***H***CH$_2$CONH$_2$); *L-glutamine amide*, **2$_Q$'**, (■): δ_H 3.46 (1H, t, *J* = 6.6 Hz, ***αH***-COOH), 2.37 (2H, app t, *J* = 7.9 Hz, CH$_2$C***H$_2$***CONH$_2$), 1.99 – 1.93 (1H, m, C***H***HCH$_2$CONH$_2$), 1.90 – 1.83 (1H, m, CH***H***CH$_2$CONH$_2$); *N-acetylglycinamide*, **7** (✖) (partial assignment): δ_H 3.92 (2H, s, C*H*$_2$).

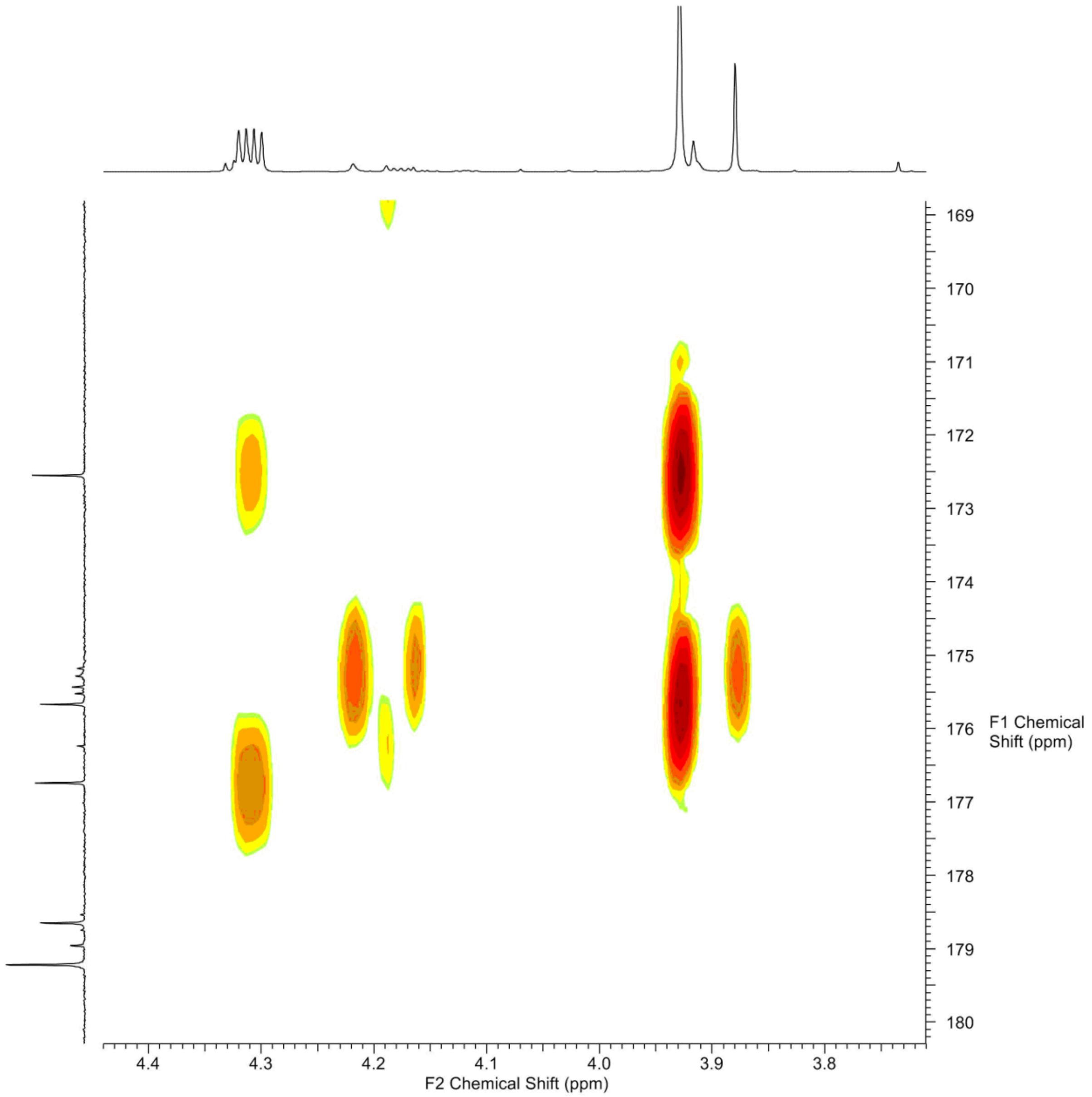

Figure S69: ^{1}H–^{13}C HMBC (^{1}H: 700 MHz [3.7-4.5 ppm], ^{13}C: 176 MHz [169-181 ppm], H_2O/D_2O 9:1) spectrum showing the diagnostic $^2J_{CH}$ and $^3J_{CH}$ coupling of the **Gln**-αH in **5$_Q$'** at 4.31 ppm with two resonances at 176.8 and 172.4 ppm, which is characteristic of amide bond formation of **Gln**.

*Coupling of N-acetylglycine nitrile **1** with glycinamide **2$_{G}$'** at pH 8.5 and room temperature*

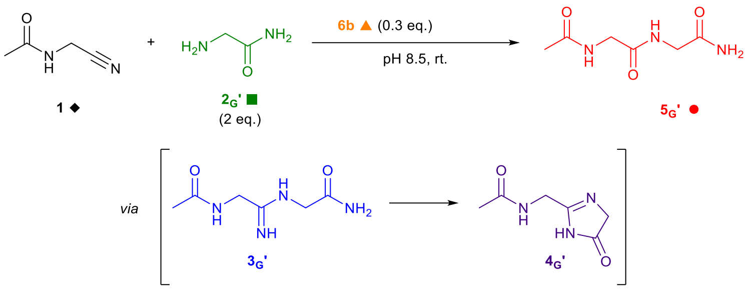

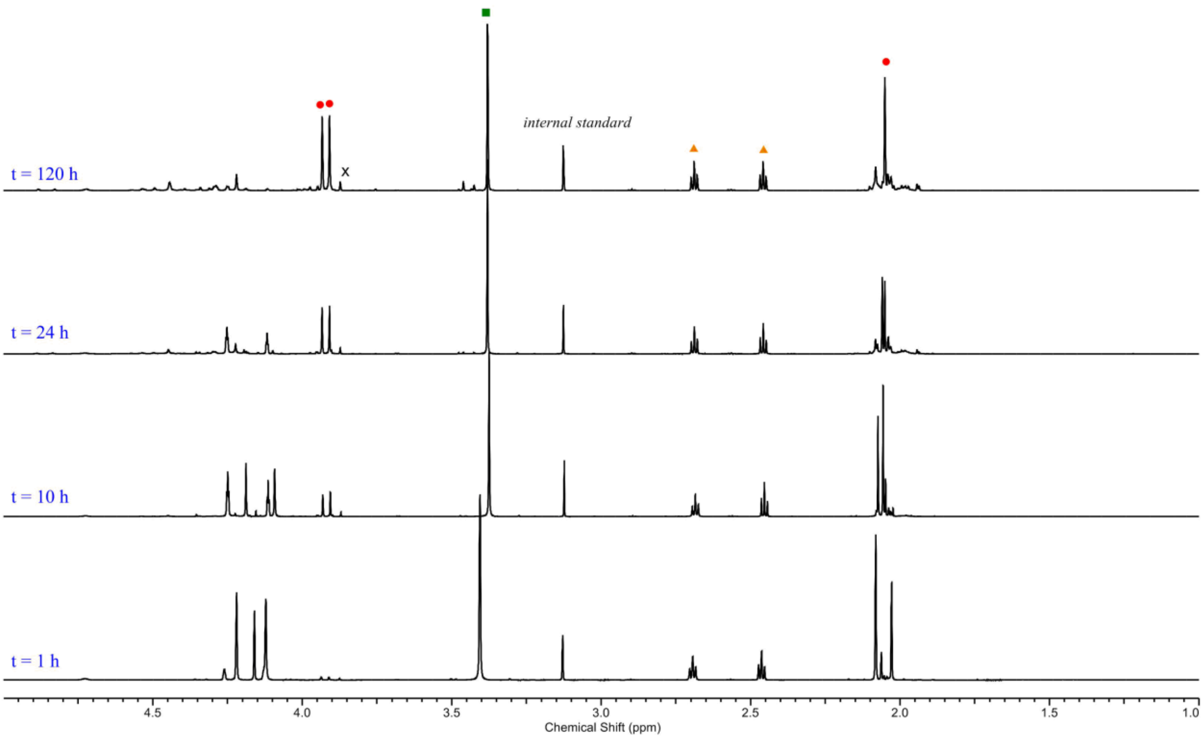

Figure S70: ^{1}H NMR (700 MHz, H_2O/D_2O 9:1, noesygppr1d, 1.0-5.0 ppm) spectrum to show the reaction of N-acetyl glycine nitrile (**1**, 200 mM) with glycinamide (**2$_{G}$'**, 400 mM) and 3-mercaptopropanoic acid (**6b**, 60 mM) with MSM (7 mM) as an internal standard at pH 9 and room temperature.

^{1}H NMR (700 MHz, H_2O/D_2O 9:1) **5$_{G}$'** (●): δ_H 3.96 (2H, s, AcNHC*H*$_2$), 3.94 (2H, s, C*H*$_2$CONH$_2$), 2.08 (3H, s, *H*$_3$C(CO)); *Glycinamide*, **2$_{G}$'** (■): δ_H 3.41 (2H, s, C*H*$_2$).

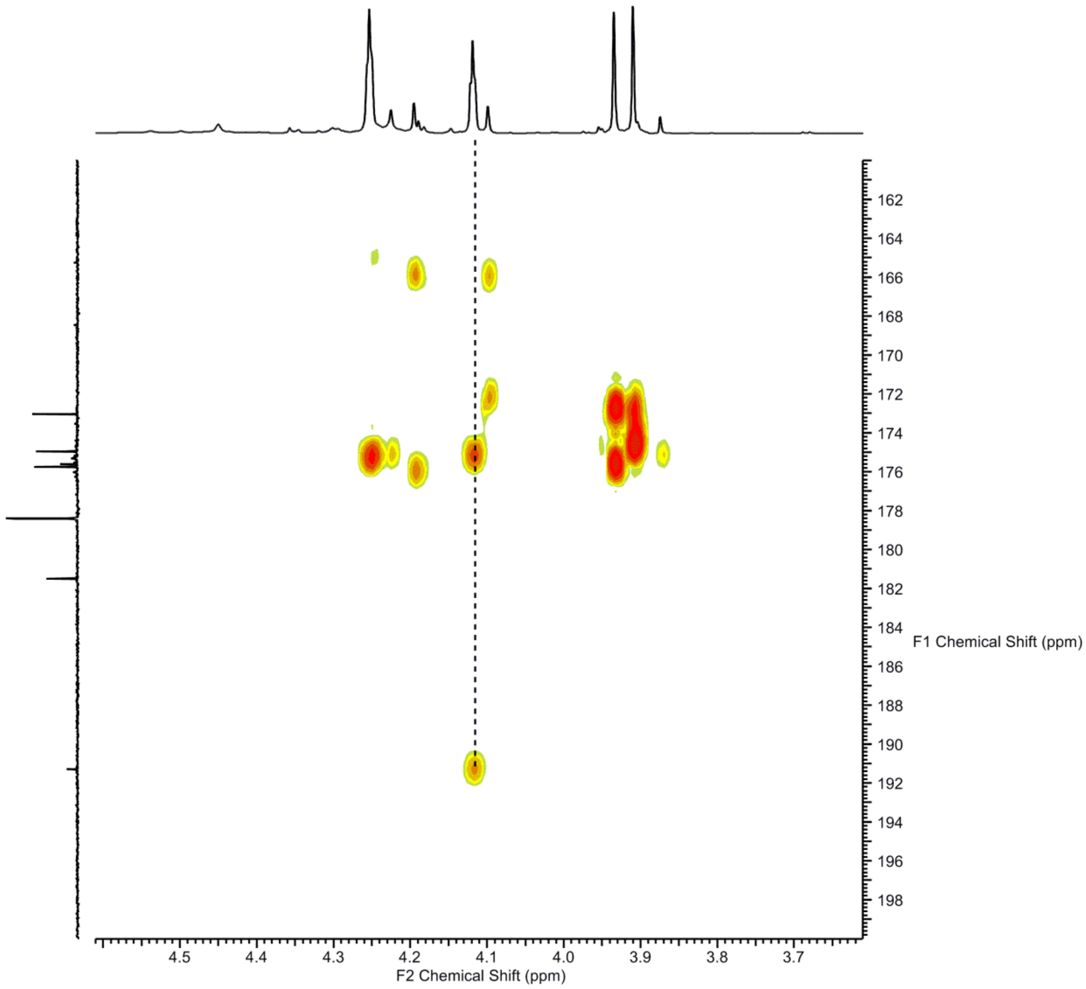

Figure S71: ^{1}H–^{13}C HMBC (^{1}H: 700 MHz [3.6-4.6 ppm], ^{13}C: 176 MHz [160-200 ppm], H_2O/D_2O 9:1) spectrum for the coupling of **1** and **2$_G$'** catalyzed by 3-mercaptopropionic acid (**6b**) at pH 8.5 after 24 h, showing the diagnostic coupling of the imidazolone amide signal in **4$_G$** at 191.3 ppm with signals at 4.12 ppm (**Gly**-αH, $^{2}J_{CH}$) which is characteristic of imidazolone formation.

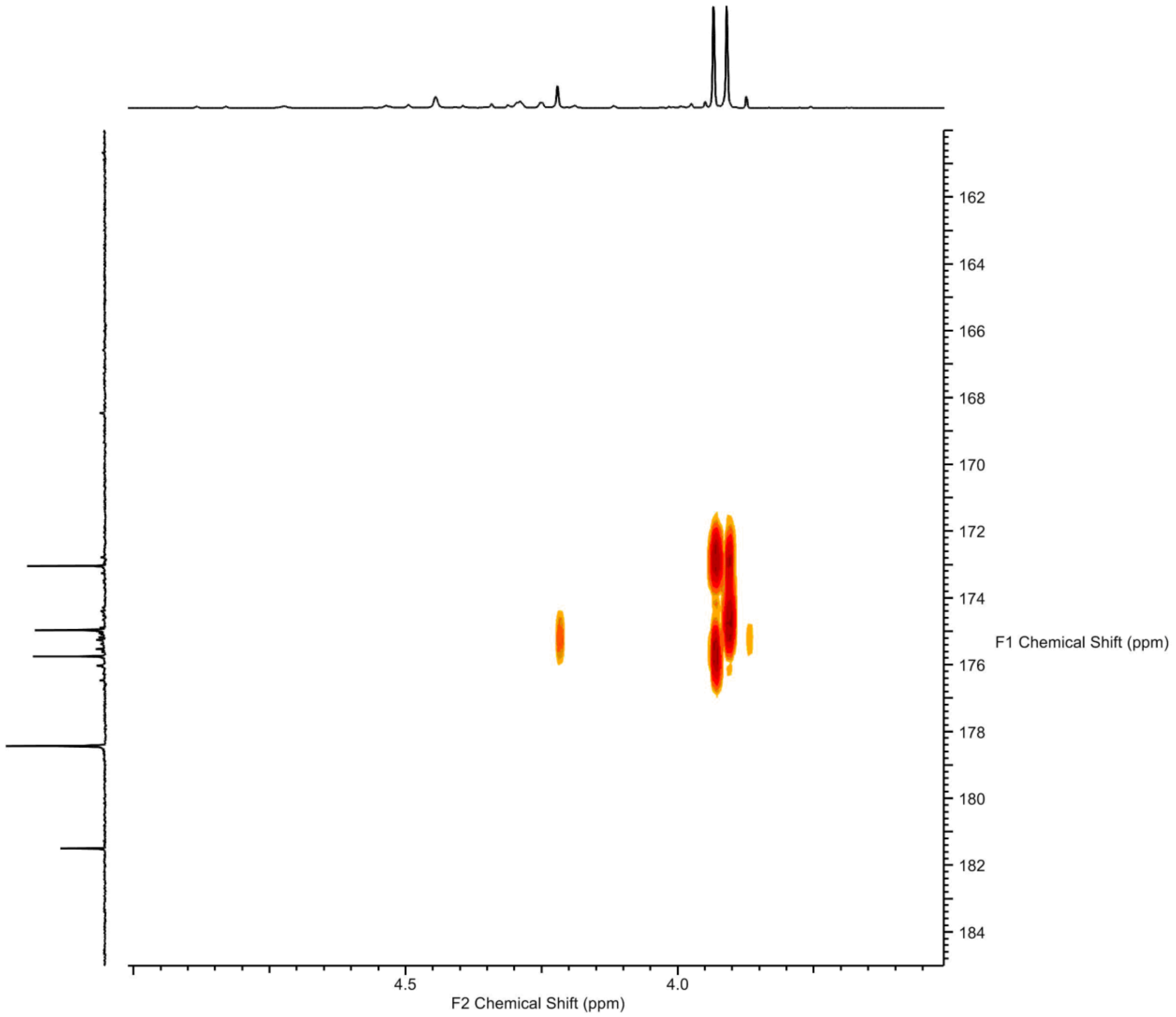

Figure S72: ^{1}H–^{13}C HMBC (^{1}H: 700 MHz [3.5–5.0 ppm], ^{13}C: 176 MHz [160-185 ppm], H_2O/D_2O 9:1) spectrum showing the diagnostic $^2J_{CH}$ and $^3J_{CH}$ coupling of **Gly**-αH-CONH$_2$ in **5$_G$’** at 3.94 ppm with two resonances at 175.0 and 173.0 ppm.

*Coupling of N-acetylglycine nitrile **1** with L-histidine amide **2**$_H$**'** at pH 8.5 and room temperature*

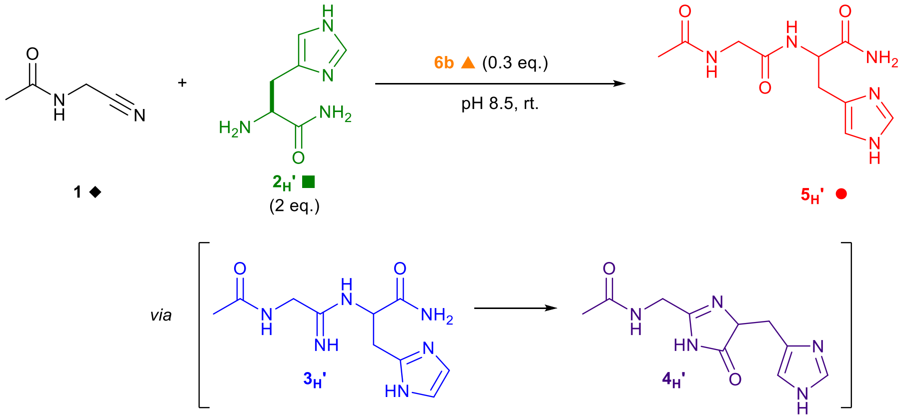

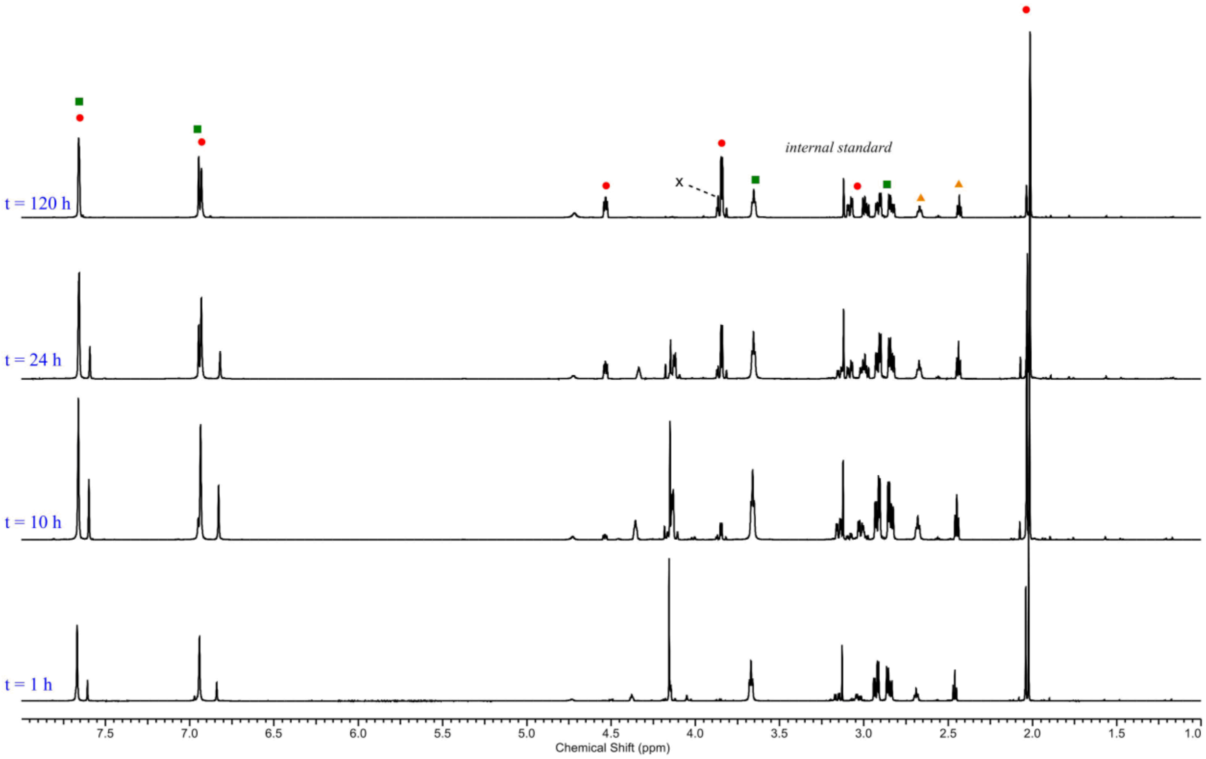

Figure S73: ^{1}H NMR (700 MHz, H_2O/D_2O 9:1, noesygppr1d, 1.0-5.0 ppm) spectrum to show the reaction of N-acetyl glycine nitrile (1, 200 mM) with L-histidine amide (**2$_H$'**, 400 mM) and 3-mercaptopropanoic acid (**6b**, 60 mM) with MSM (7 mM) as an internal standard at pH 8.5 and room temperature.

^{1}H NMR (700 MHz, H_2O/D_2O 9:1, noesygppr1d) **5$_H$'** (●): δ_H 7.69 (1H, s, Ar***H***), 6.98 (1H, s, Ar***H***), 4.57 (1H, dd, *J* = 8.3, 5.6 Hz, His-***αH***-COOH), 3.89 (1H, AB, *J* = 16.9 Hz, AcNHC***H***H), 3.86 (1H, AB, *J* = 16.9 Hz, AcNHCH***H***), 3.11 (1H, ABX, *J* = 15.0, 5.4 Hz, CH(CH***H***Ar)), 3.02 (1H, ABX, *J* = 15.0, 8.3 Hz, CH(C***H***HAr)), 2.05 (3H, s, *H*$_3$C(CO)); *L-histidine amide*, **2$_H$'** (■): δ_H 7.69 (1H, s, Ar*H*), 6.96 (1H, s, Ar*H*), 3.69 (1H, t, *J* = 6.3 Hz, α-***H***-COOH), 2.94 (1H, ABX, *J* = 14.7, 6.1 Hz, CH(CH***H***Ar), 2.87 (1H, ABX, *J* = 14.7, 7.0 Hz, CH(C***H***HAr)); *N-acetylglycinamide*, **7** (✖) (partial assignment): δ_H 3.90 (2H, s, C*H*$_2$).

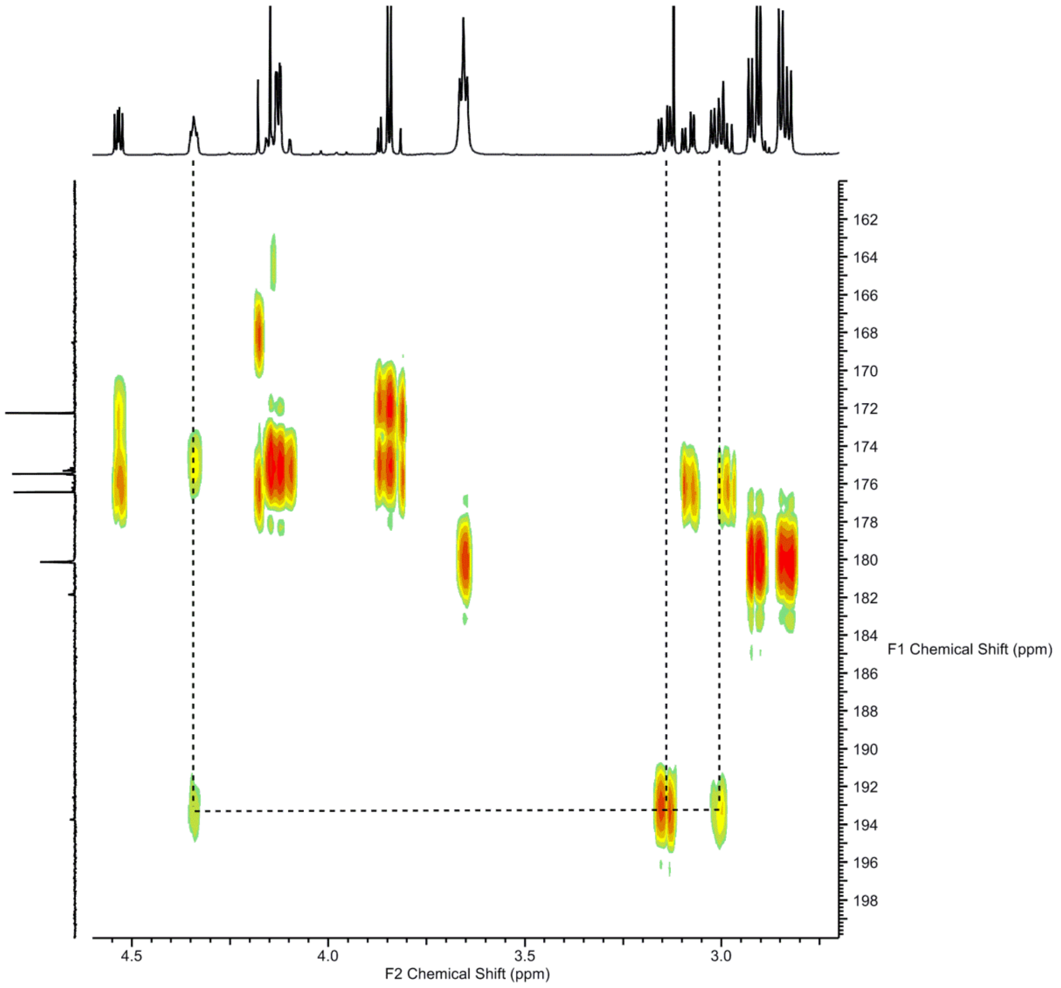

Figure S74: ^{1}H–^{13}C HMBC (^{1}H: 700 MHz [2.7-4.6 ppm], ^{13}C: 176 MHz [160-200 ppm], H_2O/D_2O 9:1) spectrum for the coupling of **1** and **2**$_{\mathbf{H}}$**'** catalyzed by 3-mercaptopropionic acid (**6b**) at pH 8.5 after 24 h, showing the diagnostic coupling of the imidazolone amide signal in **4**$_{\mathbf{H}}$ at 193.8 ppm with signals at 4.34 ppm (**His**-αH, $^2J_{CH}$), 3.15 and 3.00 ppm (**His**-βH, $^3J_{CH}$), which is characteristic of imidazolone formation.

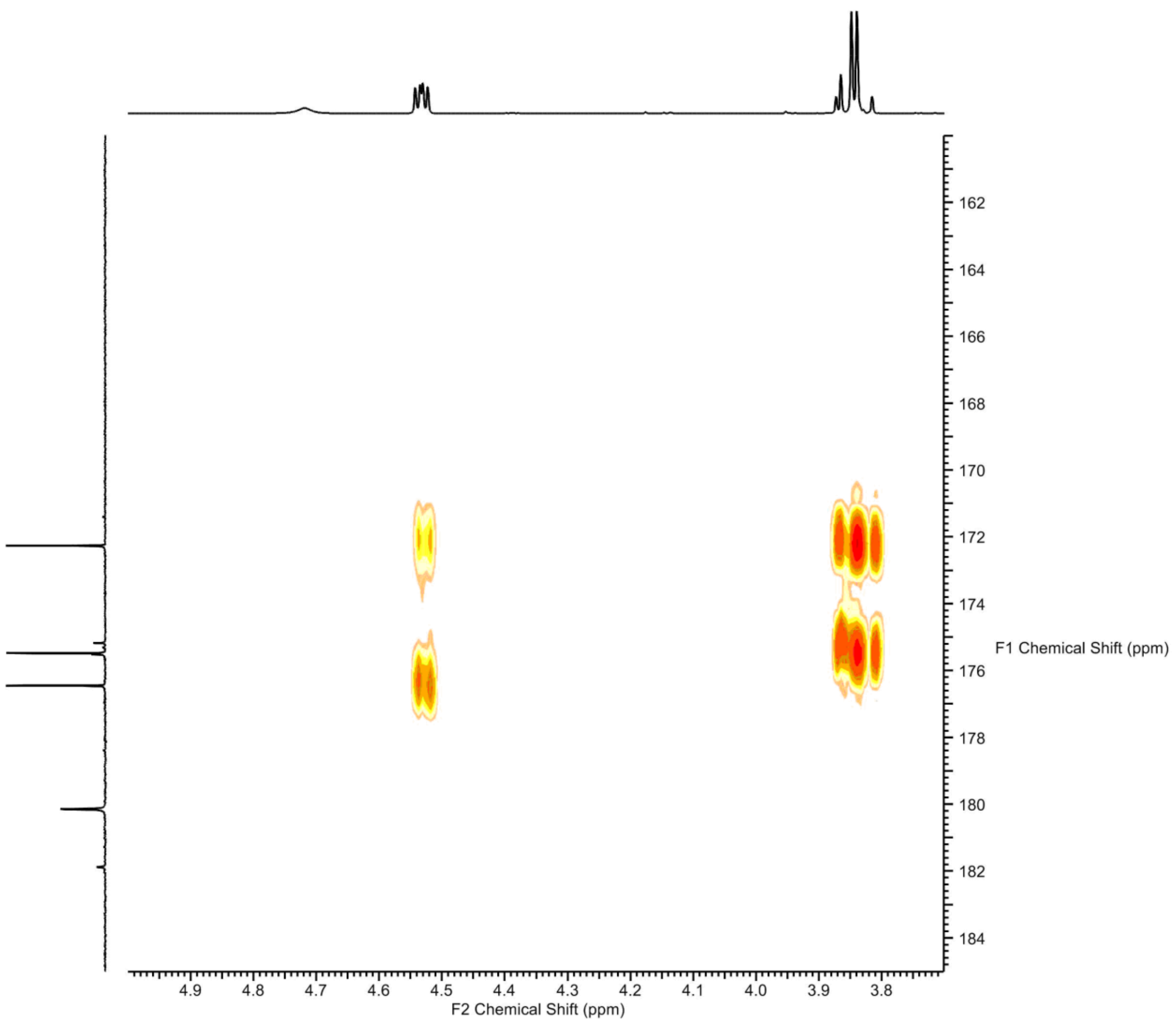

Figure S75: ^{1}H–^{13}C HMBC (^{1}H: 700 MHz [3.7–5.0 ppm], ^{13}C: 176 MHz [160–185 ppm], H_2O/D_2O 9:1) spectrum showing the diagnostic $^{2}J_{CH}$ and $^{3}J_{CH}$ coupling of the His-αH in 5_H' at 4.57 ppm with two resonances at 176.4 and 172.2 ppm.

*Coupling of N-acetylglycine nitrile **1** with L-isoleucinamide **2$_I$'** at pH 8.5 and room temperature*

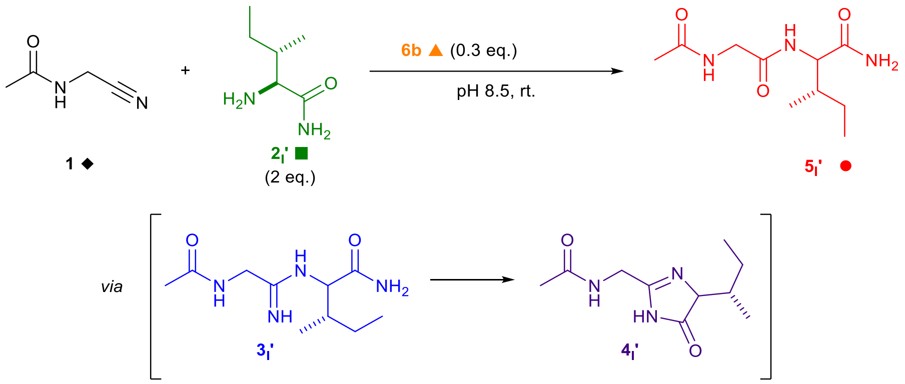

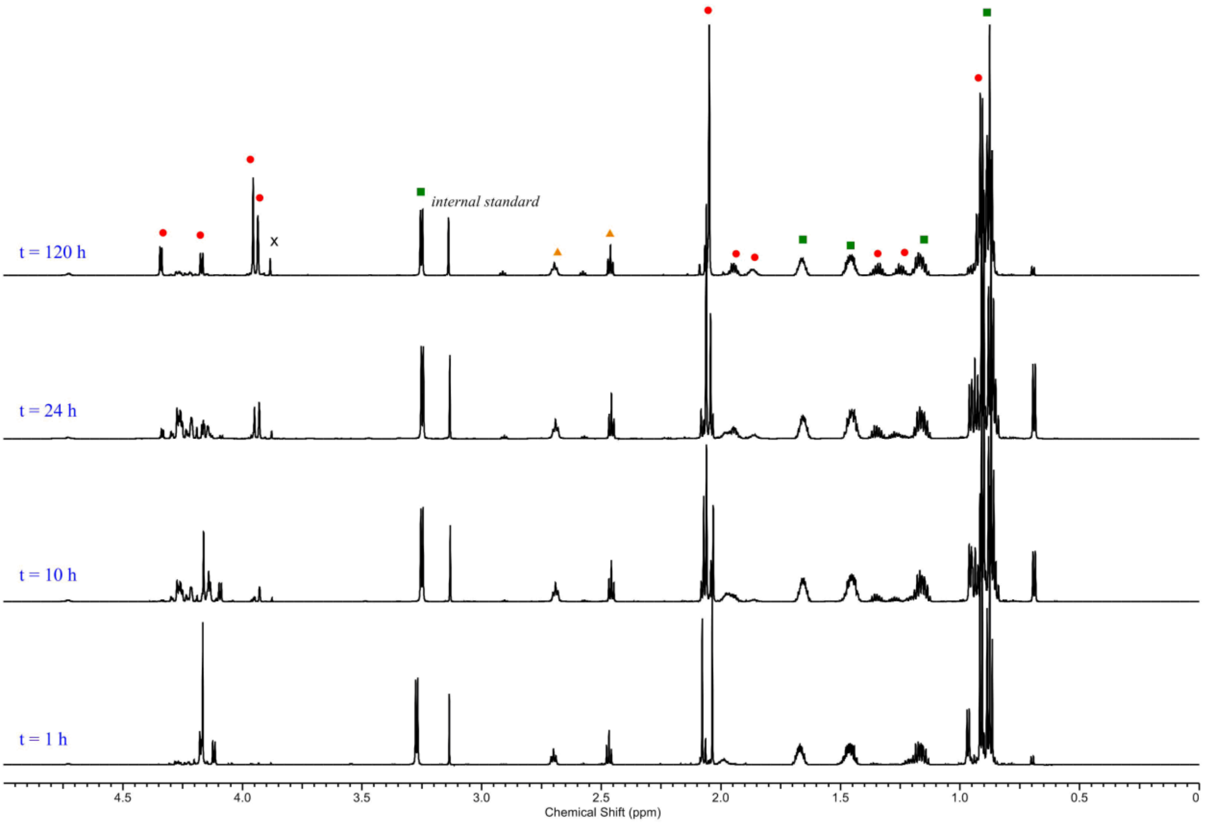

Figure S76: ^{1}H NMR (700 MHz, H_2O/D_2O 9:1, noesygppr1d, 0.0–5.0 ppm) spectrum to show the reaction of N-acetyl glycine nitrile (**1**, 200 mM) with L-isoleucinamide (**2$_I$'**, 400 mM) and 3-mercaptopropanoic acid (**6b**, 60 mM) with MSM (7 mM) as an internal standard at pH 8.5 and room temperature and ✖ = **7**.

^{1}H NMR (700 MHz, H_2O/D_2O 9:1, noesygppr1d) **5$_I$'** (●) (partial assignment, mixture of diastereomers A and B, 11:9): δ_H 4.34 (1H, d, *J* = 5.4 Hz, A Ile-***αH***-COOH), 4.18 (1H, d, *J* = 7.0 Hz, B Ile-***αH***-COOH), 3.96 (2H, s, A AcNHC***H$_2$***), 3.94 (2H, s, B AcNHC***H$_2$***), 2.05 (3H each for A and B, s, *H*$_3$C(CO)), 1.98-1.91 (m, 1H, A Ile-βC***H***(CH$_3$)CH$_2$CH$_3$), 1.38-1.31 (m, 1H, A Ile-CH(CH$_3$)C***H***HCH$_3$), 1.22-1.22 (m, 1H, A Ile-CH(CH$_3$)CH***H***CH$_3$)*; L-isoleucinamide*, **2$_I$'** (■): δ_H 3.25 (1H, d, *J* = 6.1 Hz, ***αH***-CONH$_2$) 1.69-1.63 (1H, m, Ile-βC***H***(CH$_3$)CH$_2$CH$_3$) 1.49-1.42 (1H, m, Ile-CH(CH$_3$)CH***H***CH$_3$), 1.20-1.12 (1H, m, Ile-CH(CH$_3$)CH***H***CH$_3$), 0.91 (3H, d, *J* = 7.0 Hz, Ile-CH(C***H$_3$***)CH$_2$CH$_3$), 0.88 (3H, t, *J* = 7.4 Hz, Ile-CH(CH$_3$)CH$_2$C***H$_3$***); *N-acetylglycinamide*, **7** (✖) (partial assignment): δ_H 3.90 (2H, s, C*H*$_2$).

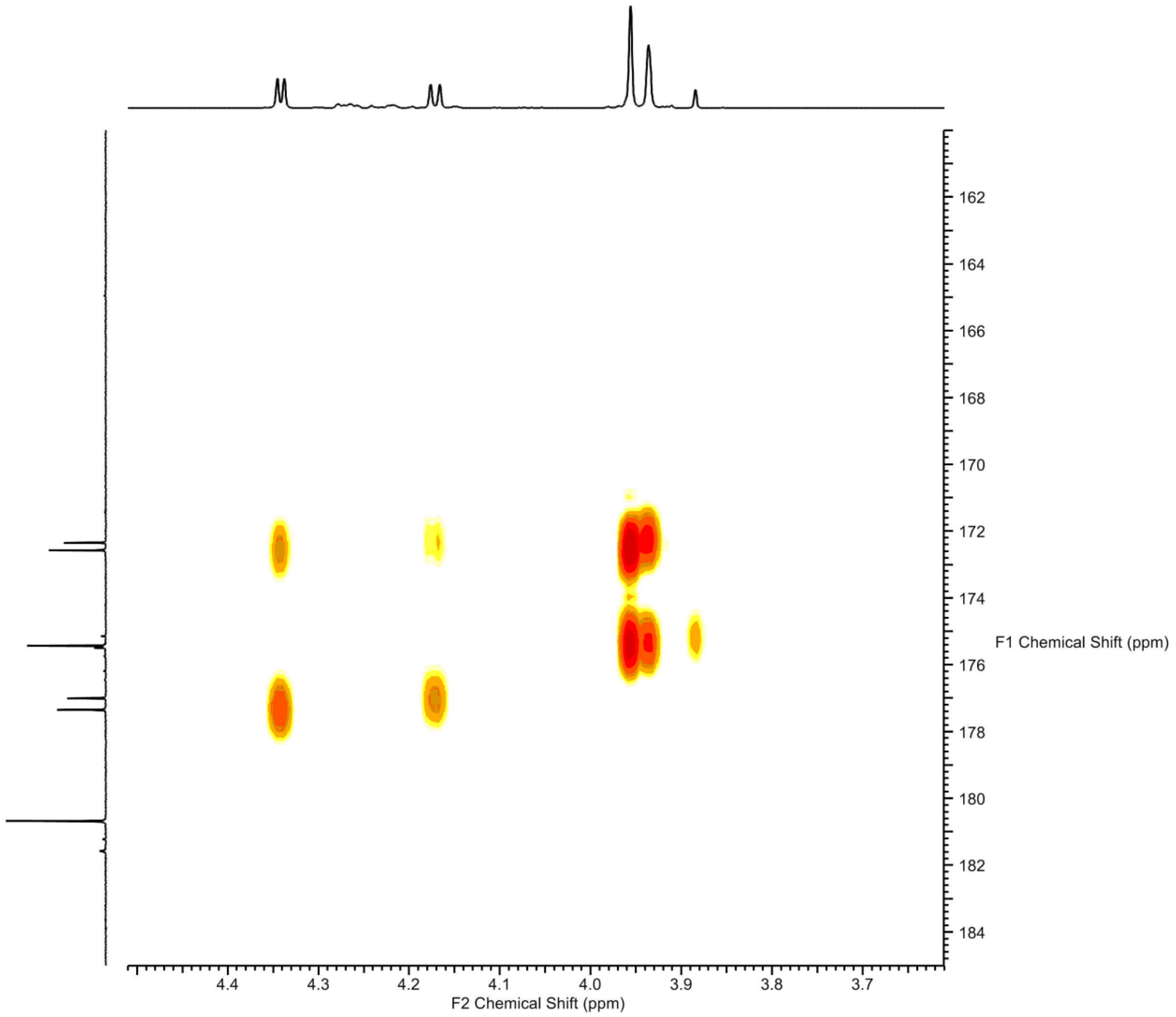

Figure S77: ^1H–^{13}C HMBC (1H: 700 MHz [3.4–4.5 ppm], 13C: 176 MHz [150–185 ppm], H2O/D2O 9:1) spectrum showing the diagnostic $^2J_{CH}$ and $^3J_{CH}$ coupling of the two diastereomers of **Ile**-αH in **5I'** at 4.34 ppm with two resonances at 177.3 and 172.6 ppm, and 4.18 ppm with two resonances at 177.0 and 172.3 ppm.

*Coupling of N-acetylglycine nitrile **1** with L-leucinamide **2L'** at pH 8.5 and room temperature*

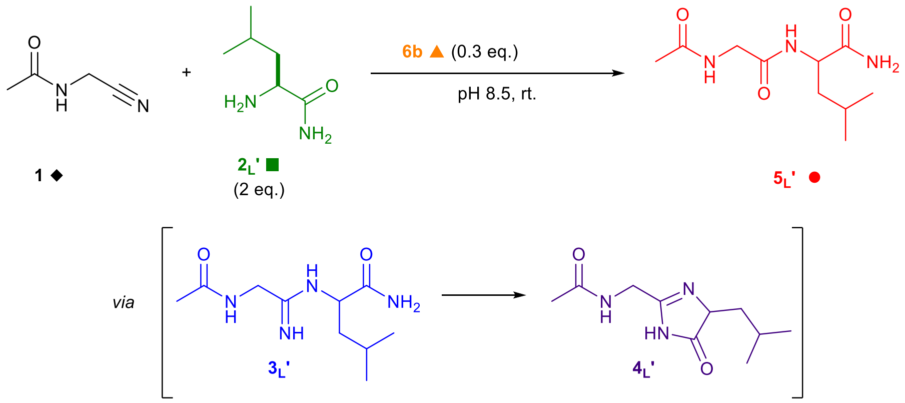

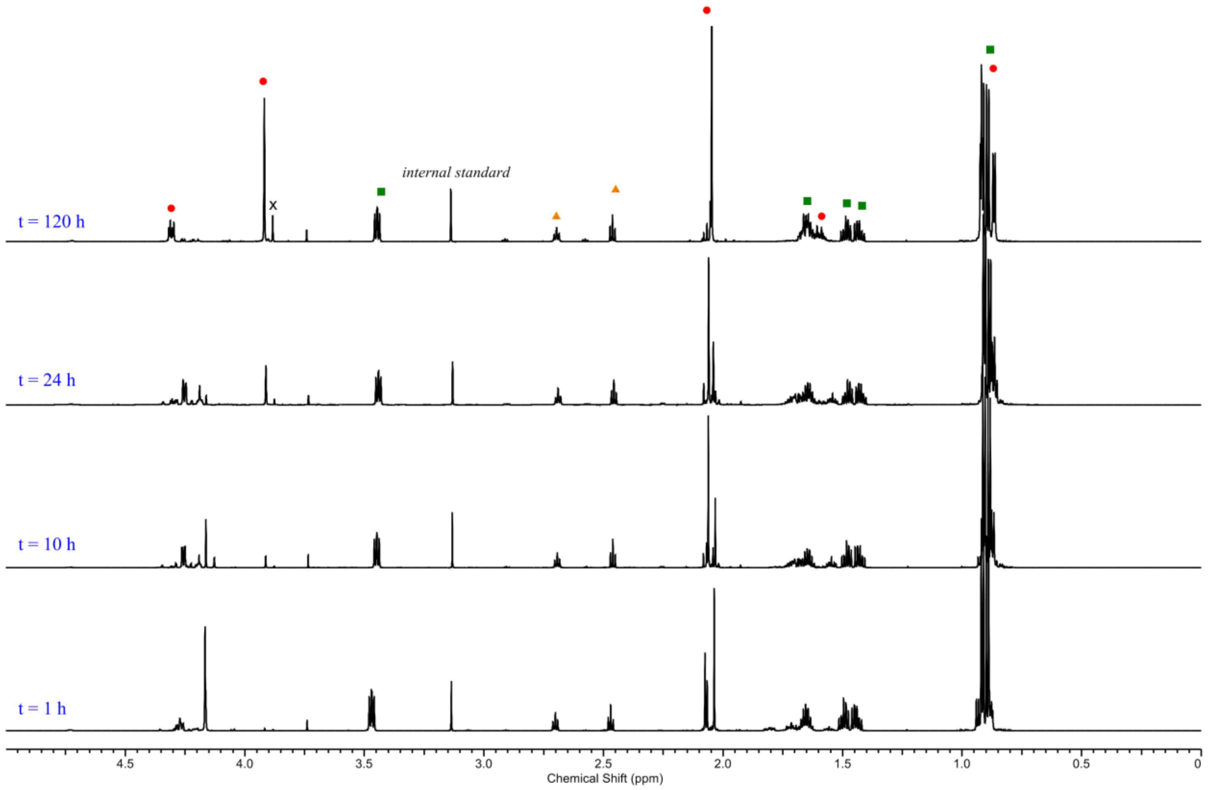

Figure S78: ^{1}H NMR (700 MHz, H_2O/D_2O 9:1, noesygppr1d, 0.0-5.0 ppm) spectrum to show the reaction of N-acetyl glycine nitrile (**1**, 200 mM) with L-leucinamide (**2L'**, 400 mM) and 3-mercaptopropanoic acid (**6b**, 60 mM) with MSM (7 mM) as an internal standard at pH 8.5 and room temperature. ✖ = **7**.

^{1}H NMR (700 MHz, H_2O/D_2O 9:1, noesygppr1d) **5L'** (●) (partial assignment): δ_H 4.34 (1H, dd, *J* = 10.4, 4.4 Hz, Leu-***αH***-COOH), 3.95 (1H, AB, *J* = 17.1 Hz, AcNHC***H₂***), 2.07 (3H, s, *H*₃C(CO)), 1.72-1.58 (3H, m; Leu-βC***H₂***-C***H***-(CH₃)₂), 0.95 (3H, d, *J* = 6.1 Hz, Leu-(C*H*₃)), 0.89 (3H, d, *J* = 6.3 Hz, Leu-(C*H*₃)); *L-leucinamide*, **2L'** (■) (partial assignment): δ_H 3.47 (1H, dd, *J* = 8.0, 6.4 Hz, ***αH***-CONH₂),1.72-1.64 (1H, m; Leu-C***H***-(CH₃)₂), 1.54-1.43 (2H, m; Leu-βC***H₂***), 0.94 (3H, d, *J* = 6.7 Hz, Leu-(C*H*₃)), 0.92 (3H, d, *J* = 6.3 Hz, Leu-(C*H*₃)); *N-acetylglycinamide*, **7** (✖) (partial assignment): δ_H 3.91 (2H, s, C*H*₂).

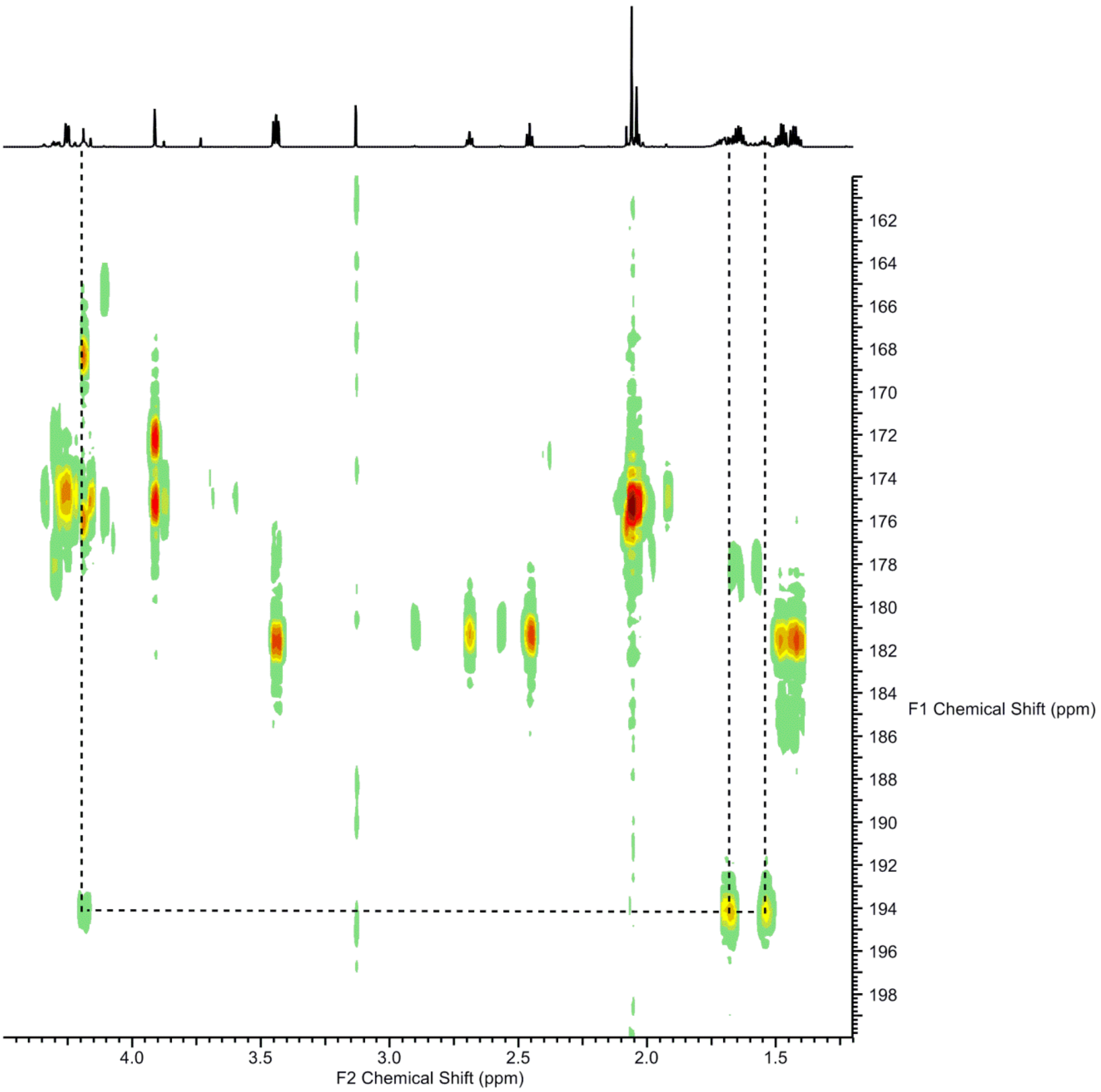

Figure S79: ^1H–^{13}C HMBC (^1H: 700 MHz [1.7-4.5 ppm], ^{13}C: 176 MHz [160-200 ppm], H_2O/D_2O 9:1) spectrum for the coupling of **1** and **2_L'** catalyzed by 3-mercaptopropionic acid (**6b**) at pH 8.5 after 24 h, showing the diagnostic coupling of the imidazolone amide signal in **4_L** at 194.2 ppm with signals at 4.19 ppm (**Leu**-αH, $^2J_{CH}$), 1.70 and 1.54 ppm (**Leu**-βH, $^3J_{CH}$), which is characteristic of imidazolone formation.

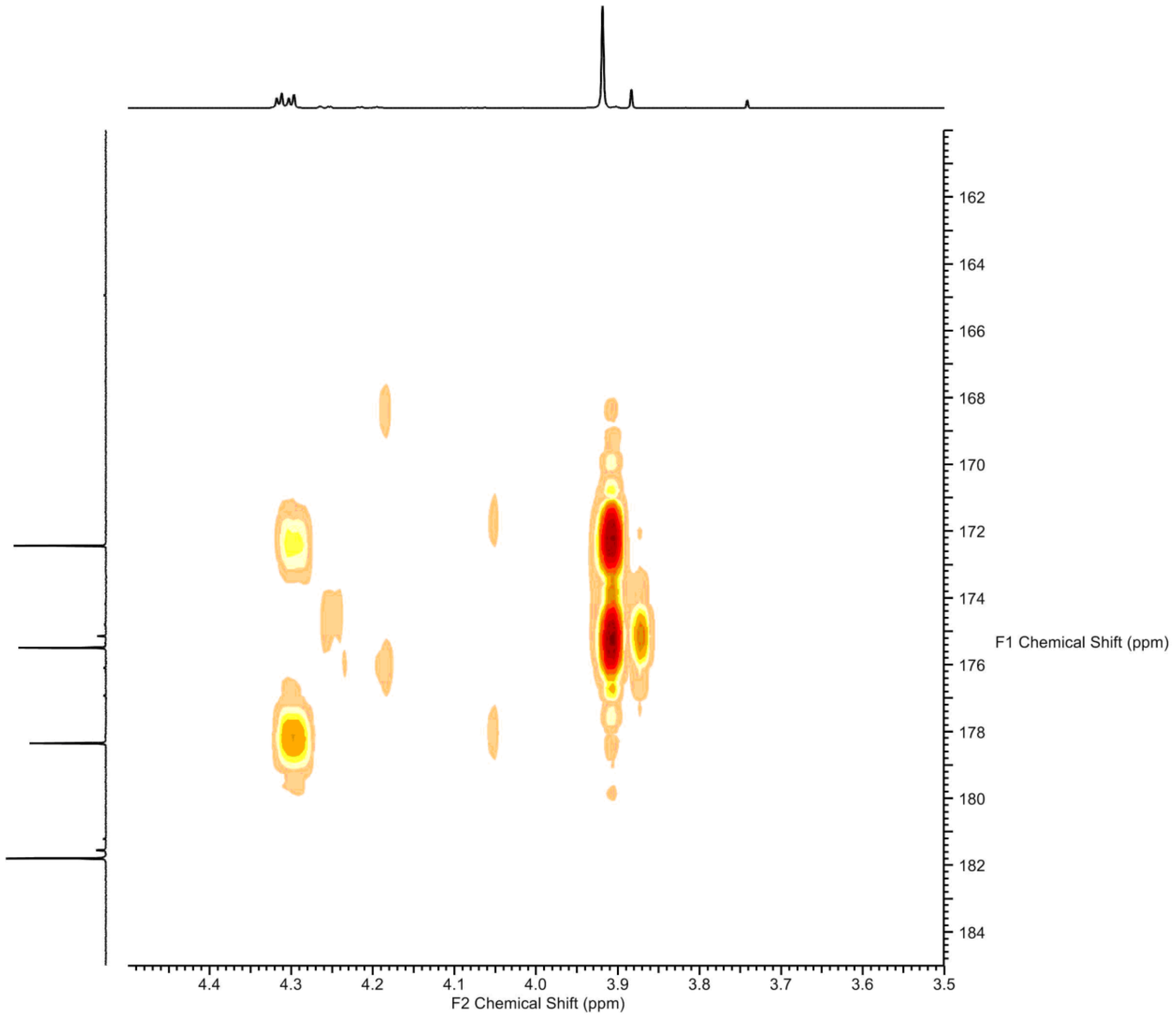

Figure S80: ^{1}H–^{13}C HMBC (^{1}H: 700 MHz [3.5-4.5 ppm], ^{13}C: 176 MHz [150-185 ppm], H_2O/D_2O 9:1) spectrum showing the diagnostic $^{2}J_{CH}$ and $^{3}J_{CH}$ coupling of the **Leu**-αH in **5L'** at 4.08 ppm with two resonances at 178.2 and 172.2 ppm, which is characteristic of amide bond formation of **2L'**.

*Coupling of N-acetylglycine nitrile **1** with DL-methionine amide **2**$_{M}$**'** at pH 8.5 and room temperature*

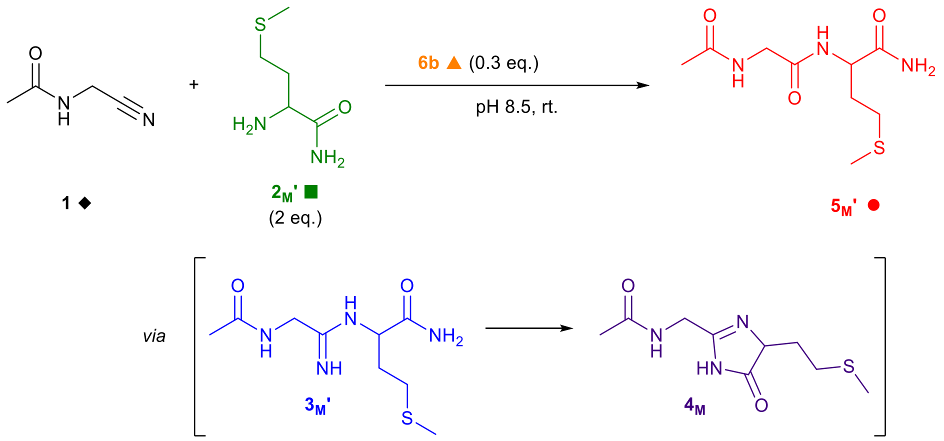

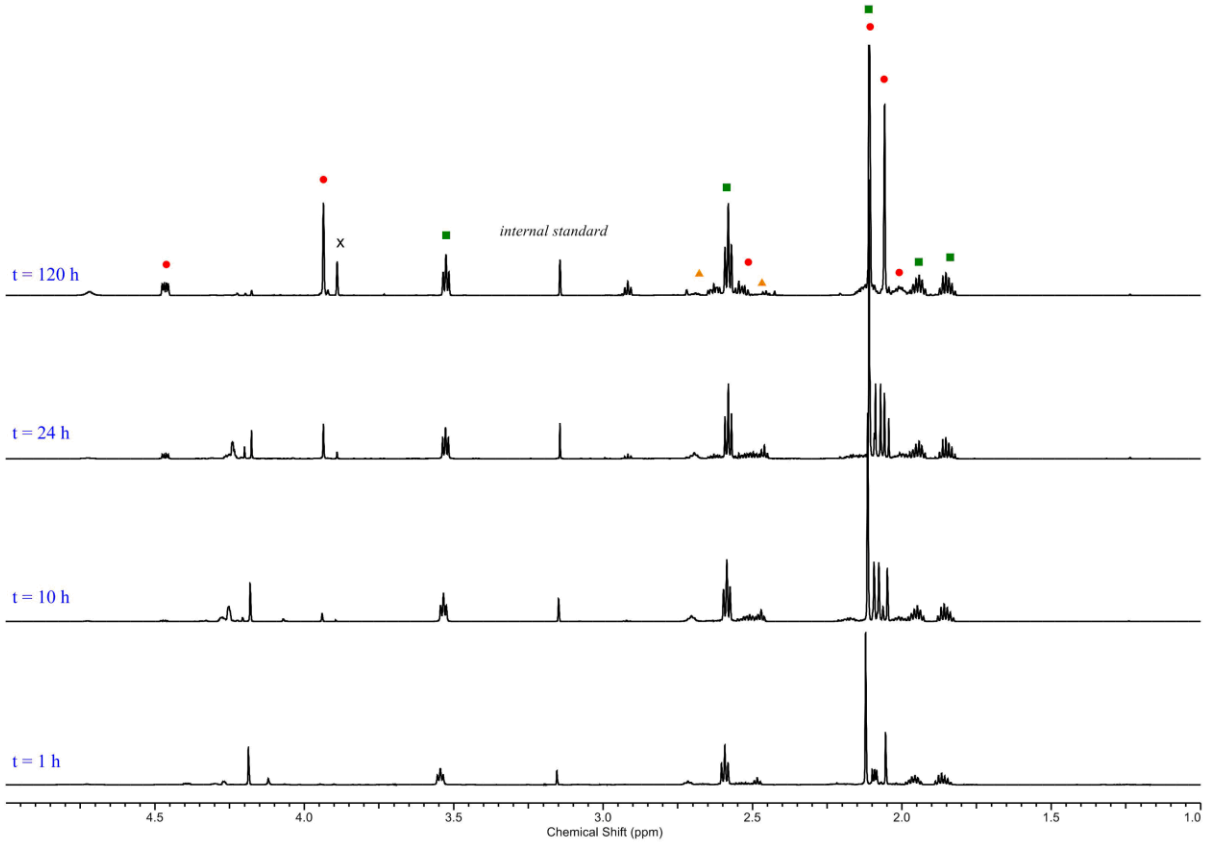

Figure S81: ^{1}H NMR (700 MHz, H_2O/D_2O 9:1, noesygppr1d, 1.0-5.0 ppm) spectrum to show the reaction of N-acetyl glycine nitrile (**1**, 200 mM) with DL-methioninamide (**2**$_{M}$**'**, 400 mM) and 3-mercaptopropanoic acid (**6b**, 60 mM) with MSM (7 mM) as an internal standard pH 8.5 and room temperature. ✖ = 7.

^{1}H NMR (700 MHz, H_2O/D_2O 9:1, noesygppr1d) **5**$_{M}$**'** (●) (partial assignment): δ_H 4.50 (1H, dd, J = 9.7, 4.5 Hz, Met-***αH***-COOH), 3.97 (2H, s, AcNHC***H***$_2$), 2.68-2.54 (2H, m, C***H***$_2$SCH$_3$), 2.14 (3H, s, CH$_2$SC*H*$_3$), 2.09 (3H, s, *H*$_3$C(CO)), 2.19-2.04 (m, C***H***$_2$CH$_2$SCH$_3$); *DL-methioninamide*, **2**$_{M}$**'** (■) (partial assignment): δ_H 3.56 (1H, br. t., J = 6.6 Hz, αH-COOH), 2.61 (2H, t, J = 7.5 Hz, C*H*$_2$SCH$_3$), 2.14 (3H, s, SC*H*$_3$); *N-acetylglycinamide*, **7** (✖) (partial assignment): δ_H 3.92 (2H, s, C*H*$_2$).

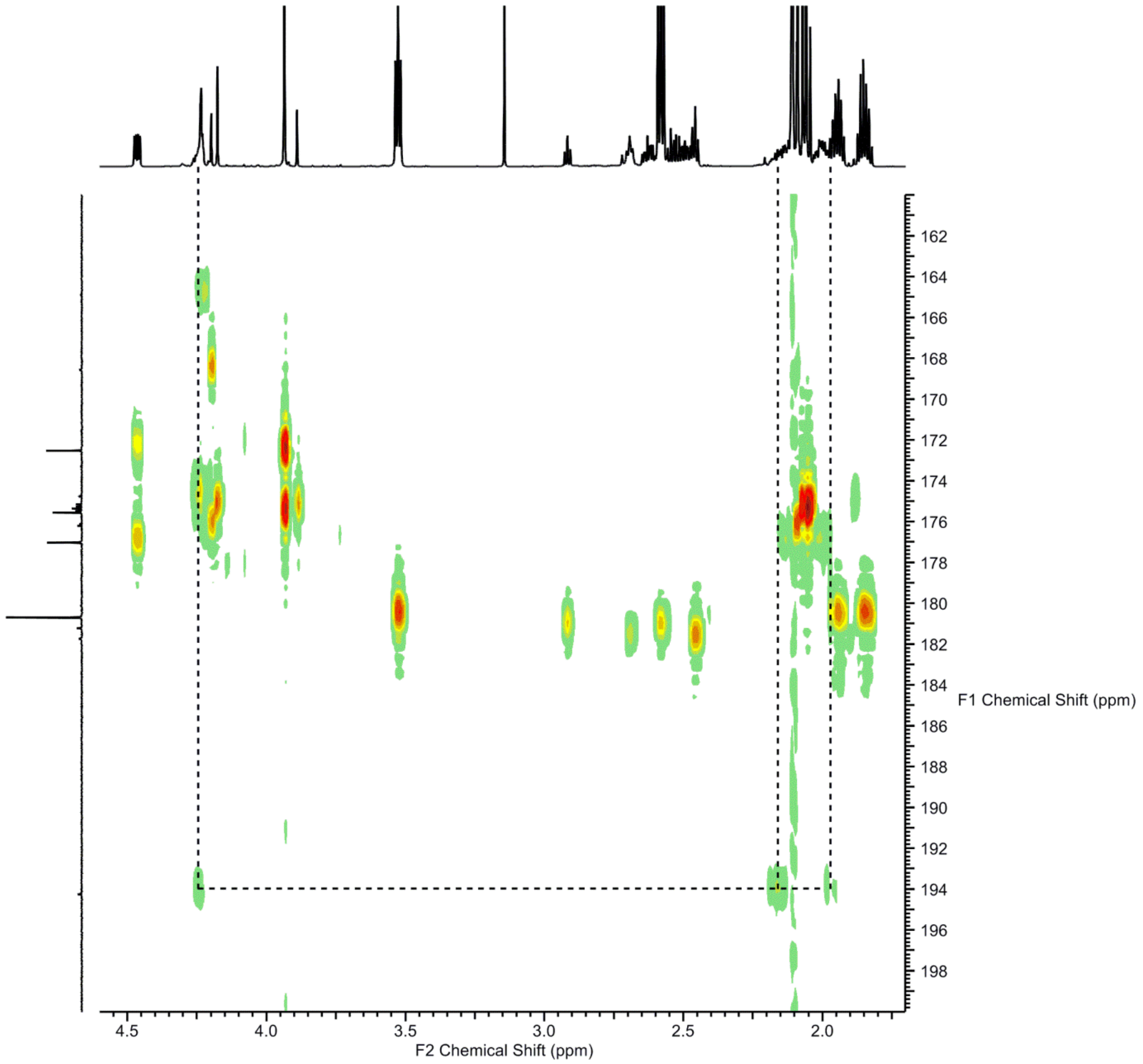

Figure S82: ^{1}H–^{13}C HMBC (^{1}H: 700 MHz [1.7-4.6 ppm], ^{13}C: 176 MHz [160-200 ppm], H_2O/D_2O 9:1) spectrum for the coupling of **1** and **2_M'** catalyzed by 3-mercaptopropionic acid (**6b**) at pH 8.5 after 24 h, showing the diagnostic coupling of the imidazolone amide signal in **4_M** at 194.1 ppm with signals at 4.24 ppm (**Met**-αH, $^2J_{CH}$), 2.16 and 1.97 ppm (**Met**-βH, $^3J_{CH}$), which is characteristic of imidazolone formation.

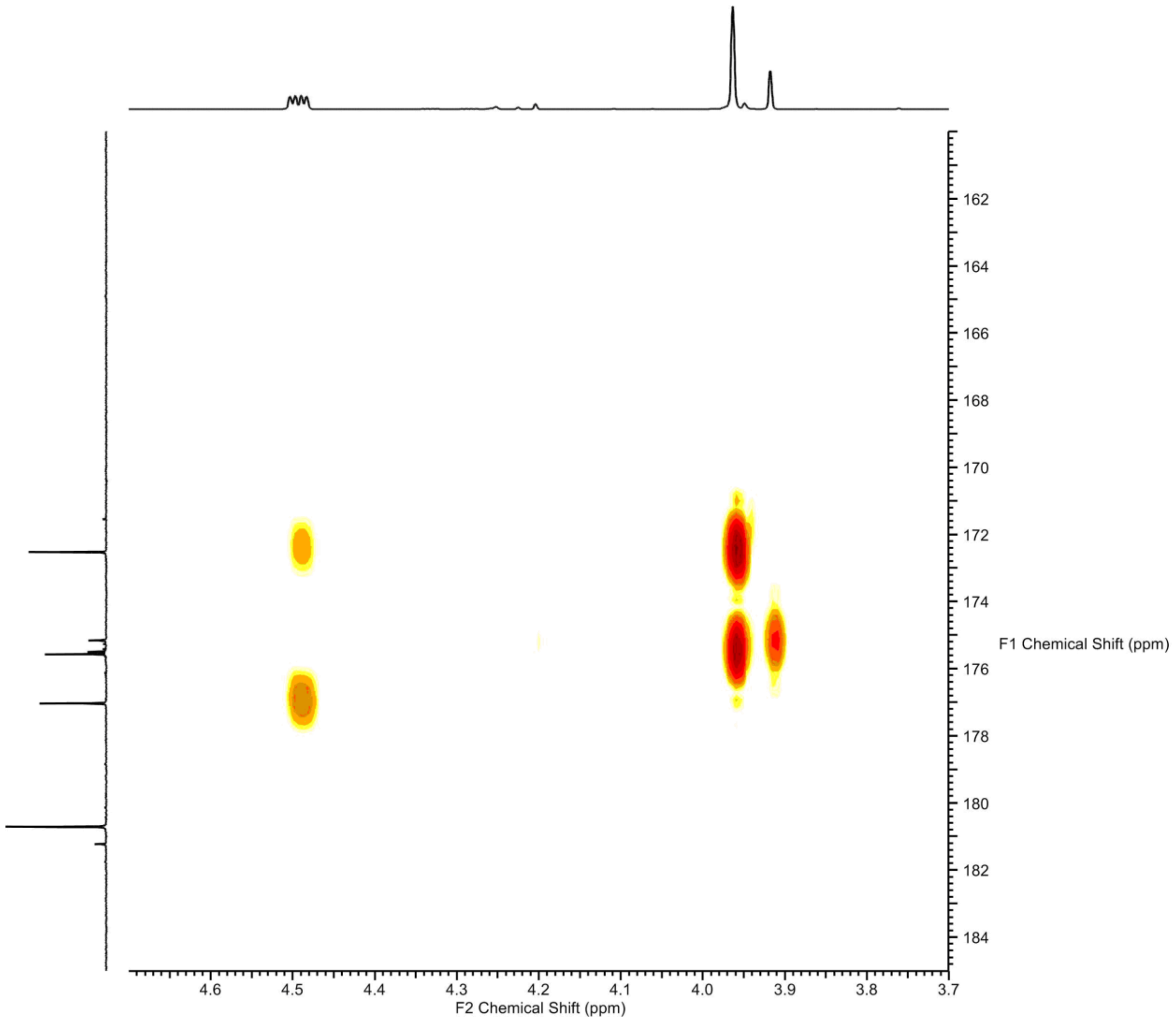

Figure S83: ^1H–^{13}C HMBC (^1H: 700 MHz [3.7-4.7 ppm], ^{13}C: 176 MHz [160-185 ppm], H_2O/D_2O 9:1) spectrum showing the diagnostic $^2J_{CH}$ and $^3J_{CH}$ coupling of the **Met**-αH in **5$_M$'** at 4.50 ppm with two resonances at 176.4 and 172.2 ppm, which is characteristic of amide bond formation.

*Coupling of N-acetylglycine nitrile **1** with L-phenylalaninamide **2$_F$'** at pH 8.5 and room temperature*

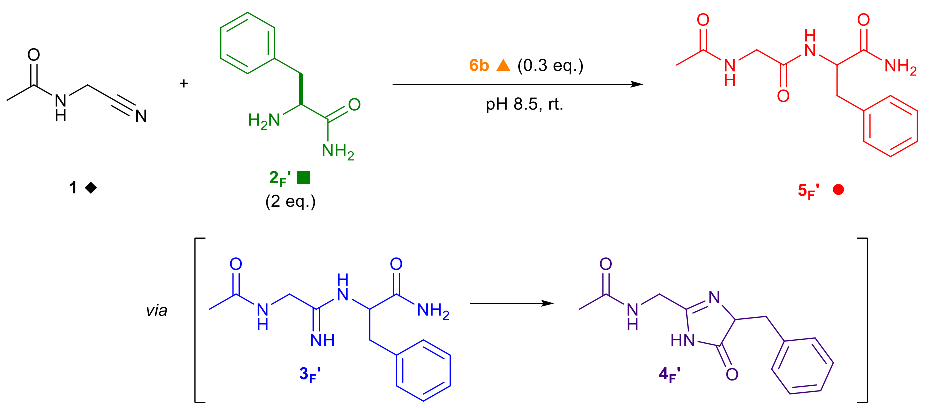

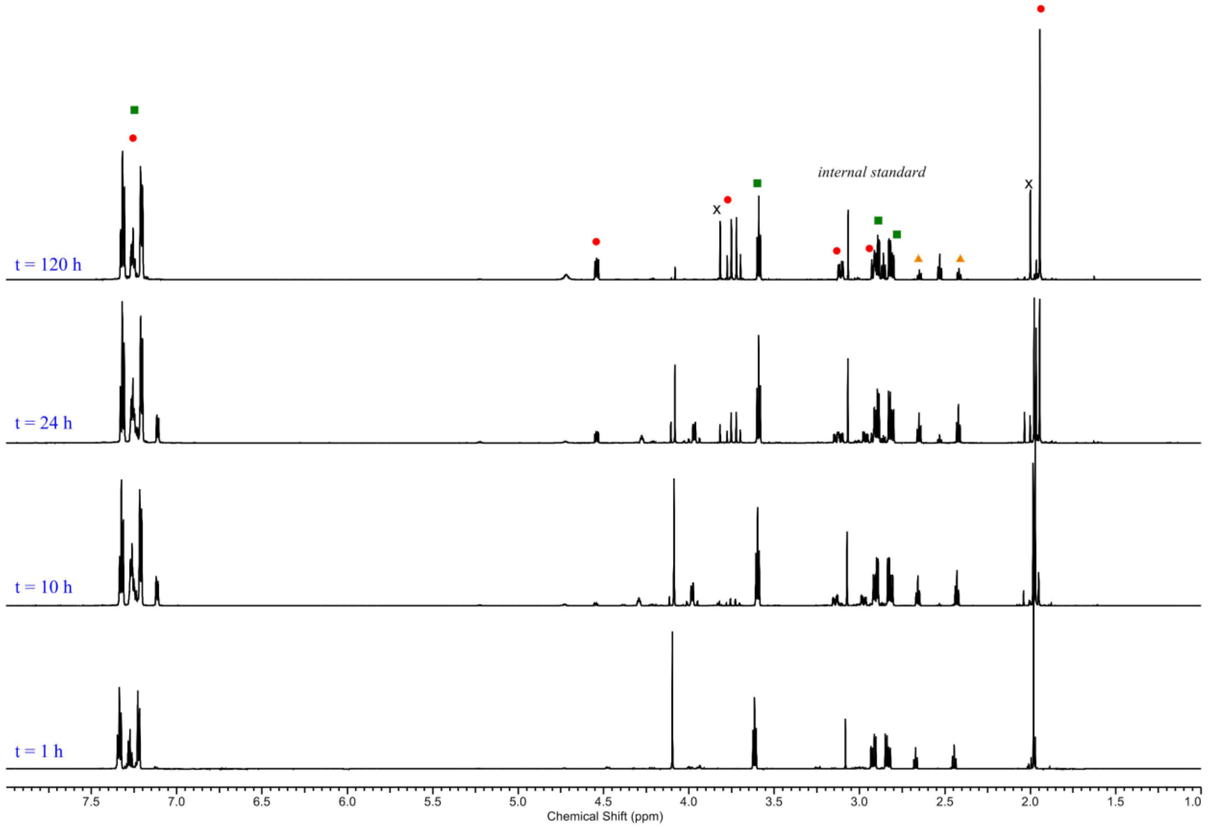

Figure S84: ^{1}H NMR (700 MHz, H_2O/D_2O 9:1, noesygppr1d, 1.0-8.0 ppm) spectrum to show the reaction of N-acetyl glycine nitrile (**1**, 200 mM) with L-phenylalaninamide (**2$_M$'**, 400 mM) and 3-mercaptopropanoic acid (**6b**, 60 mM) with MSM (7 mM) as internal standard at pH 8.5 and room temperature.

^{1}H NMR (700 MHz, H_2O/D_2O 9:1, noesygppr1d) **5$_F$'** (partial assignment) (●): δ_H 4.57 (1H, dd, *J* = 9.0, 5.8 Hz, Phe-***αH***-COOH), 3.79 (1H, AB, *J* = 17.0 Hz, AcNHC***H***H), 3.74 (1H, AB, *J* = 17.0 Hz, AcNHCH**H**), 3.14 (1H, dd, *J* = 14.0, 5.5 Hz, CHCH***H***Ph), 1.98 (3H, s, *H*$_3$C(CO)); *L-phenylalaninamide* **2$_F$'** (■) (partial assignment): δ_H 3.62 (1H, t, *J* = 6.8 Hz, ***αH***-COOH), 2.84 (1H, ABX, *J* = 13.5, 7.1 Hz, CHCH***H***Ph); *N-acetylglycinamide*, **7**(✖) (partial assignment): δ_H 3.85 (2H, s, C*H*$_2$).

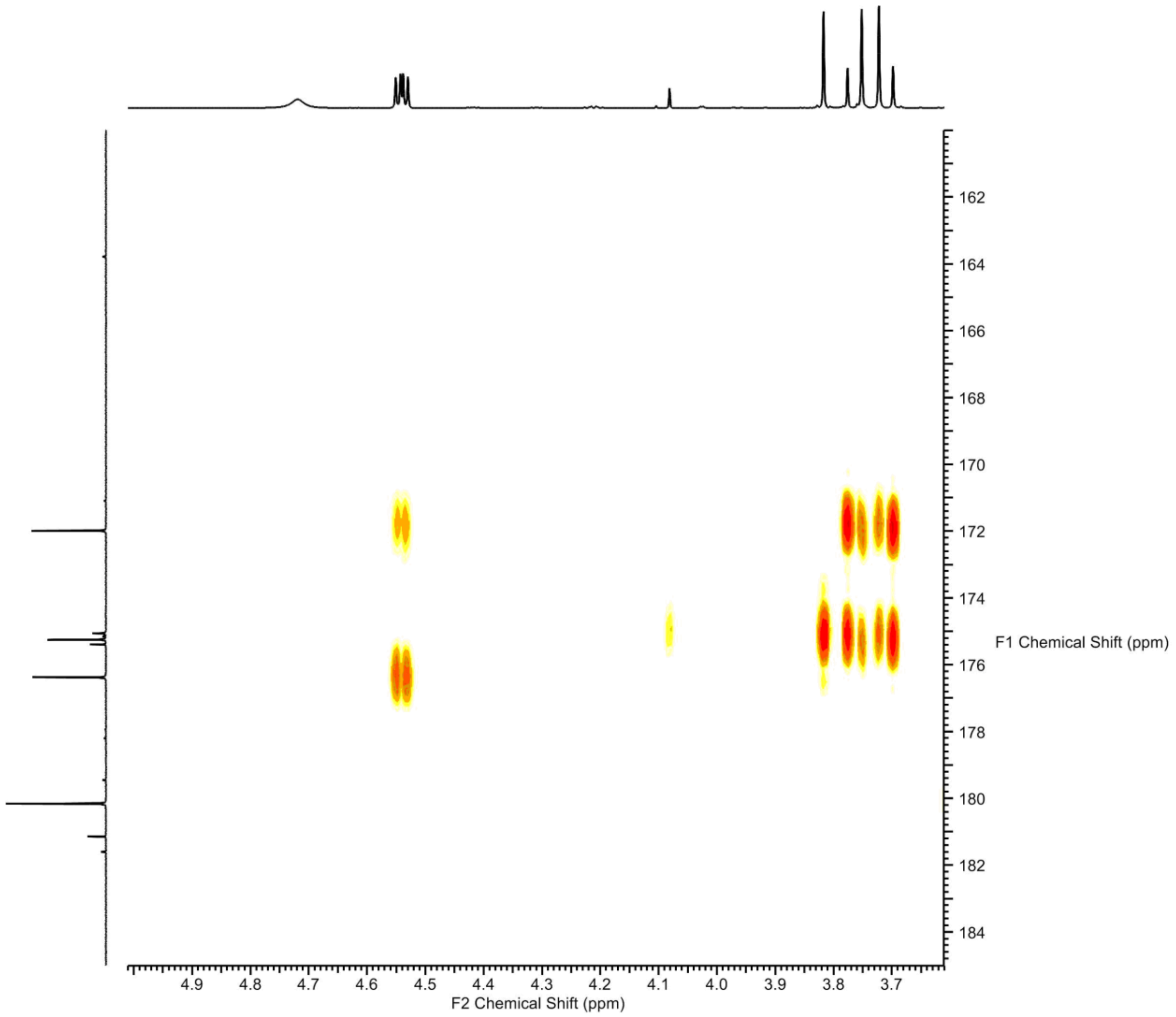

Figure S85: ^1H–^{13}C HMBC (^1H: 700 MHz [3.6-5.0 ppm], ^{13}C: 176 MHz [160-185 ppm], H_2O/D_2O 9:1) spectrum showing the diagnostic $^2J_{CH}$ and $^3J_{CH}$ coupling of the **Phe**-αH in **5$_F$'** at 4.57 ppm with two resonances at 176.2 and 172.0 ppm, which is characteristic of amide bond formation.

*Coupling of N-acetylglycine nitrile **1** with L-prolinamide **2P'** at pH 8.5 and room temperature*

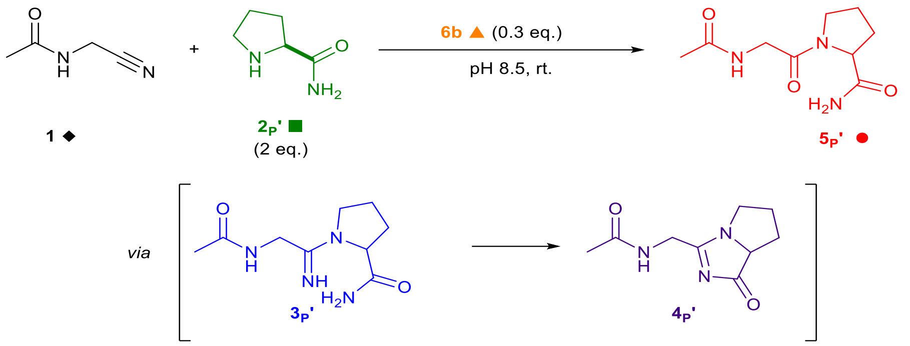

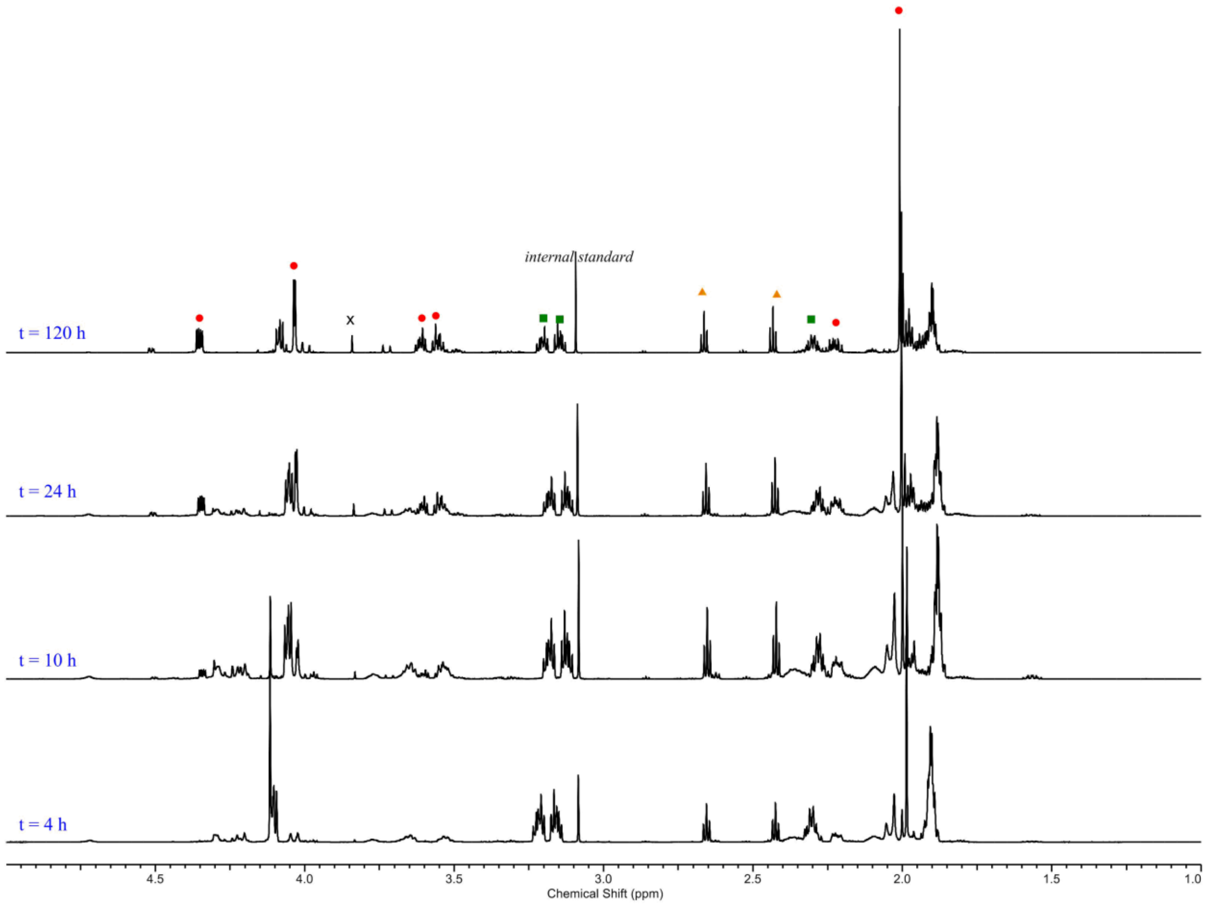

Figure S86: ^{1}H NMR (700 MHz, H_2O/D_2O 9:1, noesygppr1d, 1.0-5.0 ppm) spectrum to show the reaction of N-acetyl glycine nitrile (**1**, 200 mM) with L-prolinamide (**2P'**, 400 mM) and 3-mercaptopropanoic acid (**6b**, 60 mM) with MSM (7 mM) as an internal standard at pH 8.5 and room temperature.

^{1}H NMR (700 MHz, H_2O/D_2O 9:1, noesygppr1d) **5P'** (●) (partial assignment): δ_H 4.38 (1H, dd, *J* = 8.9, 4.4 Hz, Pro-***αH***-COOH), 4.07 (1H, AB, *J* = 17.4 Hz, AcNHCH***H***), 4.05 (1H, AB, *J* = 17.4 Hz, AcNHC***H***H), 3.66-3.62 (1H, m, NCH***H***CH_2CH_2), 3.60-3.56 (1H, m, NC***H***HCH_2CH_2), 2.28-2.23 (1H, m, NC($CONH_2$)HC***H***HCH_2), 2.03 (3H, s, *H_3*C(CO)); *L-prolinamide*, **2P'** (■) (partial assignment): δ_H 4.11 (1H, dd, *J* = 8.6, 6.4 Hz, αH-COOH), 3.25-3.21 (1H, m, HNCH***H***CH_2CH_2), 3.19-3.15 (1H, m, HNCH***H***CH_2CH_2) 2.35-2.30 (1H, m, NC($CONH_2$)HC***H***HCH_2); *N-acetylglycinamide*, **7** (✖) (partial assignment): δ_H 3.87 (2H, s, C*H_2*).

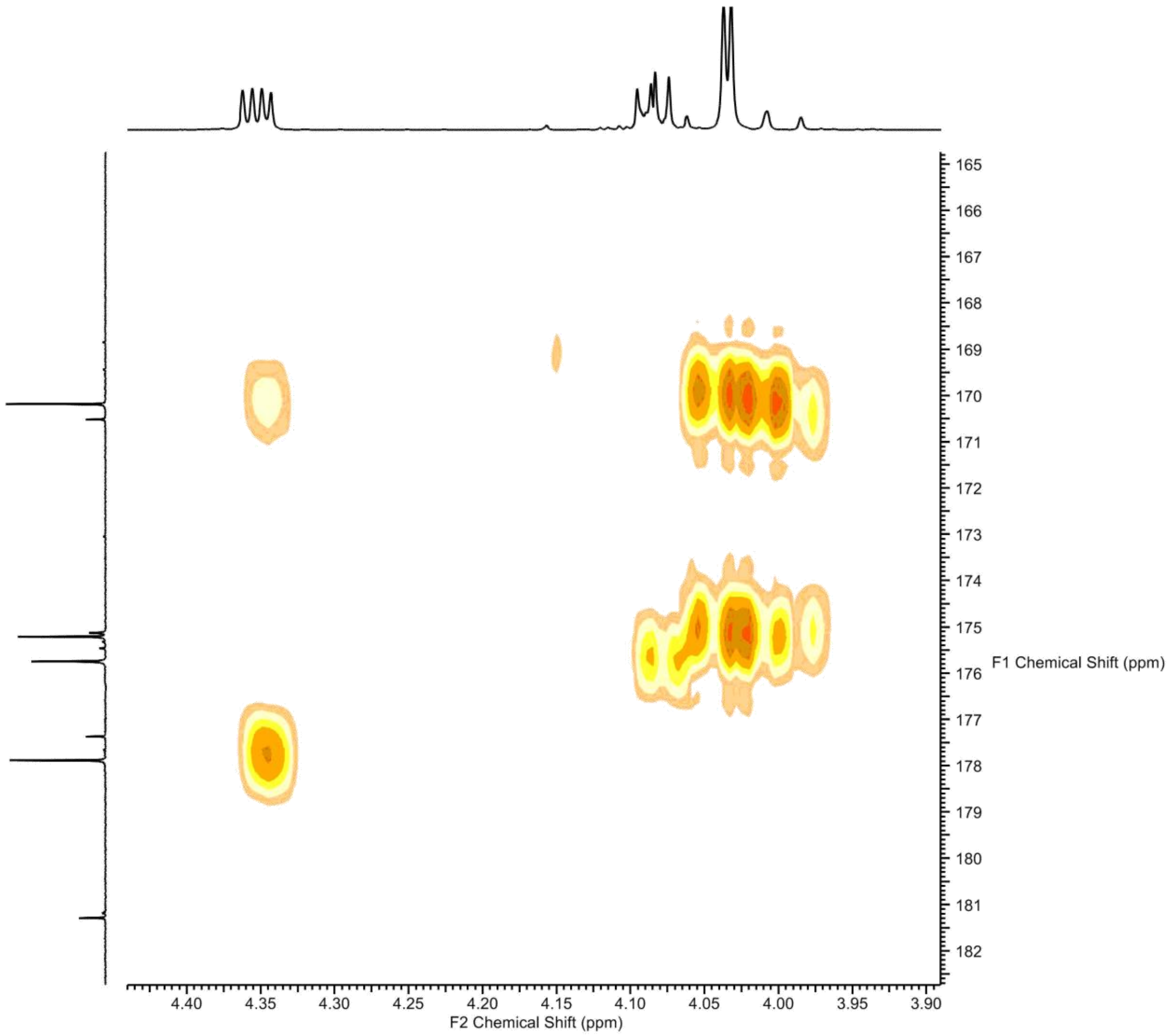

Figure S87: ^1H–^{13}C HMBC (^1H: 700 MHz [3.88-4.50 ppm], ^{13}C: 176 MHz [165-183 ppm], H_2O/D_2O 9:1) spectrum showing the diagnostic $^2J_{CH}$ coupling of the Gly-CH_2 AB in 5_P' at 4.07 and 4.05 ppm with two resonances at 175.0 and 170.0 ppm, which is characteristic of amide bond formation of Pro.

*Coupling of N-acetylglycine nitrile **1** with L-serinamide **2s'** at pH 8.5 and room temperature*

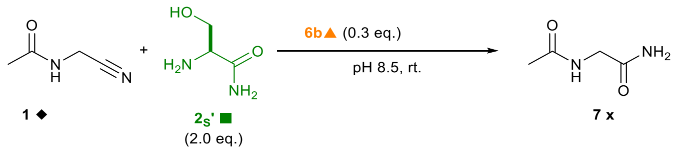

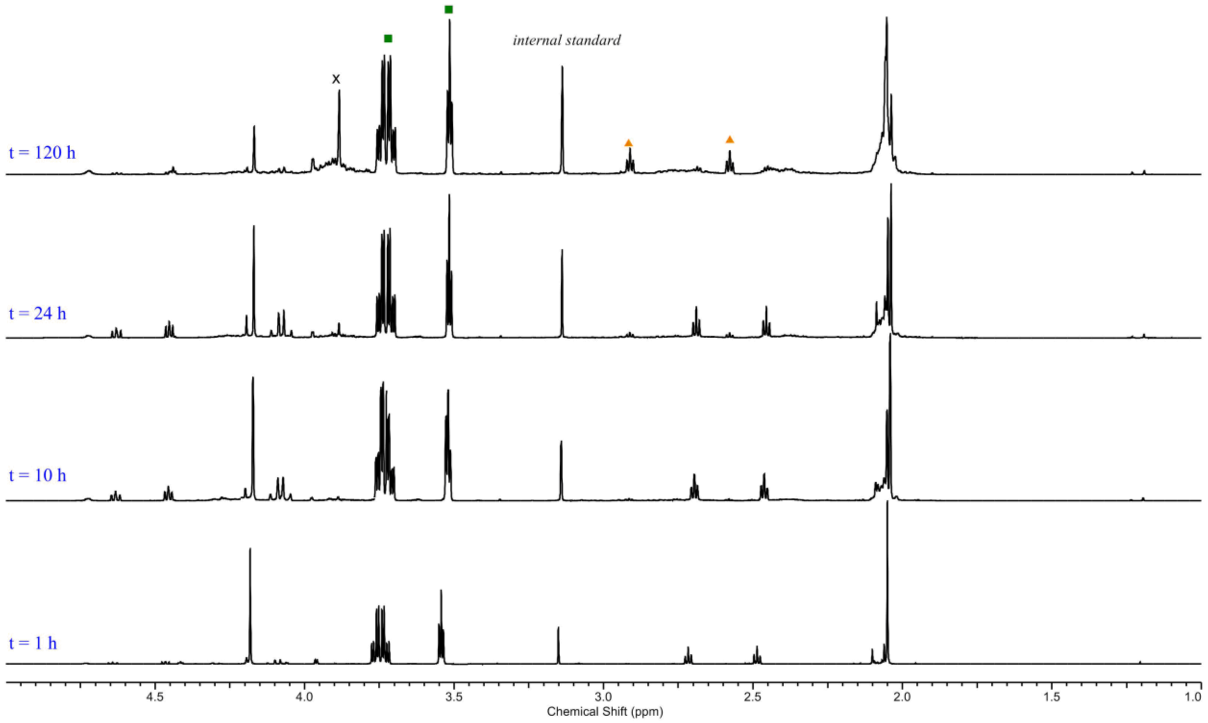

Figure S88: ^{1}H NMR (700 MHz, H_2O/D_2O 9:1, noesygppr1d, 1.0-5.0 ppm) spectrum to show the reaction of N-acetyl glycine nitrile (**1**, 200 mM) with L-serinamide (**2s'**, 400 mM) and 3-mercaptopropanoic acid (**6b**, 60 mM) with MSM (7 mM) as an internal standard at pH 8.5 and room temperature.

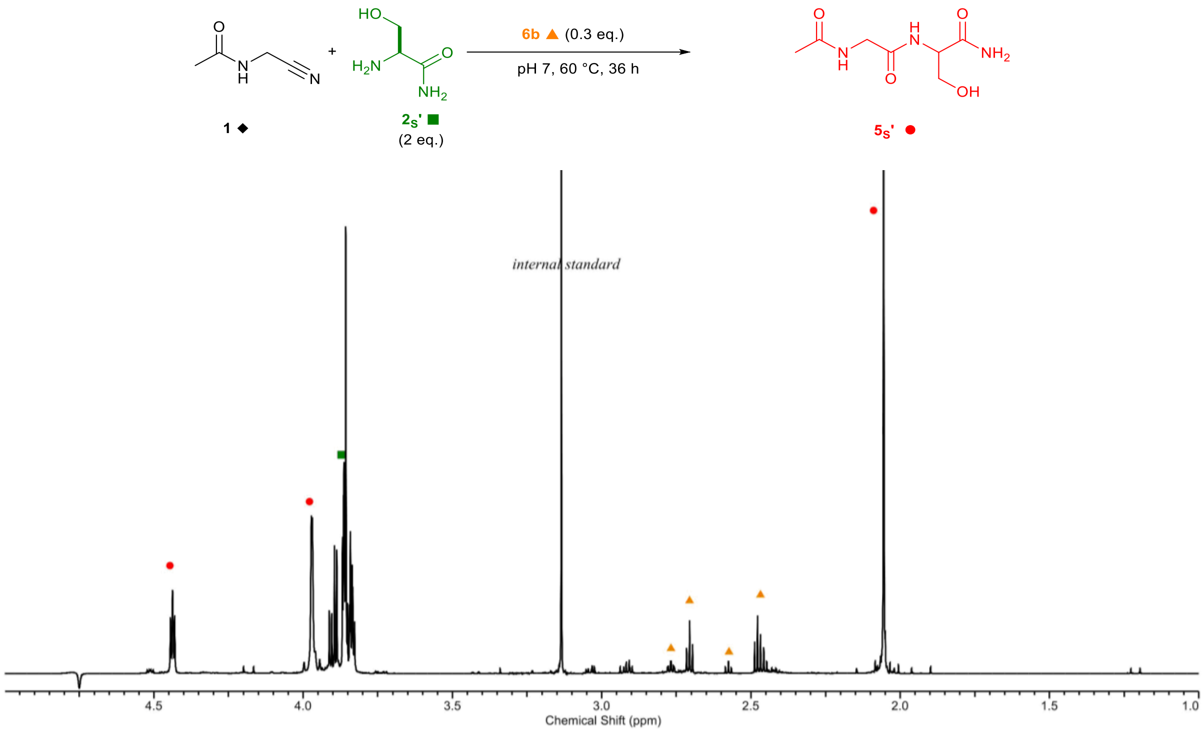

Figure S89: ^{1}H NMR (700 MHz, H_2O/D_2O 9:1, noesygppr1d, 1.0-5.0 ppm) spectrum to show the reaction of N-acetyl glycine nitrile (**1**, 200 mM) with L-serinamide (**2S'**, 400 mM) and 3-mercaptopropanoic acid (**6b**, 60 mM) with MSM (60 mM) as an internal standard at pH 7.0 and 60°C.

^{1}H NMR (700 MHz, H_2O/D_2O 9:1, noesygppr1d) **5S'** (partial assignment) (●): δ_H 4.44 (1H, app. t, *J* = 4.9 Hz, Ser-αH-CONH₂), 3.98 (1H, AB, *J* = 17.4 Hz, AcNHC***H***H), 3.96 (1H, AB, *J* = 17.4 Hz, AcNHCH***H***), 2.06 (3H, s, *H*₃C(CO)).

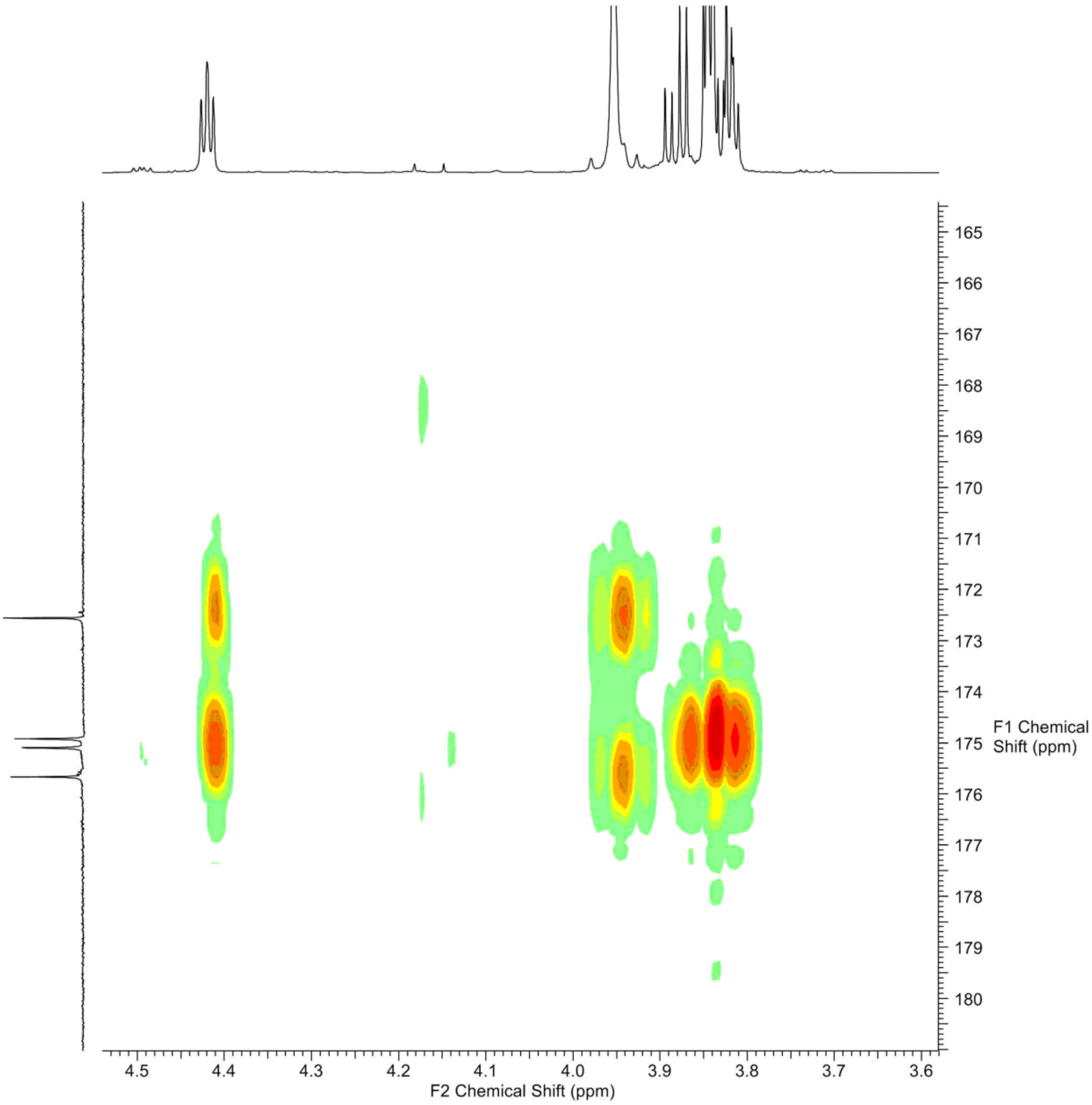

Figure S90: ^1H–^{13}C HMBC (^1H: 700 MHz [3.5–4.5 ppm], ^{13}C: 176 MHz [150-185 ppm], H_2O/D_2O 9:1) spectrum showing the diagnostic $^2J_{CH}$ and $^3J_{CH}$ coupling of the **Ser**-αH in **5s'** at 4.44 ppm with two resonances at 175.2 and 172.6 ppm which is characteristic of amide bond formation.

*Coupling of N-acetylglycine nitrile **1** with L-tryptophan amide **2**$_{W}$**'** at pH 8.5 and room temperature*

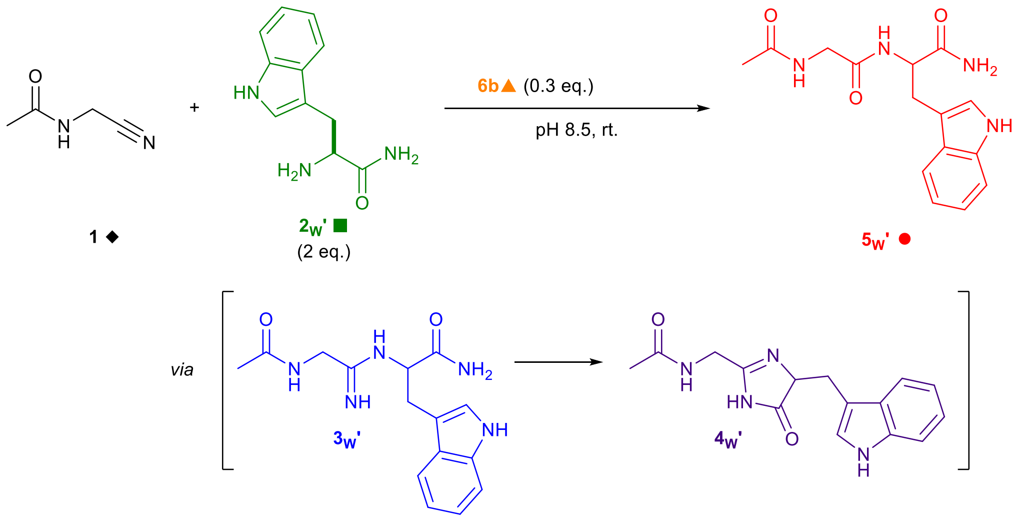

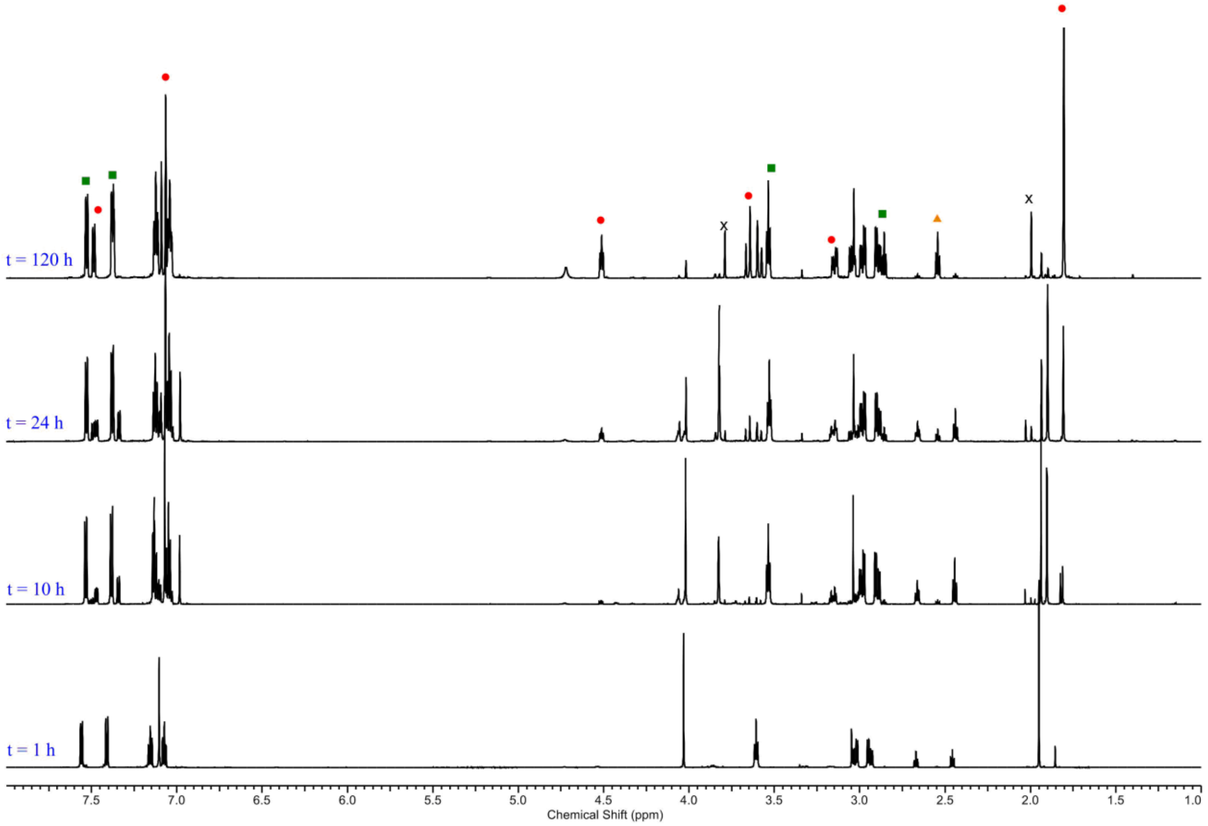

Figure S91: ^{1}H NMR (700 MHz, H_2O/D_2O 9:1, noesygppr1d, 1.0-8.0 ppm) spectrum to show the reaction of N-acetyl glycine nitrile (**1**, 200 mM) with L-tryptophan amide (**Trp**, 200 mM) and 3-Mercaptopropanoic acid (MPA, 60 mM) with MSM (7 mM) as an internal standard at pH 8.5 and room temperature.

^{1}H NMR (700 MHz, H_2O/D_2O 9:1, noesygppr1d) **5**$_{W}$**'** (partial assignment) (●): δ_H 7.52 (1H, d, *J* = 8.1 Hz, Ar***H***), 7.12 (1H, s, Ar***H***), 4.54 (1H, app. t, *J* = 6.8 Hz, Trp-***αH***-COOH), 3.67 (1H, AB, *J* = 16.9 Hz, AcNHCH***H***), 3.62 (1H, AB, *J* = 16.9 Hz, AcNHC***H***H), 3.17 (1H, ABX, *J* = 14.8, 5.8 Hz, CHCH***H***Ar), 3.07 (1H, ABX, *J* = 14.8, 7.9 Hz, CHCH***H***Ar), 1.83 (3H, s, ***H***$_3$C(CO)); *L-tryptophan amide* **2**$_{W}$**'** (partial assignment) (■): δ_H 7.55 (1H, d, *J* = 7.9 Hz, Ar***H***), 7.09 (1H, s, Ar***H***),), 3.56 (1H, app. t, *J* = 6.6 Hz ***αH***-COOH), 3.01 (1H, ABX, *J* = 14.4, 6.3 Hz, CHCH***H***Ar), 2.92 (1H, ABX, *J* = 14.4, 6.8 Hz, CHC***H***HAr); *N-Acetylglycinamide*, **7** (✖) δ_H 3.82 (2H, s, C*H*$_2$), 2.02 (3H, s, ***H***$_3$C(CO)).

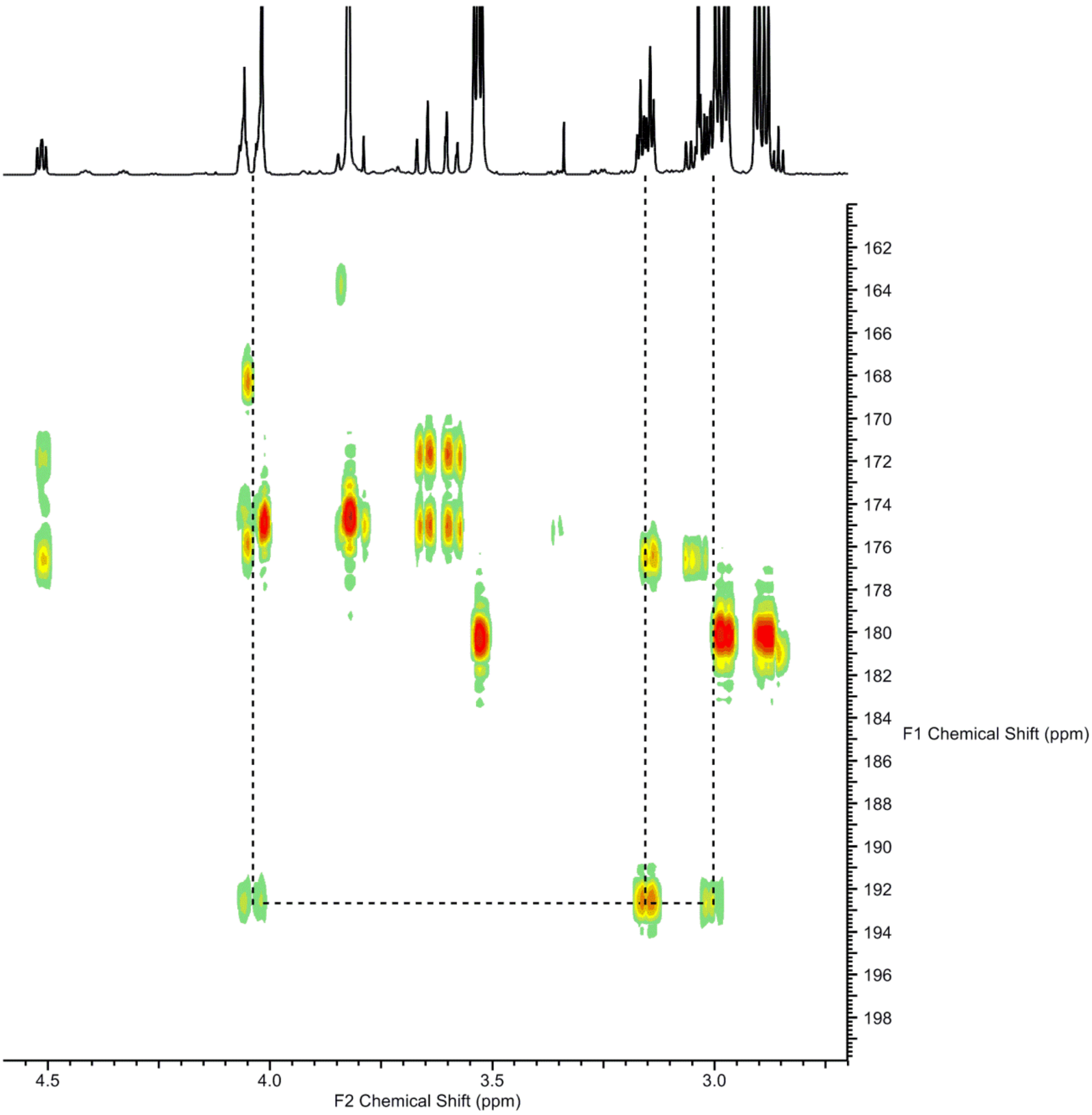

Figure S92: ^1H–^{13}C HMBC (^1H: 700 MHz [2.7-4.6 ppm], ^{13}C: 176 MHz [160-200 ppm], H_2O/D_2O 9:1) spectrum for the coupling of **1** and **2_W'** catalyzed by 3-mercaptopropionic acid (**6b**) at pH 8.5 after 24 h, showing the diagnostic coupling of the imidazolone amide signal in **4_W** at 192.4 ppm with signals at 4.00 ppm (**Trp**-αH, $^2J_{CH}$), 3.15 and 3.00 ppm (**Trp**-βH, $^3J_{CH}$), which is characteristic of imidazolone formation.

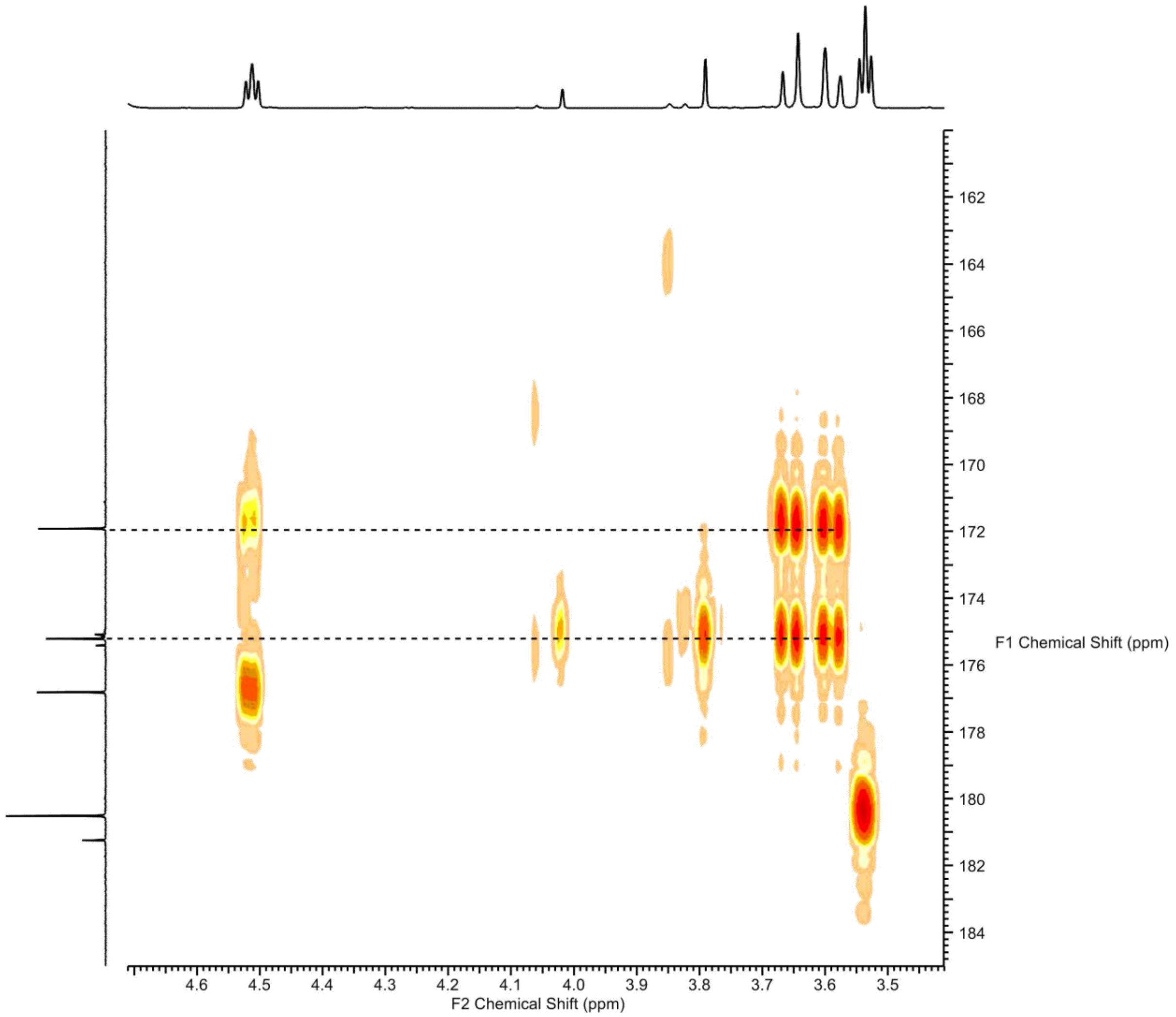

Figure S93: ^1H–^{13}C HMBC (^1H: 700 MHz [3.4–4.7 ppm], ^{13}C: 176 MHz [160–185 ppm], H_2O/D_2O 9:1) spectrum showing the diagnostic $^2J_{CH}$ and $^3J_{CH}$ coupling of the diastereotopic glycyl AB in **5w'** at 3.67 and 3.62 ppm with two resonances at 175.2 and 172.0 ppm, which is characteristic of amide bond formation.

*Coupling of N-acetylglycine nitrile **1** with L-tyrosinamide **2**$_{Y}$**'** at pH 8.5 and room temperature*

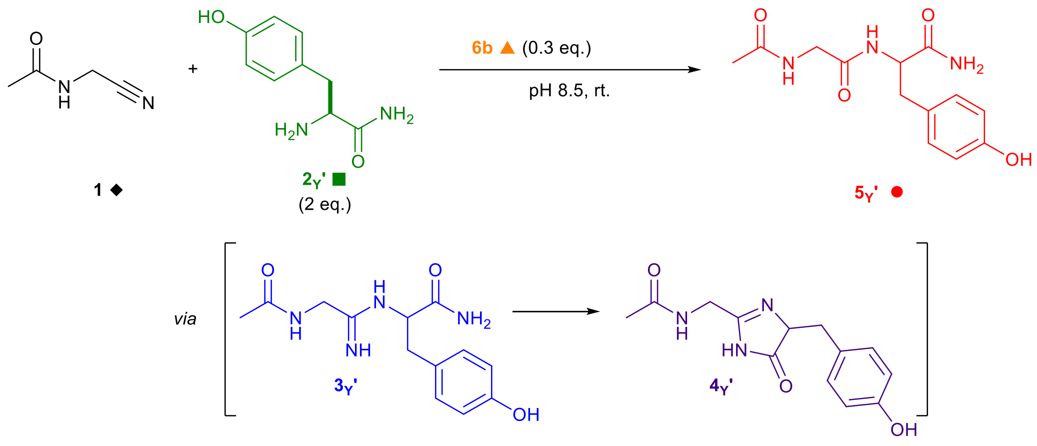

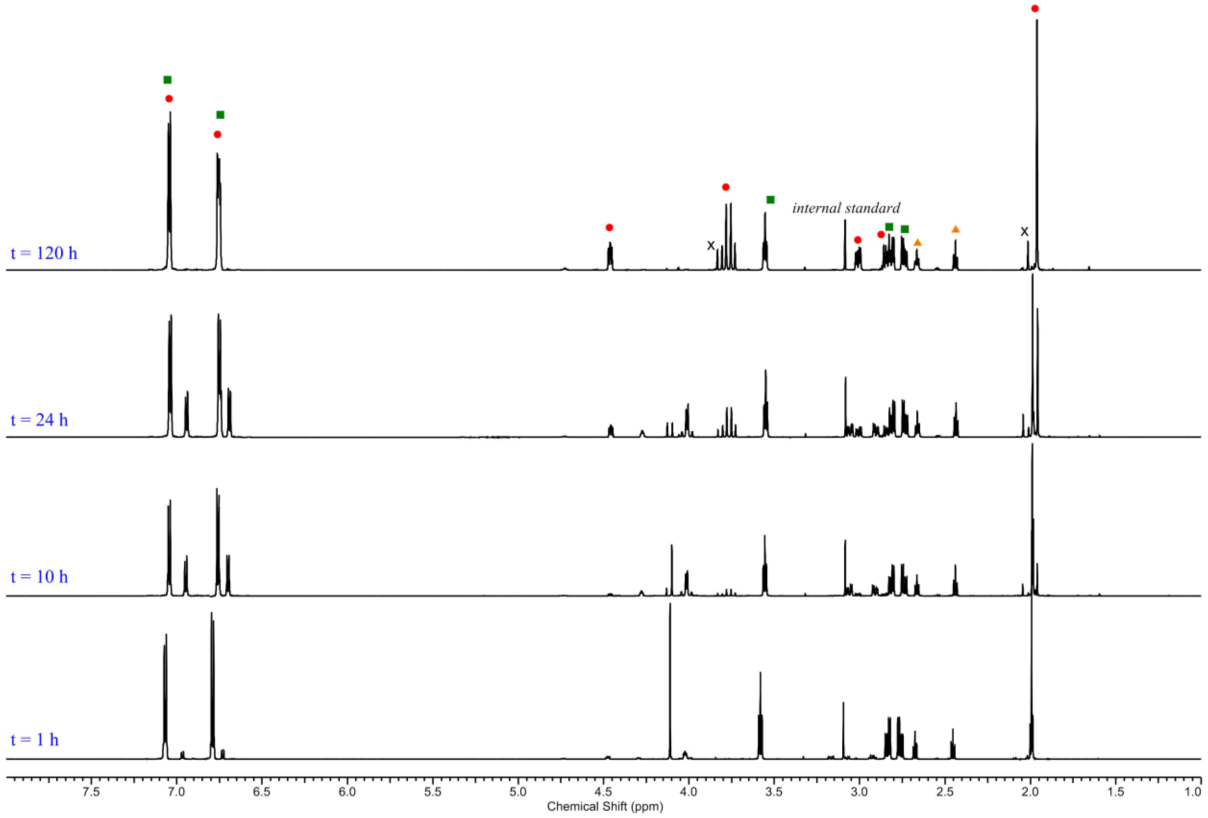

Figure S94: ^{1}H NMR (700 MHz, H_2O/D_2O 9:1, noesygppr1d, 1.0-8.0 ppm) spectrum to show the reaction of N-acetyl glycine nitrile (**1**, 200 mM) with L-tyrosinamide (**2**$_{Y}$**'** 200 mM) and 3-Mercaptopropanoic acid (MPA, 60 mM) with MSM (7 mM) as an internal standard at pH 8.5 and room temperature.

^{1}H NMR (700 MHz, H_2O/D_2O 9:1, noesygppr1d) **5**$_{Y}$**'** (partial assignment) (●): δ_H 4.49 (1H, dd, *J* = 7.9, 6.3 Hz, Tyr-***αH***-CONH$_2$), 3.82 (1H, AB, *J* = 16.8 Hz AcNHCH***H***), 3.77 (1H, AB, *J* = 16.9 Hz AcNHC***H***H), 3.04 (1H, ABX, *J* = 14.1, 5.8 Hz, CHCH***H***Ar), 1.99 (3H, s, ***H***$_3$C(CO)); *L-tyrosinamide*, **2**$_{Y}$**'** (partial assignment) (■): δ_H 3.58 (1H, app. t, *J* = 6.7 Hz ***αH***-COOH), 2.76 (1H, ABX, *J* = 13.9, 7.0 Hz, CHC***H***HAr); *N-acetylglycinamide*, **7** (✖) δ_H 3.86 (2H, s, C*H*$_2$), 2.04 (3H, s, ***H***$_3$C(CO)).

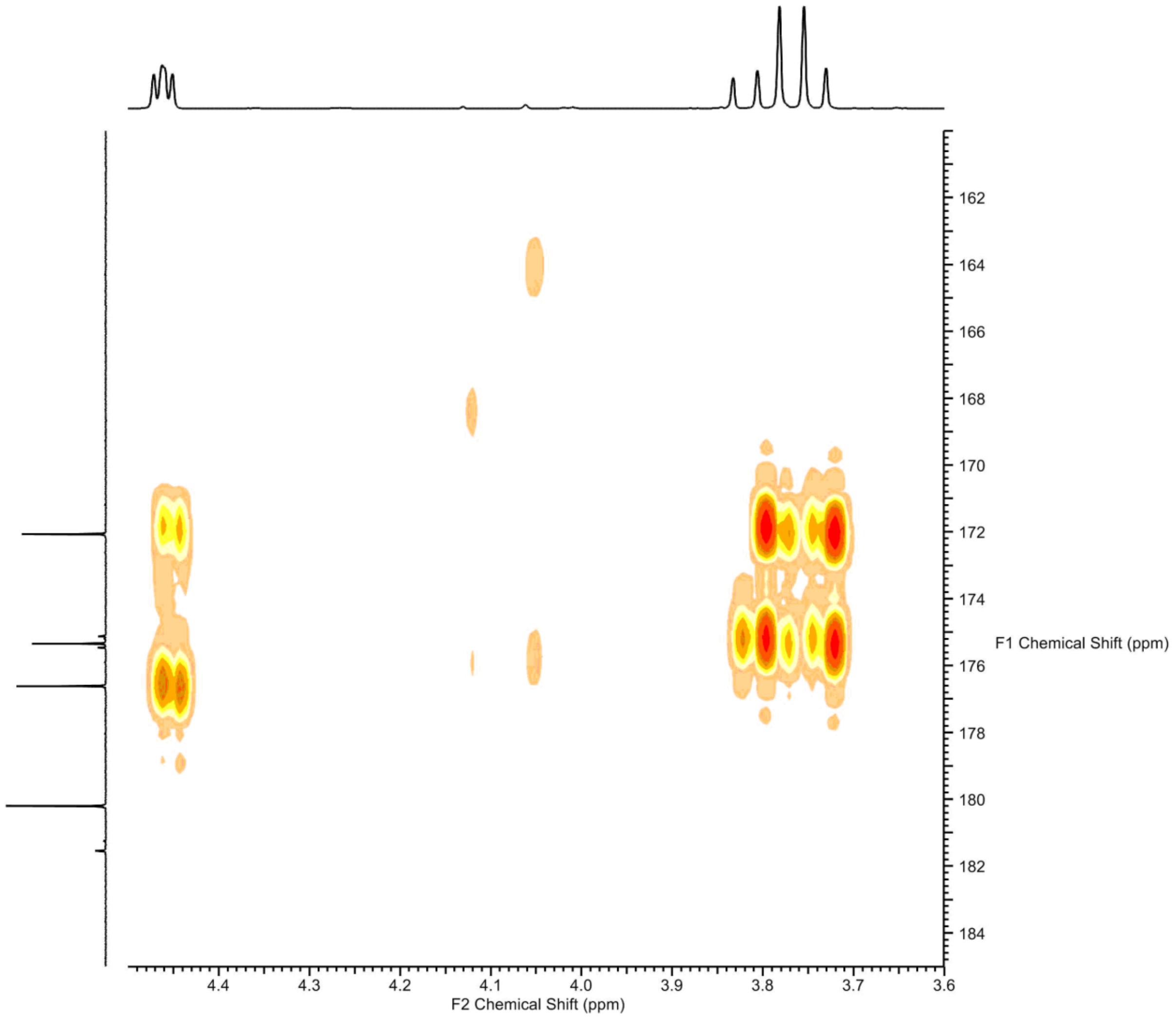

Figure S95: ^{1}H–^{13}C HMBC (^{1}H: 700 MHz [3.6–4.5 ppm], ^{13}C: 176 MHz [160–185 ppm], H_2O/D_2O 9:1) spectrum showing the diagnostic $^{2}J_{CH}$ and $^{3}J_{CH}$ coupling of the diastereotopic glycyl AB in **5Y'** at 3.82 and 3.77 ppm with two resonances at 175.2 and 172.0 ppm, which is characteristic of amide bond formation.

*Coupling of N-acetylglycine nitrile **1** with L-threoninamide **2T'** at pH 8.5 and room temperature*

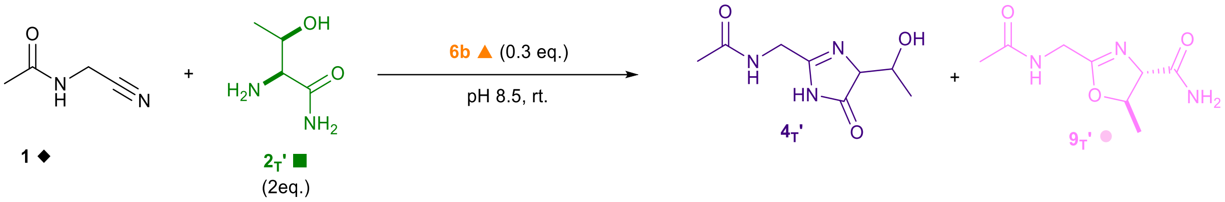

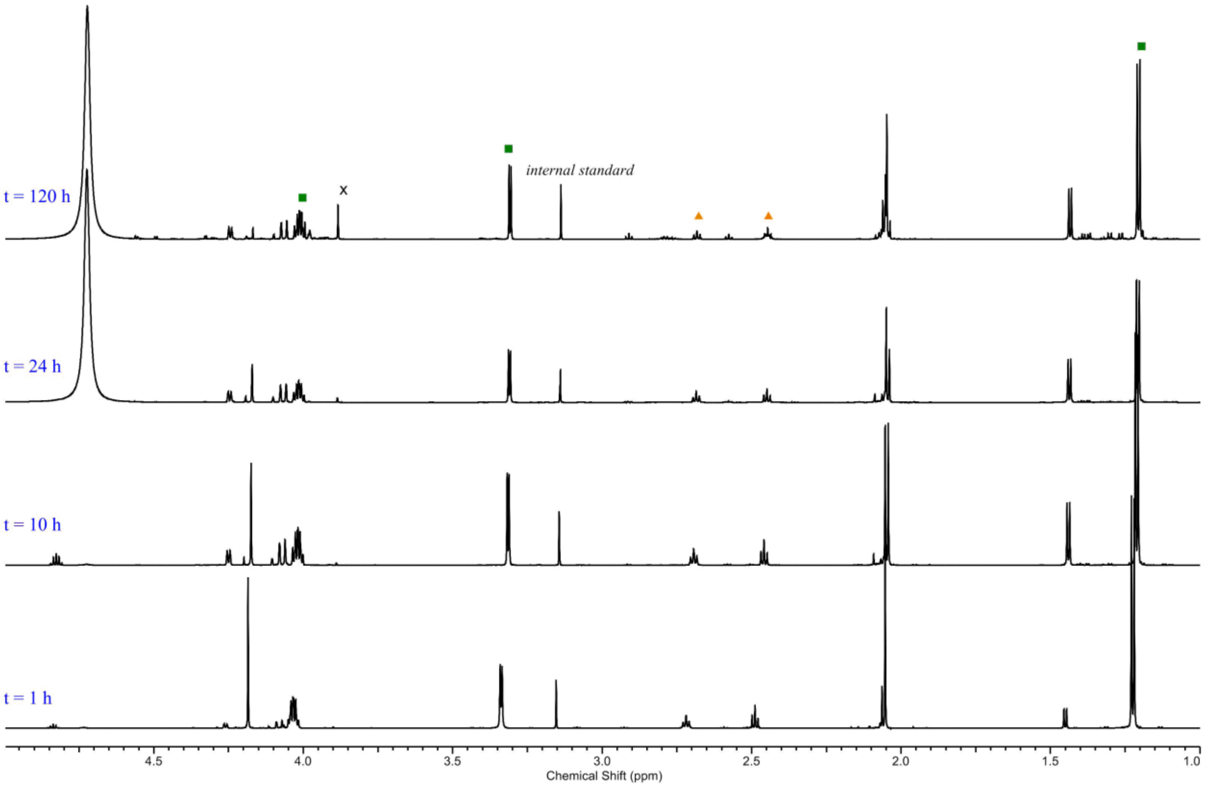

Figure S96: ^{1}H NMR (700 MHz, H_2O/D_2O 9:1, noesygppr1d, 1.0–4.7 ppm) spectrum to show the reaction of N-acetyl glycine nitrile (**1**, 200 mM) with L-threoninamide (**2T'**, 240 mM) and 3-Mercaptopropanoic acid (**6b**, 60 mM) with MSM (7 mM) as an internal standard at pH 8.5 and room temperature.

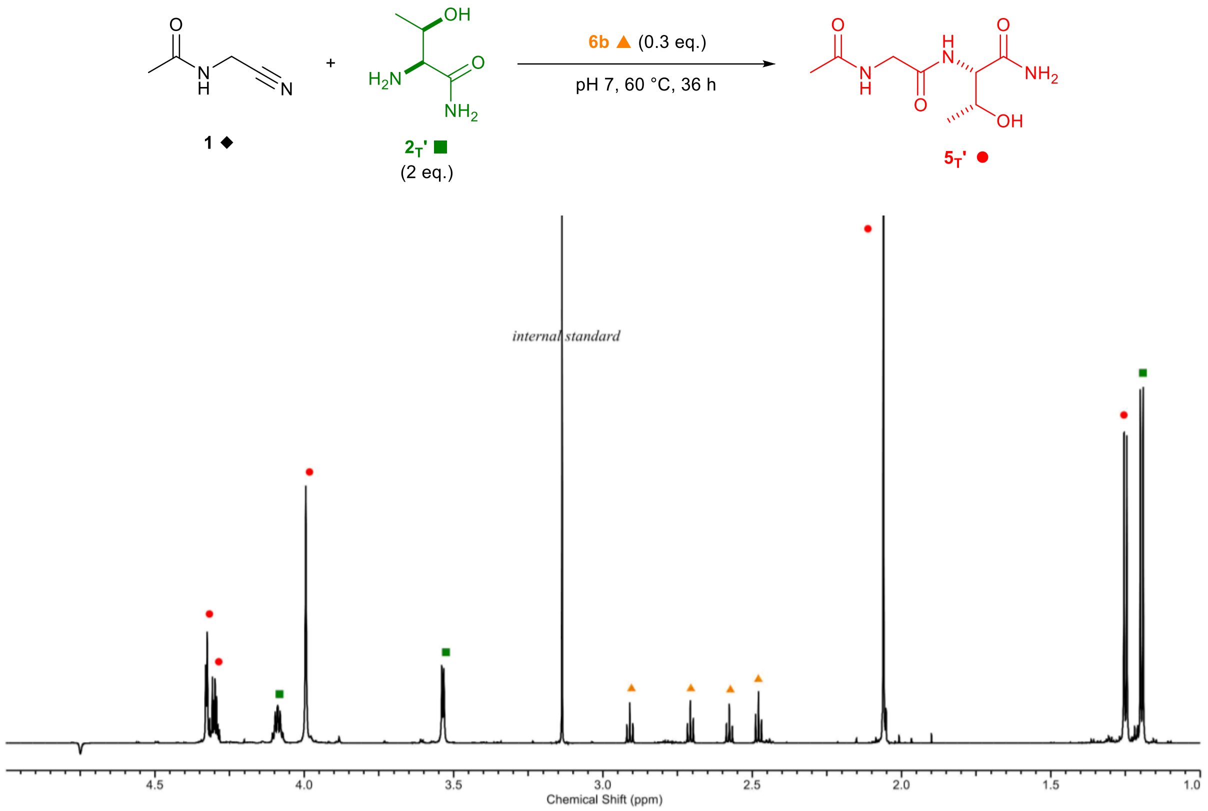

Figure S97: ^{1}H NMR (700 MHz, H_2O/D_2O 9:1, noesygppr1d, 1.0-5.0 ppm) spectrum to show the reaction of N-acetyl glycine nitrile (**1**, 200 mM) with L-threoninamide (**2S'**, 400 mM) and 3-mercaptopropanoic acid (**6b**, 60 mM) with MSM (60 mM) as an internal standard at pH 7.0 and 60 °C.

^{1}H NMR (700 MHz, H_2O/D_2O 9:1, noesygppr1d) **5_T'** (●): δ_H 4.34-4.28 (2H, m, Thr-***αH***-CONH$_2$ + HOC***H***CH$_3$), 4.00 (2H, s, AcNHC*H$_2$*), 2.06 (3H, s, *H$_3$*C(CO)), 1.25 (3H, d, *J* = 6.3 Hz, HOCHC*H$_3$*); *L-threoninamide*, **2_T'** (■): δ_H 4.09 (1H, m, HOC*H*CH$_3$), 3.54 (1H, d, *J* = 4.9 Hz, αH-COOH), 1.20 (3H, d, *J* = 6.3 Hz, HOCHC*H$_3$*).

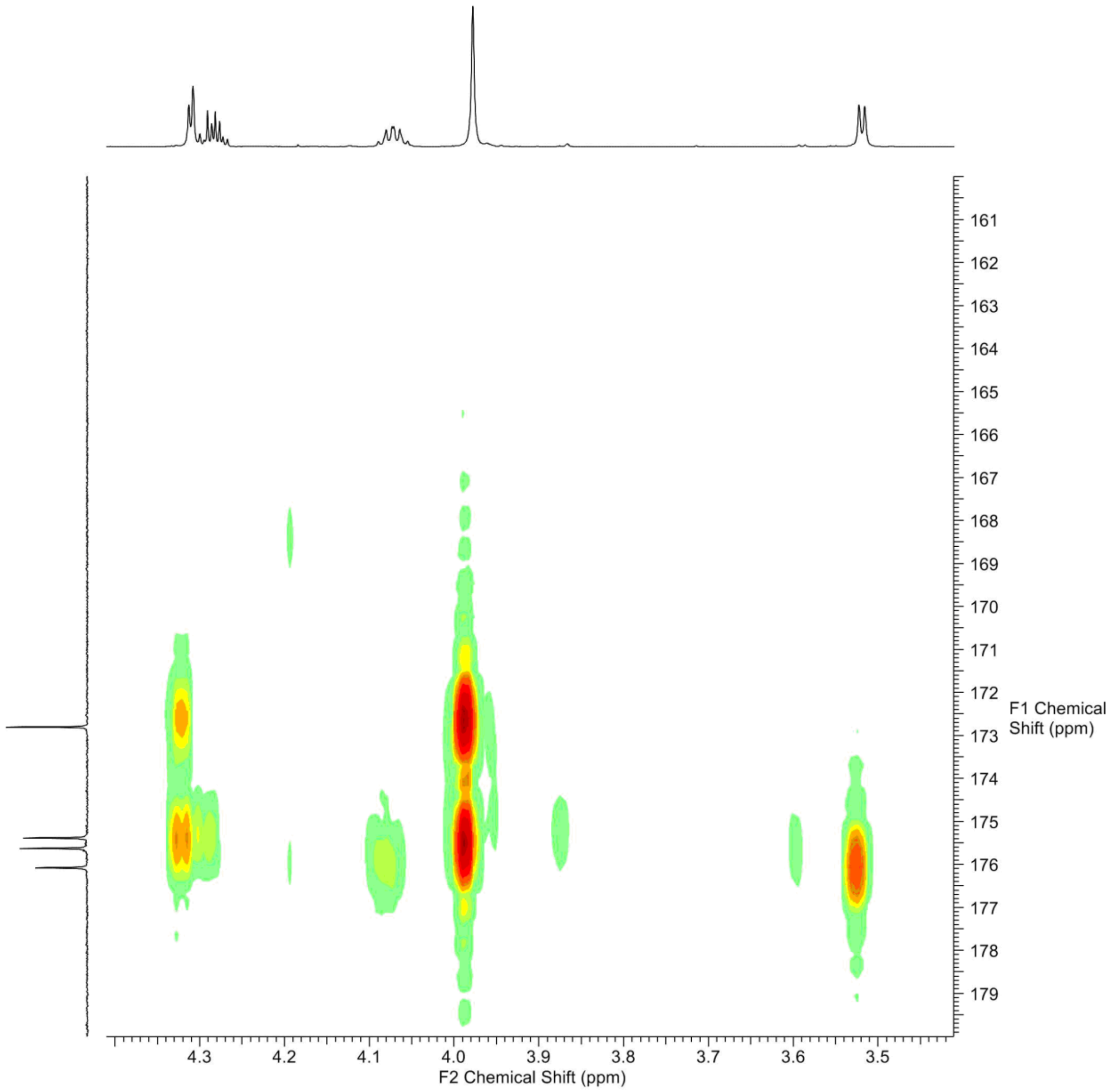

Figure S98: ^1H–^{13}C HMBC (^1H: 700 MHz [3.4-4.4 ppm], ^{13}C: 176 MHz [160–180 ppm], H_2O/D_2O 9:1) spectrum showing the diagnostic $^2J_{CH}$ and $^3J_{CH}$ coupling of the **Thr**-αH in **5T'** at 4.32 ppm with two resonances at 175.6 and 172.6 ppm, which is characteristic of amide bond formation.

Coupling of N-acetylglycine nitrile ***1*** *with L-valinamide* ***2v'*** *at pH 8.5 and room temperature*

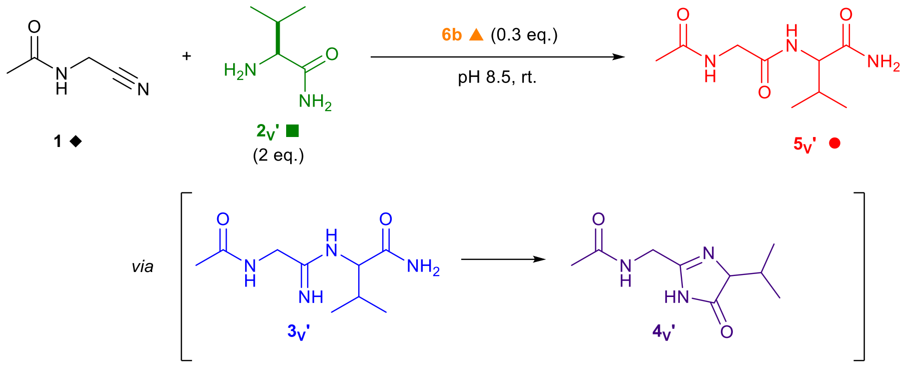

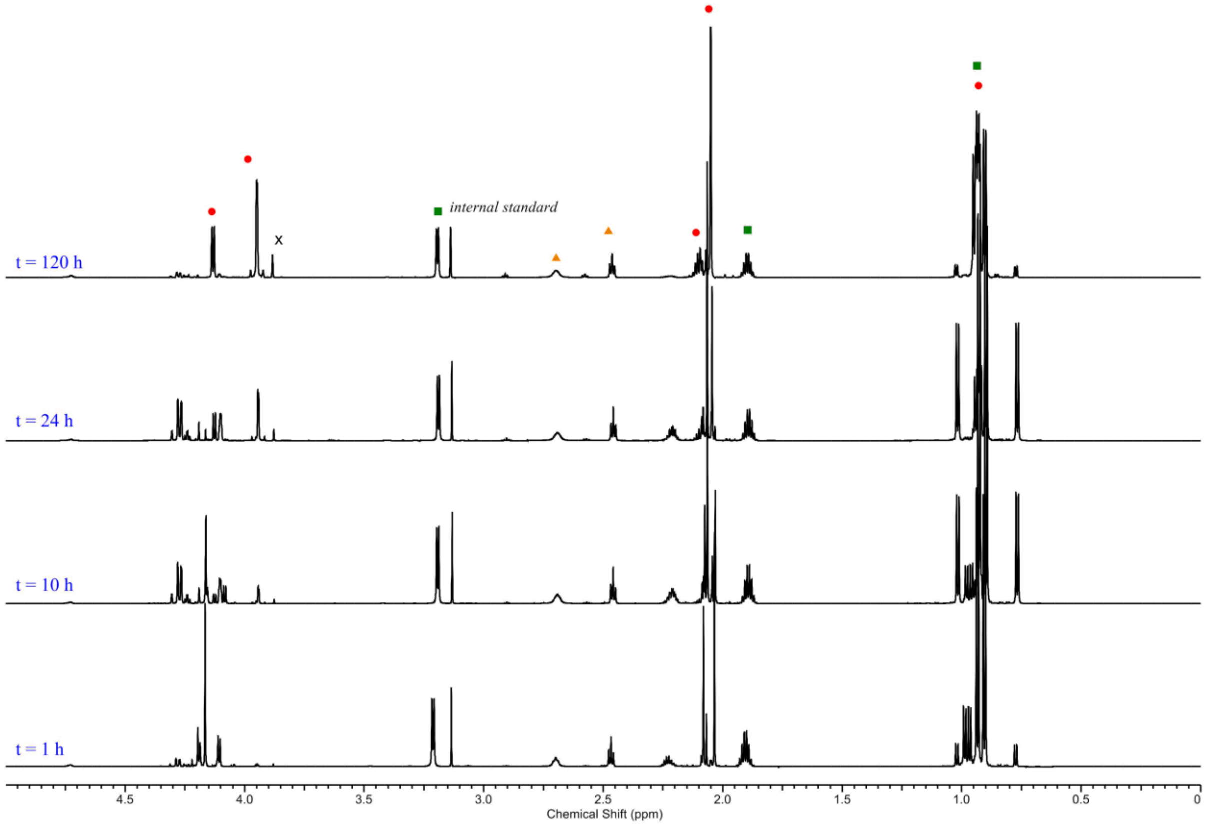

Figure S99: ^{1}H NMR (700 MHz, H_2O/D_2O 9:1, noesygppr1d, 0.0–5.0 ppm) spectrum to show the reaction of N-acetyl glycine nitrile (**1**, 200 mM) with L-valinamide (**2v'**, 400 mM) and 3-mercaptopropanoic acid (**6b**, 60 mM) with MSM (7 mM) as an internal standard at pH 8.5 and room temperature.

^{1}H NMR (700 MHz, H_2O/D_2O 9:1, noesygppr1d) **5v'** (partial assignment) (●): δ_H 4.16 (1H, d, *J* = 6.7 Hz, Val-***αH***-CONH$_2$), 3.98 (1H, AB, *J* = 17.2 Hz, AcNHC***H***H), 3.97 (1H, AB, *J* = 17.2 Hz, AcNHCH***H***), 2.15-2.10 (1H, m, H$_3$CC***H***CH$_3$), 2.07 (3H, s, *H*$_3$C(CO)); *L-valinamide*, **2v'** (■): δ_H 3.22 (1H, d. *J* = 6.1 Hz, ***αH***-CONH$_2$), 1.92 (1H, app. octet, *J* = 6.7 Hz, H$_3$CC***H***CH$_3$); *N-acetylglycinamide*, **7** (✖) (partial assignment): δ_H 3.91 (2H, s, C*H*$_2$)

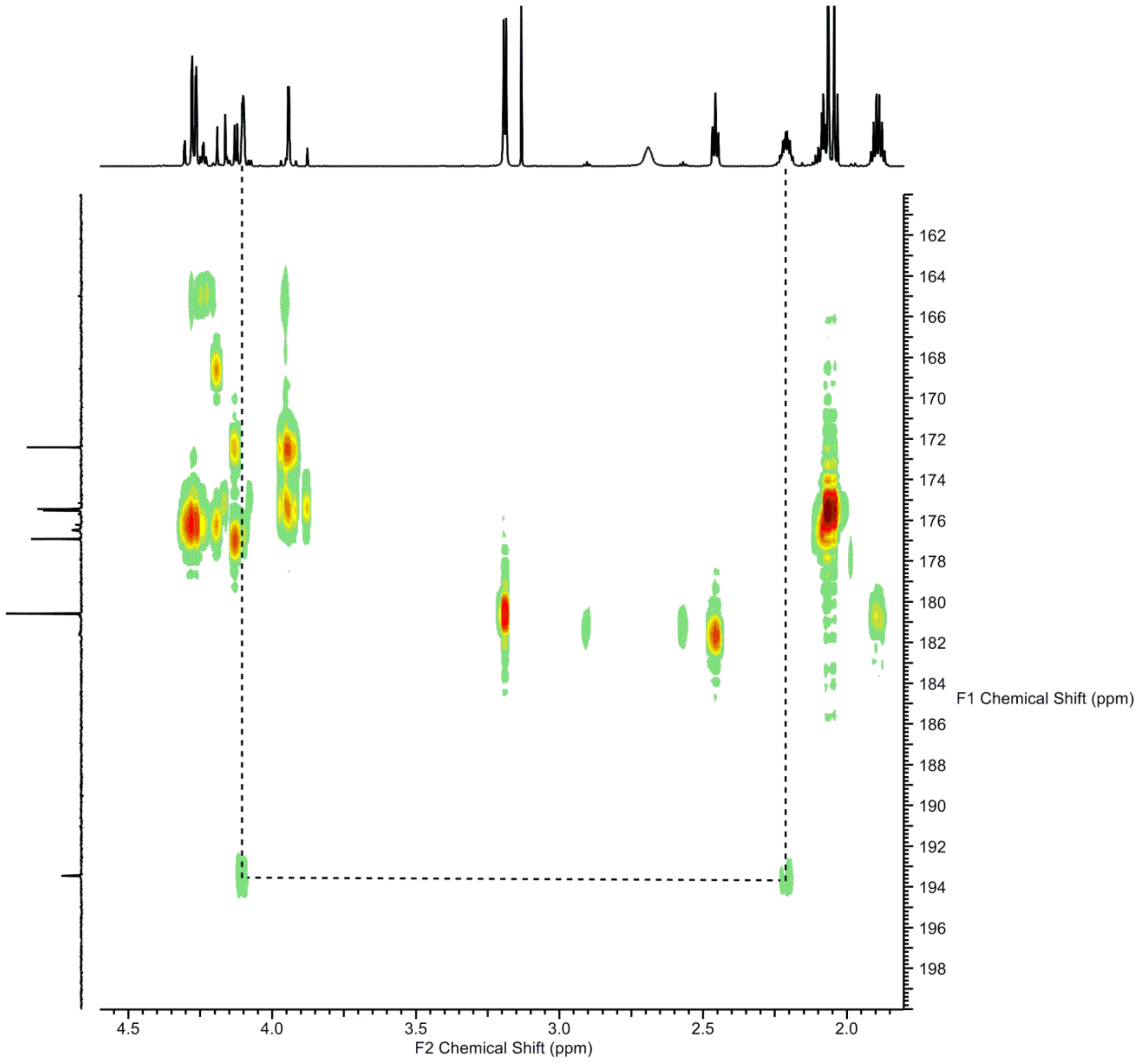

Figure S100: ^1H–^{13}C HMBC (^1H: 700 MHz [1.4-4.6 ppm], ^{13}C: 176 MHz [160-200 ppm], H_2O/D_2O 9:1) spectrum for the coupling of **1** and **2v'** catalyzed by 3-mercaptopropionic acid (**6b**) at pH 8.5 after 24 h, showing the diagnostic coupling of the imidazolone amide signal in **4v** at 193.4 ppm with signals at 4.10 ppm (**Val**-αH, $^2J_{CH}$), 2.22 ppm (**Val**-βH, $^3J_{CH}$), which is characteristic of imidazolone formation.

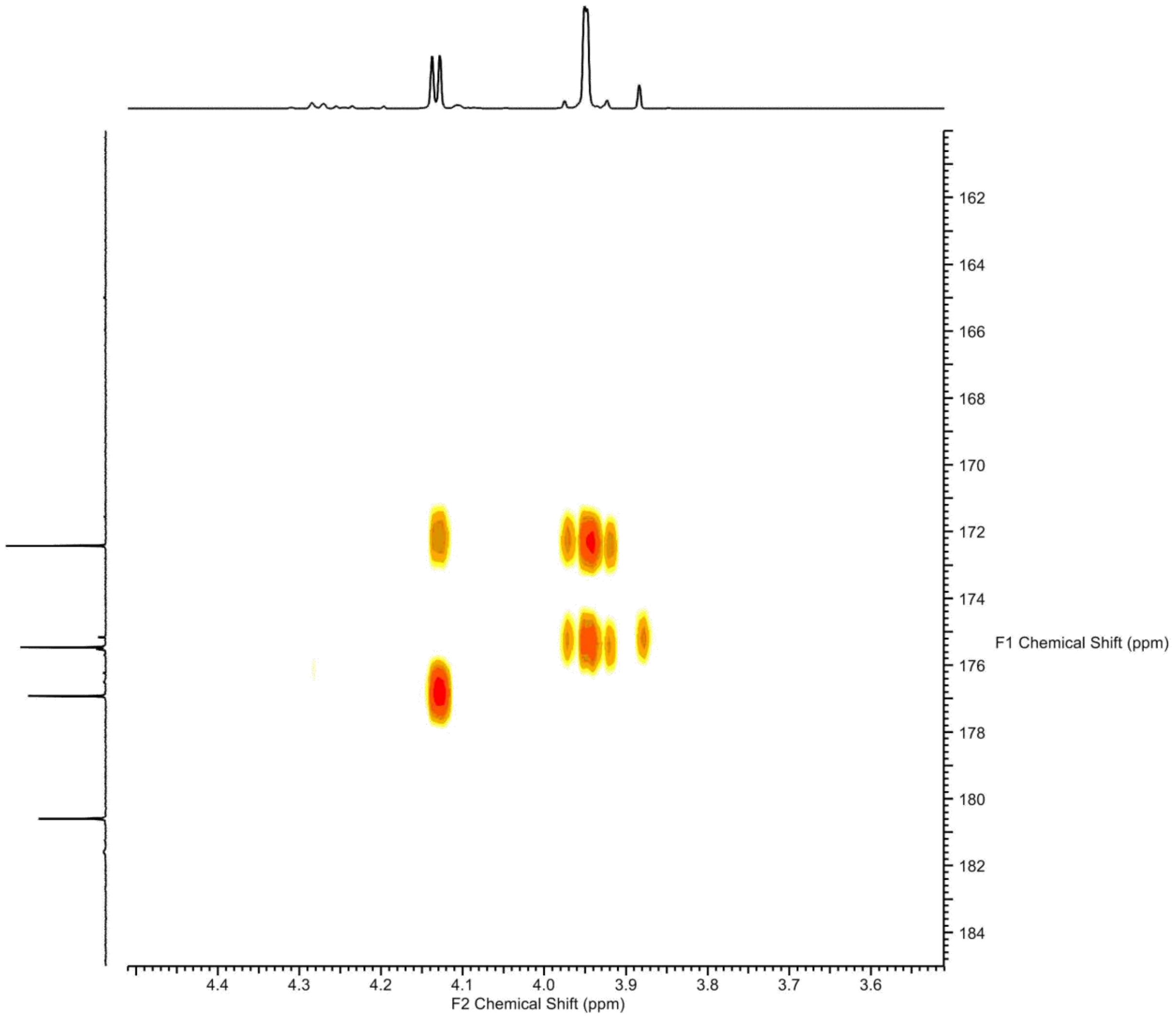

Figure S101: ^{1}H–^{13}C HMBC (^{1}H: 700 MHz [3.2-4.5 ppm], ^{13}C: 176 MHz [150–185 ppm], H_2O/D_2O 9:1) spectrum showing the diagnostic $^{2}J_{CH}$ and $^{3}J_{CH}$ coupling of the **Val**-αH in **5v'** at 4.16 ppm with two resonances at 177.2 and 172.6 ppm, which is characteristic of amide bond formation.

*Coupling of **1** with peptide fragments catalyzed by **6b***

*Coupling of **1** with L-alanylglycyl-L-alanine **2**$_{AGA}$*

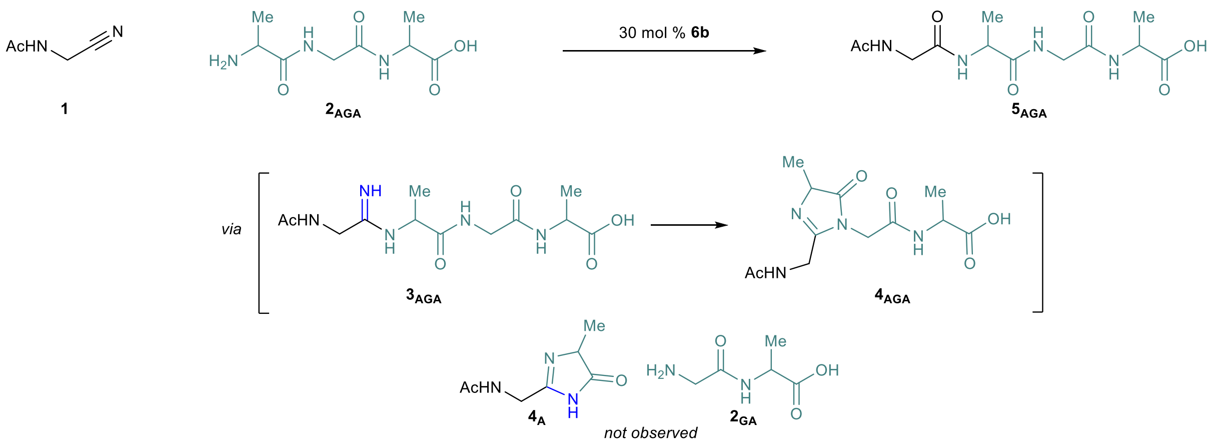

Entry	pH	temperature / °C	reaction time / h	**4**$_{AGA}$ / %	**5**$_{AGA}$ / %
1	8.5	20	12	38	trace
2	8.5	20	144	12	34
3	8.5	60	28	0	50
4	7.0	60	96	0	81

Table S21: Thiol-catalyzed ligation of **1** with Ala-Gly-Ala **2**$_{AGA}$.

Entry	pH	buffer	temperature / °C	Time	**12** / %	**7** / %	**5**$_{AGA}$ / %
1	7	no buffer	60	96 hour	5	14	81
2	7	no buffer	20	20 days	5	12	78
3	8.5	no buffer	60	28 hours	overlapped	30	50
4	8.5	1 M borate	20	30 days	overlapped	25	64

Table S22: Thiol-catalyzed ligation of **1** with Ala-Gly-Ala **2**$_{AGA}$ at pH 7 and 8.5 at different temperature.

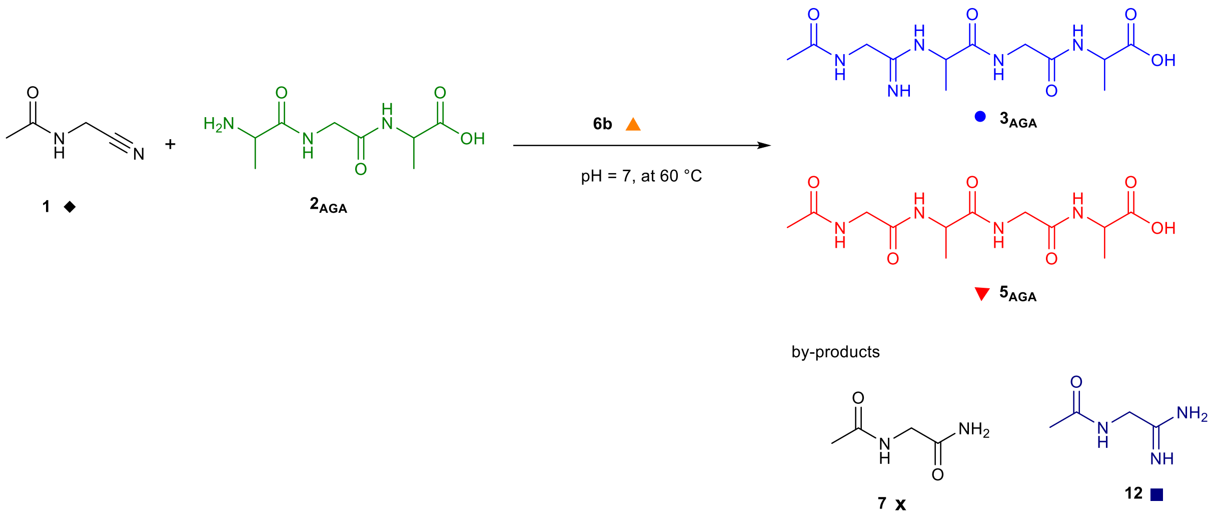

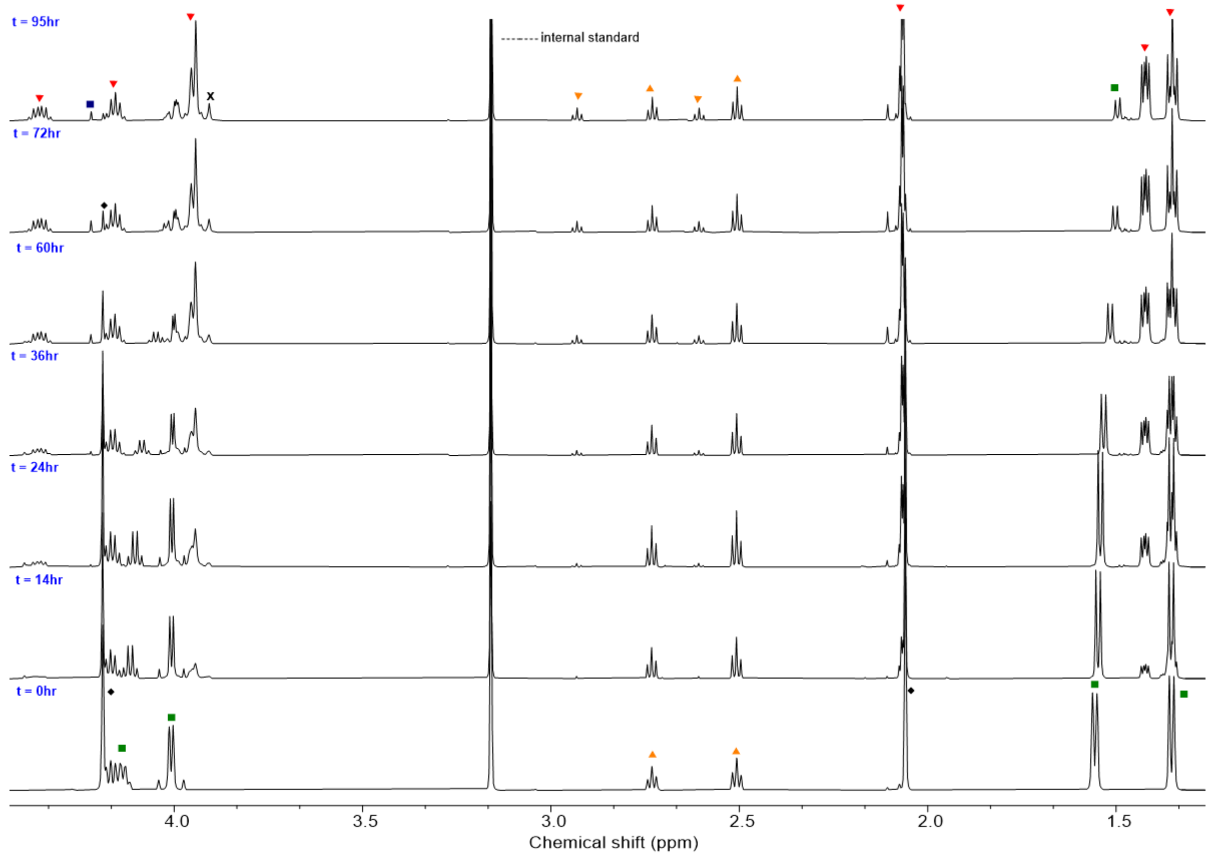

Figure S102: ^{1}H NMR (600 MHz, H_2O/D_2O 9:1, noesygppr1d, 1.0-5.0 ppm) spectrum to show the reaction of N-acetyl glycine nitrile (**1**; 100 mM), L-alanylglycyl-L-alanine (**2**$_{AGA}$; 100 mM) and 3-mercaptopropionic acid (**6b**; 60 mM) with MSM (100 mM) as an internal standard in H_2O (1 mL) at pH 7 at 60°C.

N-Acetylglycyl-DL-alanylglycyl-DL-alanine, ***5****$_{AGA}$* (2 diastereoisomers) (▼) : ^{1}H NMR (600 MHz, H_2O) δ 4.35 - 4.28 (m, 1H, Ala-(C2)–H), 4.15 - 4.08 (m, 1H, Ala-(C2)–H), 3.98 - 3.93 (m, 4H, 2 × Gly-(C2)–H), 2.03 (s, 3H, $COCH_3$),1.38 - 1.35 (m, 3H, Ala-(C3)–H_3), 1.31 - 1.29 (m, 3H, Ala-(C3)–H_3); *N-acetylglycinamide*, **7** (✖) (partial assignment): $δ_H$ 3.91 (2H, s, CH_2), 2.10 (3H, s, CH_3), **12** (■): $δ_H$ 4.21 (2H, s, $AcNHCH_2$)

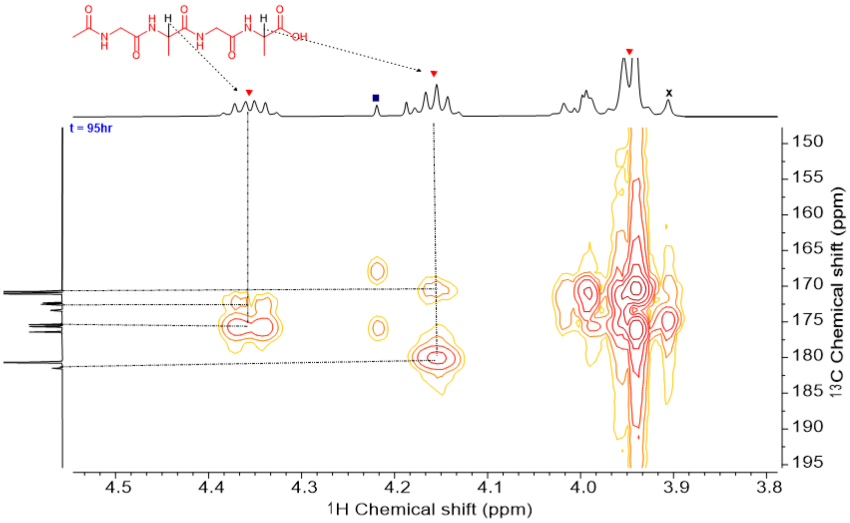

Figure S103: ^{1}H–^{13}C HMBC (^{1}H: 600 MHz [3.80–4.40 ppm], ^{13}C: 176 MHz [160-185 ppm]) spectrum showing the diagnostic $^{2}J_{CH}$ and $^{3}J_{CH}$ coupling of **Ala$_2$**-(C2)–H and **Ala$_4$**-(C2)–H in a diastereoisomeric mixture of **5$_{AGA}$** (α_H = 4.31 ppm, δ_c = 175.8, 175.61, 172.7, 171.2; α_H = 4.15 ppm, δ_c 170.7 (× 2), 179.9, 180.4 ppm.

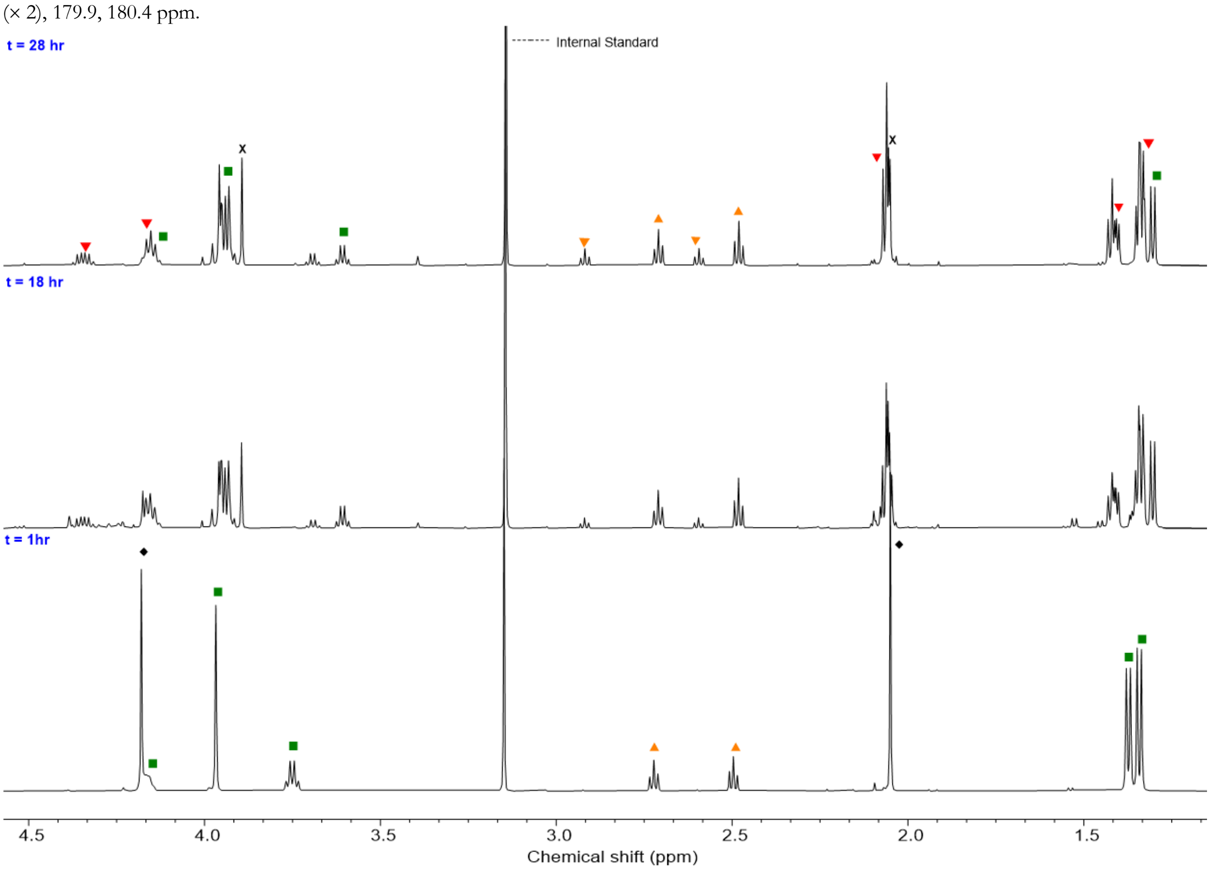

Figure S104: ^{1}H NMR (600 MHz, H_2O/D_2O 9:1, noesygppr1d, 1.0-5.0 ppm) spectrum to show the reaction of N-acetyl glycine nitrile(**1**; 100 mM), L-alanylglycyl-L-alanine (**2$_{AGA}$**; 100 mM) and 3-mercaptopropionic acid (**6b**; 60 mM) with MSM (50 mM) as an internal standard in H_2O (1 mL) at pH 8.5 at 60°C.

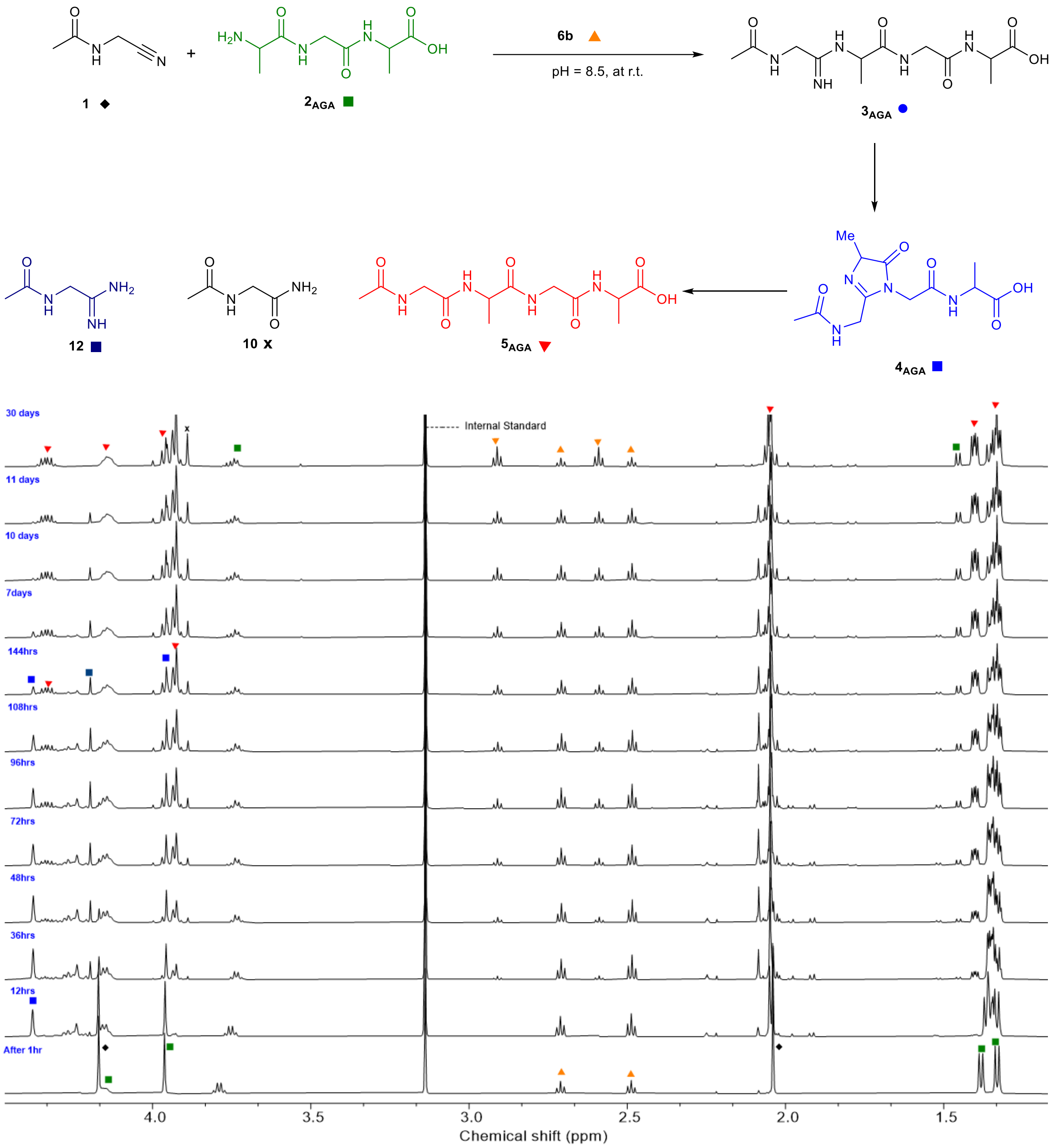

Figure S105: ^{1}H NMR (600 MHz, H_2O/D_2O 9:1, noesygppr1d, 1.3-4.5 ppm) spectrum to show the reaction of N-acetylglycine nitrile(**1**; 100 mM), L-alanylglycyl-L-alanine (**2**$_{\mathbf{AGA}}$; 130 mM), 3-mercaptopropionic acid (**6b**; 60 mM) with MSM (50 mM) as an internal standard at pH 8.5 at room temperature in borate buffer (1 M, 1 mL).

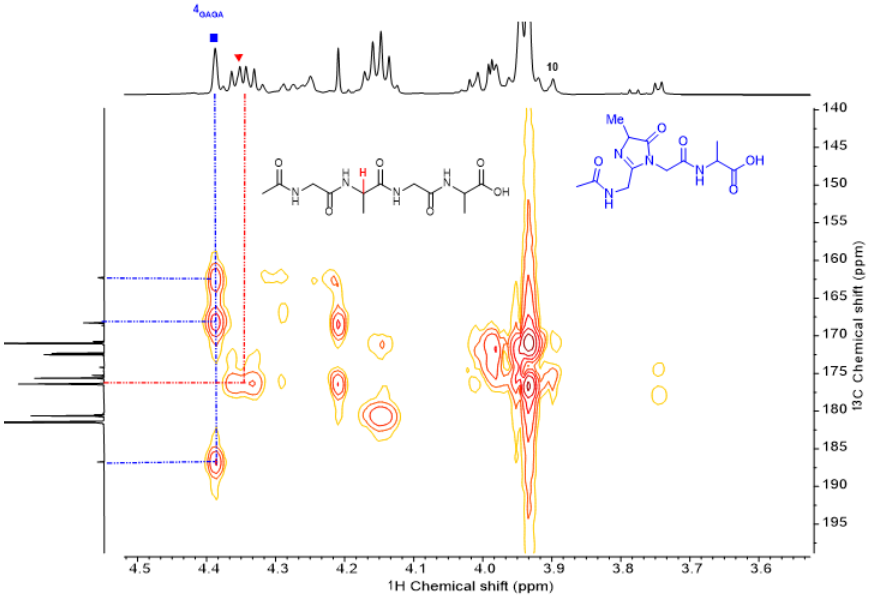

Figure S106: ^{1}H–^{13}C HMBC (^{1}H: 600 MHz [3.80–4.40 ppm], ^{13}C: 176 MHz [160-185 ppm]) spectrum of the coupling between N-acetyl glycine nitrile **1** and H-Ala-Gly-Ala-OH **2**$_{\mathbf{AGA}}$ catalysed by **6b** after 12 h showing the diagnostic for **4**$_{\mathbf{AGA}}$ imidazolone α_H = 4.39 ppm, δ_c 162.3, 168.4, 186.9 ppm.

*Coupling of **1** with L-alanyl-glycine **2_{AG}***

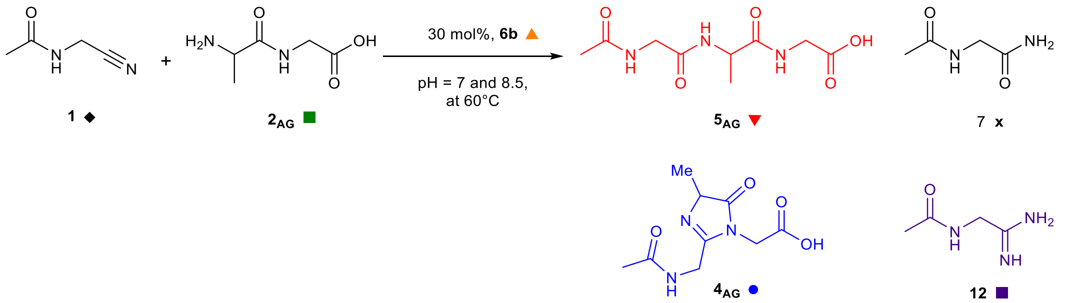

Entry	pH	2_{AG}	Temp / °C	7 / %	5_{AG} / %
1	7	H-Ala-Gly-OH	60	10	86
2	8.5	H-Ala-Gly-OH	60	25	74

Table S23: The effect of pH on thiol-catalyzed ligation of **1** with Ala-Gly $\mathbf{2_{AG}}$

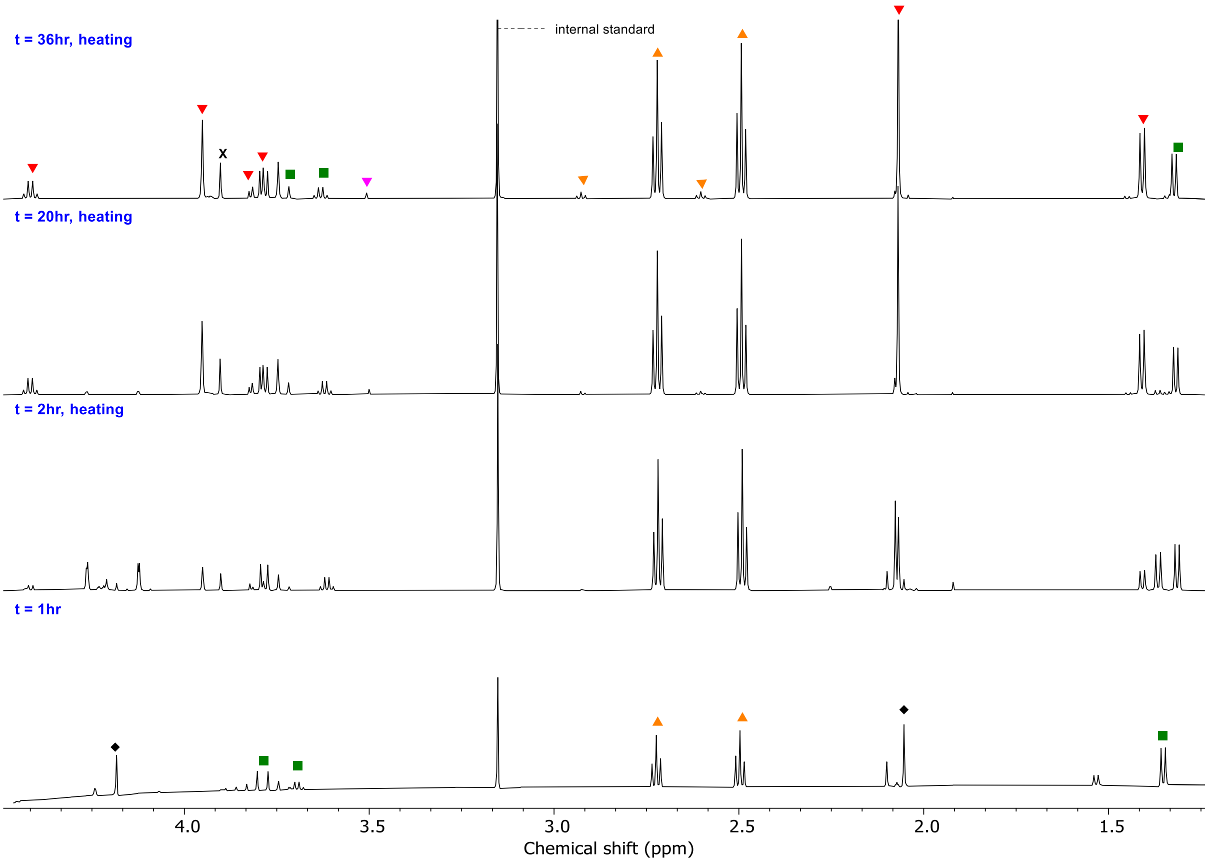

Figure S107: ^{1}H NMR (600 MHz, H2O/D2O 9:1, noesygppr1d, 1.3-4.5 ppm) spectrum to show the reaction of N-acetylglycine nitrile (**1**; 100 mM), *L*-alanyl-glycine ($\mathbf{2_{AG}}$; 130 mM), 3-mercaptopropionic acid (**6b**; 60 mM) with MSM (50 mM) as an internal standard in H2O (1 mL) at pH 8.5 at 60°C. **x** = *N*-acetylglycinamide, ▼ = glycine. Bottom spectrum = ^{1}H NMR spectrum acquired before the reaction was heated 60°C. Top spectrum = ^{1}H NMR spectrum acquired after heating reaction at 60°C for 36 h.

N-Acetylglycyl-DL-alanylglycine, $\mathbf{5_{AG}}$ (2 diastereoisomers) (▼): ^{1}H NMR (600 MHz, H_2O) δ 4.42 (q, *J* = 7.2 Hz, 1H, Ala-(C2)–H), 3.98 (s, 2H, Ac-Gly-(C***H***$_2$)–H), 3.84-3.76 (m, 2H, Gly-(C***H***$_2$)-COOH), 2.06 (s, 3H, COC***H***$_3$), 1.41 (d, *J* = 7.3 Hz, Ala-(C3)–H$_3$). **7** (✖) (partial assignment): δ_H 3.91 (2H, s, C*H*$_2$), 2.10 (3H, s, C*H*$_3$).

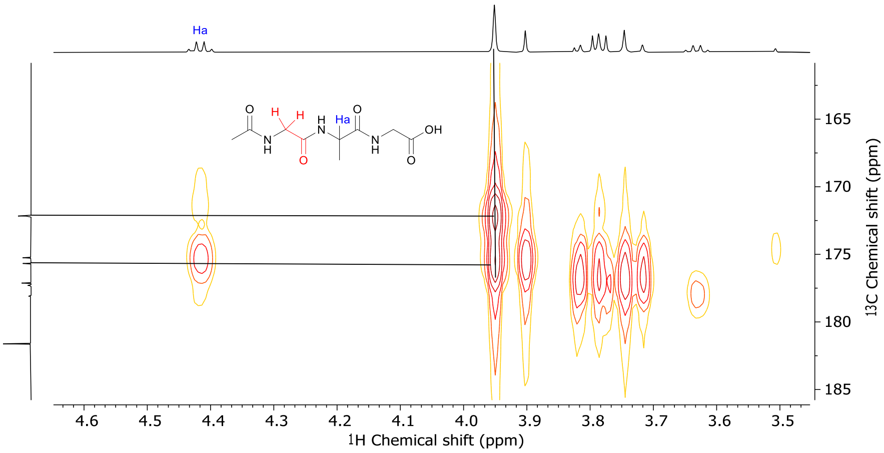

Figure S108: ^{1}H–^{13}C HMBC (^{1}H: 600 MHz [3.50–4.60 ppm], ^{13}C: 176 MHz [160-190 ppm]) spectrum showing the diagnostic $^{2}J_{CH}$ coupling of the Ac-Gly-Ala-α*H*-Gly-OH is at 4.42 ppm with two resonances at 175.5 and 172.3 ppm, which is characteristic of amide bond formation of Ala.

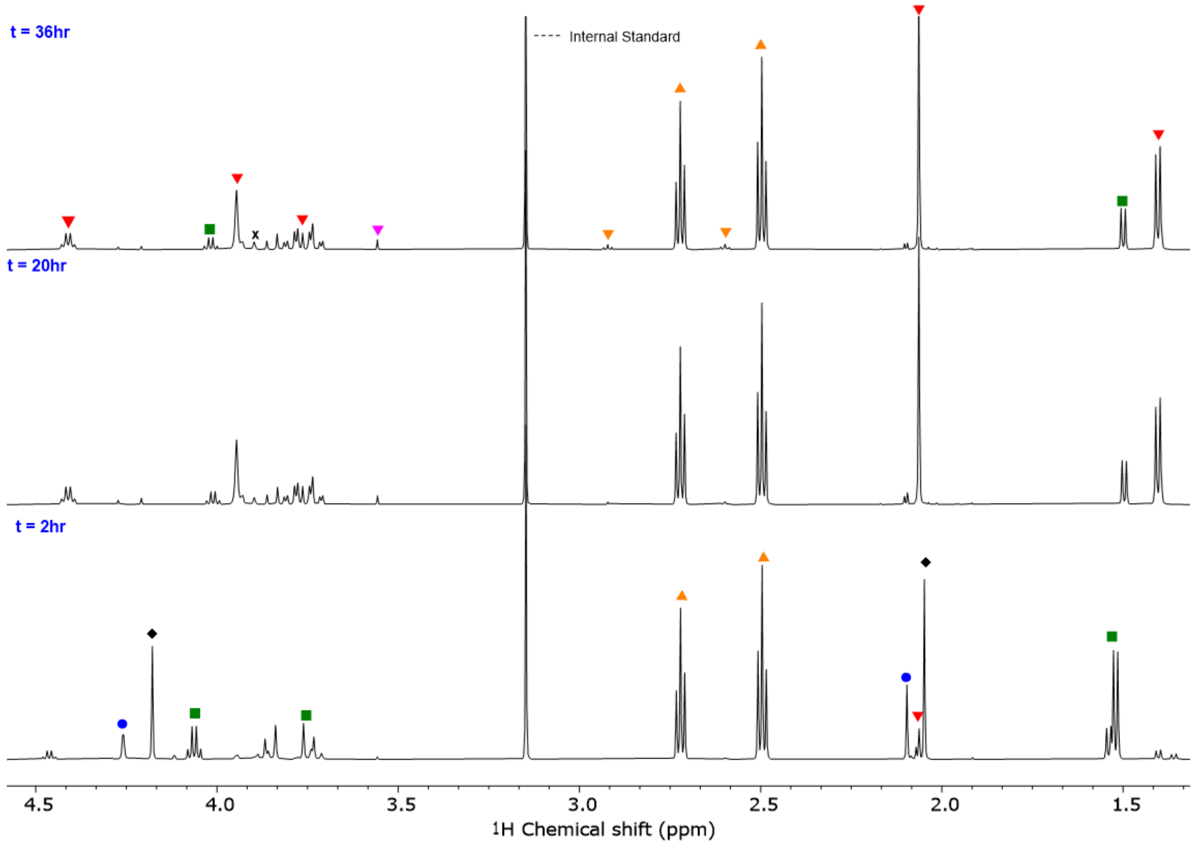

Figure S109: ^{1}H NMR (600 MHz, H_2O/D_2O 9:1, noesygppr1d, 1.3-4.5 ppm) spectrum to show the reaction of *N*-acetylglycine nitrile (**1**; 100 mM), *L*-alanyl-glycine (**2**$_{AG}$; 130 mM), 3-mercaptopropionic acid (**6b**; 60 mM) with MSM (50 mM) as an internal standard in H_2O (1 mL) at pH 7 at 60°C. **x** = *N*-acetylglycinamide, ▼ = glycine. Bottom spectrum = ^{1}H NMR spectrum acquired before the reaction was heated 60 °C. Top spectrum = ^{1}H NMR spectrum acquired after heating the reaction at 60°C for 36 h.

*Preparative synthesis of amidines **3'***

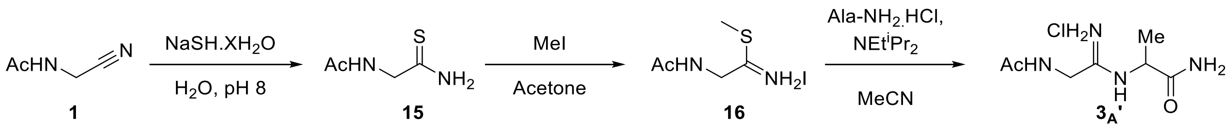

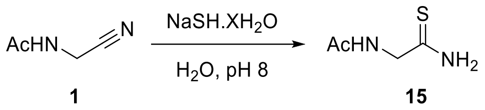

N-Acetyl glycine nitrile **1** (0.6 g, 6.3 mmol) and $B(OH)_3$ (1.5 g, 25 mmol) were dissolved in H_2O (25 mL). $NaSH.xH_2O$ (assumed 50 wt % NaSH, 3.2 g, 28 mmol) added and solution was adjusted to pH 9 with 4 M HCl. The solution as allowed to stir for 18 h, then H_2S was outgassed from the solution into a solution of concentrated bleach - a stream of N_2 was passed through the solution for 30 mins while the pH of the reaction mixture was periodically adjusted to ~pH 4 by addition of 4 M HCl. The resultant suspension was concentrated under reduced pressure and triturated with EtOAc (3 × 50 mL). The supernatant was concentrated to give **15** (440 mg, 3.3 mmol, 53%) as a yellow solid. Spectral data were consistent with those previously reported.[3]

^{1}H NMR (700 MHz, D_2O, zg30): 4.21 (2H, s, AcNHC***H₂-***), 2.07 (3H, s, ***Ac***), ppm

^{13}C NMR (176.1 MHz, D_2O , zgpg30): 205.0 (***C***(=S)NH_2), 175.5 (***C***ONH), 49.9 (AcNH***C***H_2-), 22.4 (***C***H_3CONH-) ppm.

ES-HRMS (pos.) theoretical for $[C_4H_8N_2OS+H]^+$: 133.0430; observed 133.0430.

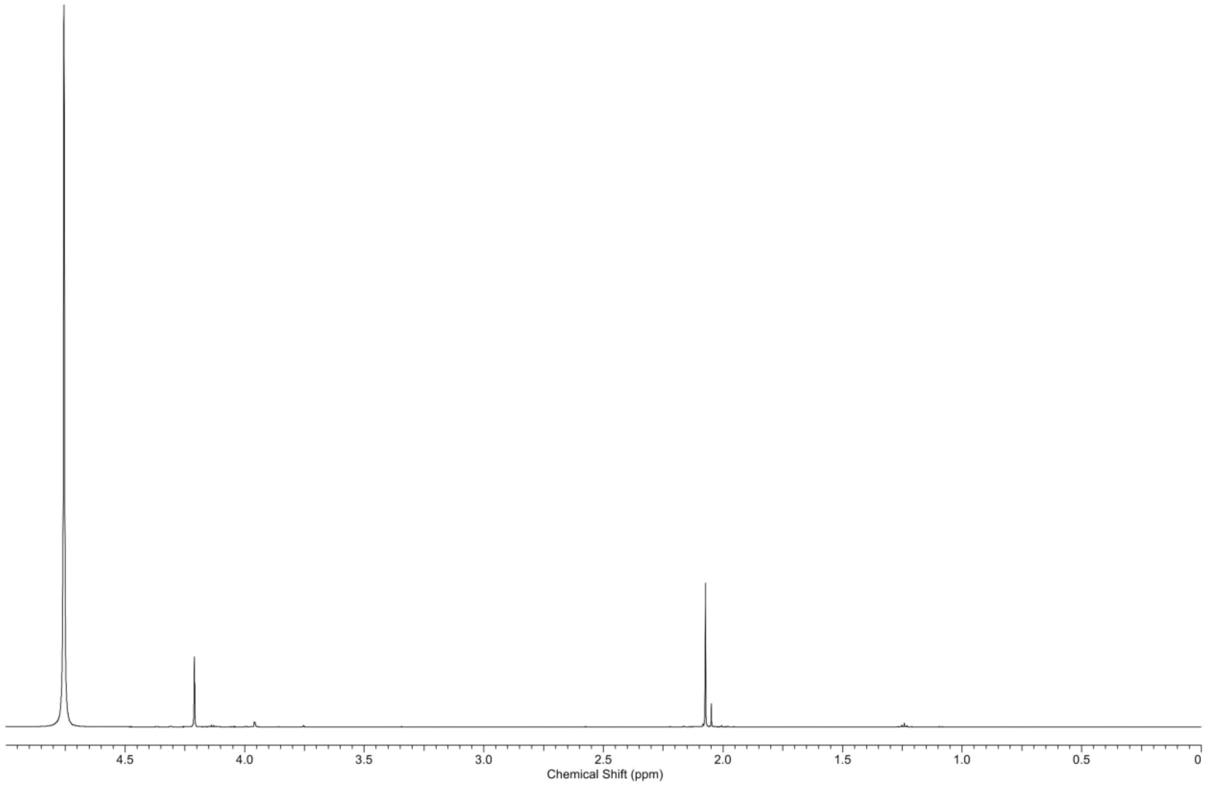

Figure S110 ^{1}H NMR (700 MHz, D_2O, zg30, 0.0-5.0 ppm) spectrum of **15**

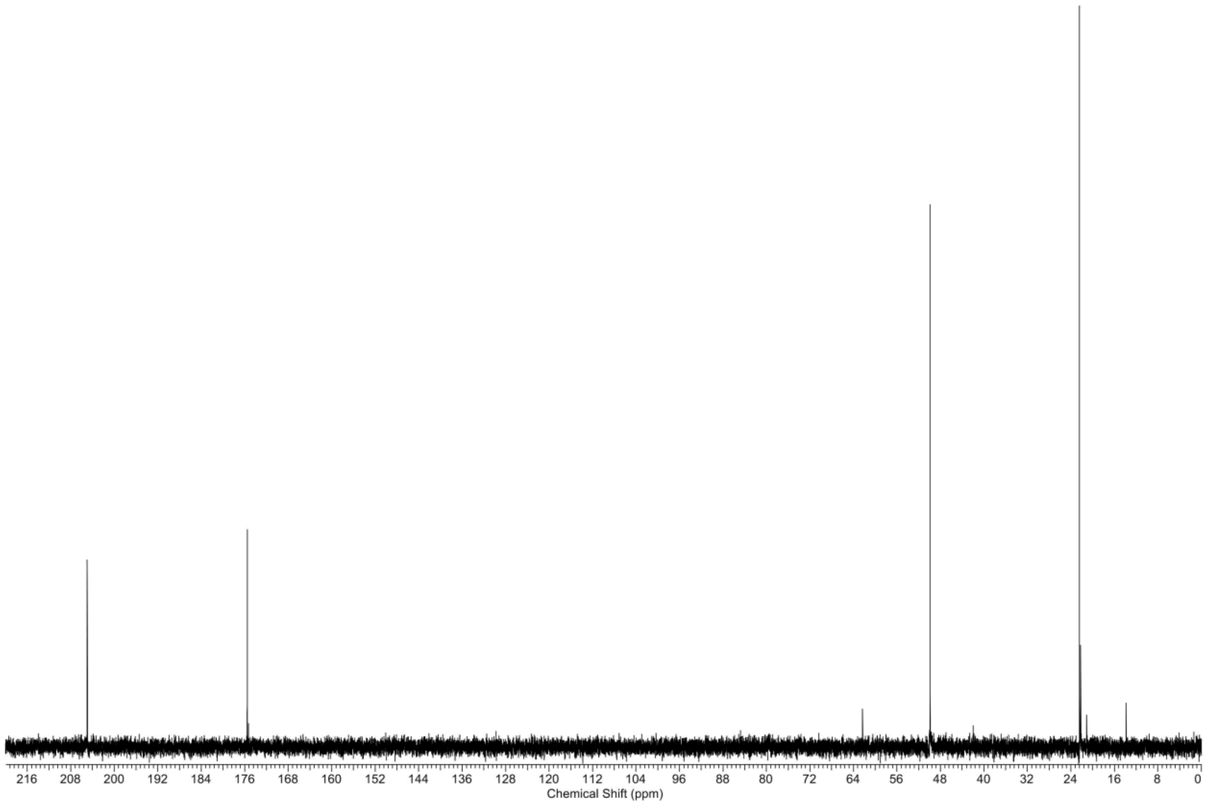

Figure S111: ^{13}C NMR (176.1 MHz, D_2O, zg30, 0-220 ppm) spectrum of **15**

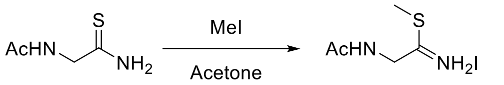

15 **16**

15 (200 mg, 1.5 mmol) was dissolved in acetone (3.0 mL, 0.5 M) and the solution was cooled to 0°C. Iodomethane (200 µL, 3.1 mmol, 2 equiv.) was added and the solution allowed to warm to room temperature and stand for 3 h. The colourless precipitate which formed in this time was separated by centrifugation, washed with acetone (3 × 3 mL) and dried under vacuum to give thioimidate **16** as a colourless solid (250 mg, 0.9 mmol, 61%).

^{1}H NMR (700 MHz, d_3-MeOD, zg30): 4.24 (2H, s, AcNHC***H₂***-), 2.05 (3H, s, ***Ac***), 2.01 (3H, s, S***Me***) ppm

^{13}C NMR (176.1 MHz, d_3-MeOD, zgpg30): 180.4 (***C***(=NH)SMe), 174.5 (***C***ONH), 41.8 (AcNH***C***H_2-), 22.3 (***C***H_3CONH-), 22.1 (S***C***H_3) ppm

ES-HRMS (pos.) theoretical for $[C_5H_{11}N_2OS]^+$: 147.0598, observed 147.0594

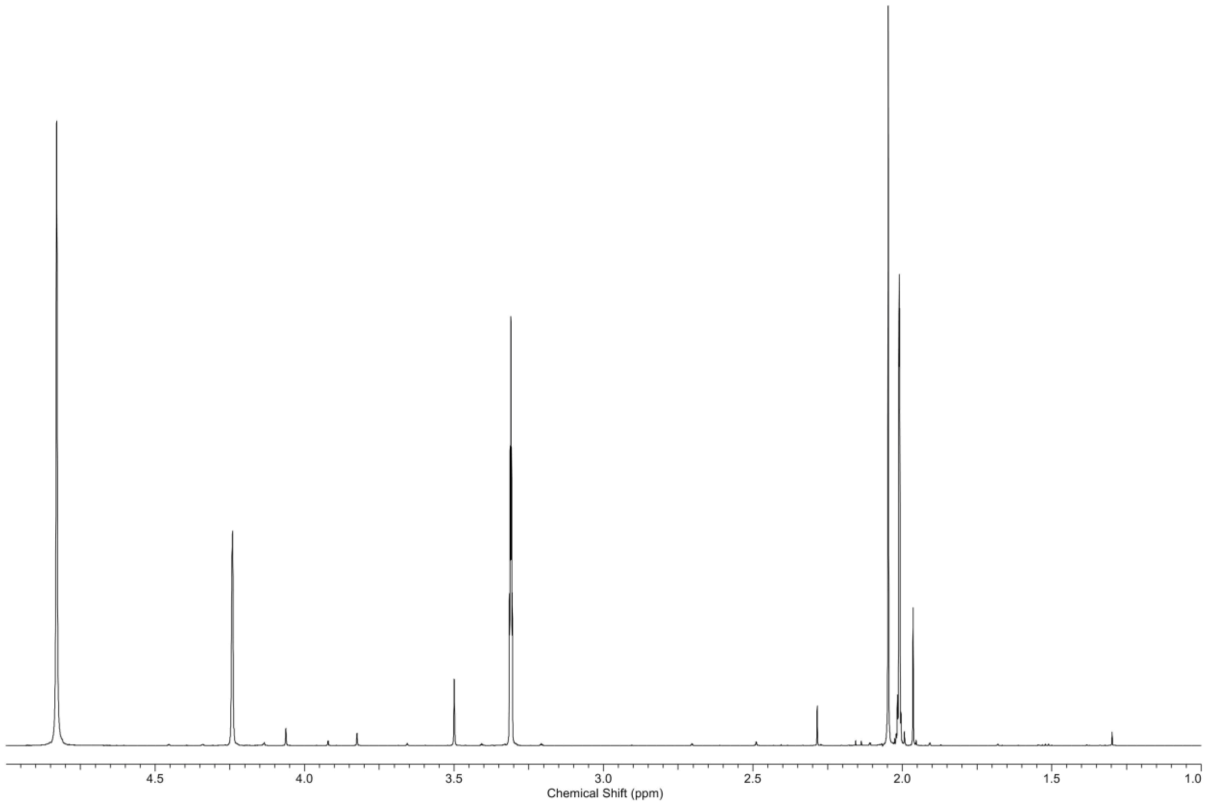

Figure S112: ^{1}H NMR (700 MHz, d_3-MeOD, zg30, 0.0-5.0 ppm) spectrum of **16**

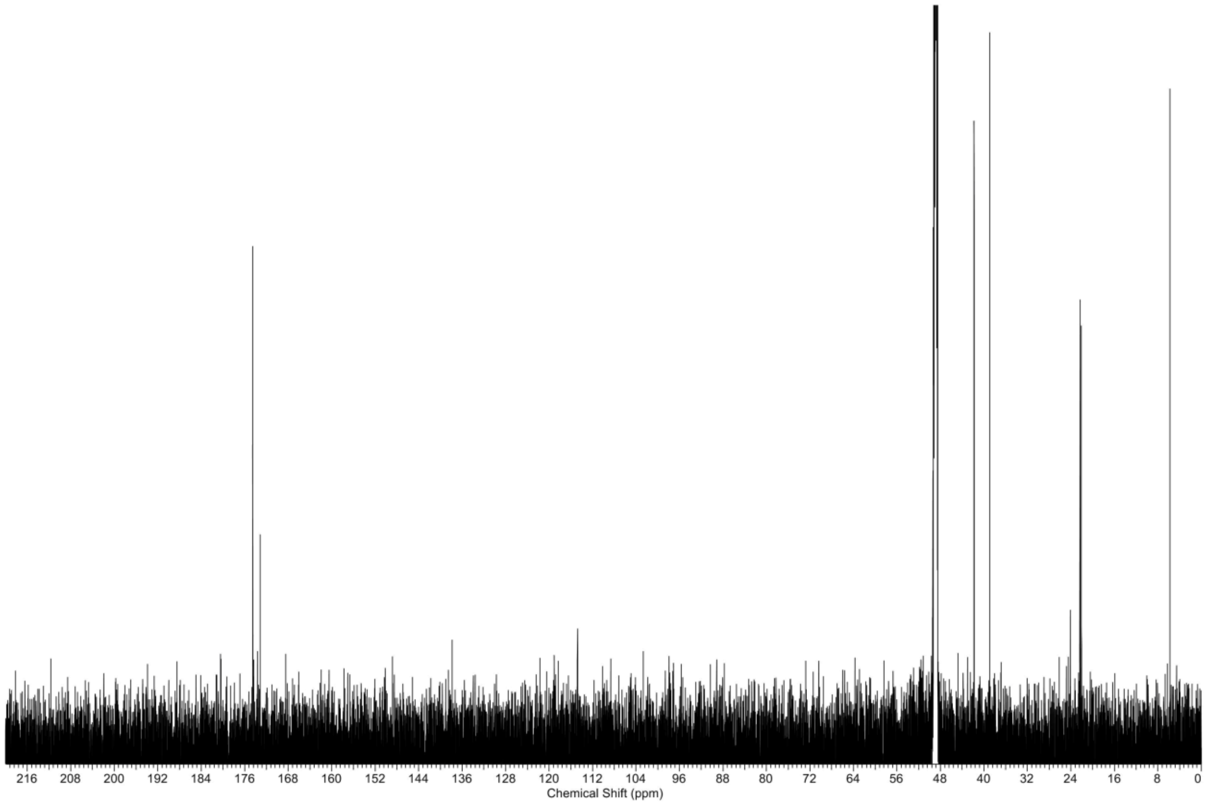

Figure S113: ^{13}C NMR (176.1 MHz, d_3-MeOD, zg30, 0-220 ppm) spectrum of **16**

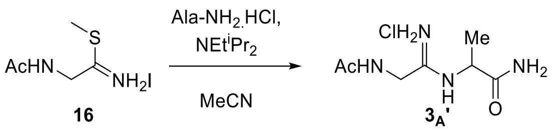

AlaNH$_2$.HCl **2$_A$'**.HCl (85 mg, 0.7 mmol) and thioimidate **16** (215 mg, 0.9 mmol, 1.3 equiv.) were suspended in anhydrous MeCN (3.6 mL) in a flame-dried flask under an atmosphere of argon. NEt^{i}Pr$_2$ (240 µL, 1.4 mmol, 2 equiv.) was added, and the homogeneous solution stirred for 2 h at which point a colourless precipitate had formed. The solid was separated by centrifugation, washed with anhydrous MeCN (3 × 2 mL) and dried under vacuum to give amidine **3$_A$'** as a colourless solid (120 mg, 0.5 mmol, 71%).

^{1}H NMR (700 MHz, D$_2$O, zg30): 4.38 (1H, q, *J* = 7.0 Hz, C***H***(Me)CONH$_2$), 4.21 (2H, AB, *J* = 17.5 Hz, AcNHC***H$_2$***-), 2.06 (3H, s, ***Ac***), 1.50 (3H, d, *J* = 7.0 Hz, ***Me***) ppm

^{13}C NMR (176.1 MHz, D$_2$O, zgpg30): 176.3 (CH$_3$*C*ONH), 174.7 (***C***ONH$_2$), 165.4 (***C***(NH)NH), 52.2 (AcNH***C***H$_2$-), 40.9 (***C***H(Me)CONH$_2$), 22.2 (***C***H$_3$CONH), 17.5 (***Me***) ppm

ES-HRMS (pos.) theoretical for [C$_7$H$_{15}$N$_4$O$_2$]$^+$: 187.1190, measured: 187.1191.

IR: 3167, 2924, 1653, 1552, 1287, 1067, 557, 465 cm^{-1}

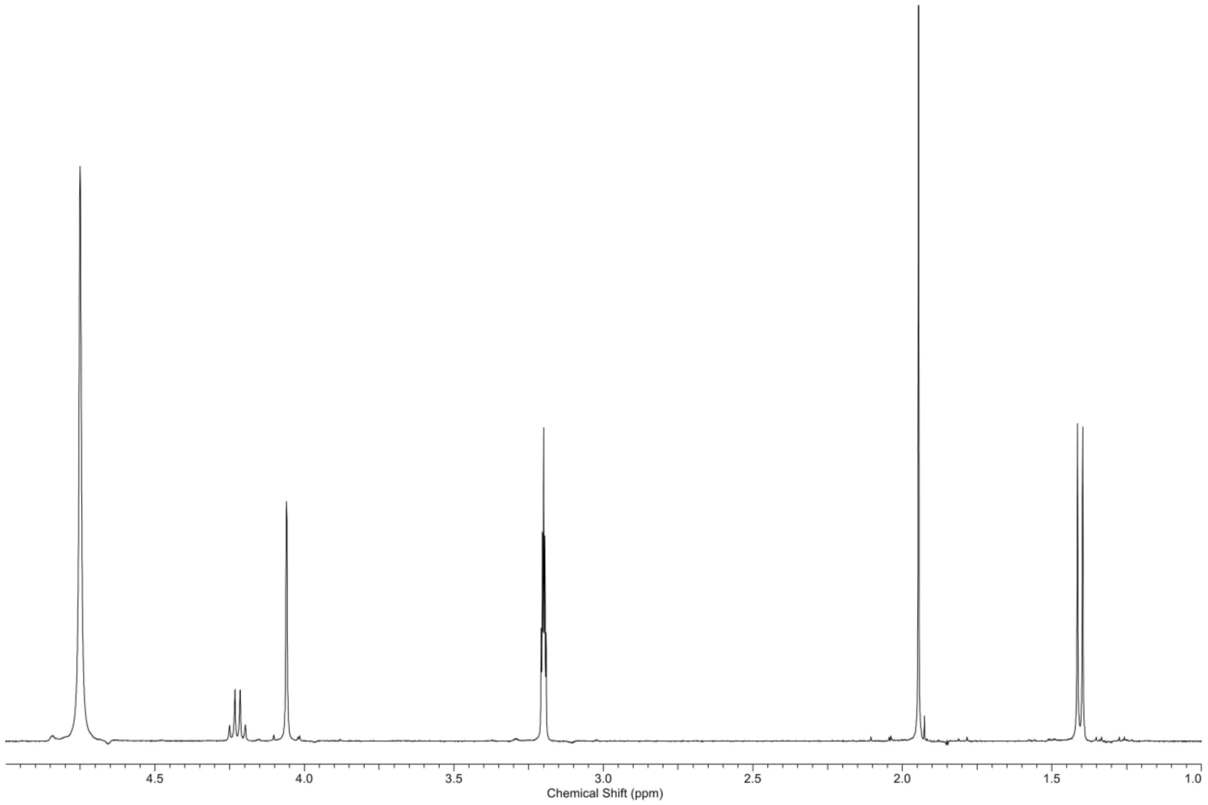

Figure S114: ^{1}H NMR (700 MHz, D$_2$O, zg30, 0.0-5.0 ppm) spectrum of **3$_A$'**

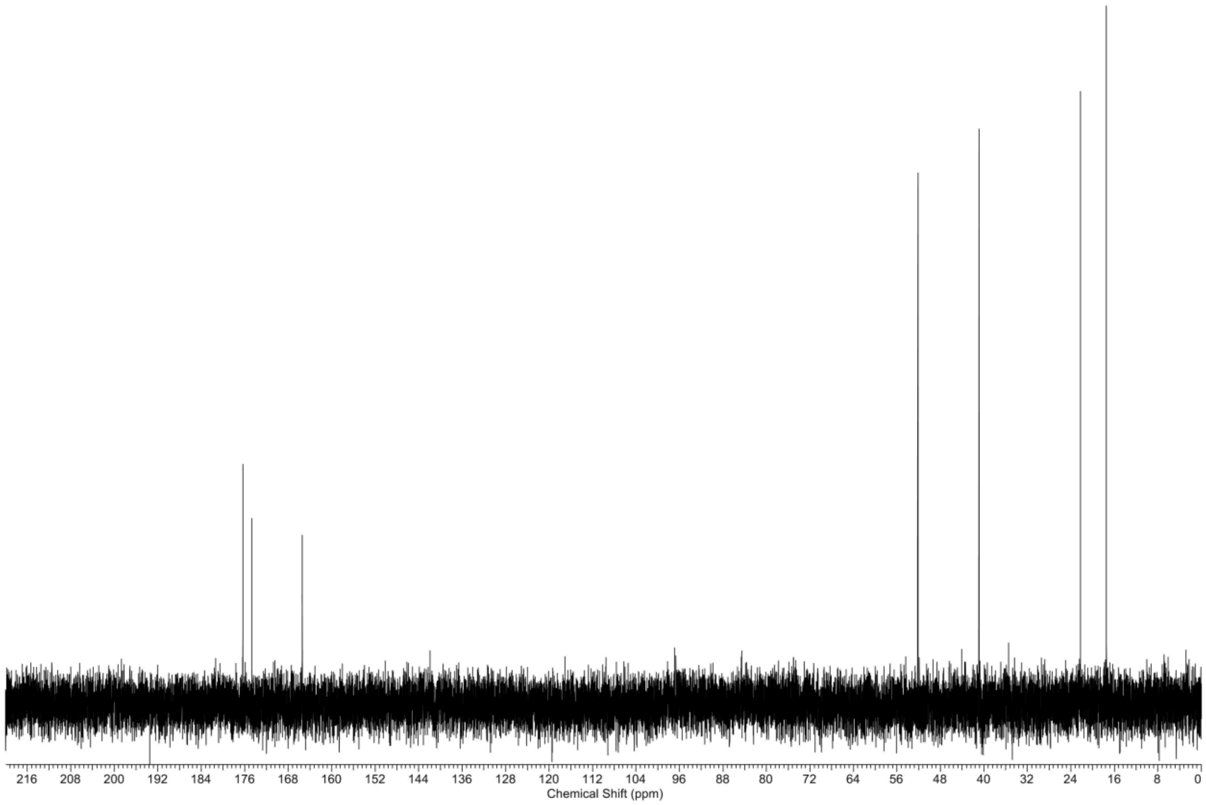

Figure S115: ^{13}C NMR (176.1 MHz, D_2O, zg30, 0-220 ppm) spectrum of **3$_A$'**

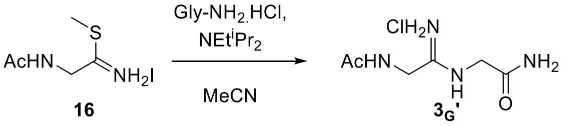

GlyNH$_2$.HCl **2$_G$'**.HCl (36.5 mg, 0.3 mmol) and thioimidate **16** (103 mg, 0.4 mmol, 1.3 equiv.) were suspended in anhydrous MeCN (2.2 mL) in a flame-dried flask under an atmosphere of argon. NEt^{i}Pr$_2$ (115 μL, 0.7 mmol, 2 equiv) was added, and the homogeneous solution stirred for 3 h at which point a colourless precipitate had formed. The solid was separated by centrifugation, washed with anhydrous MeCN (3 × 2 mL) and dried under vacuum to give amidine **3$_G$'** as a colourless solid (55 mg, 0.2 mmol, 75%).

ES-HRMS (pos.) theoretical for $[C_6H_{13}N_4O_2]^+$: 173.1033, observed 173.1032

^{1}H NMR (700 MHz, D_2O, zg30): 4.28 (2H, s, AcNHC***H$_2$***-), 4.18 (2H, s, C***H$_2$***CONH$_2$) 2.10 (3H, s, ***Ac***) ppm

^{13}C NMR (176.1 MHz, D_2O, zgpg30): 176.4 (CH$_3$*C*ONH), 171.1 (***C***ONH$_2$), 166.6 (***C***(NH)NH), 44.6 (AcNH***C***H$_2$-), 40.7 (***C***H$_2$CONH$_2$), 22.3 (***C***H$_3$CONH) ppm

IR: 3230, 3026, 1677, 1647, 1537, 1393, 748, 592, 506 cm^{-1}

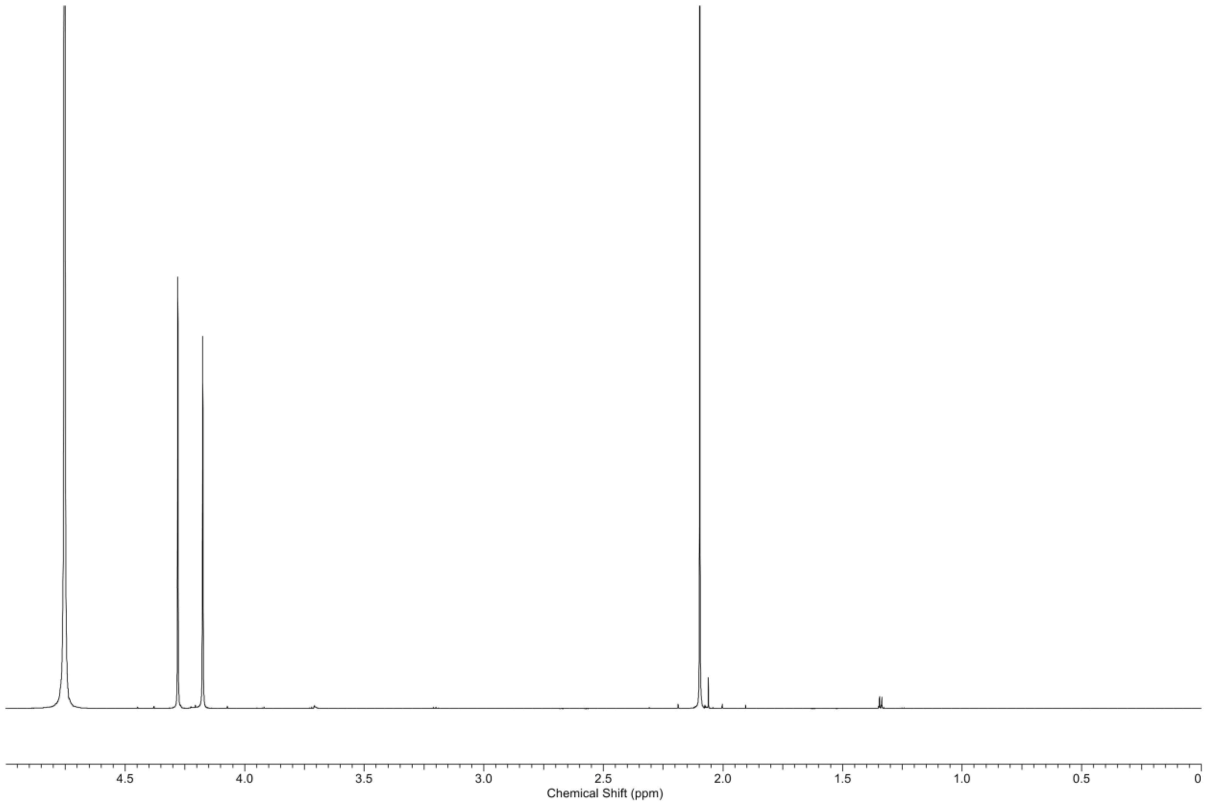

Figure S116: ^{1}H NMR (700 MHz, D_2O, zg30, 0.0-5.0 ppm) spectrum of **3G'**

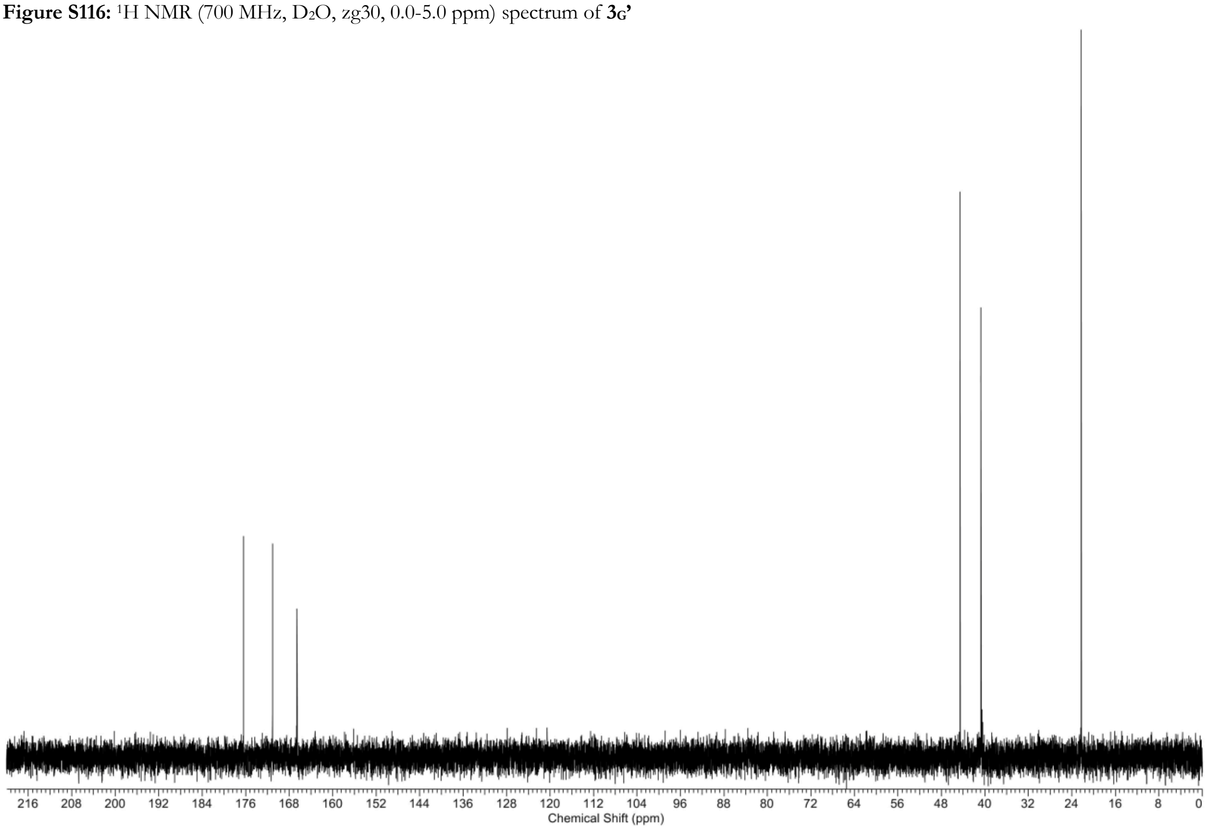

Figure S117: ^{13}C NMR (176.1 MHz, D_2O, zg30, 0-220 ppm)spectrum of **3G'**

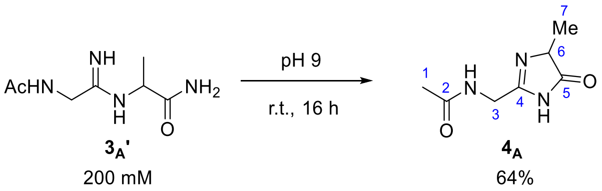

3_A' (5 mg, 0.025 mmol) and MSM [internal standard] were dissolved in H_2O (800 µL). The solution was adjusted to pH 9 with 4 M NaOH, D_2O (50 µL) was added and the solution volume was increased to 1 mL. ^1H NMR spectra were acquired periodically. After 16 h the mixture was characterised by ^1H, ^{13}C, and ^1H-^{13}C HMBC NMR. The solution was lyophilised and analysed by HRMS.

^1H NMR (700 MHz, H_2O/D_2O 19:1, noesygppr1d): 4.26 (2H, t, *J* = 2.2 Hz, C3-H_2-), 4.16 (1H, qt, *J* = 7.4, 2.2 Hz, C6-H), 2.05 (3H, s, C1-H_3), 1.33 (3H, d, *J* = 7.4 Hz, C7-H_3) ppm.

^{13}C NMR (176.1 MHz, H_2O/D_2O 19:1, zgpg30): 193.7 (C5), 175.6 (C2), 173.9 (C4), 61.4 (C6), 39.1 (C3), 22.4 (C1), 15.65 (C7) ppm.

HRMS (ESI pos.)– $[C_7H_{11}N_3O_2+H]^+$ theoretical 170.0924, measured 170.0921.

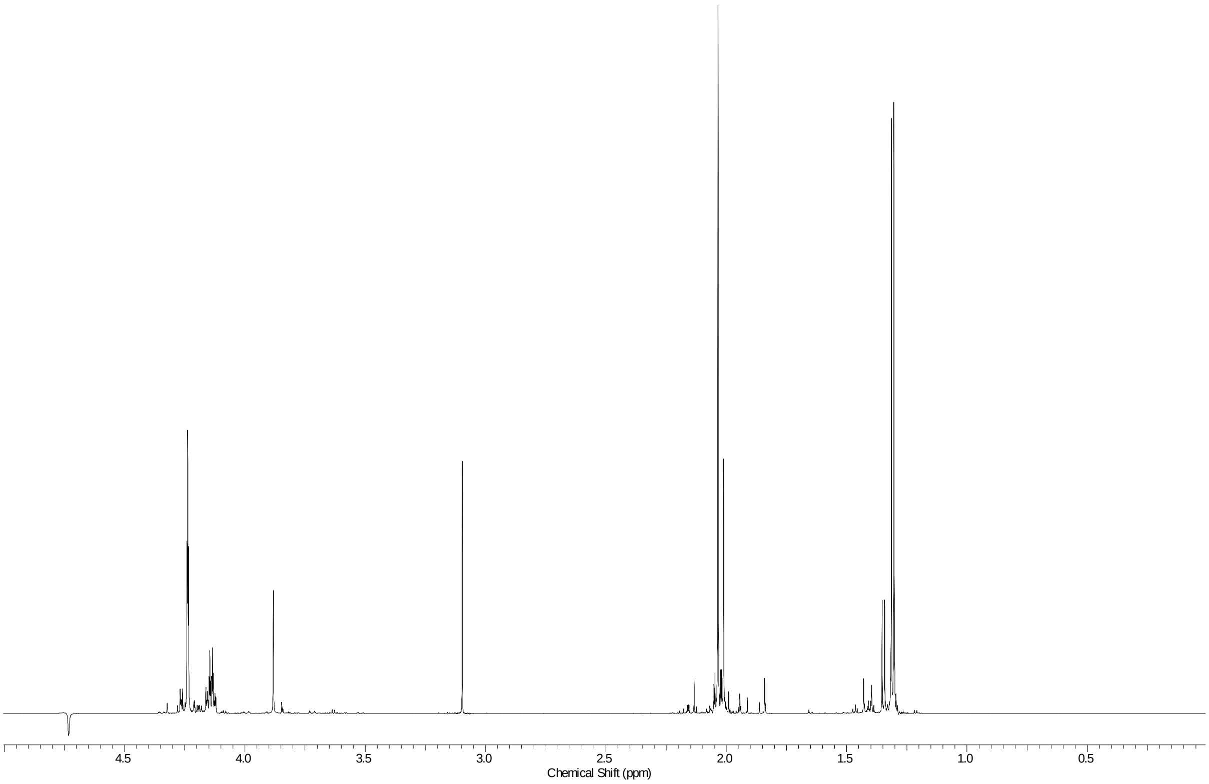

Figure S118: ^1H NMR (700 MHz, H_2O/D_2O 9:1, noesygppr1d, 0.0-5.0 ppm) spectrum to show the cyclisation of **3_A'** (25 mM) to form **4_A** after 16 h at room temperature and pH 9.

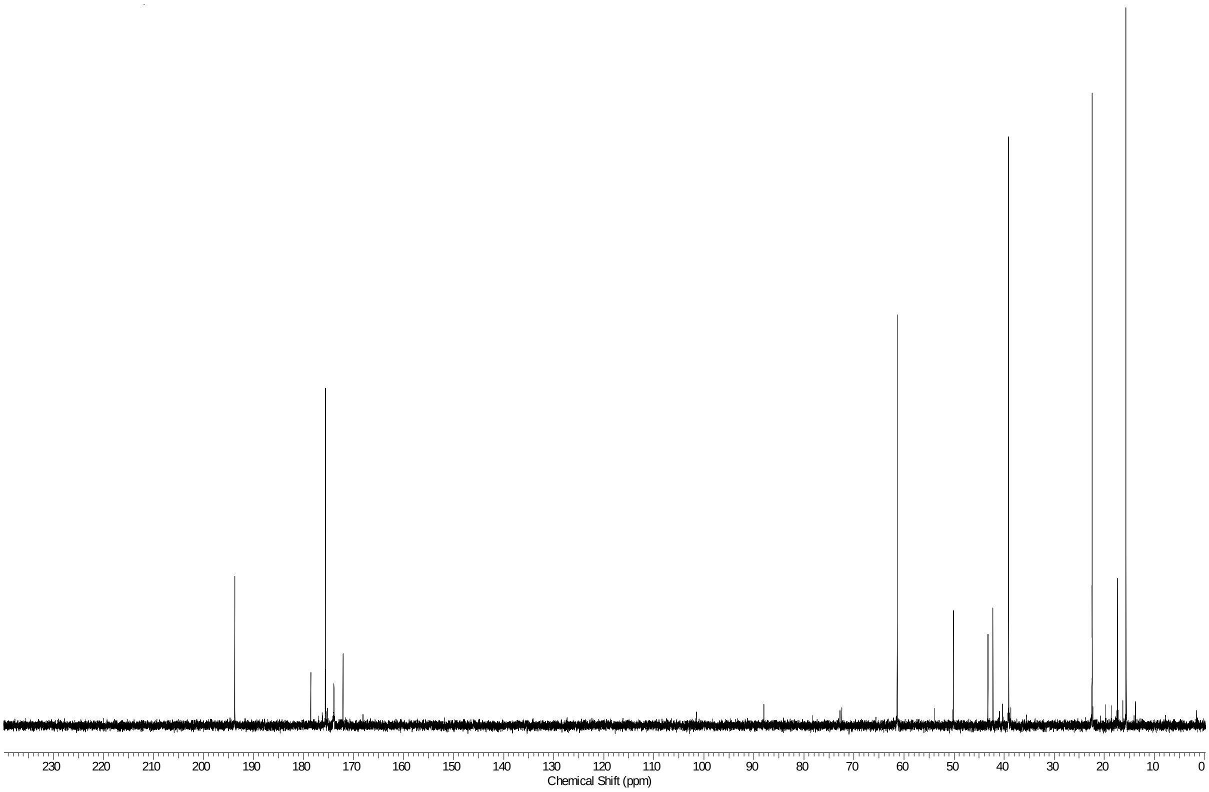

Figure S119 ^{13}C NMR (176.1 MHz, H_2O/D_2O 9:1, zg30, 0-240 ppm)) spectrum to show the cyclisation of **3A'** (25 mM) to form **4A** after 16 h at room temperature and pH 9.

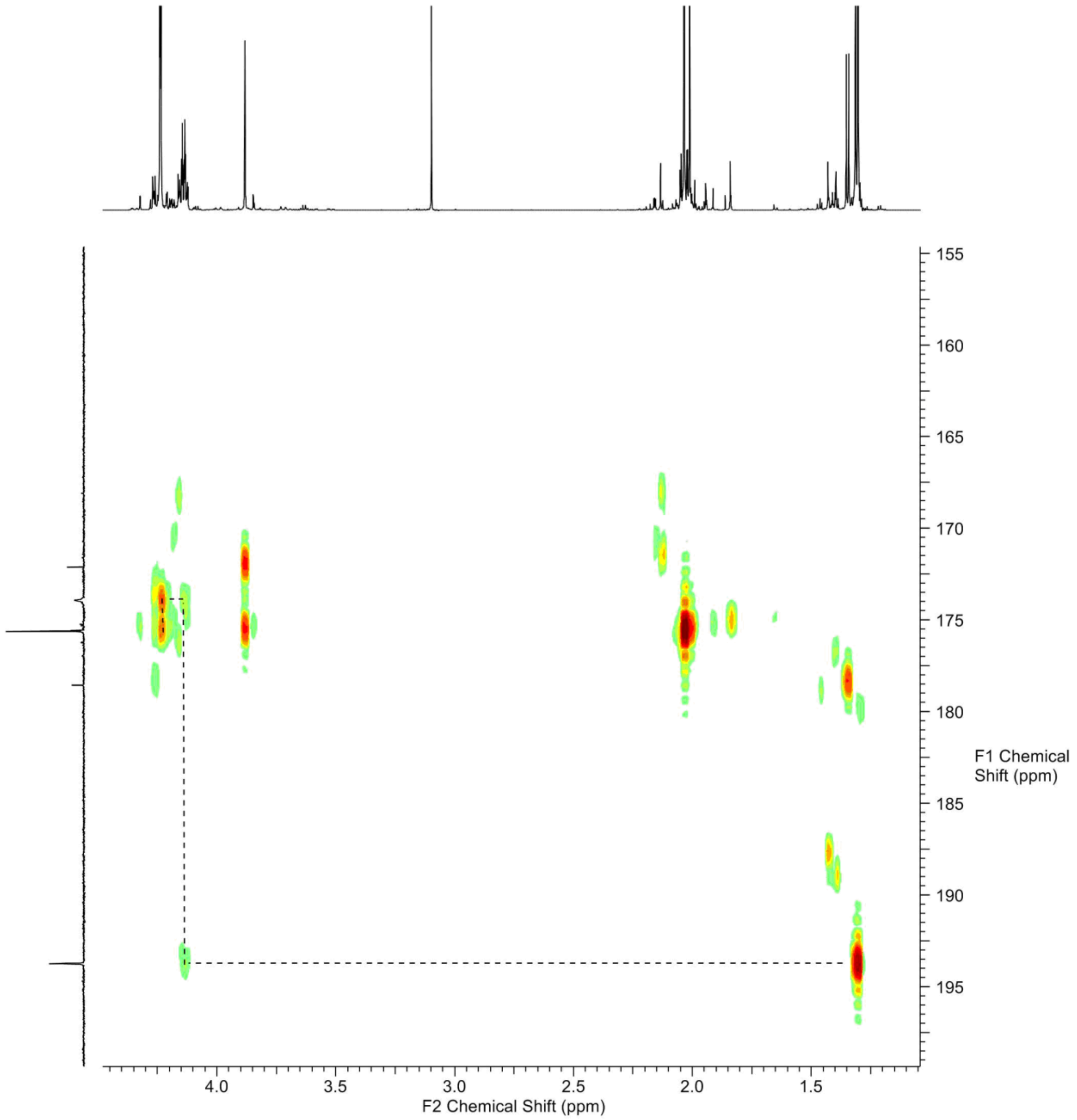

Figure S120: ^{1}H–^{13}C HMBC (^{1}H: 700 MHz [1.0-4.5 ppm], ^{13}C: 176 MHz [155-200 ppm], H_2O/D_2O 9:1) spectrum to show the cyclisation of **3A'** (25 mM) to form **4A** after 16 h at room temperature and pH 9, showing the diagnostic $^{2}J_{CH}$ and $^{3}J_{CH}$ coupling of **Ala**-αH in **4A** at 4.15 ppm with two resonances at 174.5 and 188.7 ppm, which is characteristic of imidazolone formation.

*Isolation of **3**$_A$*

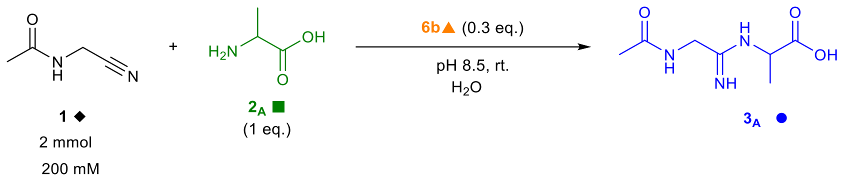

N-Acetyl glycine nitrile **1** (200 mg, 2 mmol), L-alanine **2**$_A$ (2 mmol) and 3-mercaptopriopionic acid **6b** were dissolved in H_2O (10 mL) and the solution was adjusted to pH 8.5 with 4 M NaOH. The reaction was stirred at room temperature for 2 days, then the solution was adjusted to pH 3.0 with 4 M HCl and extracted with Et_2O (3 × 25 mL) to remove the thiol. The solution was adjusted to pH 7.0 and lyophilised. The solid residue was triturated with EtOH (2 × 15 mL) to leave **3**$_A$ as a colourless solid (250 mg, 1.33 mmol, 67%).

^1H NMR (700 MHz, D_2O, zg30): 4.24 (2H, AB, *J* = 17.4 Hz, AcNHC***H*₂**-), 4.13 (1H, q, *J* = 7.1 Hz, C***H***(Me)CONH$_2$) 2.10 (3H, s, ***Ac***), 1.46 (3H, d, *J* = 7.1 Hz, ***Me***) ppm

^{13}C NMR (176.1 MHz, D_2O, zgpg30): 177.0 (***C***ONH$_2$), 176.2 (CH$_3$***C***ONH), 164.0 (***C***(NH)NH), 53.8 (***C***H(Me)CONH$_2$), 40.7 (AcNH***C***H$_2$-), 22.3 (***C***H$_3$CONH), 17.4 (**Me**) ppm

IR: 3238, 2932, 1641, 1563, 1362, 771, 731, 457cm^{-1}

ES-HRMS (pos.) theoretical for $[C_7H_{13}N_4O_2+H]^+$: 188.1030, observed 188.1030

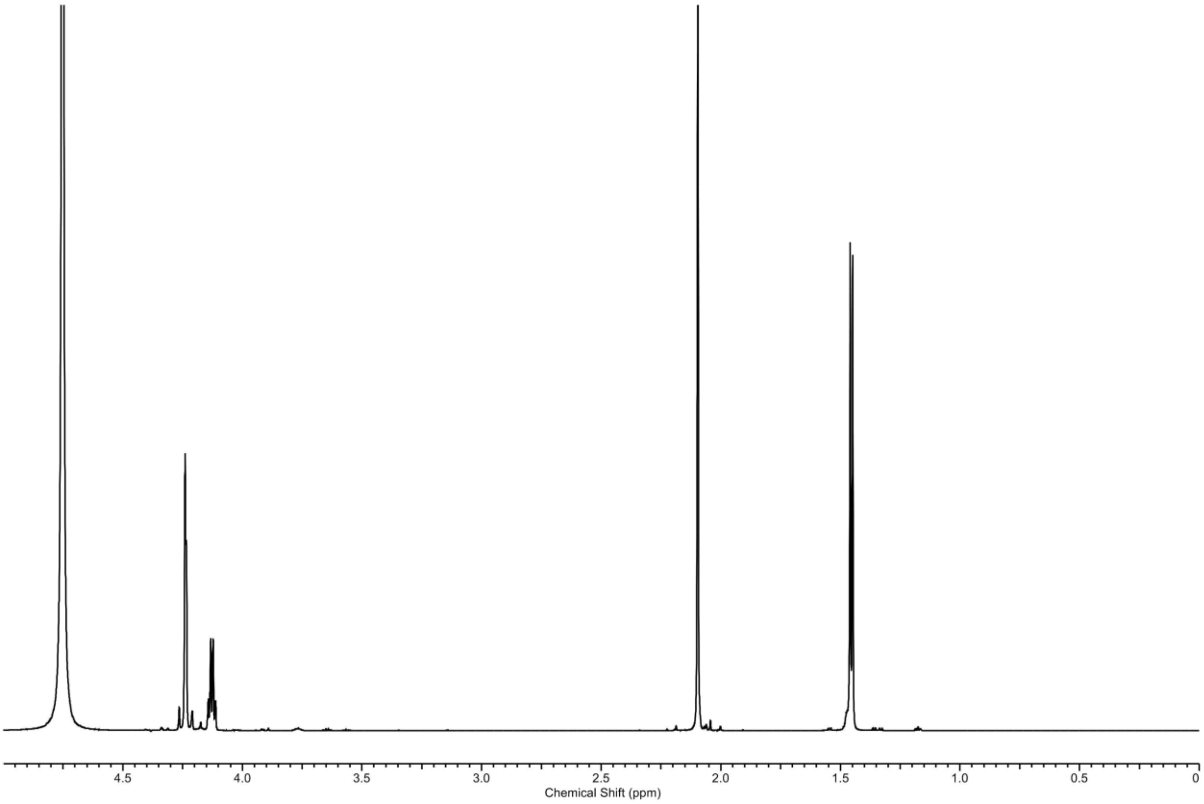

Figure S121: ^1H NMR (700 MHz, D_2O, zg30, 0.0-5.0 ppm) spectrum to show **3**$_A$.

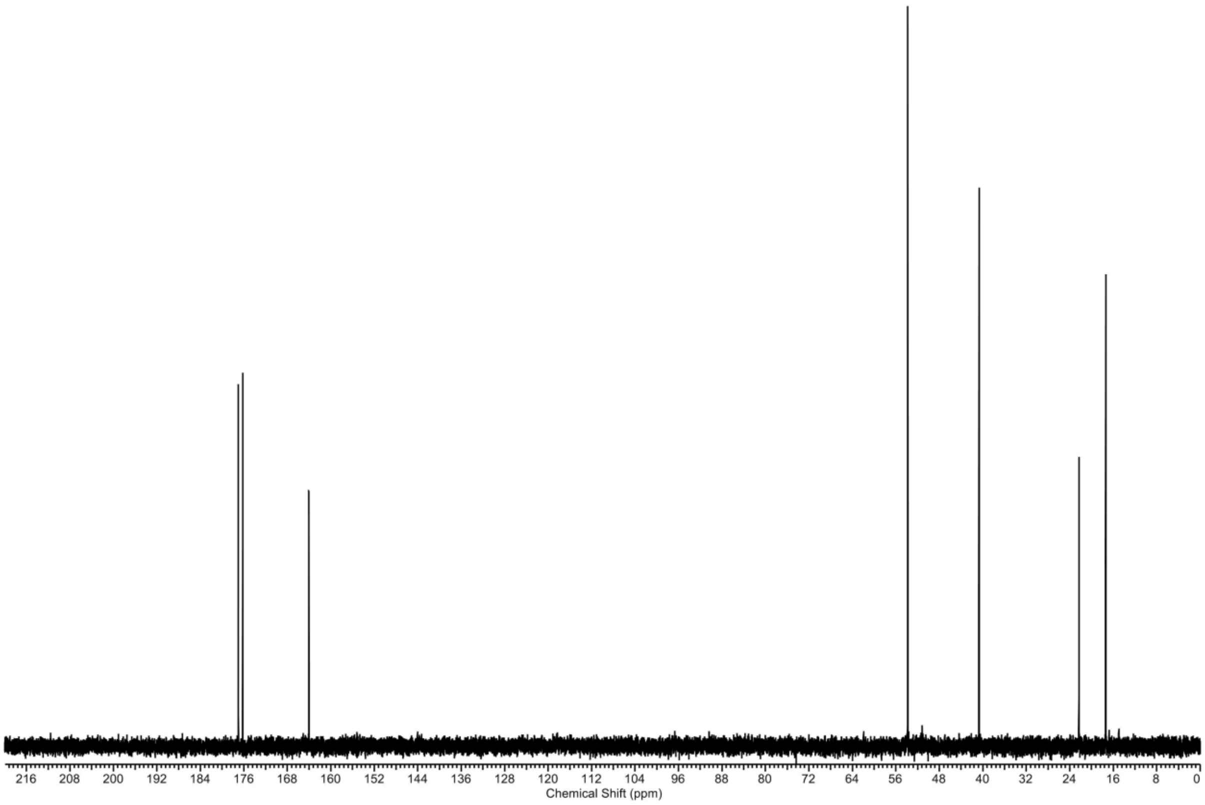

Figure S122: ^{13}C NMR (176.1 MHz, H_2O/D_2O 9:1, zg30, 0-220 ppm)) spectrum to show **3A**.

General procedure for the hydrolysis of authentic amidines

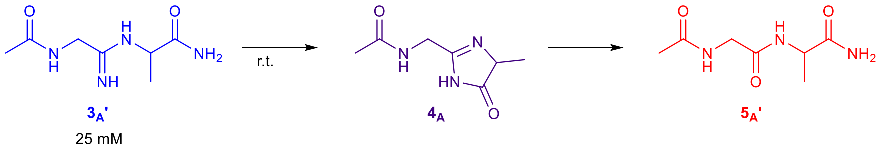

Amidine **3A'** (5 mg, 0.025 mmol) and MSM (30 mM) [internal standard] were dissolved in H_2O (800 µL). The solution was adjusted to the desired pH with 4 M NaOH, D_2O (50 µL) was added and volume was increased to 1 mL with H_2O. NMR spectra were acquired periodically.

*Hydrolysis of authentic amidines **3'***

*Hydrolysis of **3$_A$'** at pH 7 in phosphate buffer (100 mM)*

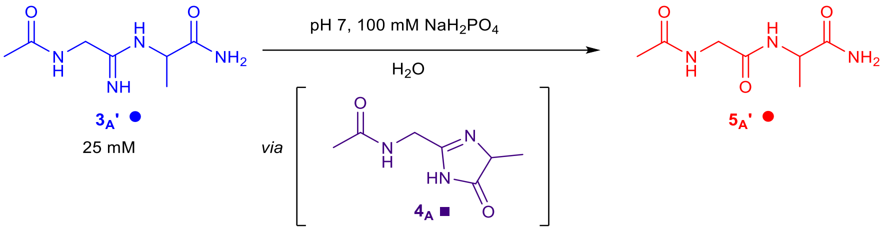

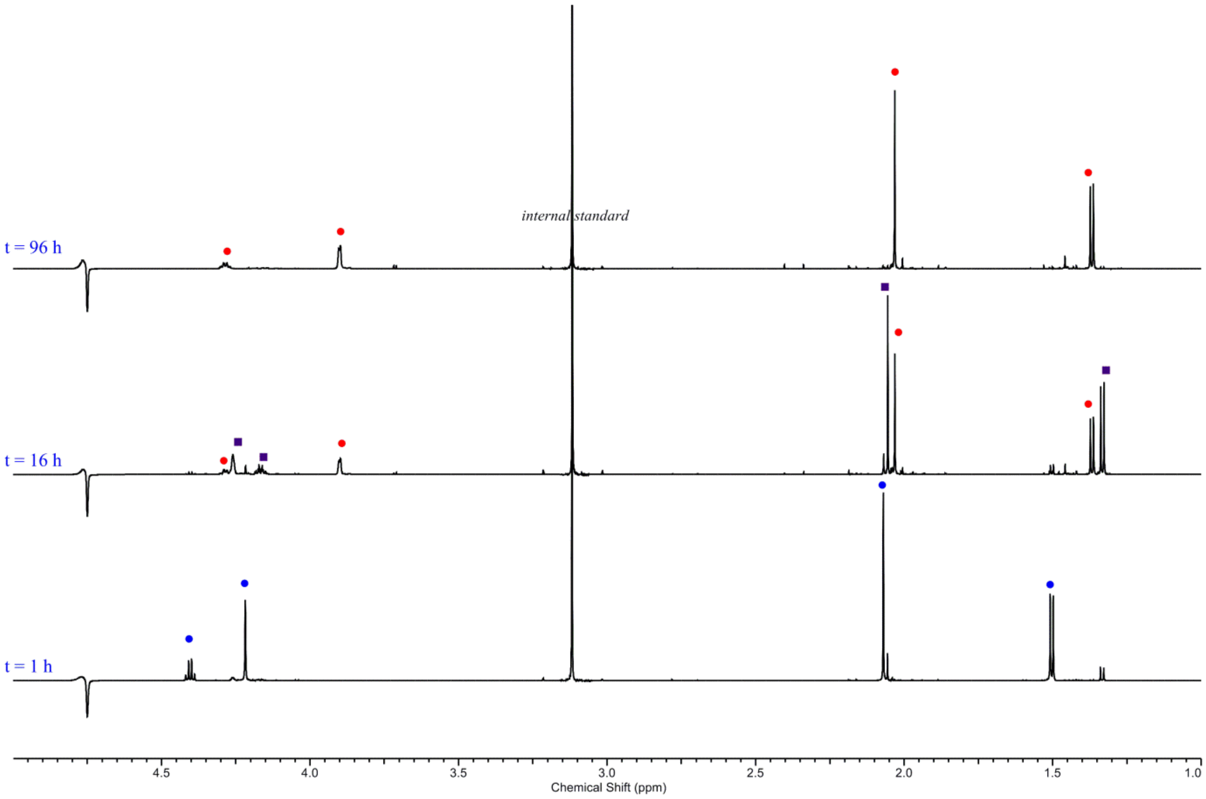

Figure S123. ^{1}H NMR (700 MHz, H_2O/D_2O 9:1, noesygppr1d, 1.0-5.0 ppm) spectra to show the hydrolysis of **3$_A$'** (25 mM) in phosphate buffer (100 mM) and MSM (30 mM) as an internal standard at pH 7.0 and room temperature.

time/ h	**3$_A$'** / %	**4$_A$'** / %	**5$_A$'** / %
1	88	11	0
8	27	54	14
16	7	50	33
24	3	36	47
36	2	21	60
48	2	12	67
72	2	4	76
96	2	2	77
144	2	0	76

Table S24. Hydrolysis of **3$_A$'** at pH 7 in phosphate buffer (100 mM).

*Hydrolysis of **3A'** at pH 7*

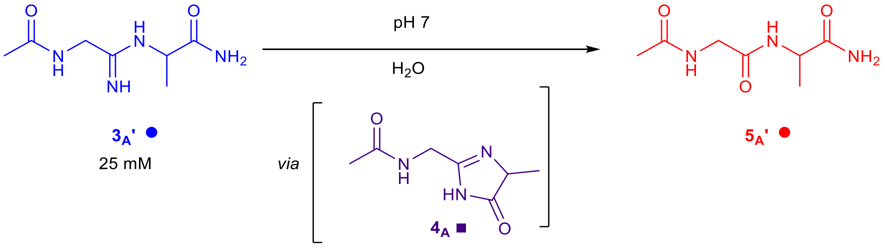

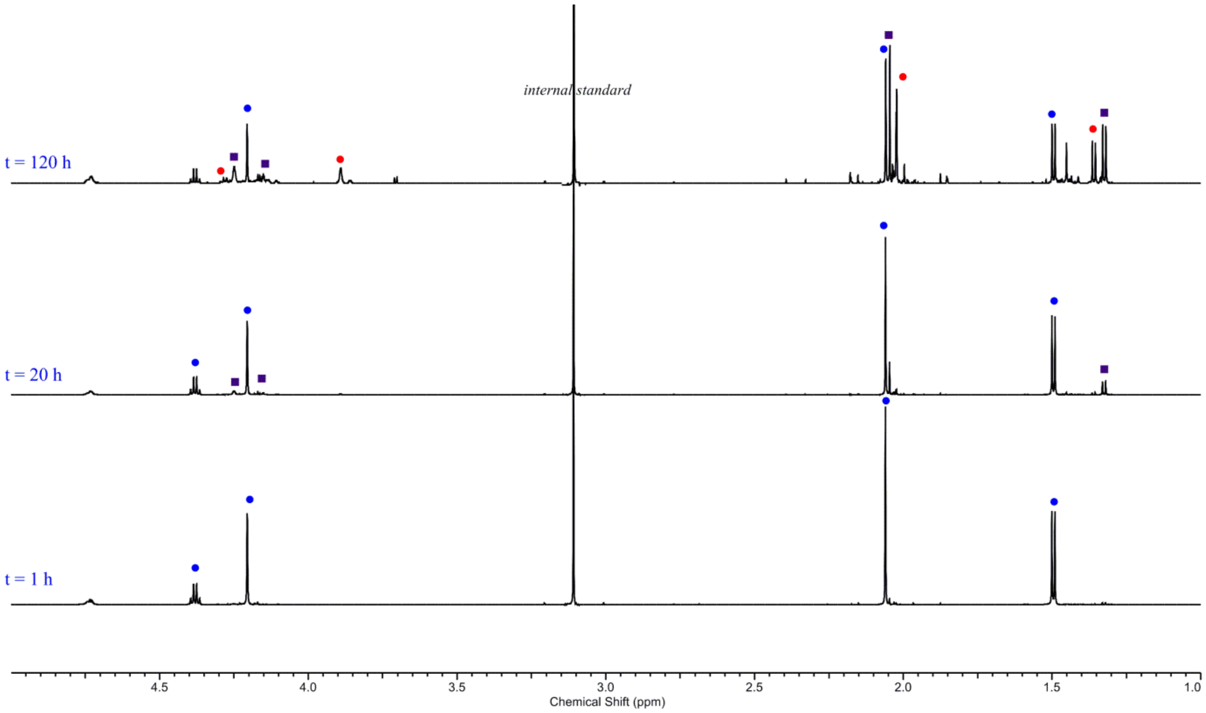

Figure S124. ^{1}H NMR (700 MHz, H_2O/D_2O 9:1, noesygppr1d, 1.0-5.0 ppm) spectra to show the hydrolysis of **3A'** (25 mM) with MSM (30 mM) as an internal standard at pH 7.0 and room temperature.

time/ h	**3A'** / %	**4A'** / %	**5A'** / %
1	99	3	0
3	97	4	0
12	92	10	1
20	84	14	2
46	67	23	6
60	53	24	9
120	32	24	19

Table S25. Hydrolysis of **3A'** at pH 7

Hydrolysis of ***3$_A$'*** *at pH 9*

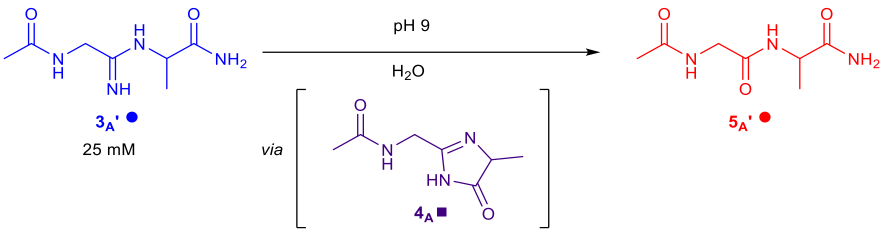

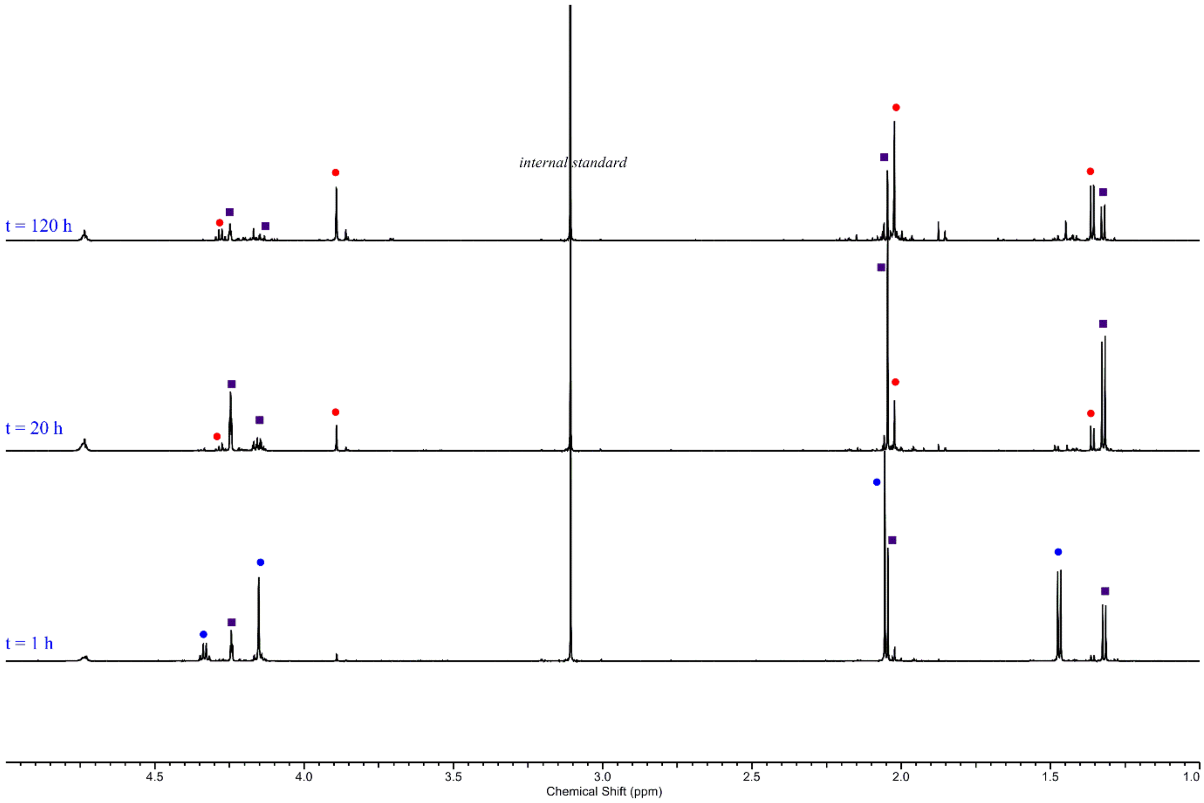

Figure S125. ^{1}H NMR (700 MHz, H_2O/D_2O 9:1, noesygppr1d, 1.0-5.0 ppm) spectra to show the hydrolysis of **3$_A$'** (25 mM) with MSM (30 mM) as an internal standard at pH 9.0 and room temperature.

time/ h	**3$_A$'** / %	**4$_A$'**/ %	**5$_A$'**/ %
1	81	37	4
3	46	59	8
12	18	78	14
20		75	18
46		54	28
60		45	33
120		22	42

Table S26. Hydrolysis of **3$_A$'** at pH 9.

Hydrolysis of $\mathit{3_A}$*' at pH 10*

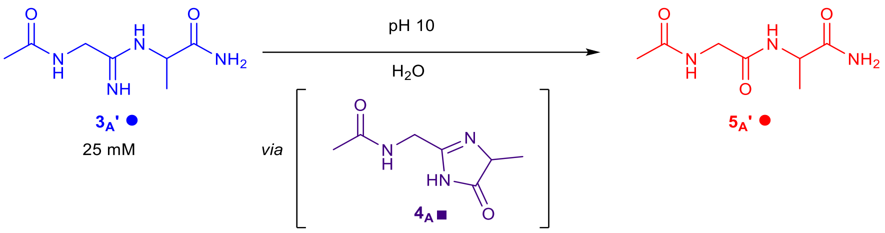

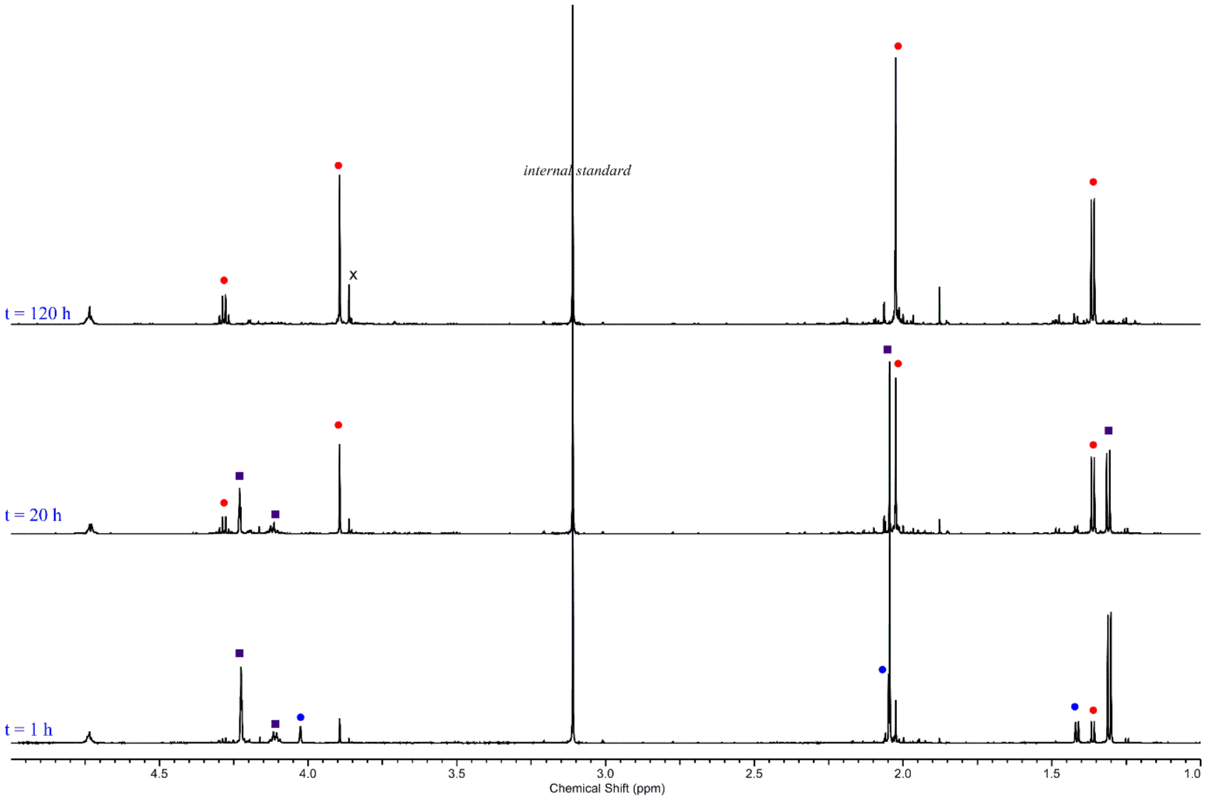

Figure S126. ^{1}H NMR (700 MHz, H_2O/D_2O 9:1, noesygppr1d, 1.0-5.0 ppm) spectra to show the hydrolysis of **3A'** (25 mM) with MSM (30 mM) as an internal standard at pH 10.0 and room temperature.

time/ h	**3A'** / %	**4A'**/ %	**5A'**/ %
1	15	63	9
3	2	62	15
12	0	43	27
20	0	33	34
46	0	12	47
60	0	6	52
120	0	4	55

Table S27. Hydrolysis of **3A'** at pH 10.

*Hydrolysis of **3**$_G$' at pH 9*

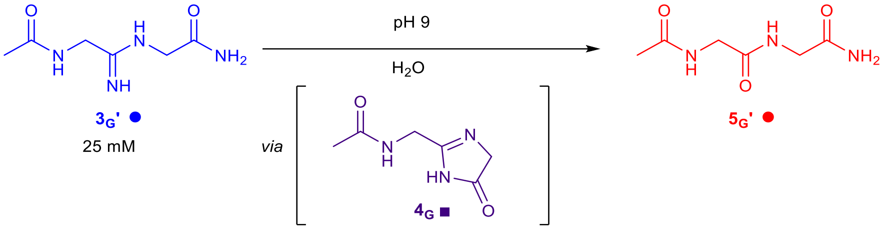

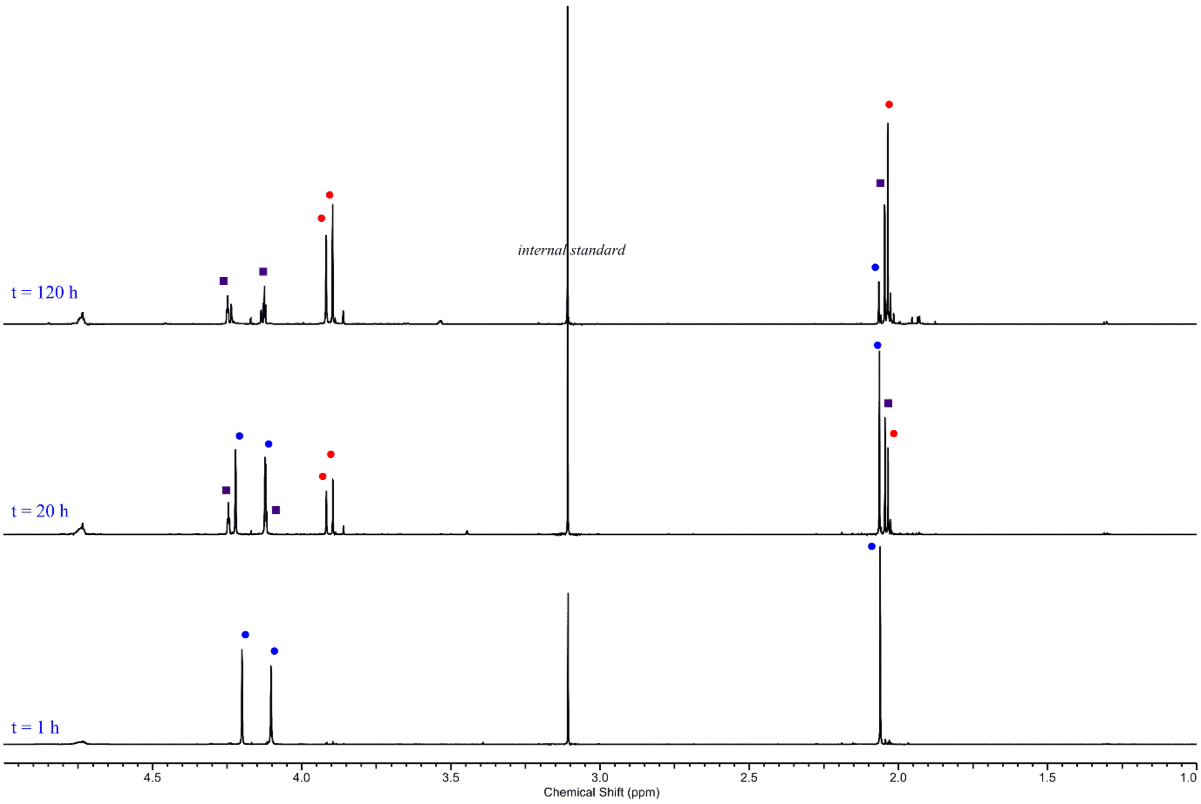

Figure S127. ^{1}H NMR (700 MHz, H_2O/D_2O 9:1, noesygppr1d, 1.0-5.0 ppm) spectra to show the hydrolysis of **3**$_G$' (25 mM) with MSM (30 mM) as an internal standard at pH 9.0 and room temperature.

time/ h	**3**$_G$' / %	**4**$_G$'/ %	**5**$_G$'/ %
1	86	4	2
4	77	10	6
8	69	16	10
14	57	22	15
24	43	28	20
36	34	30	25
48	27	31	29
81	18	27	36
118	10	22	40

Table S28. Hydrolysis of **3**$_G$' at pH 9

*Hydrolysis of **3_G’** at pH 9 in the presence of 3-mercaptopropionic acid **6b***

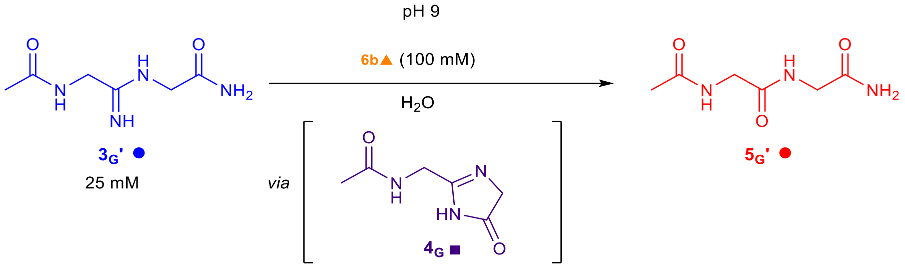

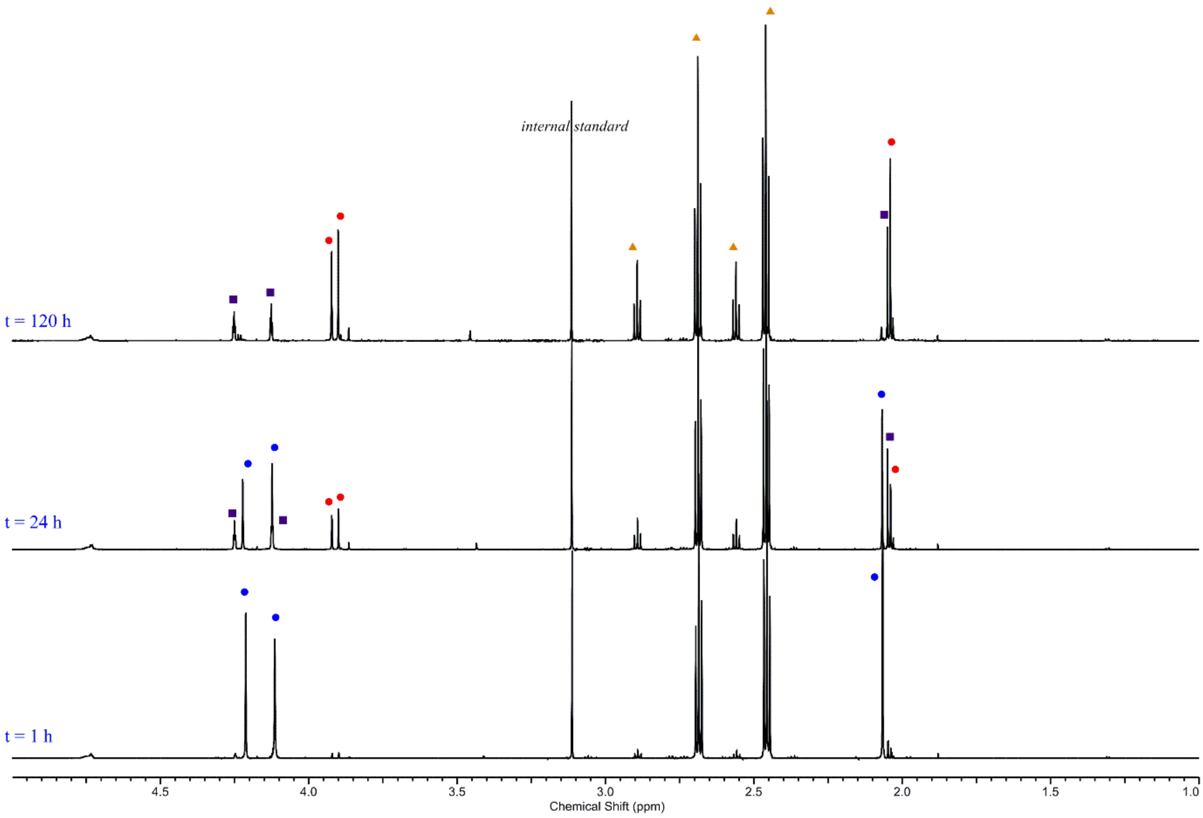

Figure S128. ^{1}H NMR (700 MHz, H_2O/D_2O 9:1, noesygppr1d, 1.0-5.0 ppm) spectra to show the hydrolysis of **3_G’** (25 mM) in the presence of 3-mercaptopropionic acid (**6b**, 100 mM) with MSM (30 mM) as an internal standard at pH 9.0 and room temperature.

time/ h	**3_G’** / %	**4_G’** / %	**5_G’** / %
1	95	7	3
4	89	11	6
8	77	18	10
14	63	25	14
24	46	33	21
36	32	39	28
48	23	39	32
118	10	37	43

Table S29. Hydrolysis of **3_G’** at pH 9 in the presence of 3-mercaptopropionic acid **6b**

*Hydrolysis of **3G'** at pH 9 in the presence of L-alanine (**2A**)*

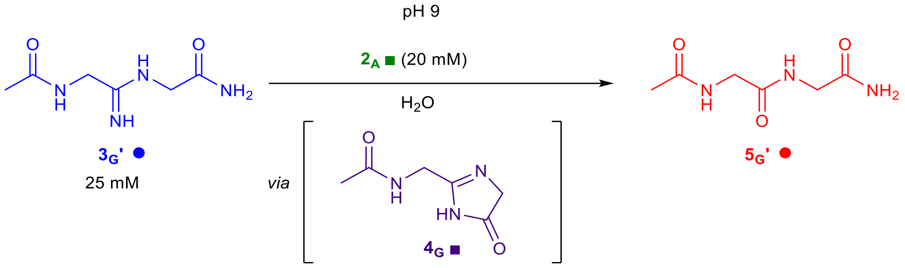

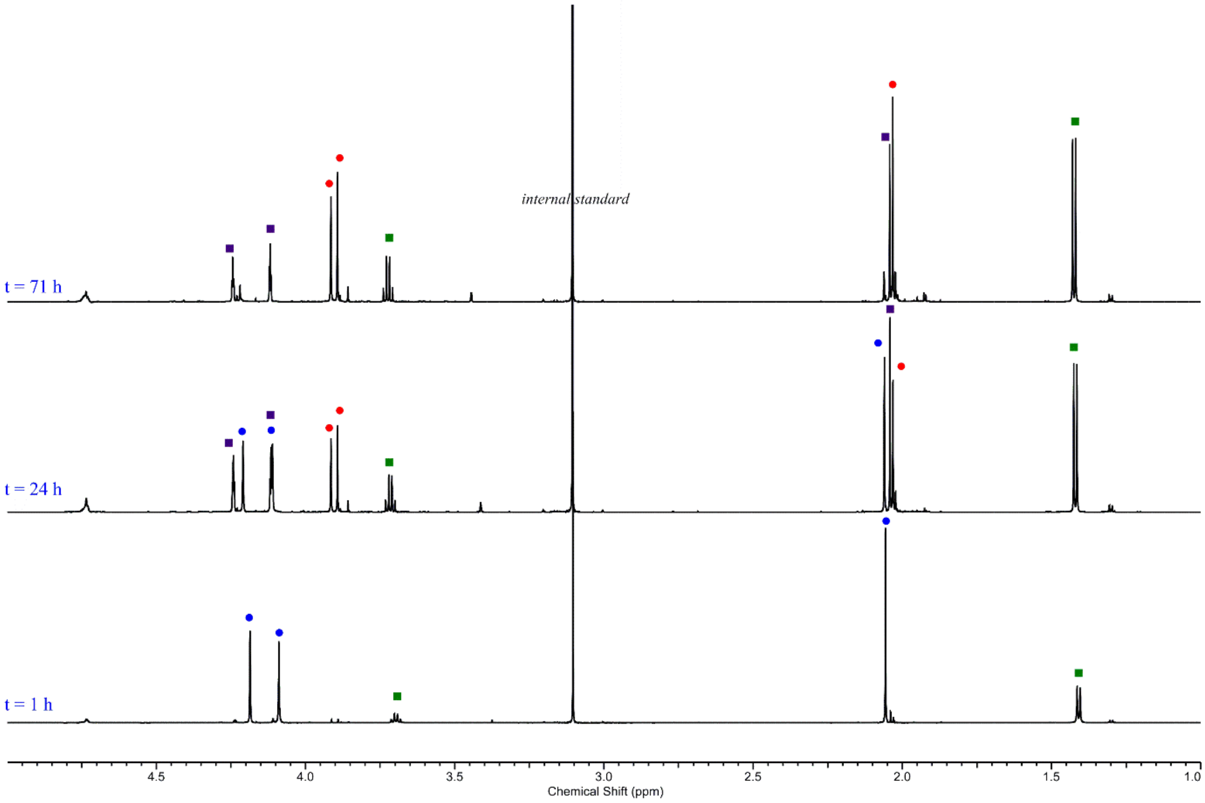

Figure S129. ^{1}H NMR (700 MHz, H_2O/D_2O 9:1, noesygppr1d, 1.0-5.0 ppm) spectra to show the hydrolysis of **3G'** (25 mM) in the presence of alanine (**2A**, 20 mM) with MSM (30 mM) as an internal standard at pH 9.0 and room temperature.

time/ h	**3G'** / %	**4G'** / %	**5G'** / %
1	87	6	3
3	76	14	7
7	62	23	12
15	43	32	20
24	30	37	25
35	20	36	31
48	14	34	35
71	9	29	42

Table S30. Hydrolysis of **3G'** at pH 9 in the presence of L-alanine (**2A**).

*Hydrolysis of **3$_G$'** at pH 9 in the presence of L-alaninamide (**2$_A$'**)*

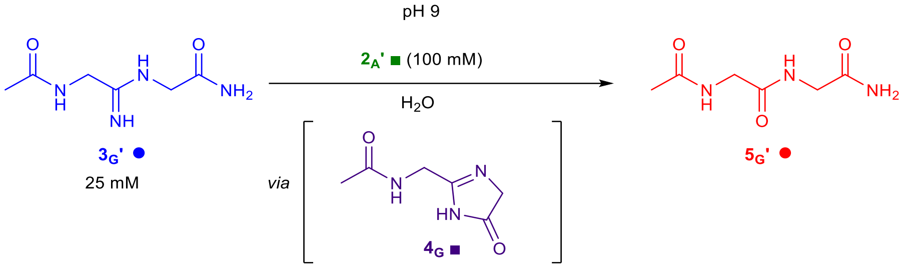

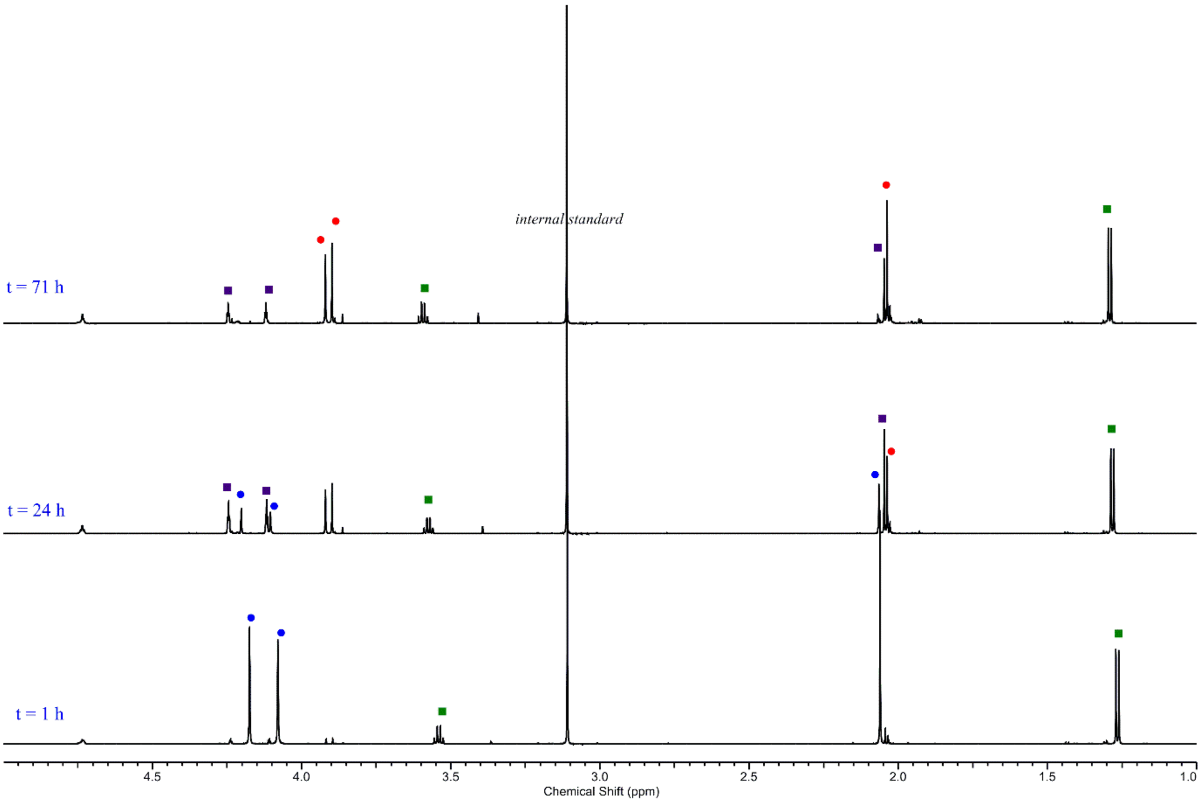

Figure S130. ^{1}H NMR (700 MHz, H_2O/D_2O 9:1, noesygppr1d, 1.0-5.0 ppm) spectra to show the hydrolysis of **3$_G$'** (25 mM) in the presence of alaninamide (**2$_A$'**, 20 mM) with MSM (30 mM) as an internal standard at pH 9.0 and room temperature.

time/ h	**3$_G$'** / %	**4$_G$'** / %	**3$_G$'** / %
1	90	6	2
3	73	18	10
7	54	28	16
15	33	37	24
24	22	42	30
35	13	41	36
48	10	36	41
71	3	34	49

Table S31. Hydrolysis of **3$_G$'** at pH 9 in the presence of L-alaninamide (**2$_A$'**).

No incorporation of either alanine **2$_A$** or alaninamide **2$_A$'** was observed.

*Hydrolysis of **3**$_A$*

*Hydrolysis of **3**$_A$ at pH 7*

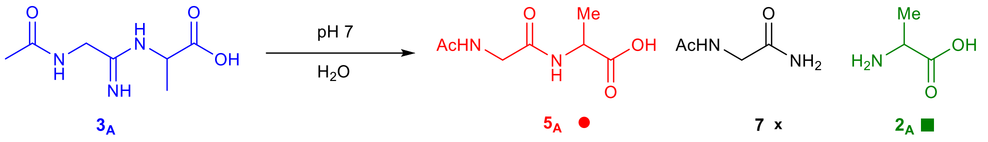

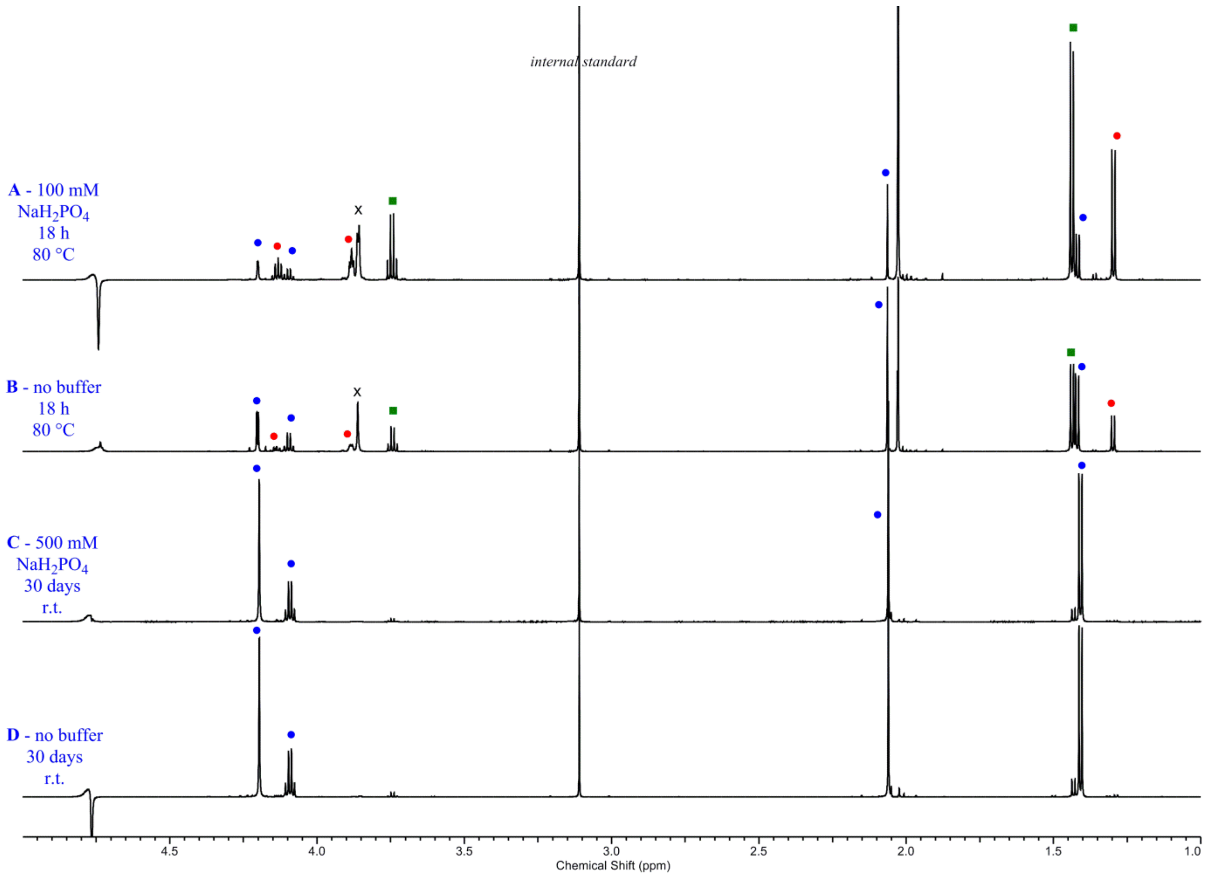

Figure S131. ^1H NMR (700 MHz, H_2O/D_2O 9:1, noesygppr1d, 1.0-5.0 ppm) spectra to show the hydrolysis of **3$_A$** (25 mM) at pH 7.0 **A** after 18 at 80°C in phosphate buffer (100 mM); **B** after 18 h at 80°C; **C** after 30 days at room temperature in phosphate buffer (500 mM).

*Hydrolysis of **3***

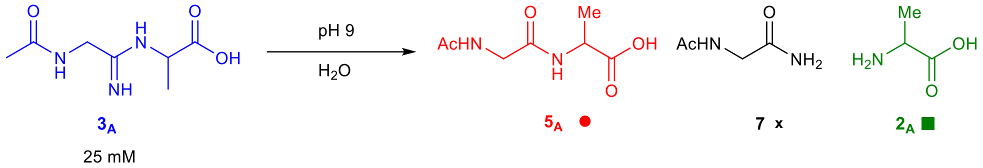

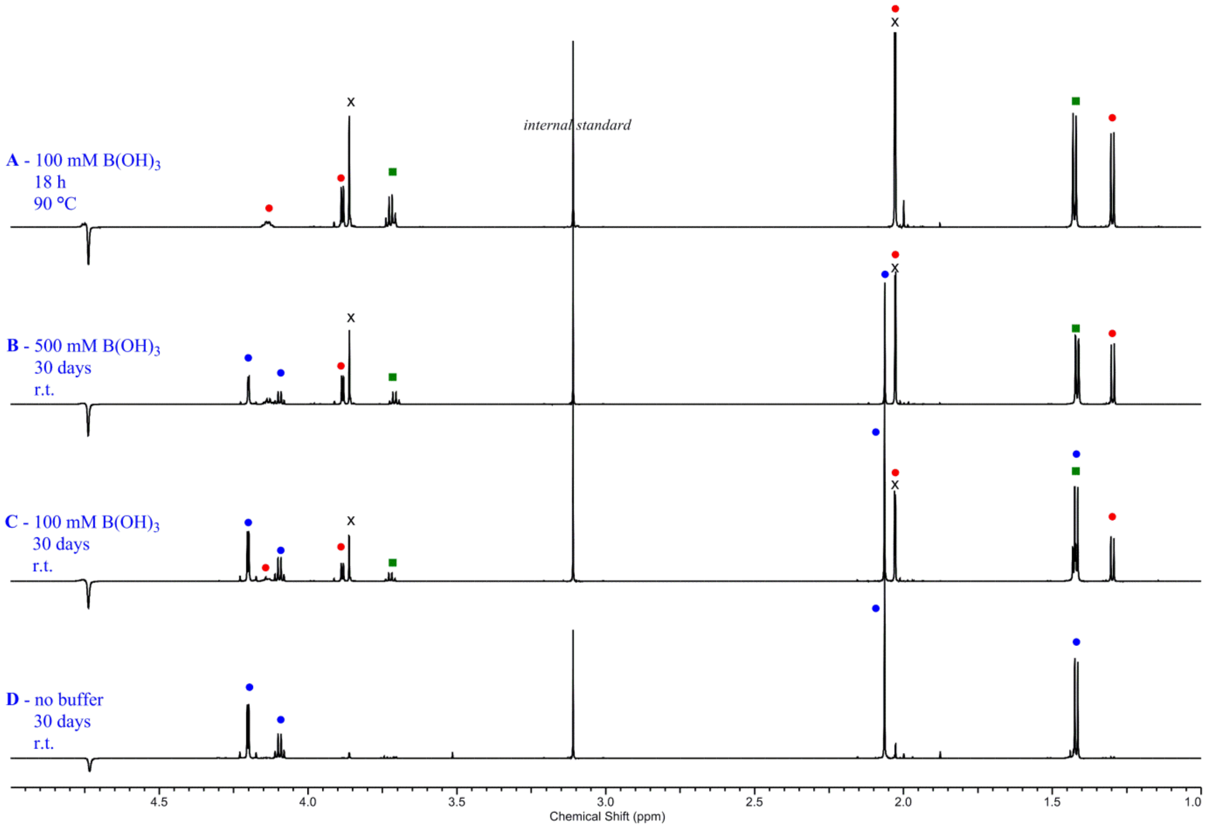

Figure S132. ^{1}H NMR (700 MHz, H_2O/D_2O 9:1, noesygppr1d, 1.0-5.0 ppm) spectra to show the hydrolysis of $\mathbf{3_A}$ (25 mM) at pH 9.0 **A** after 18 at 80 °C in the presence of 100 mM $B(OH)_3$; **B** after 30 days at room temperature in borate buffer (500 mM); **C** after 30 days at room temperature in borate buffer (100 mM); **D** after 30 days at room temperature

*Coupling of N-acetylglycine nitrile **1** with L-lysinamide **2$_K$'** at pH 8.5 and room temperature*

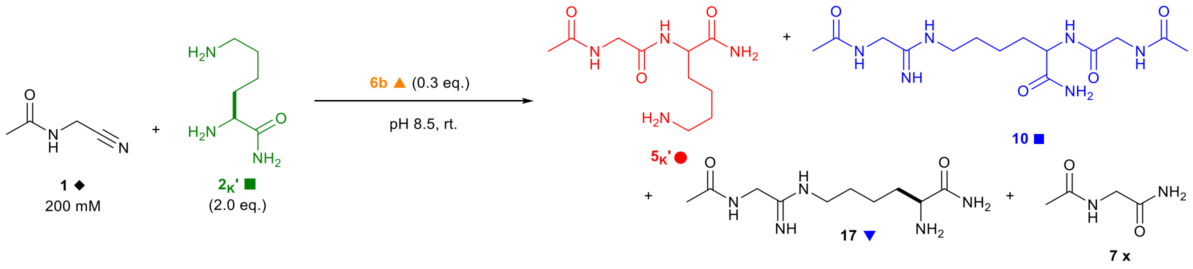

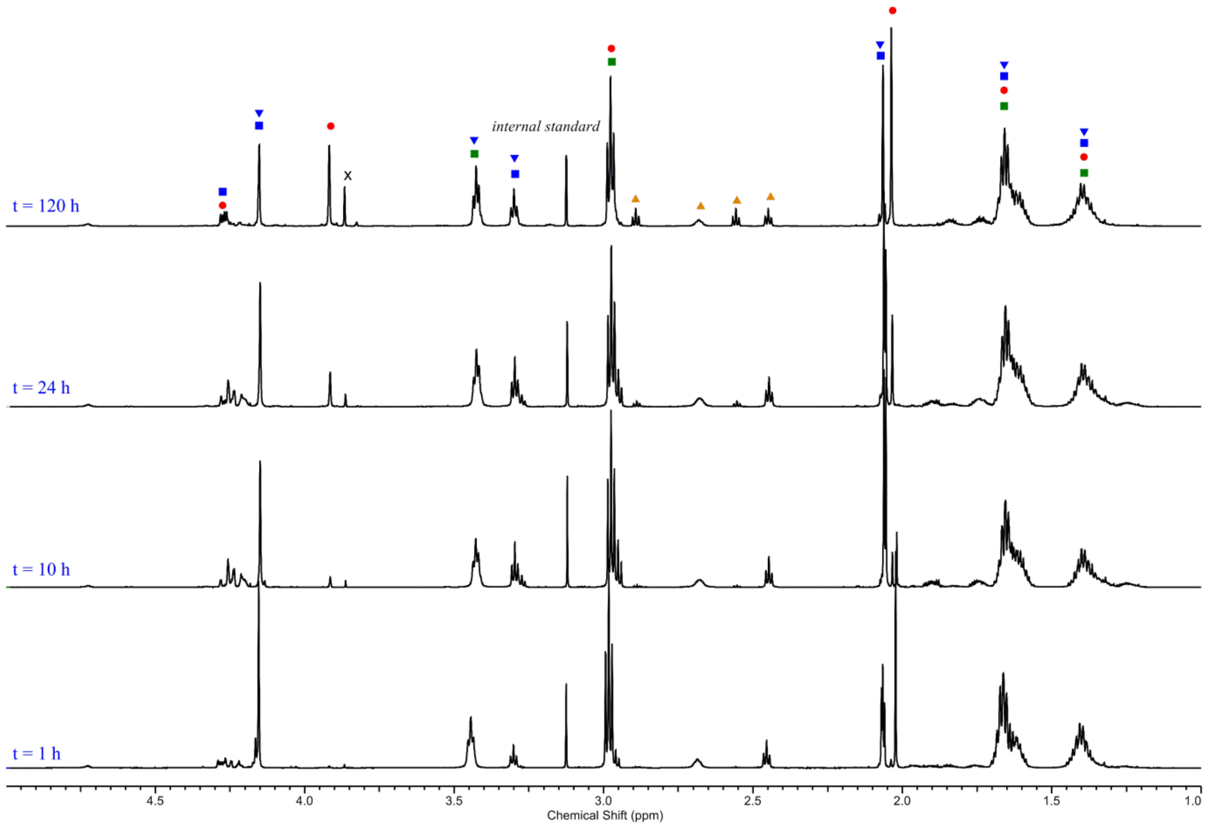

Figure S133: ^{1}H NMR (700 MHz, H_2O/D_2O 9:1, noesygppr1d, 1.0-5.0 ppm) spectrum to show the reaction of N-acetyl glycine nitrile (**1**, 200 mM) with L-lysinamide (**2$_K$'**, 400 mM) and 3-mercaptopropanoic acid (**6b**, 60 mM) with MSM (7 mM) as an internal standard at pH 8.5 and room temperature.

^{1}H NMR (700 MHz, H_2O/D_2O 9:1) α-*peptide* (partial assignment) (●, ■): δ_H 4.29 (1H, dd, *J* = 9.4, 4.9 Hz, Lys-α-C***H***CONH$_2$), 3.94 (2H, s, C***H$_2$***CONH), 2.06 (3H, s, ***H$_3$***C(CO)); *ε-amidine* (partial assignment) (■, ▼): δ_H 4.18 (2H, s, C***H$_2$***C(NH)NH), 3.32 (2H, t, *J* = 7.0 Hz, Lys–ε-C***H$_2$***NH$_2$), 2.09 (3H, s, ***H$_3$***C(CO)); *L-lysinamide*, **2$_K$'** (partial assignment) (■): δ_H 3.45 (1H, t, *J* = 6.4 Hz, C***H***CONH$_2$), 3.00 (2H, t, *J* = 7.5 Hz, ε-C***H$_2$***NH$_2$); *N-acetylglycinamide*, **7** (✖) (partial assignment): δ_H 3.89 (2H, d, C*H$_2$*).

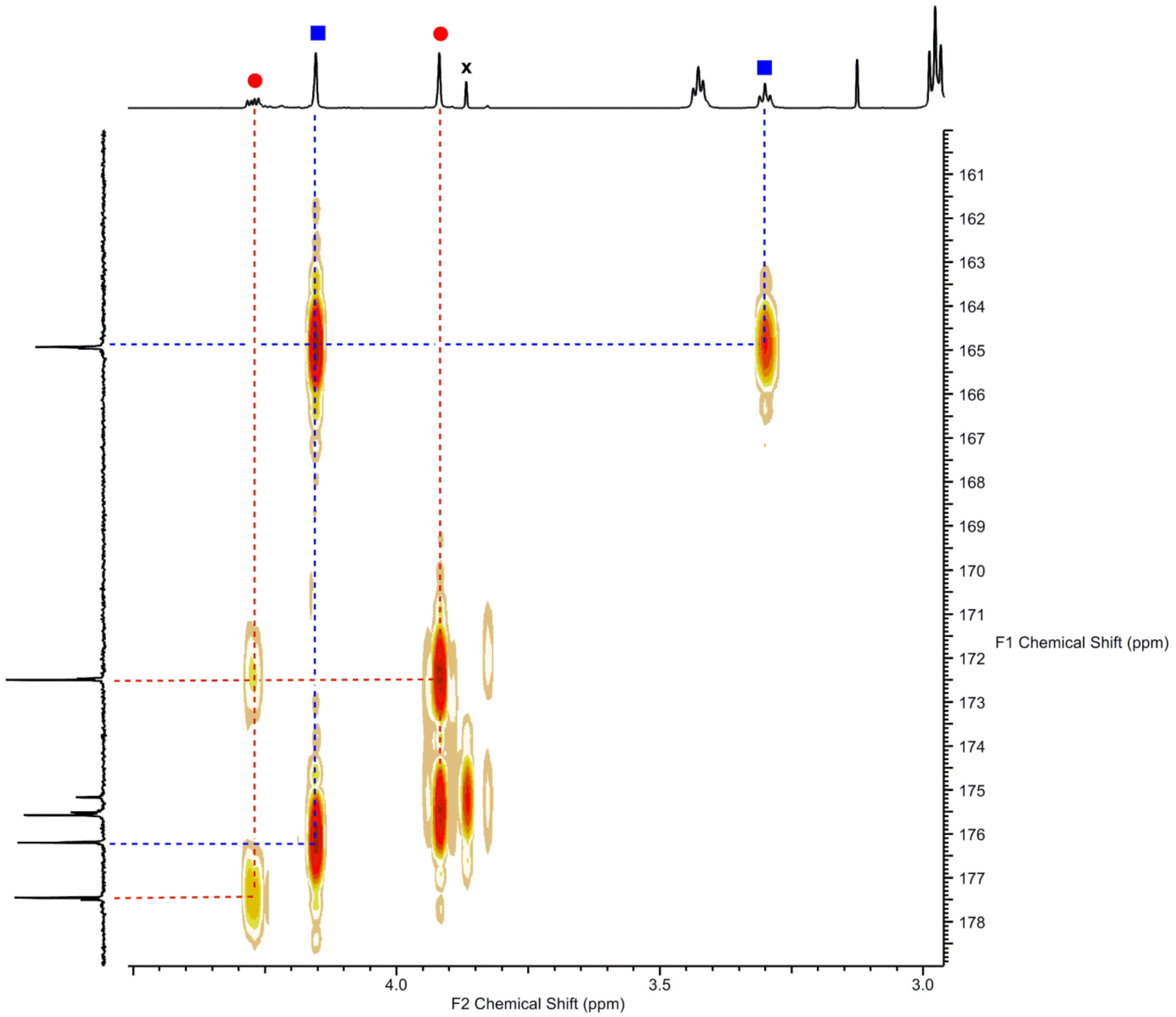

Figure S134: ^{1}H–^{13}C HMBC (^{1}H: 700 MHz [3.9-4.5 ppm], ^{13}C: 176 MHz [160-180 ppm], H_2O/D_2O 9:1) spectrum showing the $^{2}J_{CH}$ and $^{3}J_{CH}$ coupling of the **Lys**-αH in **5K'** and **10** at 4.29 ppm with resonances at 172.5 and 177.4 ppm, characteristic of peptide formation, alongside the $^{3}J_{CH}$ coupling of the **Lys**-εH2 in **10** and **17** at 3.32 ppm with a resonance at 164.9 ppm, characteristic of amidine formation.

Entry	**α-amide (5K' + 10)** / %	**ε-amide (10 + 17)** / %	**7**/ %
1	42	37	16

Table S32. Coupling of N-acetylglycine nitrile **1** with L-lysinamide **2K'** at pH 8.5 and room temperature.

*Coupling of N-acetylglycine nitrile **1** with L-lysinamide **2K'** at pH 9.0, 80 °C*

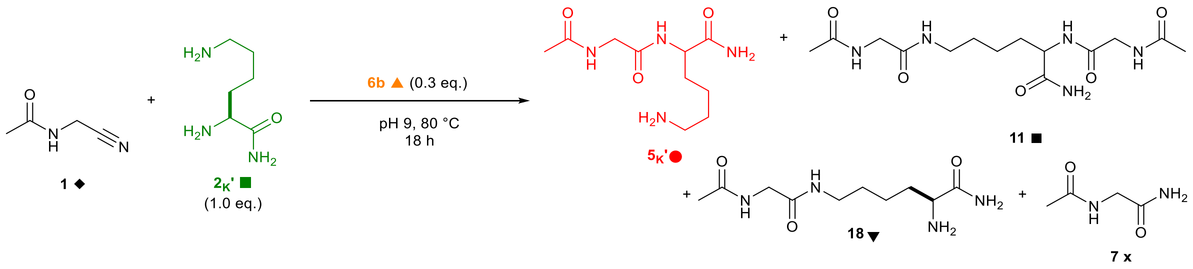

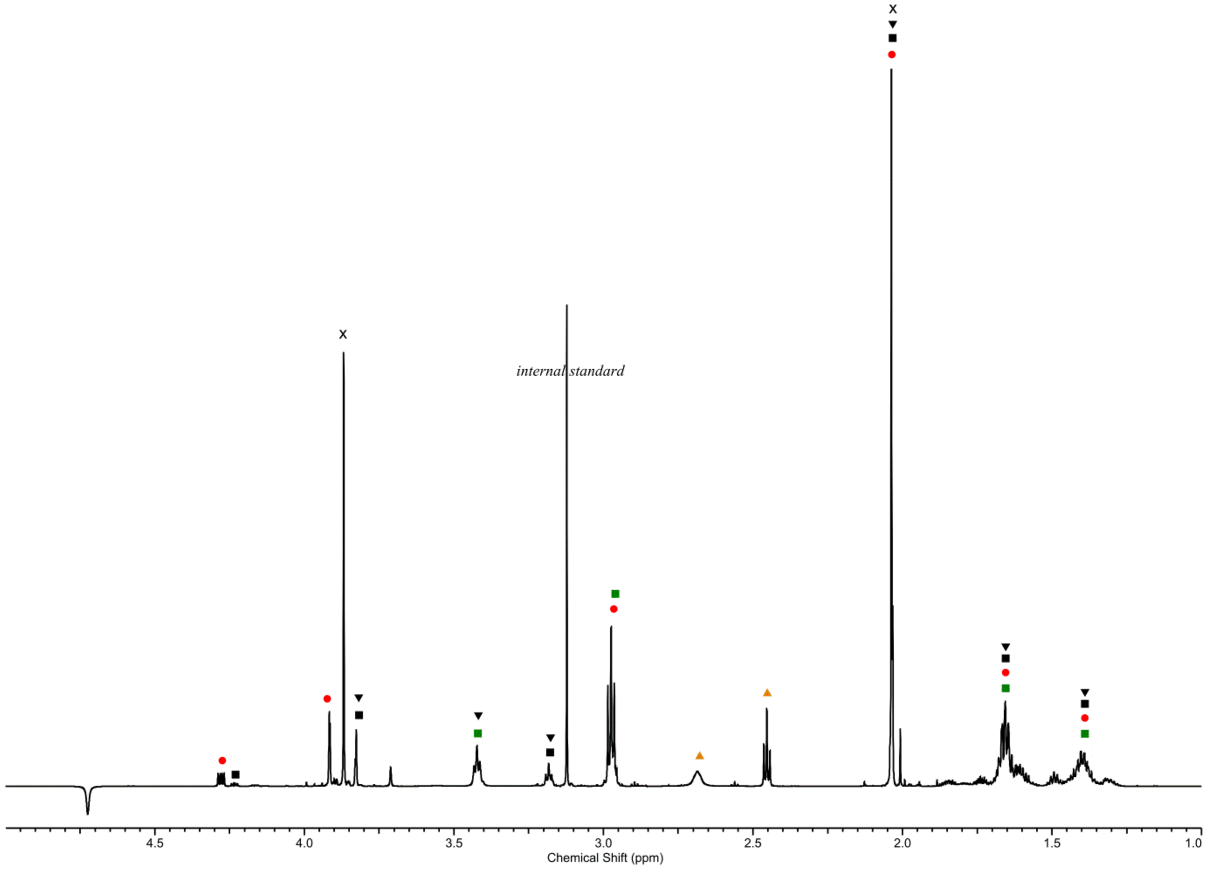

Figure S135: ^{1}H NMR (700 MHz, H_2O/D_2O 9:1, noesygppr1d, 1.0-5.0 ppm) spectrum to show the reaction of N-acetyl glycine nitrile (**1**, 200 mM) with L-lysinamide (**2K'**, 200 mM) and 3-mercaptopropanoic acid (**6b**, 60 mM) with MSM (17 mM) as an internal standard at pH 9.0 and 80°C after 18 h.

ε-amide (partial assignment) (■, ▼): δ_H 3.85 (2H, s, C***H***$_2$C(NH)NH), 3.21 (1H, t, *J* = 7.0 Hz, Lys–ε-C***H***$_2$NH$_2$), 2.09 (3H, s, ***H***$_3$C(CO)).

Entry	**α-amide (5K' + 11)** / %	**ε-amide (11 + 17)** / %	**7**/ %
1	20	15	59

Table S33. Coupling of N-acetylglycine nitrile **1** with L-lysinamide **2K'** at pH 9.0, 80 °C

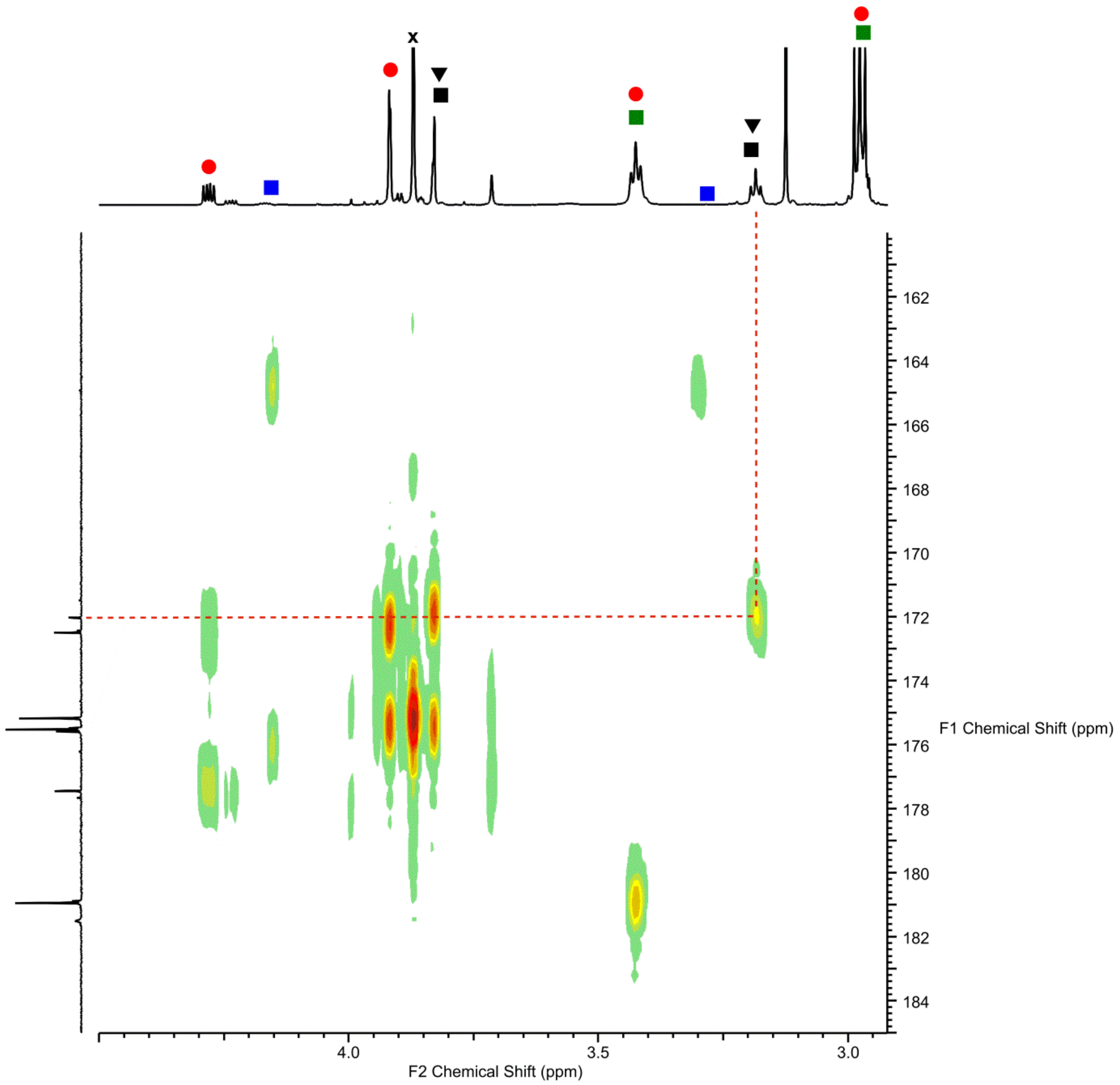

Figure S136: ^1H–^{13}C HMBC (^1H: 700 MHz [3.9-4.5 ppm], ^{13}C: 176 MHz [160-180 ppm], H_2O/D_2O 9:1) spectrum showing the $^3J_{CH}$ coupling of the **Lys**-εH_2 in **11** and **18** at 3.21 ppm with a resonance at 164.9 ppm, characteristic of amide formation.

*Coupling of N-acetylglycine nitrile **1** with L-lysinamide **2K'** at pH 7.0, 80°C*

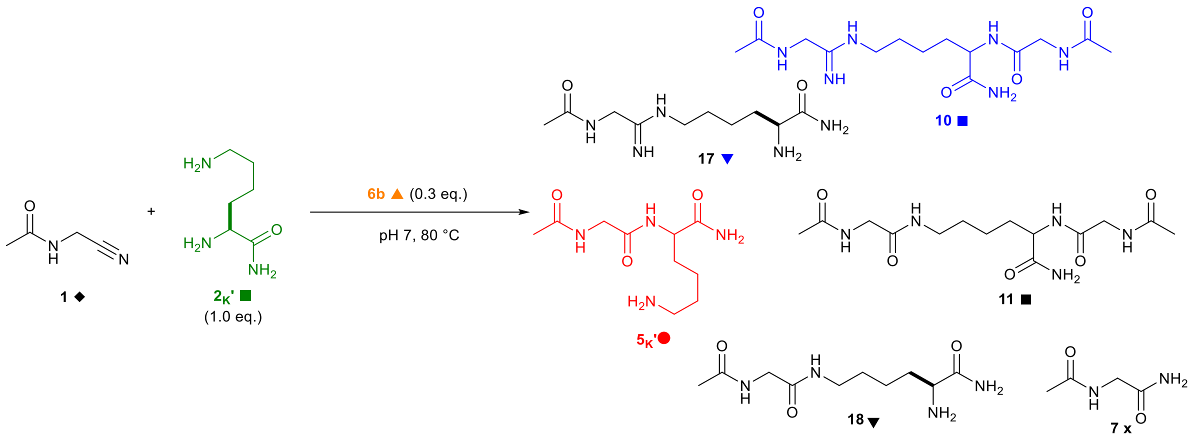

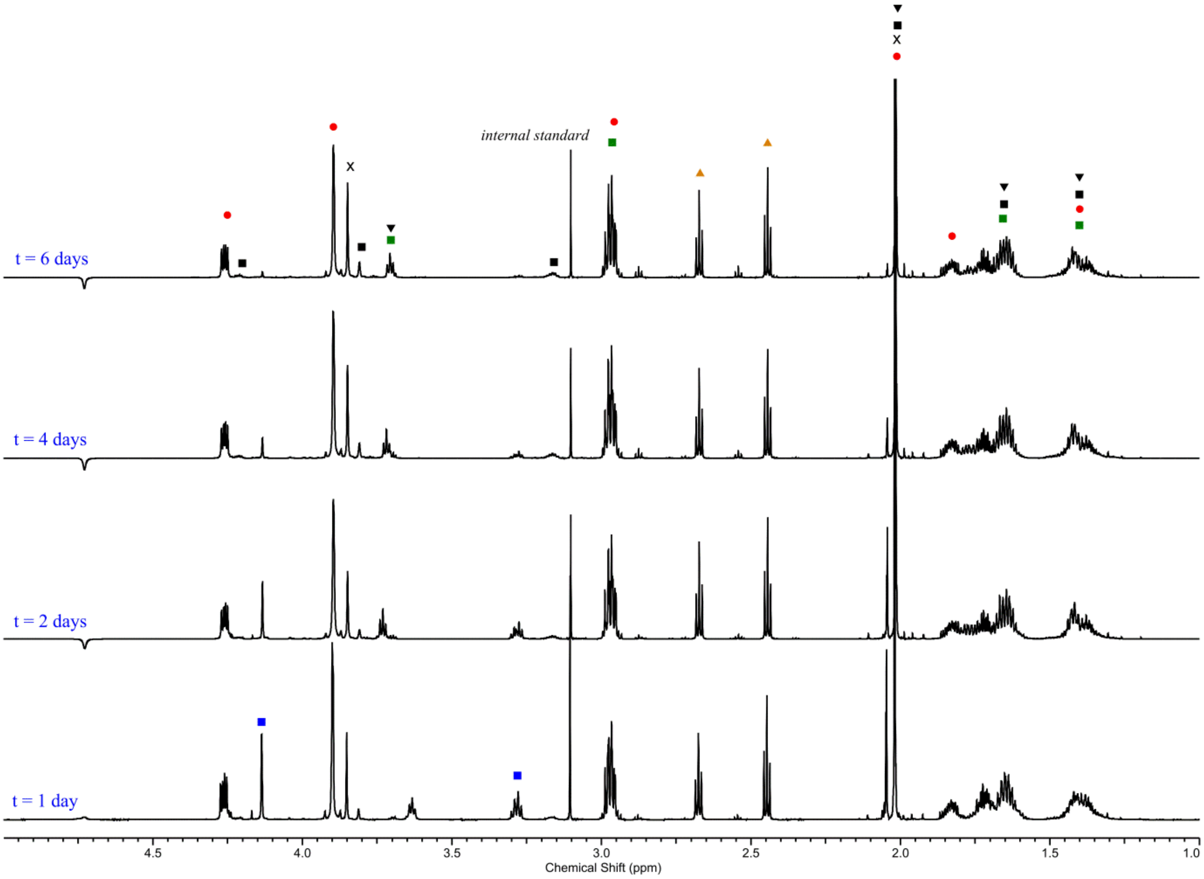

Figure S137: ^{1}H NMR (700 MHz, H_2O/D_2O 9:1, noesygppr1d, 1.0-5.0 ppm) spectrum to show the reaction of N-acetyl glycine nitrile (**1**, 200 mM) with L-lysinamide (**2K'**, 200 mM) and 3-mercaptopropanoic acid (**6b**, 60 mM) with MSM (17 mM) as an internal standard at pH 9.0 and 80°C.

Entry	time / days	α-amide (5K' + 10 + 11) / %	ε-amidine (10 + 17) / %	ε-amide (11 + 18) / %	7/ %
1	1	67	19	trace	16
2	2	68	14	4	19
3	4	68	5	6	24
4	6	65	trace	7	25

Table S34. Coupling of N-acetylglycine nitrile **1** with L-lysinamide **2K'** at pH 7.0, 80 °C

*Coupling of N-acetylglycine nitrile **1** with L-lysylglycine **2**$_{KG}$ at pH 7.0, 80 °C*

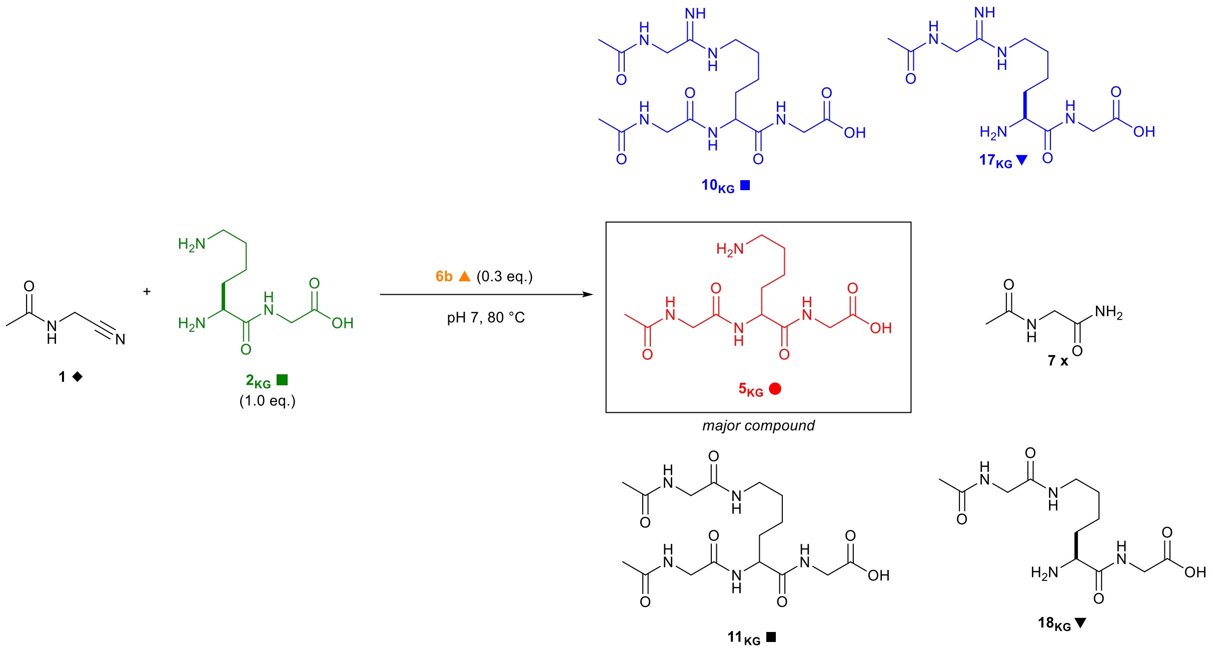

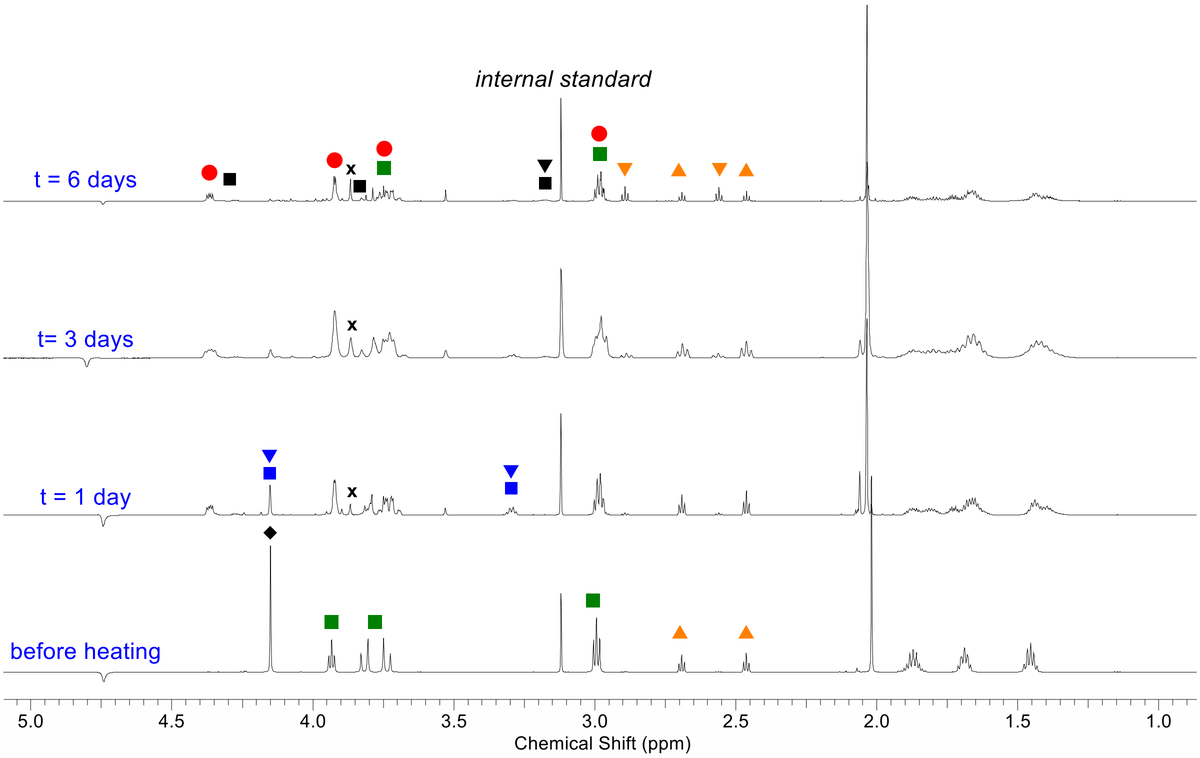

***Figure S138**.* ^{1}H NMR (700 MHz, H_2O/D_2O 9:1, noesygppr1d, 1.0-5.0 ppm) spectrum to show the reaction of N-acetyl glycine nitrile (**1**, 200 mM) with L-lysylglycine (**2**$_{KG}$, 200 mM) and 3-mercaptopropanoic acid (**6b**, 60 mM, 0.3 eq.) with MSM (40 mM) as the internal standard at pH 7.0 and 80 °C. ▼ = 2-carboxyethyldisulfide (formed by aerial oxidation of **6b**).

^{1}H NMR (700 MHz, H_2O/D_2O 9:1) *a-peptide* (partial assignment) (●, ■, ■): δ_H 4.36 (1H, dd, *J* = 9.2, 4.9 Hz, Lys-α-C***H***CONHGlyOH), 3.93 (1H, AB, *J* = 17.3 Hz, C***H***CONHLysOH), 3.91 (1H, AB, *J* = 17.3 Hz, C***H'***CONHLysOH), 3.71 (1H, ABX, *J* = 17.3, 4.0 Hz, LysC***H***CO_2H), 2.03 (3H, s, ***H***$_3$C(CO)); *ε-amidine* (partial assignment) (■, ▼): δ_H 4.15 (2H, s, C***H***$_2$C(NH)NH), 2.06 (3H, s, ***H***$_3$C(CO)); *LL-lysylglycine*, **2**$_{KG}$ (partial assignment) (■): δ_H 3.80 (1H, AB, *J* = 17.3 Hz, LysC***H***CO_2H); *N-Acetylglycinamide*, **7** (**x**) (partial assignment): δ_H 3.87 (2H, s, C*H*$_2$). HRMS (ESI) m/z for **5**$_{KG}$ $[C_{12}H_{23}N_4O_5]^+$: calcd 303.1663, found 303.1661.

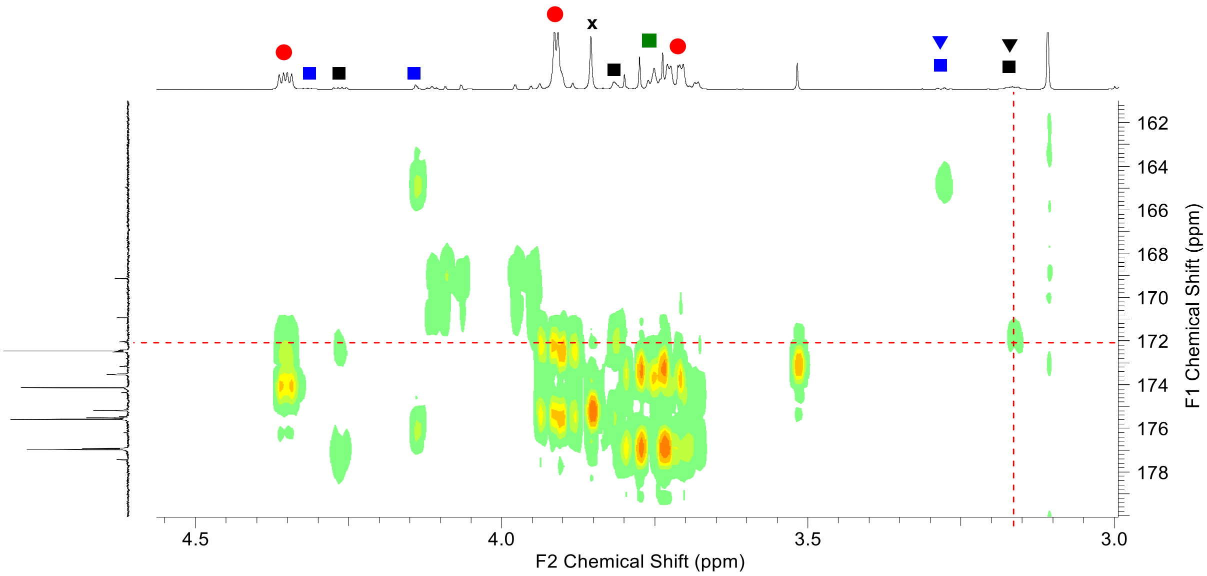

Figure S139. ^1H–^{13}C HMBC (^1H: 700 MHz [3.0-4.6 ppm], ^{13}C: 176 MHz [162-180 ppm], H_2O/D_2O 9:1) spectrum showing the $^3J_{CH}$ coupling of the Lys-ε**H**$_2$ in **11**$_{KG}$ and **18**$_{KG}$ at 3.18 ppm with a resonance at 172 ppm, characteristic of amide formation.

Entry	**time / days**	**α-amide (5$_{KG}$ + 10$_{KG}$ + 11$_{KG}$)** / %	**ε-amidine (10$_{KG}$ + 17$_{KG}$) / %**	**ε-amide (11$_{KG}$ + 18$_{KG}$)** / %
1	1	55	17	2
2	3	50	7	4
3	6	59	2	6

Table S35. Coupling of N-acetylglycine nitrile **1** with L-lysylglycine **2**$_{KG}$ at pH 7.0, 80 °C. **7** could not be accurately quantified due to overlap with glycyl peaks.

*Coupling of N-acetylglycine nitrile **1** with L-lysyl-L-lysine **2_{KK}** at pH 7.0, 80 °C*

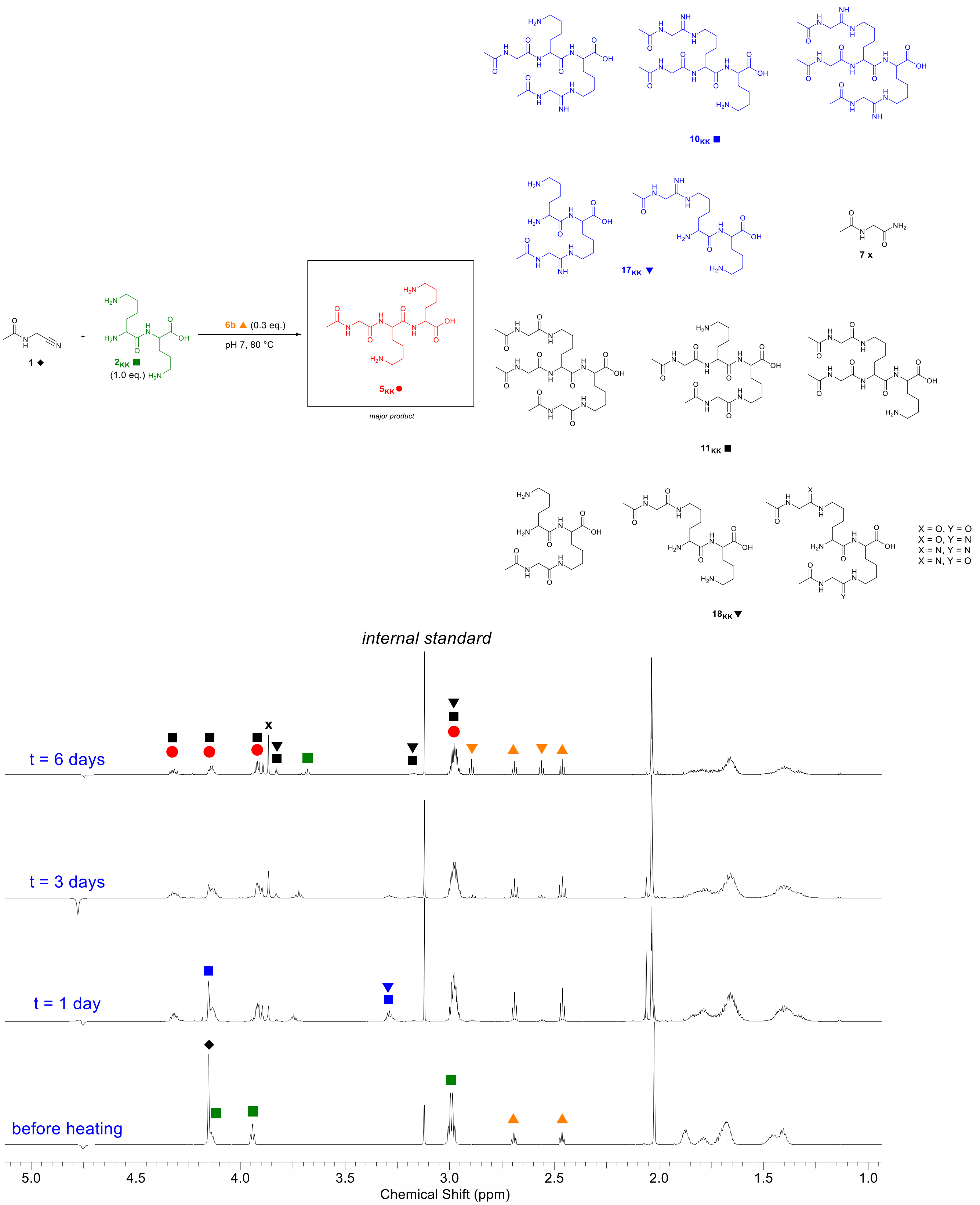

Figure S140: ^{1}H NMR (700 MHz, H_2O/D_2O 9:1, noesygppr1d, 1.0-5.0 ppm) spectrum to show the reaction of N-acetyl glycine nitrile (**1**, 200

mM) with LL-lysyllysine (**2**$_{KK}$, 200 mM) and 3-mercaptopropanoic acid (**6b**, 60 mM, 0.3 eq.) with MSM (33 mM) as the internal standard at pH 7.0 and 80 °C. ▼ = 2-carboxyethyldisulfide (formed by aerial oxidation of **6b**).

^{1}H NMR (700 MHz, H_2O/D_2O 9:1) *α-peptide* (partial assignment, mixture of diastereomers) (●, ■): δ_H 4.31 (1H, overlapping dd, Lys-α-C***H***CONHLysOH), 3.93 (1H, AB, *J* = 16.8 Hz, C***H***CONH), 3.91 (1H, AB, *J* = 16.8 Hz, C***H***CONH), 2.03 (3H, s, ***H***$_3$C(CO)); *ε-amidine* (partial assignment, mixture of diastereomers) (■, ▼): δ_H 4.15 (2H, s, C***H***$_2$C(NH)NH), 2.06 (3H, s, ***H***$_3$C(CO)); *LL-lysyllysine*, **2**$_{KK}$ (partial assignment) (■): δ_H 3.68 (1H, t, *J* = 6.6 Hz, C***H***CONH$_2$); *N-Acetylglycinamide*, **7** (**x**) (partial assignment): δ_H 3.87 (2H, s, C*H*$_2$). HRMS (ESI) m/z for **5**$_{KK}$ $[C_{16}H_{32}N_5O_5]^+$: calcd 374.23980, found 374.2398.

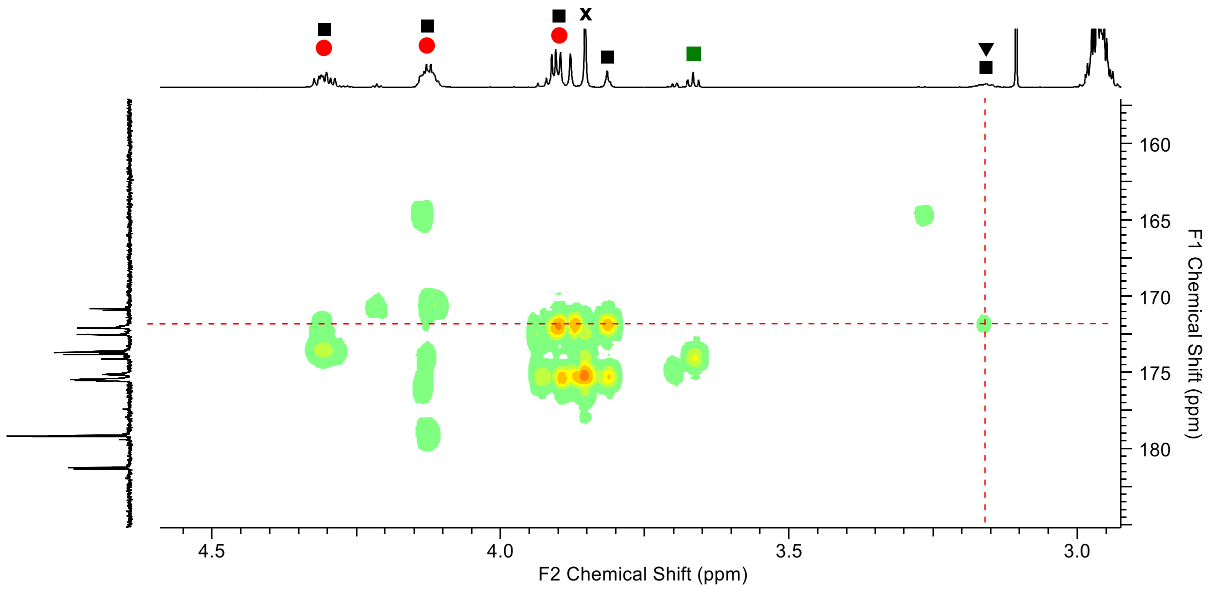

Figure S141. ^{1}H–^{13}C HMBC (^{1}H: 700 MHz [2.9-4.6 ppm], ^{13}C: 176 MHz [160-185 ppm], H_2O/D_2O 9:1) spectrum showing the $^3J_{CH}$ coupling of the **Lys**-εH_2 in **11**$_{KK}$ and **18**$_{KK}$ at 3.21 ppm with a resonance at 172 ppm, characteristic of amide formation.

Entry	time / days	α-amide (**5**$_{KK}$ + **10**$_{KK}$ + **11**$_{KK}$) / %	ε-amidine (**10**$_{KK}$ + **17**$_{KK}$) / %	ε-amide (**11**$_{KK}$ + **18**$_{KK}$) / %
1	1	63	33	3
2	3	62	13	9
3	6	70	2	13

Table S36. Coupling of N-acetylglycine nitrile **1** with LL-lysyllysine **2**$_{KK}$ at pH 7.0, 80 °C. **7** could not be accurately quantified due to overlap with glycyl peaks.

*Coupling of N-acetylglycine nitrile **1** with O-methyl serinamide **2_{MeS}'** catalyzed by **6b***

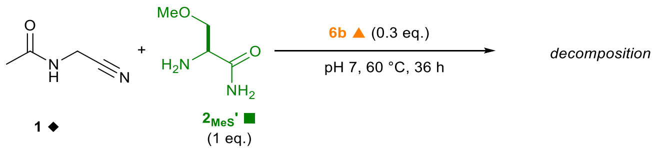

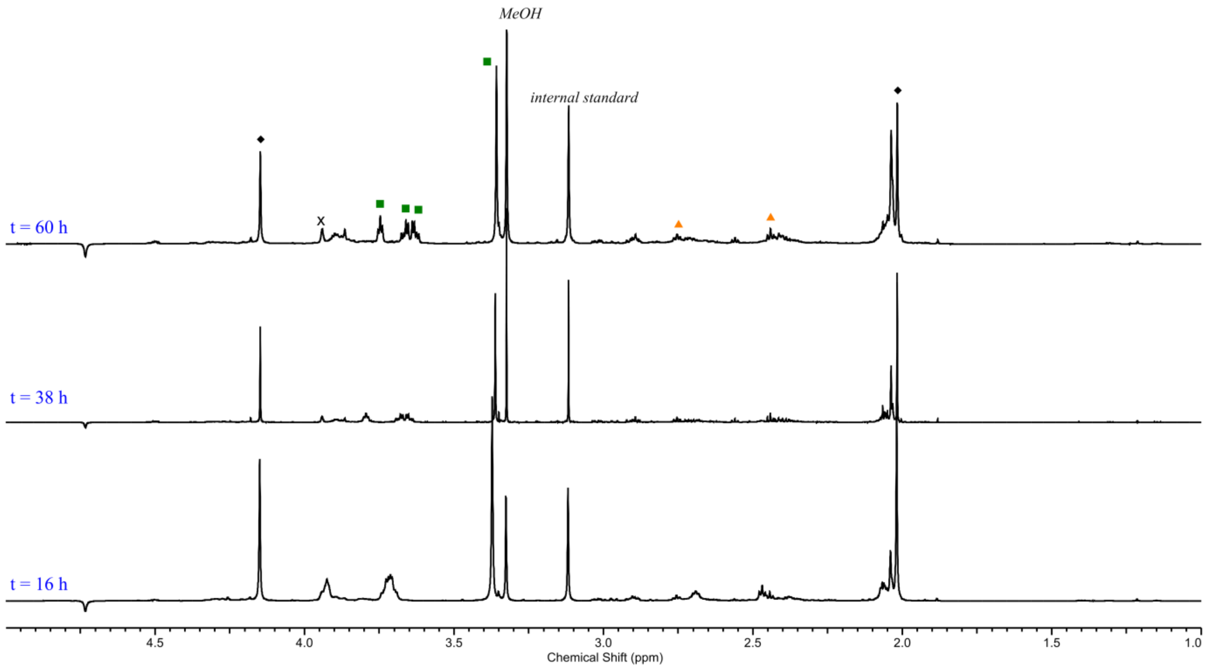

Figure S142: ^{1}H NMR (700 MHz, H_2O/D_2O 9:1, noesygppr1d, 1.0-5.0 ppm) spectrum to show the reaction of N-acetyl glycine nitrile (**1**, 200 mM) with O-methyl serinamide hydrochloride (**2_{MeS}'**, 200 mM) and 3-mercaptopropanoic acid (**6b**, 60 mM, 0.3 eq.) with MSM (33 mM) as the internal standard at pH 7.0 and 60 °C.

Coupling between **1** and **2_{MeS}'** at pH 7 and 60 °C shows a similar result to coupling between **1** and **2_S'** at pH 8.5 (Fig. S88). The methyl group prevents oxazoline formation which enables serinamide **2_S'** to form peptides in high yield at neutral pH (Fig. S89).

---

[1] Covington, A. K., Paabo, M., Robinson, R. A. & Bates, R. G. Use of the glass electrode in deuterium oxide and the relation between the standardized pD (paD) scale and the operational pH in heavy water. *Anal. Chem.* **1968**, 40, 700-706.
[2] Kohn, H.; Hyup Lee, S. Phosphine-Assisted Rearrangement of 4,5-Dihydroxy-1,2-Dithianes to 4-Hydroxy-3-Mercaptotetrahydrothiophenes. *Heterocycles* **2003**, *60*, 47-56.
[3] Canavelli, P.; Islam, S.; Powner, M. W., Peptide ligation by chemoselective aminonitrile coupling in water. *Nature* **2019**, *571*, 546-549.
